# An Efficient and Comprehensive Strategy for Genetic Diagnostics of Polycystic Kidney Disease

**DOI:** 10.1371/journal.pone.0116680

**Published:** 2015-02-03

**Authors:** Tobias Eisenberger, Christian Decker, Milan Hiersche, Ruben C. Hamann, Eva Decker, Steffen Neuber, Valeska Frank, Hanno J. Bolz, Henry Fehrenbach, Lars Pape, Burkhard Toenshoff, Christoph Mache, Kay Latta, Carsten Bergmann

**Affiliations:** 1 Bioscientia Center for Human Genetics, Ingelheim, Germany; 2 Department of Pediatrics, Children’s Hospital Memmingen, Memmingen, Germany; 3 Clinic for Pediatric Kidney, Liver and Metabolic Diseases, MHH Hannover, Germany; 4 Department of Pediatrics I, University Children’s Hospital Heidelberg, Germany; 5 Department of Pediatrics, Medical University Graz, Austria; 6 Clementine Children’s Hospital, Frankfurt, Germany; 7 Renal Division, Department of Medicine, University Freiburg Medical Center, Freiburg, Germany; Leiden University Medical Center, NETHERLANDS

## Abstract

Renal cysts are clinically and genetically heterogeneous conditions. Autosomal dominant polycystic kidney disease (ADPKD) is the most frequent life-threatening genetic disease and mainly caused by mutations in *PKD1*. The presence of six *PKD1* pseudogenes and tremendous allelic heterogeneity make molecular genetic testing challenging requiring laborious locus-specific amplification. Increasing evidence suggests a major role for *PKD1* in early and severe cases of ADPKD and some patients with a recessive form. Furthermore it is becoming obvious that clinical manifestations can be mimicked by mutations in a number of other genes with the necessity for broader genetic testing. We established and validated a sequence capture based NGS testing approach for all genes known for cystic and polycystic kidney disease including *PKD1*. Thereby, we demonstrate that the applied standard mapping algorithm specifically aligns reads to the *PKD1* locus and overcomes the complication of unspecific capture of pseudogenes. Employing careful and experienced assessment of NGS data, the method is shown to be very specific and equally sensitive as established methods. An additional advantage over conventional Sanger sequencing is the detection of copy number variations (CNVs). Sophisticated bioinformatic read simulation increased the high analytical depth of the validation study and further demonstrated the strength of the approach. We further raise some awareness of limitations and pitfalls of common NGS workflows when applied in complex regions like *PKD1* demonstrating that quality of NGS needs more than high coverage of the target region. By this, we propose a time- and cost-efficient diagnostic strategy for comprehensive molecular genetic testing of polycystic kidney disease which is highly automatable and will be of particular value when therapeutic options for PKD emerge and genetic testing is needed for larger numbers of patients.

## Introduction

An emerging number of clinically and genetically heterogeneous diseases is related to the dysfunction of cilia (collectively termed “ciliopathies”) [1,2]. Virtually all ciliopathies have a renal cystogenic component. Important cystic kidney disorders are autosomal dominant (ADPKD), autosomal recessive polycystic kidney disease (ARPKD) and nephronophthisis (NPH) [3–5] ADPKD is one of the most common Mendelian disorders with a prevalence of 1/400 to 1/1000, and typically a late-onset disease with mutations in *PKD1* or *PKD2* that can also be expressed as a recessive trait. While the kidney is the main organ involved, there can be a profound extrarenal disease burden (e. g. liver and intracranial aneurysms).

Due to the size and structure of major PKD genes as well as marked clinical and genetic heterogeneity with most mutations being private in single families genetic testing is cumbersome. In particular, analysis of the *PKD1* gene is complicated by the presence of six pseudogenes (*PKD1P1-P6*) on chromosome 16 with 97.7% sequence identity as a consequence of segmental duplications of the main part of the 5’ genomic regions (exons 1–33) [6,7]. Therefore, conventional routine diagnostic testing employs laborious locus-specific amplification by long-range PCR (LR-PCR) [8] followed by Sanger sequencing, which is expensive with limited capacities and only allows for a stepwise approach. Next-generation sequencing (NGS) has revolutionized genetic diagnostics and allows for parallel sequencing of all disease genes that have to be considered in a given patient. Recently, Rossetti et al. have adopted amplicon-based NGS restricted to *PKD1* and *PKD2* and still dependent on laborious LR-PCR [9]. Genome partitioning approaches like capture-by-hybridization have been developed as an elegant and due to a multiplexing option cost-effective method of targeted re-sequencing [10]. However, due to risk of concurrent capture of pseudogene sequence this enrichment approach was speculated not be applicable for the duplicated region of *PKD1* [9].

By detection of copy-number variations (CNVs) NGS may not only improve the throughput, but also the quality of genetic diagnostics, as previously shown [11]. For most genes, large deletions/duplications probably make up about 5–10% of the total mutational spectrum of the respective gene. For some genes the proportion is even much higher (e. g. for *HNF1B* almost 50% of patients have large deletions). So far, conventional CNV analysis has been usually done by multiplex-ligation probe amplification (MLPA) which results in additional costs and efforts, however. For many genes commercial MLPA kit are even not available and CNVs in these genes are thus usually missed by conventional approaches.

About 2–5% of ADPKD patients present with an early and severe phenotype [12]. Notably, affected families with early-manifesting offspring have a high recurrence risk for the birth of a further child with similar clinical manifestation, which clearly hints to a common familial modifying background for early and severe disease expression [13]. These early affected ADPKD patients are sometimes clinically indistinguishable from the recessive form ARPKD with mutations in *PKHD1*. Although much rarer, several further phenocopies are known and mutations in *HNF1B* (as a master regulator with a major effect on many cystic kidney disease genes) or genes that typically cause other ciliopathies with extrarenal features such as nephronophthisis (NPH) and Bardet-Biedl syndrome (BBS) can mimic PKD especially in the prenatal setting and early childhood [12]. Overall, increasing heterogeneity illustrates the need for a more comprehensive genetic testing approach targeting all genes that may have to be discussed for differential diagnosis.

A clear molecular diagnosis is indispensable for accurate genetic counselling, prenatal diagnosis, and genotype-phenotype correlations. Information on the underlying genotype is clearly also of advantage for proper clinical management and when selecting well-defined patients for clinical trials and future treatment options. However, in times of increasing cost pressure in the health care system this requires time- and cost-efficient genetic testing approaches. Thus, we demonstrate the utility of a sequence capture based NGS strategy that will largely alleviate genetic testing of PKD. In a single step, our approach allows specific mutation analysis of all genes known for cystic and polycystic kidney disease including the complete *PKD1* locus as well as the detection of CNVs in these genes. We illustrate that the employed mapping algorithm specifically aligns reads to *PKD1* enabling efficient variant calling. However, we also depict rare conditions and sites where discrimination of reads between master gene and pseudogenes is challenging with an increased risk of generating false-negative results. Finally, we propose a complete testing strategy to cope with these issues by adoption of the analytical pipeline and by consideration of conventional techniques to minimize the risk of false negative results.

## Results

### Sufficient coverage of *PKD1* and other genes known for cystic and polycystic kidneys as a prerequisite for diagnostic testing

**Fig 1:**
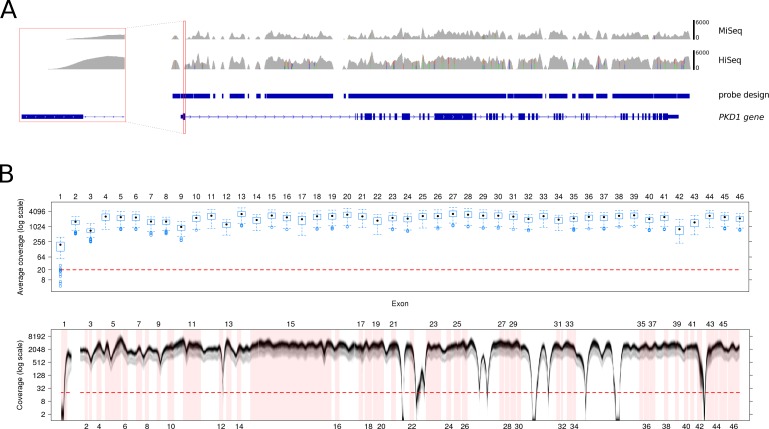
Probe design for the *PKD1* locus and coverage of the *PKD1* genomic and coding region. A. Coverage of the *PKD1* target region corresponds to probe design being nearly complete for the coding region with gaps in intronic segments. The red inbox (zoom) illustrates coverage of exon 1 with MiSeq and HiSeq sequencing showing the only obvious performance difference in this segment. B. The boxplot illustrates variation of the average coverage for each *PKD1* exon over all 55 samples of the validation cohort (log scale). The box boundaries mark the upper and lower quartiles, and whiskers extend to the most extreme data point in the 1.5-fold interquartile range. Outliers outside that range are displayed as a dot. In all exons except exon 1, a minimum coverage of >20x (red dashed line) is achieved even for the MiSeq samples as sufficient prerequisite for diagnostic testing. The levelplot below shows the coverage distribution of all samples by base position (intron 1 is omitted). The color intensity of each spot reflects the number of samples at that position, with a darker shading indicating more samples at that position. Exons are marked by red boxes. Except for exon 1, all samples at all exonic base positions have a coverage above 20x (red dashed line). MiSeq and HiSeq samples are mixed in that figure, therefore a shadowed grey area below the black main graph can be observed which makes up the MiSeq samples with a lower coverage in general.

In total, we sequenced 55 samples, 10 samples on the MiSeq and 45 samples on two lanes of a HiSeq 1500 system with 2 × 150 bp paired-end runs. For the whole target region including genes for cystic and polycystic kidney disease and potential phenocopies (in total, n = 40) (S1 Table) enriched and sequenced in parallel to *PKD1* a mean target coverage of 650x was achieved on the MiSeq and of 1443x on the HiSeq system on average across all samples with about 96.6% and 97.4% of the target regions covered with at least 20x, respectively (S2 Table). The average coverage of the completely targeted genomic region of the *PKD1* gene was 914x (91.8% >20x) on the MiSeq and 1993x (92.9% >20x) on the HiSeq (Fig. 1A) with gaps in the intronic sections. The 46 coding exons of *PKD1* were covered with an average coverage of 1198x and 2584x, respectively, nearly covering the complete coding sequence with 20x (98.6 and 99.1%). Moreover, nearly all coding positions in all exons were covered far more than 100x in all samples (Fig. 1B). As expected due to major hassles also known from conventional Sanger sequencing, the only region that could not be completely sequenced was the first half of exon 1 due to its high GC content and despite thorough rebalancing with 50.6% and 23.5% on average being 20x covered on the HiSeq and MiSeq, respectively. Nearly all target genes beside *PKD1* were on average covered >20x for 100% of the coding region with only minor exceptions due technical limitations (S3 Table).

### SNV and small indel detection by our capture-based NGS approach

**Fig 2:**
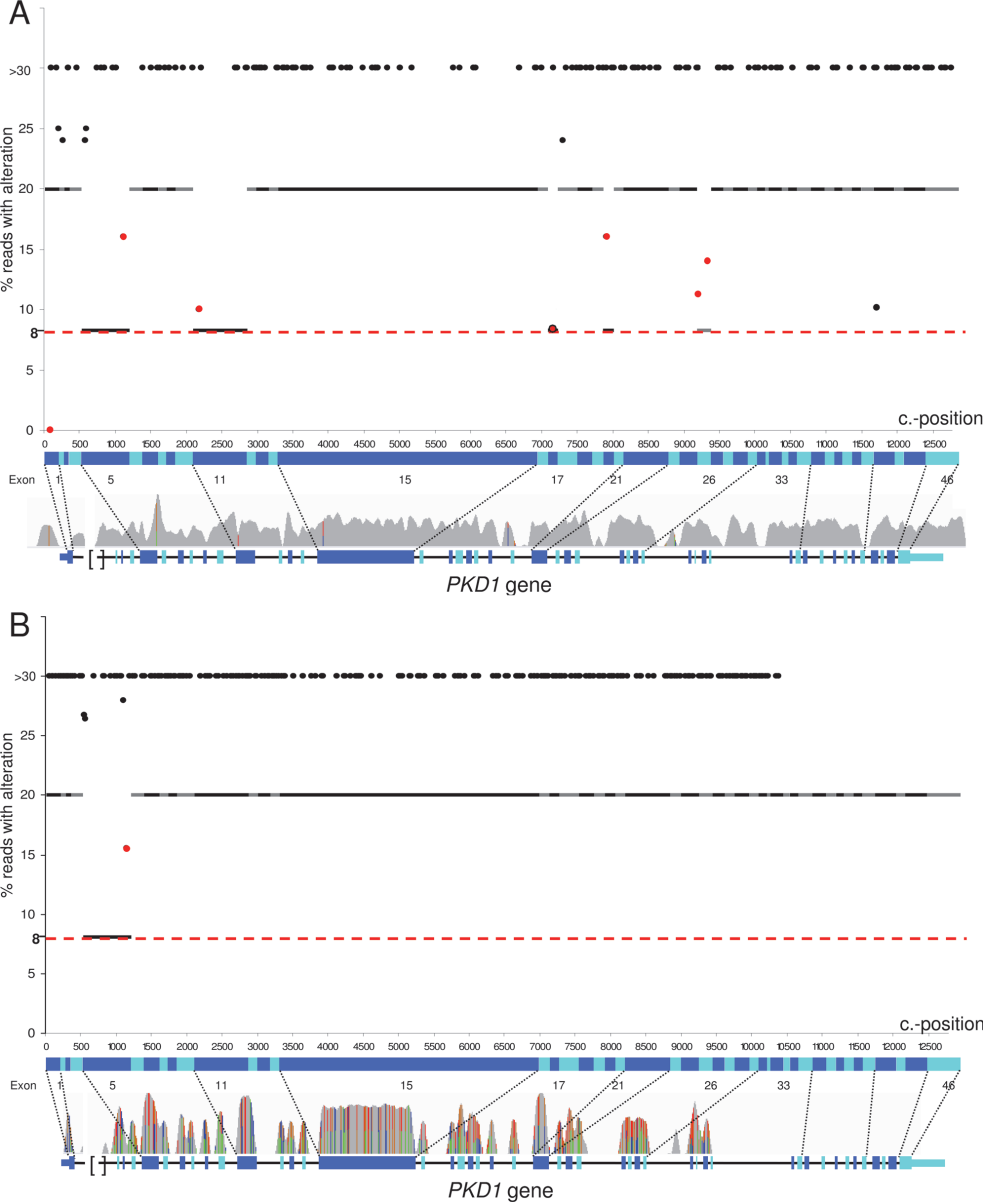
Detection level and distribution of *PKD1* variants in our cohort and by variant simulation. **A** Percentage of alternative reads detected by our NGS approach for all *PKD1* variants (black dots) from our cohort is displayed. All except seven variants could be detected above the standard detection threshold (black/grey lines displaying exon boundaries). The variants highlighted with red dots lie in the duplicated region and required second-step analysis with a lower detection threshold (8% alternative reads, red dashed line) to be detected in these critical exons. One variant in exon 1 could not be detected due to insufficient coverage on the MiSeq system. The variant in exon 42 lies at position +28 and is hardly covered due to insufficient coverage of this intronic region (no further adjustment of the analysis). Underneath, the *PKD1* genomic locus with a coverage plot is shown. **B** Percentage of alternative reads detected by our bioinformatic workflow for *PKD1* variants (black dots) from the read simulation by Wgsim. An evenly distributed variant density which is even higher than in the real dataset could be achieved across all coding exons in the duplicated region. Only one variant (red dot) in exon 5 could be detected below the default detection threshold (black/grey lines displaying exon boundaries). The red dashed line marks the lower detection threshold applied in negative cases. Underneath, the *PKD1* genomic locus with its coverage by simulated reads is shown. The coloured lines reflect the positions of detected variants.

NGS data of 53 *PKD1* SNV (single nucleotide variant)/ indel (insertions and deletions) positive patient samples were compared to results of previous analyses of these patients by conventional LR-PCR followed by Sanger sequencing. All but one of the 160 different variants (681 of 683 total variants, Table 1) virtually distributed evenly over all 46 *PKD1* exons (Fig. 1A) and including different mutation types (various SNVs, short insertions up to a 15 bp-duplication as well as short deletions with a maximal 12 bp-deletion) were identified (S4 Table) mainly with standard settings. All definitely pathogenic mutations, in total 31 nonsense mutations and indels, were clearly detected. One variant not detected by MiSeq sequencing located in exon 1 that was found to be insufficiently covered (S5 Table). However, this variant might have been detected when sequencing the sample on the HiSeq instrument (Fig. 1). Among putatively pathogenic variants, which were either predicted pathogenic (S6 Table) or had been reported with indefinite nature or were private to the respective patient, only one could not be detected (S5 Table). This specific indeterminate variant, c.2180T>C (p.Leu727Pro), in exon 11 could in one case not be detected at all due to missing the stringent and invariable filter criteria in variant calling (patient 21) and in a second case only after reducing the detection threshold in a second step analysis. In total, SNVs at only six distinct sites (Fig. 2A) in five exons of the duplicated region were either detected below the threshold of 20% reads at the altered position or were called with different zygosity (S5 Table). Inspection of the alignment showed two different cases: First, in most of these cases variants at these sites exist in at least one pseudogene copy with the complication that reads carrying the exchange map to 100% to a homologous region. Only few read pairs are present in the alignment which map to the master gene as discrimination is enabled by the second read of this pair (S1 Fig.). However, a proportion of all pairs show low mapping quality due to little sequence divergence between master gene and pseudogenes. In the second case two of these critical sites show sequence identity between *PKD1* and the pseudogenes at the site of interest. Filtering of reads against their mapping quality resulted in better results only at these two sites maybe due to filtering for pairs carrying the variant, for which mapping discrimination resulted in higher mapping quality (S2 Fig.). However, in general, the challenge of variant detection at these few sites could be coped with a second-step analysis by simple adjustment of the detection threshold to 8% alternative reads in all but the two above discussed false negative sites. It should be emphasized that relaxed filter criteria did not result in an increased detection of false-positive calls showing high specificity of the applied mapping algorithm even at a lower stringency. Interestingly, the 17 allegedly false positive calls not been detected by conventional testing restricted to only two sites in exons 10 and 11. Because of sequence identity of the genuine gene with the duplicated regions at these sites any spurious alignment of pseudogene regions causing their calls may be ruled out. As these two false-positive variants were detected in NGS only slightly above the threshold of 20% reads at the altered position and as they were not detectable by Sanger sequencing even by using alternative primer pairs, they most probably represent recurring sequencing artefacts specific to the whole workflow, which are to be filtered out by their internally measured allele frequency. Taken together, the approach showed a very high sensitivity (99.7%) and specificity (99.8%) (S4 Table) comparable to conventional testing methods.

**Table 1:** Validation statistics in *PKD1* mutation-positive patient cohort.

Variant statistics	# Variants	# Detected	Detection rate
Sanger verified variants (SNVs, insertions/deletions)	683	681	99.6%
Different variants	160	159	99.4%
Definitely and putatively pathogenic mutations	63	62	98.4%
Different mutations	58	58	100%
Novel mutations (different ones)	36	36	100%

### Variant simulation in the duplicated *PKD1* region with a high variant density underscores sensitivity of our approach

In our cohort almost all exons of the *PKD1* gene were covered by real variants. To have a closer look at gaps that escaped evaluation and to rule out further coding segments in the duplicated region, in which detection of variants might be challenging due to the high homology between *PKD1* and its pseudogenes at and around a site of interest, SNVs and indels in the *PKD1* gene were simulated using Wgsim by having at least one alteration within each 100 bp of coding sequence in the duplicated region (exons 1–33). Simulated reads were generated with a coverage of 1000x and combined with unmodified reference genomic sequence of all six pseudogenes (*PKD1P1–6*) with equal coverage. The combined reads were finally mapped and analysed using our in-depth bioinformatic pipeline (S3 Fig.). Thereby, a total of 241 different mutations were simulated with an even distribution over all 33 exons of the duplicated region (Fig. 2B, S7 Table), which is an ever higher variant density than for the 126 different changes from the validation cohort in this region. All except one mutation in exon 5 could be detected at standard filter criteria (20% alternative reads) resulting in 99.6% sensitivity without adjustment of the detection threshold. Interestingly, the simulated nucleotide exchange at position c.1155 detected only in 15.5% of all reads is located close to the most common critical real variant at position c.1119 discussed above demonstrating that variant detection at this segment is challenging due to competitive mapping with pseudogene regions emphasizing how realistic our simulation reflected the real world scenario.

### Potential CNV detection in all target genes

**Fig 3:**
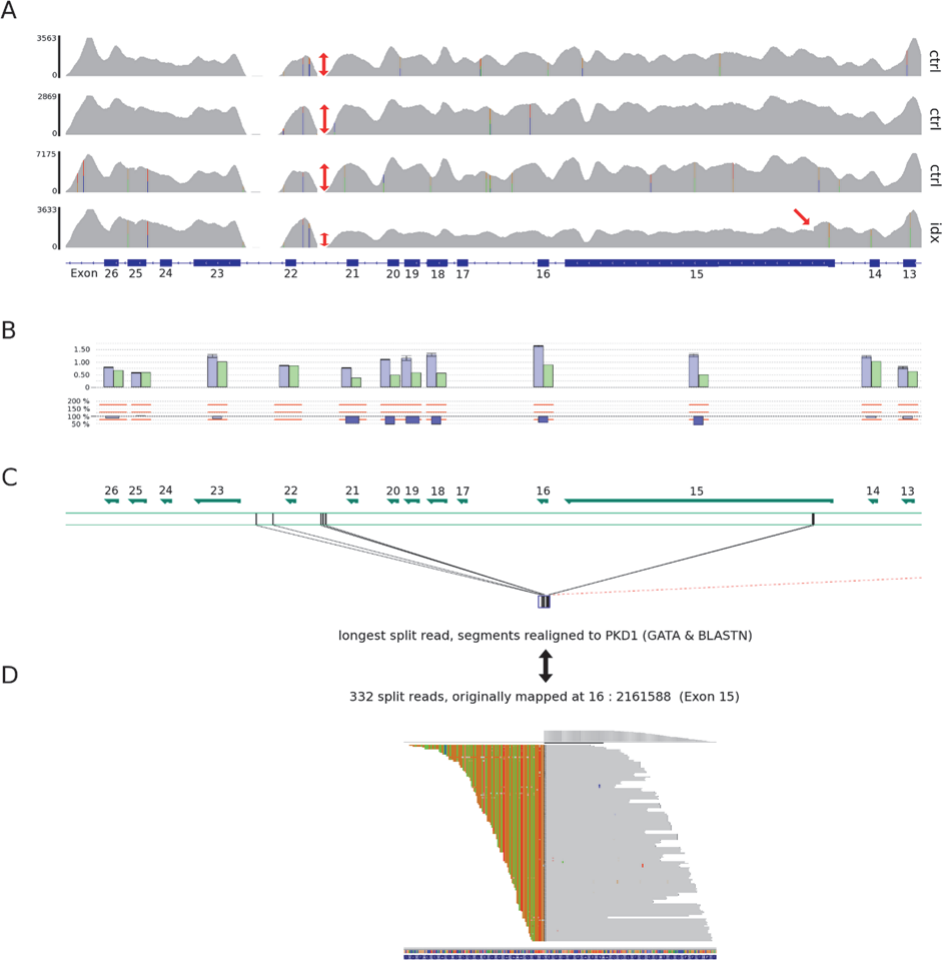
CNV detection of multiple exon deletion in *PKD1* for patient 46 with breakpoint analysis. **A** Coverage plots (IGV) of three control (ctrl) patients versus the index (idx) sample illustrate the statictical readout with a drop in coverage at *PKD1* exons 15–21 for the index (red double arrows) compared to controls indicating a deletion event at this position. Correspondingly, a within-sample decrease in coverage to surrounding exons can be observed in the index patient. The 5’ decrease in coverage is located at a clear-cut position at the beginning of exon 15 (red arrow). The *PKD1* gene is displayed from the right to the left. **B** Result from MLPA analysis displayed by MLPA module in JSI SeqPilot software showing the deletion of exons 15–21 in *PKD1*. The relative peak area (RPA) of the patient result file (green) and of the control result file (blue) with standard deviation (error bar) is shown. Ratio RPA was calculated as RPA of the patient versus controls. Deletions are indicated if the ratio RPA falls below 75%. **C** and **D** Evaluation of the exact breakpoint of the deletion using split reads. Initially, the ClipCrop tool identified an occurrence of excess split reads in exon 15, here visualized using IGV (D). The soft-clipped (unaligned) part of split reads was further inspected in part (C) by application of general motif search tools (GATA and BLASTN algorithm). The highest-scoring alignment within *PKD1* was found in the repeat-rich intronic region between exons 21 and 22, therefore representing the most likely 3’ deletion breakpoint. This repeat-rich region escapes sequence capture and probably represents a hotspot for chromosomal rearrangements.

We utilized VarScan to evaluate the potential of our approach for CNV analysis. Two patients harbouring previously known deletions in *PKD1* (patient 30 carrying a single-exon deletion of exon 22 and patient 46 bearing a genomic deletion comprising exons 15–21) were used as positive controls. Six samples that were previously proven by MLPA to have normal copy numbers for *PKD1* served as negative controls. As expected from our results for SNV calling, regions with evaluated competitive mapping in the duplicated region (five exons) were challenging to analyse and generated false-positive CNV calls in these exons. Therefore these and recurrent CNV artefacts as for exons 1 and 42 attributable to insufficient or varying coverage of these regions well-known for their complexity were excluded from the analysis. Applying those stringent filter criteria, a deletion of exon 15 in patient 46 and a deletion of exon 22 in patient 30 could be clearly detected, showing the CNV convincingly against all control samples (Fig. 3 and S4 Fig., S8 Table). When lowering the detection threshold to against 50% control samples and with the anchor point of exon 15 being most probably deleted, the extent of the above multi-exon deletion encompassing exons 15–21 could be precisely predicted from NGS CNV data. Interestingly, combining this depth of coverage detection method with a split read approach using ClipCrop, 332 reads were obtained which supported a breakpoint in exon 15 (Fig. 3). A Smith-Waterman alignment of the unmapped part of the spilt reads against the entire DNA sequence of *PKD1* found the best match in intron 21 known to be repetitive.

**Fig 4:**
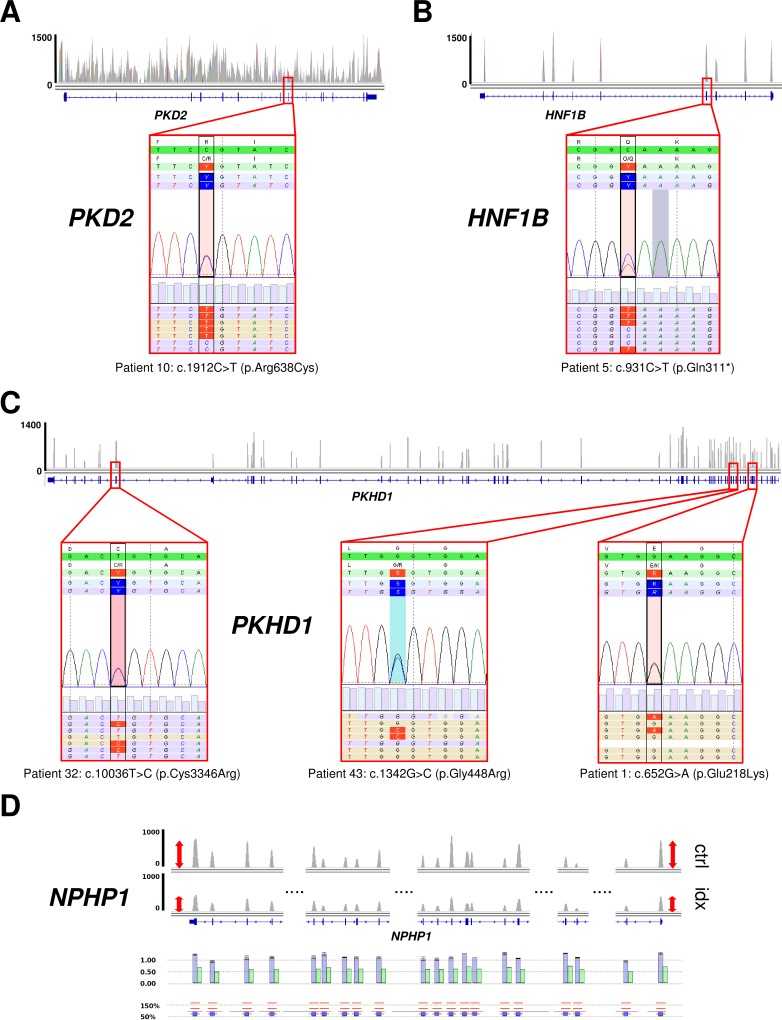
Mutations detected in other genes for cystic and polycystic kidney disease. Examples for sufficient coverage (plots, upper panel) and detected mutations (pseudo-electropherograms and alignments in SeqNext software) in *PKD2* (**A**), *HNF1B* (**B**) and *PKHD1* (**C**). The small red box indicates the position of the mutation in the gene track (IGV). Robust CNV detection is possible in all genes of the panel as demonstrated for a heterozygous deletion of *NPHP1* (**D**, for details see legend Fig. 2).

The feasibility of the sequence capture approach for CNV analysis in the other targeted genes could be demonstrated by detection of a known heterozygous deletion of the complete *NPHP1* gene in patient 3 (Fig. 4D). As seen in previous gene-panel sequencing setups detection of deletions in the *HNF1B* gene, which might account for up to 50% of *HNF1B*-mutation positive cases, is also accessible by a sequence capture based NGS approach (S5 Fig.). Overall, CNV detection in the other targeted genes might be even more solid than for *PKD1*. Further optimization is reasonable before conventional MLPA analysis for *PKD1* can be ultimately regarded superfluous in mutation-negative ADPKD patients. However, under stringent filter criteria for *PKD1* analysis CNVs present in our patient cohort were correctly detected.

### Evaluation of variants in other genes for cystic and polycystic kidney disease

As seen for CNV analysis and as already proven by previous gene-panel based NGS studies parallel analysis of all other targeted genes does not constitute a major technical issue [14],[15],[16]. As proof-of-principle NGS data of our patient cohort were comprehensively analysed for pathogenic aberrations in other genes for cystic and polycystic kidney disease which were all sufficiently covered for diagnostic testing (Table 2). For example, in patient 10 the detection of a mutation in *PKD2* (Fig. 4A) underscored the feasibility of the approach for parallel analysis of the second known gene for ADPKD. In this patient with suspected ARPKD and negative family history with normal parental ultrasounds, *trans*-heterozygosity of a hypomorphic *PKD1* allele and a *PKD*2 mutation might be speculated on, but this hypothesis needs further validation before firm conclusions can be drawn. In patient 5 with unremarkable family history and a phenotype in between ARPKD and ADPKD, a nonsense mutation in *HNF1B* was identified *in trans* to a likely hypomorphic *PKD1* variant (Fig. 4B). In a number of other patients with early and severe PKD and negative family history suspicious for ARPKD, conventional approaches would have started with Sanger sequencing of *PKHD1* which is already laborious and costly due to the huge size of this gene. In some of these patients the identification of a mutation in *PKHD1* would have potentially led to the wrong assumption that *PKHD1* might be the major disease locus and that genetic testing of other genes is thus dispensable (Fig. 4C). However, only knowledge of the mutational status of other genes such as *PKD1* revealed that the main disease locus is a different one in these individuals (S6 Fig.). Conclusively, our data demonstrate the power of the setup for parallel analysis of all genes known to date for cystic and polycystic kidney disease and related disorders in a single step.

**Table 2:** Mutations and variants identified in other genes for cystic and polycystic kidney disease.

Patient	*PKD1* mutation	Classification	Additional variant(s)	Classification	Comment
10	c.8293C>T (p.Arg2765Cys) (het)	LH*	*PKD2*: c.1912C>T (p.Arg638Cys) (het); *PKHD1*: c.334G>A (p.Gly112Arg) (het); *ANKS6*: c.2464T>C (p.Phe855Ser) (het)	HLP*; P**; LP	*Trans*-heterozygosity of *PKD1* and *PKD2* mutations may have led to PKD in this patient with suspected ARPKD and negative family history; *ANKS6* variant might represent accidental carriership of a putatively pathogenic alteration; a modifying effect on the patient’s phenotype cannot be ruled out
5	c.9484C>T (p.Arg3162Cys) (het)	LH*	*HNF1B*: c.931C>T (p.Gln311*) (het)	DP	Patient with unremarkable family history and PKD phenotype carries causative *HNF1B* nonsense mutation *in trans* to a likely hypomorphic *PKD1* variant
3	c.7345_7356del (p.Thr2449_Gly2452del) (het)	PP	*NPHP1*: deletion exons 1–20 (het)	DP	*PKD1* revealed as major disease locus in a patient with suspected ARPKD and negative family history; *NPHP1* deletion might represent accidental carriership of a well known mutation; case demonstrates detectability of CNVs in genes beside *PKD1*
2	c.6040C>T (p.Gln2014*) (het)	DP*	*PKHD1*: c.2168G>T (p.Arg723Leu) (het)	LH	*PKD1* revealed as major disease locus in a patient with a putatively hypomorphic *PKHD1* variant
1	c.5014_5015del (p.Arg1672Glyfs*98) (het); c.12019C>T (p.Arg4007Cys) (het)	DP*; HLP*	*PKHD1*: c.652G>A (p.Glu218Lys) (het)	P**	Detection of pathogenic *PKHD1* mutation in a patient with suspected ARPKD and negative family history might have been indicative for this gene being the main disease locus without knowledge of the *PKD1* genotype
43	c.12671_12674del (p.Thr4224Serfs*133) (het)	DP	*PKHD1*: c.1342G>C (p.Gly448Arg) (het); *MRE11A*: c.1516G>T (p.Glu506*) (het)	P**; DP	Detection of pathogenic *PKHD1* mutation in a patient with suspected ARPKD (family history unclear at time of initial testing) may have been misleading; *MRE11A* mutation might represent accidental carriership of a putatively pathogenic alteration; a modifying effect on the patient’s phenotype cannot be ruled out
17	c.4697_4698insA (p.Ser1567Glufs*11) (het)	DP	*PKHD1*: c.3407A>G (p.Tyr1136Cys) (het)	PP**	Detection of probably pathogenic *PKHD1* mutation in a patient with suspected ARPKD and negative family history may have been misleading
32	c.3542A>G (p.Tyr1181Cys) (het)	LP	*PKHD1*: c.10036T>C (p.Cys3346Arg) (het)	P**	Detection of pathogenic *PKHD1* mutation in a patient with suspected ARPKD and negative family history may have been misleading

NGS data that demonstrate the power of the setup for parallel analysis of all genes known to date for cystic and polycystic kidney disease and related disorders in a single step.

* Classification from PKD mutation database (ADPKD Mutation Database (http://pkdb.mayo.edu/)

** classification taken from ARPKD/*PKHD1* database (http://www.humgen.rwth-aachen.de); het—heterozygous; LH—likely hypomorphic; LP—likely pathogenic; HLP—highly likely pathogenic; DP—definitely pathogenic; PP—probably pathogenic; P—pathogenic.

## Discussion

Genetics becomes increasingly important for proper clinical management and prognostic issues, but still the cost of genetic testing often is a major barrier. Thus, there is a need for novel time- and cost-efficient strategies in molecular genetic testing. NGS has already entered the field of clinical diagnostics and will further accelerate molecular genetic testing of a broad spectrum of inherited human diseases [11],[17],[18]. However efficient capture-by-hybridization approaches have been precluded from the analysis of duplicated regions, e.g. most parts of the *PKD1* gene, due to suspected risk of concurrent capture of pseudogenic regions and misalignment of reads from the duplicated region to the master gene [9]. Here we demonstrate the utility of a sequence capture based NGS approach validated in a cohort of 55 *PKD1* mutation positive patient samples. The results of this validation study showed high sensitivity, specificity and detection rates (> 98%) comparable to conventional Sanger sequencing approaches and LR-PCR-based NGS setups [9],[19]. Due to an optimized probe design nearly complete coverage of the whole *PKD1* coding region was achieved on both used sequencing platforms. Our approach also emphasizes that the analysis of a limited number of genes in a panel-based setup is currently far superior over exome capture protocols which only achieve partial and incomplete enrichment e. g. of the *PKD1* duplicated region (S7 Fig.).

The essential clue to be successful with our approach was to target the whole genomic region of the *PKD1* locus allowing parallel capture of the duplicated regions in the six pseudogenes (*PKD1P1-P6*). Specificity of this approach was mainly achieved by accurate and specific mapping of 2 × 150 bp paired-end sequencing reads to the master gene, but also equivalent mapping of reads to the six pseudogenes (S8 Fig.). Using a mapping algorithm with mainly standard settings, standard filter and variant calling criteria similarly applied for routine sequence capture approaches are sufficient for unambiguous detection of sequence variants in most of the *PKD1* regions. Thereby, erroneous calling of false positive variants due to misalignment of pseudogene reads to the master gene does not represent a threat of this setup. However, we depict limitations of the bioinformatic algorithms at six different sites, where competitive mapping of read pairs between master gene and pseudogene regions impairs efficient read discrimination. Especially reads with variants present in at least one pseudogene sequence—maybe as a consequence of a gene conversion event or an accidental mutation—and therefore mapping to 100% to one homologous region constitute a major challenge of the algorithm, as most of these variant reads are not aligned to the genuine gene (S1 Fig.). However, although mapping of “mutated” *PKD1* reads to one pseudogene is preferred due to the identity to this region there is still a low level of reads at these sites which map to the master gene, most probably due to a discriminative effect of the second read of the pair, so that the challenge of detecting such variants could be overcome by a second-step analysis applying a lower detection threshold (8% alternative reads) without generating additional false positive calls. Further decreasing this threshold did not result in detection of all evaluated variants: One of the two false negatives was simply not covered by MiSeq sequencing, the second did not pass the stringent and invariable filter criteria in variant calling which are not to be adopted to avoid more false positive calls. As a relevant proportion of reads at the critical sites have low mapping quality we did not apply filtering reads against mapping quality out of the total reads, although in few cases this filter step proofed to increase the percentage of altered reads (S5 Table). Sequencing of longer fragments to enable read discrimination by a more distant second read of one read pair, longer reads (2x300bp), which are either possible on an Illumina or another platform (e.g. Pacific Biosystems), as well as optimized mapping algorithm settings might provide handles to further improve discriminative mapping at such sites to minimize the risk of false negative results. The bioinformatic analysis pipeline will clearly benefit from further evaluation of data generated with such improved protocols as well as from re-evaluation of larger datasets to identify additional critical sites to further optimize settings for variant filtration and calling.

Variant simulation in the duplicated *PKD1* region underscored the high sensitivity of our approach and added 214 further sites tested to the 126 different “real” variants evaluated in the validation cohort, finally covering all exons in the duplicated region. All except one mutation could be detected in the simulation with standard settings. The fact that the one mutation identified slightly below the standard threshold located close to the most common critical real variant demonstrated suitability of the simulation. Most importantly, due to a high variant density comprehensive simulation efforts circumvented the need of a discovery cohort in our study. For technical validation of our setup it was rather far more appropriate to clarify the performance of the bioinformatic workflow in gap regions not covered by a “real variant”.

The presented sequence capture based NGS approach has some obvious advantages over conventional techniques. By this, it is of utmost importance to notice that Sanger sequencing is by far not as perfect as often thought and shows several significant shortcomings in addition to its known limitations in throughput and costs. For instance, conventional LR-PCR approaches for *PKD1* show residual contaminating signals from pseudogenes if combined with Sanger sequencing (S9 Fig.), but also with amplicon-based NGS [9]. Other limitations of Sanger sequencing which may lead to false negative results (e.g. allele dropout due to uneven allele amplification) have been described elsewhere in detail [9],[20],[21],[22] and are overcome by our approach, which is independent of site-specific primers.

A further plus of our strategy is its ability to detect CNVs present in about 5% of ADPKD patients [21],[23] which is not possible by Sanger sequencing and was also not reported for the amplicon-based NGS setup by Rossetti et al. [9]. Although there are still technical limitations, proof-of-principle is shown in two patients. Application of optimized CNV calling algorithms, e.g. the use of split read based CNV callers (Fig. 3), might be able to cope with the challenge of misbalanced amounts of captured sequence due to the concurrent mapping of pseudogene reads at distinct sites encountered by depth-of-coverage based tools. CNV detection will also benefit from optimized protocols sequencing larger insert fragments and employing longer read lengths probably making detection of large deletions in *PKD1* as robust as seen for other targeted genes in our setup. Conventional CNV analysis is currently usually done by MLPA causing additional costs and efforts. Moreover, for many genes no MLPA kit is available and CNVs in these genes are thus generally missed. Furthermore, results from MLPA analysis are sometimes not unambiguous and difficult to interpret. Our approach is attractive and most cost-effective as CNV calling can be performed in parallel to SNV and small indel detection in a one-step analysis.

ADPKD is typically a late-onset disease and mainly caused by mutations in *PKD1* or *PKD2*, but about 2–5% of patients show an early and severe phenotype that is clinically often indistinguishable from autosomal recessive polycystic kidney disease (ARPKD). Some of these severely affected patients carry more than one mutation in PKD genes probably aggravating the phenotype [24]. Moreover, clinical manifestations of PKD can be mimicked by mutations in a number of genes. We and others have shown that mutations in genes that typically cause other ciliopathies such as nephronophthisis or Bardet-Biedl syndrome may sometimes phenocopy PKD which underscores the need for more comprehensive genetic testing. This especially holds true for patients lacking a positive family history as well as for children and in prenatal settings when other features (e.g., polydactyly, obesity, retinitis pigmentosa) are not present [12],[25],[26]. We have previously demonstrated the feasibility and efficiency of a capture-based NGS multi-gene panel approach (besides *PKD1)* in a number of other studies for PKD and other ciliopathies, which is not surprising as there are only very few complex or duplicated sites in those other genes discussed [14],[16],[27]. Our data demonstrate the feasibility of such an approach for the parallel analysis of targeted genes with the addition of a complete *PKD1* setup. This is exemplified by the detection of further mutations in some of our patients. This only allowed to finally understand the genetic basis of the disease in these individuals which was different from the initial clinical assumption (Table 2, Fig. 4). Overall, targeted screening of a number of genes increases the detection rate, allows for better interpretation of identified variants and helps to avoid genetic misdiagnoses. These latter aspects are also illustrated by patients with suspected ARPKD and only one detectable *PKHD1* mutation (a constellation that is present in a considerable number of patients) in which the second mutation is then often argued to have been missed due to technical issues or deep intronic location. However, in fact, the major disease locus might be different from *PKHD1*. Often it is *PKD1* as shown for some of our patients (Table 2, S6 Fig.).

A recent study proposed a targeted sequence capture based NGS setup for ADPKD diagnostics [28]. However, essential shortcomings of this work were obvious: First, the authors only provided low analytical depth employing a small validation cohort of *PKD1* mutation-positive patients. Systematic evaluation of all Sanger-identified variants including polymorphisms was not carried out therefore providing a very low variant density in the duplicated region even not covering all *PKD1* exons. Second, they recognized that mutation calls are on average lower than reference calls employing a similar mapping algorithm at standard settings we used. However, they did not reflect any critical sites with limitations of the setup probably requiring any further adjustment of their variant detection pipeline to minimize the risk of false negative calls due to competitive mapping between master gene and its pseudogenes. Finally, the only limitation the authors of this study pointed out is insufficient coverage of exons 1 and 42 restricting additional testing to completion of these regions by conventional Sanger sequencing. Here we demonstrate that the quality of NGS data in genes like *PKD1* not only relies on coverage of the target regions, but also on verification of bioinformatic mapping algorithms and the identification of possible limitations and pitfalls of the applied setup based on a high analytical depth in the validation study.

**Fig 5:**
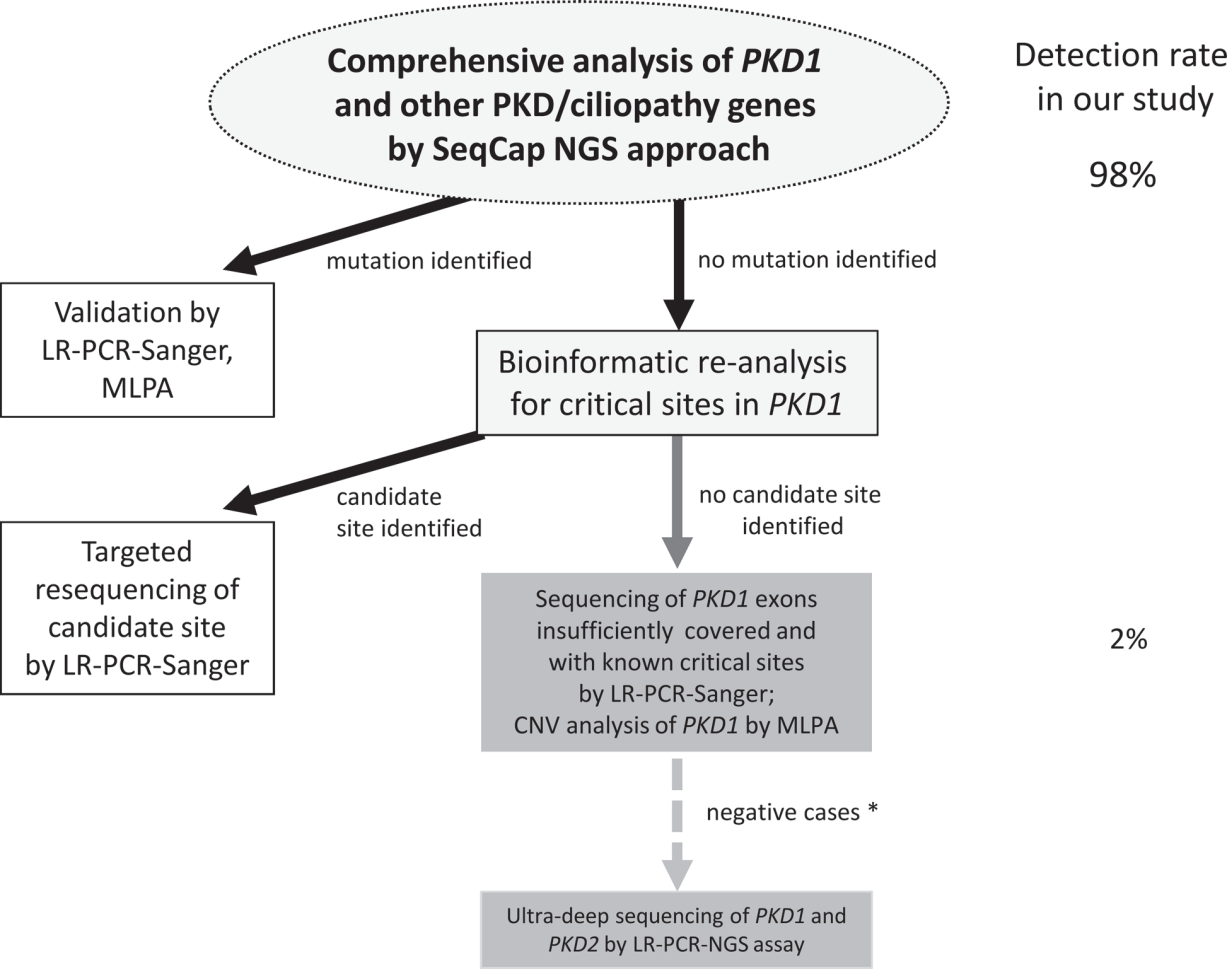
Comprehensive and complete testing strategy for ADPKD diagnostics. Sequence capture based NGS for variant detection in *PKD1* and all other targeted ciliopathy genes at standard filter criteria is the central and first step in the analysis which includes validation of putative mutations by conventional techniques (LR-PCR-Sanger, MLPA). By this, in more than 98% of our cohort the disease-causing mutation could already be identified. Further optional steps to minimize the risk of false negative results include deeper bioinformatic analysis of acquired NGS data and restricted use of conventional methods. Bioinformatic re-analysis by lowering the detection threshold (> 8% alternative reads) in mutation-negative samples might provide candidate sites in *PKD1* where concurrent mapping of reads between the genuine gene and homologous regions impairs proper variant detection. Candidate sites are validated by conventional Sanger sequencing. If still no convincing mutation is present, restricted sequencing by LR-PCR-Sanger of insufficiently covered regions (exon 1) as well as those exons with empirically evaluated critical regions bearing mapping difficulties (in our study exons 5, 11, 17, 21, 26) is the next option and would have generated the correct result for patient 21 of our study. In parallel, MLPA analysis of *PKD1* can be used to rule out false-negative CNV results. An optional final step might be ultra-deep sequencing with very high coverage of the *PKD1* and *PKD2* coding sequence (S9 Table) especially in those mutation-negative patients with a clear ADPKD phenotype and negative family history (*) to allow for detection of low-level mosaicism.

Finally, we propose a complete testing strategy combining NGS based and conventional methods (Fig. 5). The initial and central step of the workflow comprises variant detection in *PKD1* and all other targeted genes by our NGS approach at standard filter criteria and should already result in detection of the disease-causing mutation in the vast majority of patients. Further optional steps to minimize the risk of false negative results include deeper bioinformatic analysis of acquired NGS data and restricted use of conventional methods. Optimization of the laboratory and bioinformatic workflow will be of importance to further improve the NGS setup, increase the awareness of potential false negative results and to further substitute conventional testing. However, already in its current form the described method represents an attractive option for diagnostic labs to further streamline their workflow. Yet, implementation requires thorough on-site validation and experience particular in handling *PKD1* NGS data for optimal performance of the test. Finally, ultra-deep sequencing of *PKD1* and *PKD2* based on LR-PCR amplification (S9 Table) might be an option especially in mutation-negative patients with a clear ADPKD phenotype and negative family history to allow for detection of low-level mosaicism.

## Materials and Methods

### Ethics Statement

All samples were obtained with written informed consent. Clinical investigations were conducted according to the Declaration of Helsinki. The study was approved by the institutional review board of the Ethics Committee of the University Freiburg Medical Center.

### Samples and preceded Sanger sequencing

DNA samples of 55 patients were included as positive controls in this study, 53 of them were previously sequenced by conventional LR-PCR based Sanger sequencing. Two samples harboured a large deletion in the *PKD1* gene previously detected by MLPA (multiplex ligation dependent probe amplification). Genomic DNA was isolated from EDTA blood following standard protocols. Preceded Sanger sequencing had been conducted on long-range PCR (LR-PCR) amplicons as described elsewhere [8,9]. For reassessment of the exons 10 and 11 of *PKD1* alternative primer pairs were used as described in [19] (S1 Materials And Methods). Identified sequence variants had been annotated according to the guidelines published by the Human Genome Variation Society.

### NimbleGen SeqCap EZ choice library design

The complete genomic region of the *PKD1* gene with 1 kb of flanking sequence (NC_000016.9; chr16:2137709–2186899) was targeted by a custom SeqCap EZ choice library (NimbleGen, Madison, Wisconsin, USA). In order to capture the exons in the duplicated region the preferred stringent settings of allowing three close matches in the genome for probe selection were relaxed to up to ten close matches. Due to the high GC content of exon 1 and the low coverage for exon 42 of *PKD1* obtained in previous sequence capture approaches we added additional twelve times replicated probes in these regions (rebalancing). In terms of differential diagnosis, the complete genomic sequence of the *PKD2* gene and the coding and non-coding exons of 38 other genes for allied human diseases with related clinical manifestations (S1 Table) with 20 bp of flanking genomic sequence were additionally targeted by our design with preferred settings resulting in 820 target regions with 300 kb of target sequence. Rebalancing was also applied to regions showing low depth of coverage in previous panel sequencing (e.g. exon 1 of *PKD2*). The design covered about 97% of all requested target regions. The coding exons of the *PKD1* gene could be completely targeted and about 84% of the whole genomic region was covered. For more details about the NimbleGen design see S1 Materials And Methods. The BED file of the final capture design is available on request.

### Sequence capture and next-generation sequencing

Libraries were prepared using NEBNext DNA library prep master mix set for Illumina (New England Biolabs, Ipswich, MA, USA) according to the manufacturer’s protocol. In brief, 1 μg of genomic DNA per sample was sheared to generate DNA fragments of 250–300 bp using the Covaris S2 AFA system (Covaris Inc. Woburn, MA, USA) and ligated to Illumina specific adaptors for multiplexing. Pre-capture amplified samples were pooled—10 samples for MiSeq and up to 23 samples for HiSeq 1500 sequencing—and hybridized to the customized in-solution capture library for 72 hours, subsequently eluted and post-capture amplified by ligation mediated (LM-) PCR. This amplified enriched DNA was used as input for direct cluster generation and sequencing on an Illumina MiSeq system (2 × 150 bp PE reads) or for cluster generation on a cBot system and sequencing in rapid mode (23 samples per lane, altogether up to 46 samples) on an Illumina HiSeq 1500 instrument (2 × 150 bp PE reads) (Illumina, San Diego, CA, USA). A more detailed protocol is available in online S1 Materials And Methods.

### Bioinformatic data analysis

NGS data were analysed with an in-house bioinformatic pipeline as previously described [11],[16],[29]. Demultiplexed reads from Illumina MiSeq and HiSeq sequencers were mapped against the hg19 human reference genome using BWA v0.7.8 [30] with the BWA-MEM alignment algorithm and the recommended standard settings and preprocessed with SAMtools v0.1.19 [31] to convert the SAM files into BAM files and sorting them on coordinate order. Duplicate reads were marked with MarkDuplicates from Picard v1.112 (http://picard.sourceforge.net), and the tools RealignerTargetCreator, IndelRealigner, BaseRecalibrator and PrintReads from GATK (Genome Analysis Toolkit) v3.1 software package [32] were applied for a local realignment and base quality score recalibration of the mapped reads. All tools were used with the recommended standard settings. This workflow is in accordance with the best practices from the Broad Institute. Variants were called with the tool UnifiedGenotyper from GATK. Mapping and coverage statistics for the *PKD1* regions were calculated using the tool DepthOfCoverage from GATK, as well as the tool CalculateHsMetrices from Picard. The identified variants were annotated using ANNOVAR and the RefSeq gene-based annotation method. After this, all variants were checks against the population frequency databases 1000 Genomes Project (1000g2012apr), Exome Sequencing Project (esp6500si_all). Variants have already been annotated with the dbSNP build 138 database during variant detection with UnifiedGenotyper in a previous workflow step. A more detailed description of the bioinformatics workflow and used scripts are supplied in online S1 Materials And Methods. Annotation (dbNSFP, HGMD), functional prediction and classification of identified variants was conducted as described previously [11],[29]. Additionally, *PKD1* variants were annotated with the entry from the ADPKD Mutation Database Version 3.0 (http://pkdb.mayo.edu/). Results from NGS were compared to results from preceded Sanger sequencing (gold standard) in exonic regions ± 30 bp of flanking intronic sequence using bioinformatic scripts and by visual comparison.

For detection of variants in the *PKD1* gene the following criteria were applied:

1. Variants were included in the analysis when ≥ 20% of total reads at the position showed the alteration. A variant was called homozygous when ≥ 85% of all reads had the variation.
2. Variants previously identified by Sanger sequencing or annotated in the ADPKD database which could only be detected below the threshold of 20% of total reads were used as indicators for exonic areas in the duplicated region (exon 1–33) where discriminative mapping of reads between master gene and pseudogene was insufficient due to high sequence homology in these reads or where the alternative allele might consequently be underrepresented (S5 Table). Such cases could be evaluated in five exons (5, 11, 17, 21, 26; Fig. 1). If filtering against variants with reads ≥ 20% did not result in detection of a pathogenic alteration, in a second step analysis criteria in the duplicated region of *PKD1* (exon 1–33) were relaxed to ≥ 8% to detect candidate pathogenic variants for subsequent confirmation by Sanger sequencing. The value of 8% was empirically determined as tradeoff between detection of further variants and generating more false positive results. Further relaxing of the threshold did also not result in detection of all inspected variants due to stringent variant calling criteria ahead of this filtering step.

Further filtering of the variants in routine testing against minor allele frequency (MAF≤1%) and *in silico* predicted pathogenicity is performed as previously described [11] with nonsense, frameshift and canonical splice site variants being considered pathogenic.

For visualization of the identified SNVs, BAM-files were loaded into the SeqPilot SeqNext module (v4.1.2, JSI medical systems, Kippenheim, Germany) and IGV (Integrative Genomics Viewer) from Broad Institute.

All identified variants are validated by conventional Sanger sequencing. Divergent zygosity of variants may be corrected to the Sanger result after bioinformatic inspection of the alignment at the site of interest in IGV.

### Copy number variation analysis

We performed copy number variation (CNV) analysis on highly covered samples sequenced on the Illumina Hiseq1500 system. Potential copy number alterations (CNA) were initially identified with the tools copynumber and copyCaller from VarScan v2.3.6 [33] on mapped reads with a maximum segment size of 300. All other parameter were used with standard settings. Thereby coverage of every target region of the sample of interest was internally normalized and compared versus normalized control data of other samples of the same run. CNVs were annotated using RefSeq gene file from UCSC (ftp://hgdownload.cse.ucsc.edu/ goldenPath/hg19/database/refGene.txt.gz). Potential CNVs were initially taken into account, if the CNV was detected by VarScan against at least 85% of the control patients and if the log_2_ threshold was ≥ 0.6 in case of an amplification or ≤ −0.6 in case of a deletion. CNVs below this threshold (detected against 50% to 85% of the control patients) were indicated, but filtered against stringent criteria to be considered for subsequent MLPA validation: CNVs in critical exons with restricted discriminative mapping (see above and Fig. 2) and recurrent CNV artefacts in exons 43 and 46 were neglected. If heterozygous reads were evident in exons with an indicated CNV, these CNVs were also neglected as were CNVs in exons 1 and 42 due to insufficient or varying coverage. Results from CNV analysis were compared to previous results from MLPA (multiplex ligation dependent probe amplification) analysis. For the *PKD1* gene the SALSA MLPA probemixes P351-C1 and P352-C2 were used (MRC-Holland, Amsterdam, The Netherlands). A total of six MLPA negative samples were used as negative controls for CNV detection.

As split read method we used the ClipCrop [34] program for identification of soft-clipped reads in *PKD1*. For patient 46, no CNV was explicitly called using the default thresholds of the program, but a manual review of the extracted split reads revealed an excess of such reads in a position in exon 15. These reads were visualized using IGV (Fig. 3). With 332 split reads supporting a breakpoint in that position, we further inspected the unmapped part of the split reads to find a potential matching deletion endpoint. A Smith-Waterman alignment of this sequence was performed versus the entire DNA sequence of *PKD1*, and, as suggested by coverage-based CNV analysis, the best match was found in the intronic region between exons 21 and 22. This represents a repeat-rich region that could not be enriched in the target capture process. Such a constellation might cause problems in the CNV calling module of ClipCrop as accompanying split reads at the potential deletion endpoint cannot be found. To further unravel the genomic structure of the patient at the CNV breakpoint, we applied the GATA tool [35] facilitating a more general motif search. We suppose that multiple re-shuffling of transposable repeat-elements in the genomically unstable regions of introns 21 and 22 could have created sequence alterations that cannot be mapped with conventional alignment programs. Application of the GATA tool decomposed the read sequence to short fragments in a sliding window approach (using windows of 20 bases) and subsequently mapped all fragments to the *PKD1* sequence via BLASTN. The best-aligning fragments delimit the CNV region boundaries as shown in Fig. 3. A more detailed description of the used bioinformatic scripts are supplied in online S1 Materials And Methods.

### Read simulation

In a first step, FASTA files for *PKD1* exons 1–33 where extracted from the hg19 reference genome using exon coordinates from the RefSeq gene file (ftp://hgdownload.cse.ucsc.edu/goldenPath/hg19/database/refGene.txt.gz) as well as the FASTA files for *PKD1* pseudogenes *PKD1P1–6* using hg19 coordinates from GeneCards (e.g. http://www.genecards.org/cgi-bin/carddisp.pl?gene=PKD1P1) [36]. For all locations we added 20 bp of flanking sequence. Next, we calculated the length for every location and the amount of simulated reads to achieve an average coverage which will be equal to those of a MiSeq run under the assumption that we need 1000X coverage for every sequencing fragment with a length of 200 bp (S3 Fig.). After this, we simulated the 2 × 150 bp paired-end reads in FASTQ format with Wgsim from the SAMtools package (Li, H. wgsim—read simulator for next generation sequencing). For the parameter of Wgsim we used a base error rate of 0.001, an outer distance between the two ends of 200 bp and a length of the first and second read of 150 bp for all locations. For the reads of *PKD1* exons 1–33 we simulated a mutation rate of 0.02 and for *PKD1P1–6* a rate of 0 which simulates wild-type reads with a base error rate only and no mutations. The number of simulated paired-end reads was adjusted to the appropriate location. All other parameters were used with standard settings. After all reads had been simulated, we merged all simulated FASTQ files into one file for R1 and R2 respectively and analyzed them with our in-house bioinformatic pipeline (see Bioinformatic data analysis). For more details see S1 Materials And Methods.

### Ultra-deep sequencing

For only targeting the *PKD1* coding region, long-range PCR (LR-PCR) amplicons were generated as described elsewhere using locus-specific primers [8,9]. Concentration of amplicons was measured by a fluorescence-based method. To minimize variation of coverage between amplified exons, LR-amplicons were equimolarly pooled or based on empirically determined ratios. These amplicon pools were taken as input for library preparation using the NEBNext DNA library prep master mix set for Illumina (New England Biolabs, Ipswich, MA, USA) as described above. Libraries of five to six patients were pooled and subjected to sequencing on an Illumina MiSeq system (2 × 150 bp PE reads). Data analysis was performed by the standard bioinformatics pipeline.

### Data repository

Sequence data for *PKD1* have been deposited at the European Genome-phenome Archive (EGA), which is hosted by the EBI, under accession number EGAS00001001003. Access can be gained upon request to the authors.

## Supporting Information

S1 Materials And Methods Supporting materials and methods. (PDF)

S1 Fig Challenging discriminative mapping between master gene and pseudogene regions at one critical site. (PDF)

S2 Fig Critical sites with positive effect when filtering total reads against mapping quality. (PDF)

S3 Fig Coverage plots for real *PKD1* data and for variant simulation data by Wgsim in the duplicated region (exons 1–33). (PDF)

S4 Fig CNV detection in exon 22 for patient 30. (PDF)

S5 Fig CNV detection of *HNF1B* by sequence capture-based NGS approach. (PDF)

S6 Fig Differential diagnosis can be challenging in patients with polycystic kidney disease. (PDF)

S7 Fig Coverage achieved by our sequence capture approach compared with available exome capture data. (PDF)

S8 Fig Specific alignment of reads from the genuine *PKD1* gene and duplicated regions to their correct locations. (PDF)

S9 Fig Examples for superior performance of our NGS based approach versus LR-PCR-Sanger sequencing. (PDF)

S1 Table List of all genes targeted by the NimbleGen SeqCap EZ choice library. (PDF)

S2 Table Coverage statistics of all target regions and the *PKD1* locus. (PDF)

S3 Table Coverage statistics of all target genes sequenced and analysed in parallel to *PKD1*x. (PDF)

S4 Table List of all variants included in this study as detected by conventional LR-PCR-Sanger sequencing and by our NGS approach. (PDF)

S5 Table Sites with discordance between Sanger sequencing and NGS. (PDF)

S6 Table List of all “putatively” pathogenic *PKD1* variants previously reported in patients of the validation cohort. (PDF)

S7 Table List of all variants simulated in the duplicated region of *PKD1*. (PDF)

S8 Table Results from CNV analysis. (PDF)

S9 Table Coverage statistics for a proof-of-principle ultra-deep sequencing setup for *PKD1*. (PDF)
