# Supplementary material for: An Efficient and Comprehensive Strategy for Genetic Diagnostics of Polycystic Kidney Disease
Source: PLoS One. 2015 Feb 3;10(2):e0116680. doi: 10.1371/journal.pone.0116680 (PMC4315576; doi:10.1371/journal.pone.0116680)
Supplement: S1 Materials And Methods — (PDF) [file pone.0116680.s001.pdf]

Supporting Information Materials and Methods

An efficient and comprehensive strategy for genetic diagnostics of polycystic kidney disease

Tobias Eisenberger[1], Christian Decker[1], Milan Hiersche[1], Ruben Hamann[1], Eva Decker[1], Steffen Neuber[1], Valeska Frank[1], Hanno J. Bolz[1,2], Kay Latta[3], Carsten Bergmann[1,4]

[1]Bioscientia Center for Human Genetics, Ingelheim, Germany
[2]Institute of Human Genetics, University Hospital of Cologne, Germany
[3]Clementine Children's Hospital, Frankfurt, Germany
[4]Renal Division, Department of Medicine, University Freiburg Medical Center

Supporting Materials and Methods

NimbleGen SeqCap EZ choice library design

Target genes see Table S1.

Settings: The majority of the probe set contains probes with up to 10 close matches in the genome as determined by the SSAHA (Sequence Search and Alignment by Hashing Algorithm) algorithm.

Rebalancing settings: Probes covering "Rebalancing_High Bait"-regions have been replicated 12x:

Rebalancing_High Bait					
**gene**	**exon**	**chr**	**start**	**stop**	**replicate probes**
PKD1	1	16	2185400	2185899	12x
PKD1	15	16	2160943	2160967	12x
PKD1	42	16	2141400	2141623	12x
PKD1	43	16	2141066	2141082	12x
PKD2	1	4	88928799	88929480	12x
MUC1	2-4	1	155160939	155162036	12x

Details of the NimbleGen SeqCap design:

```
Genome: hg19
Offset in bases 0    100
Consolidated/Padded Regions     815  815
Target Bases    299826    299826
Target bases covered 275258    290886
Percent target bases covered    91.8 97.0
Target bases not covered   24568 8940
Percent target bases not covered     8.2  3.0
```

Sequence Capture and Next Generation Sequencing

Libraries were prepared using NEBNext® DNA library prep master mix set for Illumina (New England Biolabs) according to the manufacturer´s protocol. 1 µg of genomic DNA in 52µl of 1X low TE buffer (10 mmol/l Tris; 0.1 mmol/l EDTA) per sample was sheared to generate DNA fragments of 250-300 bp using the Covaris™ S2 AFA system (Covaris Inc.) with the following settings: duty cycle 10%, intensity 5.0, 200 cycles per burst, 2x60 s, temperature at 6°C, power mode: frequency sweeping. Sheared DNA was finally enzymatically end-repaired, A-tailed and ligated to Illumina specific paired-end adaptors for multiplexing, purified in between using AMPure XP beads (Beckman Coulter) according to the NEBNext® protocol. Pre-capture PCR amplification was carried out with eight cycles (10 s at 98°C; 30 s at 65°C; 30 s at 72°C; final elongation at 72°C for 5 min) using the supplied universal and index primers and purification of the PCR products was performed using AMPure XP beads. Pre-capture amplified samples were quantified using Qubit reagents (Life Technologies) and size-controlled (peak at 300-400 bps) with a DNA7500 chip on a Bioanalyzer 2100 instrument (Agilent Technologies). Capture of *PKD1* and all other target genes was conducted following the instructions of the NimbleGen SeqCap EZ Library SR User´s Guide v3.0 (downloadable from the NimbleGen website). Pre-capture amplified samples were pooled – 10 samples for MiSeq and up to 23 samples for HiSeq 1500 sequencing – with a final amount of 1.1 µg and hybridized to the customized in-solution capture library for 72 hours at 47°C. Enrichment was carried out making use of biotinylated capture baits using M-270 streptavidin Dynabeads (Life Technologies) and purification performed with stringent to less stringent washing conditions according to the manufacturer´s protocol. Post-capture amplification by ligation mediated (LM) PCR was conducted according to pre-capture PCR with 19 cycles using the general primers TS-PCR 1 and 2. Post-capture amplified DNA was purified using the QIAquick PCR purification kit (QIAGEN) and again size controlled with a DNA7500 chip on a Bioanalyzer 2100 instrument (Agilent Technologies). Enrichment was checked by qPCR using internal controls supplied by NimbleGen according to the user´s guide. The amplified enriched DNA was used as input for direct cluster generation and sequencing on an Illumina MiSeq™ system (2 x 150 bp PE reads, MiSeq Reagent Kit V2 – 300 cycles) or for cluster generation on a cBot system and sequencing in rapid mode (23 samples per lane, altogether up to 46 samples) on an Illumina HiSeq 1500™ instrument (2 x 150 bp PE reads) (Illumina). For latter sequencing setup the TruSeq Rapid Duo cBot Sample loading kit, the TruSeq Rapid PE Cluster Kit HS and the TruSeq Rapid SBS Kit HS (200 and 2x 50 cycles) were used.

Bioinformatic pipeline and used bioinformatic scripts

Image analysis, base calling and de-multiplexing of reads was conducted using the MiSeq Reporter Software (2.4.60.8) and in case of the Illumina HiSeq™ 1500 system, BCL files were converted to FASTQ files via bcl2fastq (CASAVA , version 1.8.4).

```
# convert bcl files
configureBclToFastq.pl --input-dir ./Data/Intensities/BaseCalls/ --
output-dir FASTQ --sample-sheet ./SampleSheetBcl2fastq.csv --fastq-
cluster-count 15000000 --no-eamss --force
make -j 8
```

Demultiplexed reads from Illumina MiSeq™ and HiSeq™ sequencers were trimmed using Trim Galore! (Babraham Institute, Cambridge, CB22 3AT, http://www.bioinformatics.babraham.ac.uk/projects/trim_galore/) against forward and reverse adapter sequence and mapped against the hg19 human reference genome using the Burrows- Wheeler Aligner, BWA v0.7.8 [1] with the BWA-MEM alignment algorithm and the recommended standard settings.

```
# Trimming
trim_galore --phred33 -a AGATCGGAAGAGCACACGTCTGAACTCCAGTCA -a2
AGATCGGAAGAGCGTCGTGTAGGGAAAGAGTGT --paired
$tmp_dir/$patient.R1.fastq $tmp_dir/$patient.R2.fastq -o $tmp_dir
```

```
# BWA paired end mapping
bwa mem -t $cpu_number -a -M -R
"@RG\tID:$patient\tCN:$institution\tDS:$panel\tDT:`date "+%Y-%m-
%d"`\tLB:$platform\tPL:$platform\tPU:$platform\tSM:$patient"
$grch37_bwa_idx $tmp_dir/$patient.R1_val_1.fq
$tmp_dir/$patient.R2_val_2.fq > results/$patient/$file.sam
```

Reads were then preprocessed with SAMtools v0.1.19 [2] to convert the SAM files into BAM files and sorted on coordinate order:

```
# SAM to BAM file conversion
samtools view -bS -@ $cpu_number results/$patient/$file.sam >
results/$patient/$file.bam
```

```
# Sorting BAM file
samtools sort -@ $cpu_number results/$patient/$file.bam
results/$patient/$file.sorted
```

Duplicate reads were marked with MarkDuplicates from Picard v1.112 (http://picard.sourceforge.net).

```
# Duplicate read filter
java -Xmx10G -jar MarkDuplicates.jar VALIDATION_STRINGENCY=SILENT
I=results/$patient/$file.sorted.bam
METRICS_FILE=results/$patient/$file.mdup.metrics
O=results/$patient/$file.sorted.mdup.bam REMOVE_DUPLICATES=true
ASSUME_SORTED=true READ_NAME_REGEX='[@A-Z0-9]+:[0-9]+:[-A-Z0-9]+:[0-
9]+:([0-9]+):([0-9]+):([0-9]+).*'

# Index BAM file
samtools index results/$patient/$file.sorted.mdup.bam
```

For local realignment and base quality score recalibration of the mapped reads the tools RealignerTargetCreator, IndelRealigner, BaseRecalibrator and PrintReads from GATK (Genome Analysis Toolkit) v3.1 software package [3] were applied. All tools were used with the recommended standard settings. This workflow is in accordance with the best practices from the Broad Institute.

```
# Indel realignment
java -Xmx10G -jar GenomeAnalysisTK.jar -nt 24 -nct 1 -T
RealignerTargetCreator -dt NONE -I
results/$patient/$file.sorted.mdup.bam -R $genome -known
$indelfile1KG -known $millsfile -L
$intervals_path/$panel.bait.100.intervals -o
results/$patient/$patient.realigner.intervals
java -Xmx10G -jar GenomeAnalysisTK.jar -T IndelRealigner -dt NONE -I
results/$patient/$file.sorted.mdup.bam -R $genome --
filter_bases_not_stored -targetIntervals
results/$patient/$patient.realigner.intervals -known $indelfile1KG -
known $millsfile -L $intervals_path/$panel.bait.100.intervals -o
results/$patient/$file.sorted.mdup.realigned.bam
```

```
# Base recalibration
java -Xmx10G -jar GenomeAnalysisTK.jar -nct 8 -T BaseRecalibrator -
dt NONE -I results/$patient/$file.sorted.mdup.realigned.bam -R
$genome -cov CycleCovariate -cov ContextCovariate -knownSites
$dbsnpfile -knownSites $indelfile1KG -knownSites $millsfile -L
$intervals_path/$panel.bait.100.intervals -o
results/$patient/$patient.recal_data.grp
java -Xmx10G -jar GenomeAnalysisTK.jar -nct 8 -T PrintReads -dt NONE
-I results/$patient/$file.sorted.mdup.realigned.bam -R $genome -BQSR
results/$patient/$patient.recal_data.grp -L
$intervals_path/$panel.bait.100.intervals -o
results/$patient/$file.sorted.mdup.realigned.recal.bam
```

Variants were called with the tool UnifiedGenotyper from GATK.

```
# Variant calling
java -Xmx10G -jar GenomeAnalysisTK.jar -nt 24 -nct 1 -T
UnifiedGenotyper --pcr_error_rate 0 --min_base_quality_score 10 -dt
NONE -I results/$patient/$file.sorted.mdup.realigned.recal.bam -R
$genome -rf BadCigar -A DepthPerAlleleBySample -A AlleleBalance -glm
SNP -stand_call_conf 10.0 -stand_emit_conf 30.0 --dbsnp $dbsnpfile -
L $intervals_path/$panel.bait.100.intervals -o
results/$patient/SNV_InDel/$file.ug.SNP
java -Xmx10G -jar GenomeAnalysisTK.jar -nt 24 -nct 1 -T
UnifiedGenotyper --pcr_error_rate 0 --min_base_quality_score 10 -dt
NONE -I results/$patient/$file.sorted.mdup.realigned.recal.bam -R
$genome -rf BadCigar -A DepthPerAlleleBySample -glm INDEL -
minIndelCnt 3 -minIndelFrac 0 -stand_call_conf 10.0 -stand_emit_conf
30.0 --dbsnp $dbsnpfile -L $intervals_path/$panel.bait.100.intervals
-o results/$patient/SNV_InDel/$file.ug.INDEL
```

```
# Variant filtation
java -Xmx10G -jar GenomeAnalysisTK.jar -T VariantFiltration -dt NONE
-R $genome --filterExpression "QD < 2.0" --filterName QDFilter --
filterExpression "MQ < 40.0" --filterName MQFilter --
filterExpression "FS > 60.0" --filterName FSFilter --
filterExpression "HaplotypeScore > 13.0" --filterName
```

```
HaplotypeScoreFilter --filterExpression "MQRankSum < -12.5" --
filterName MQRankSumFilter --filterExpression "ReadPosRankSum < -
8.0" --filterName ReadPosRankSumFilter --variant
results/$patient/SNV_InDel/$file.ug.SNP -o
results/$patient/SNV_InDel/$file.ug.SNP.flt
java -Xmx10G -jar GenomeAnalysisTK.jar -T VariantFiltration -dt NONE
-R $genome --filterExpression "QD < 2.0" --filterName QDFilter --
filterExpression "ReadPosRankSum < -20.0" --filterName
ReadPosRankSumFilter --filterExpression "FS > 200.0" --filterName
FSFilter --variant results/$patient/SNV_InDel/$file.ug.INDEL -o
results/$patient/SNV_InDel/$file.ug.INDEL.flt

# Left align and trim variants
java -Xmx10G -jar GenomeAnalysisTK.jar -T LeftAlignAndTrimVariants -
dt NONE -R $genome --variant
results/$patient/SNV_InDel/$file.ug.INDEL.flt -L
$intervals_path/$panel.bait.100.intervals -o
results/$patient/SNV_InDel/$file.ug.INDEL.flt.lat

# Combining variants
java -Xmx10G -jar GenomeAnalysisTK.jar -nt 24 -T CombineVariants -dt
NONE -R $genome -genotypeMergeOptions UNIQUIFY --
variant:$file.ug.SNP.flt results/$patient/SNV_InDel/$file.ug.SNP.flt
--variant:$file.ug.INDEL.flt.lat
results/$patient/SNV_InDel/$file.ug.INDEL.flt.lat -o
results/$patient/SNV_InDel/$file.ug.SNP.flt.$file.ug.INDEL.flt.lat.c
omb
```

Mapping and coverage statistics for the *PKD1* regions were calculated using the tool DepthOfCoverage from GATK, as well as the tool CalculateHsMetrices from Picard.

```
# Calculating coverage
java -Xmx10G -jar $tools_path/picard/CalculateHsMetrics.jar
BAIT_INTERVALS=$intervals_path/$panel.bait.intervals
TARGET_INTERVALS=$intervals_path/$panel.target.10.intervals
I=results/$patient/$file.sorted.mdup.realigned.recal.bam
O=results/$patient/$patient.HSMetrices REFERENCE_SEQUENCE=$genome
VALIDATION_STRINGENCY=SILENT
```

```
java -Xmx10G -jar GenomeAnalysisTK.jar -T DepthOfCoverage -dt NONE -
I results/$patient/$file.sorted.mdup.realigned.recal.bam -R $genome
-rf BadCigar --includeDeletions --omitDepthOutputAtEachBase --
summaryCoverageThreshold 5 --summaryCoverageThreshold 10 --
summaryCoverageThreshold 15 --summaryCoverageThreshold 20 --
summaryCoverageThreshold 25 --summaryCoverageThreshold 30 --
summaryCoverageThreshold 40 --summaryCoverageThreshold 50 --
summaryCoverageThreshold 60 --summaryCoverageThreshold 70 --
summaryCoverageThreshold 80 --summaryCoverageThreshold 90 --
summaryCoverageThreshold 100 --summaryCoverageThreshold 110 --
summaryCoverageThreshold 120 --summaryCoverageThreshold 130 --
summaryCoverageThreshold 140 --summaryCoverageThreshold 150 --
summaryCoverageThreshold 160 --summaryCoverageThreshold 170 --
summaryCoverageThreshold 180 --summaryCoverageThreshold 190 --
summaryCoverageThreshold 200 -L
$intervals_path/$panel.target.10.intervals -o
results/$patient/$patient.targetCoverage
```

The identified variants were annotated using ANNOVAR and the RefSeq gene-based annotation method.

```
# Gene annotation
convert2annovar.pl
results/$patient/SNV_InDel/$file.ug.SNP.flt.$file.ug.INDEL.flt.lat.c
omb --format vcf4 --includeinfo >
results/$patient/SNV_InDel/$patient.avinput

annotate_variation.pl --geneanno --dbtype refGene --hgvs --exonsort
--exonicsplicing --splicing_threshold 10 --buildver hg19
results/$patient/SNV_InDel/$patient.avinput -outfile
results/$patient/SNV_InDel/$patient.avinput
$flat_file_dbs_path/annovar/
```

After this, all variants were checks against the population frequency databases 1000 Genomes Project (1000g2012apr), Exome Sequencing Project (esp6500si_all).

```
# Database annotation
annotate_variation.pl --filter --dbtype 1000g2012apr_all --buildver
hg19 results/$patient/SNV_InDel/$patient.avinput
$flat_file_dbs_path/annovar/

annotate_variation.pl --filter --dbtype esp6500si_all --buildver
hg19 results/$patient/SNV_InDel/$patient.avinput
$flat_file_dbs_path/annovar/
```

Annotation (dbNSFP, HGMD), functional prediction and classification of identified variants was conducted as described previously [4],[5]. Additionally, *PKD1* variants were annotated with the entry from the ADPKD Mutation Database Version 3.0 (http://pkdb.mayo.edu/).

For detection of variants in the *PKD1* gene the following criteria were applied:

1. Variants were included in the analysis when ≥ 20% of total reads at the position showed the alteration. A variant was called homozygous when ≥ 85% of all reads had the variation.

Further filtering of the variants in routine testing against minor allele frequency (MAF≤1%) and *in silico* predicted pathogenicity is performed as previously described [4] with nonsense, frameshift and canonical splice site variants being considered pathogenic.

Copy number variation analysis

We performed copy number variation (CNV) analysis on highly covered samples sequenced on the Illumina Hiseq1500™ system. Potential copy number alterations (CNA) were initially identified with the tools copynumber and copyCaller from VarScan v2.3.6 [6] on mapped reads with a maximum segment size of 300. All other parameter were used with standard settings. Thereby coverage of every target region of the sample of interest was internally normalized and compared versus normalized control data of other samples of the same run. CNVs were annotated using RefSeq gene file from UCSC (ftp://hgdownload.cse.ucsc.edu/goldenPath/hg19/database/refGene.txt.gz). Potential CNVs were initially taken into account, if the CNV was detected by VarScan against at least 85 % of the control patients and if the $\log_2$ threshold was ≥ 0.6 in case of an amplification or ≤ -0.6 in case of a deletion.

#CNV detection

```
samtools mpileup -B -f $genome results/$test_patient/$file.bam >
results/$test_patient/CNV/$test_patient.mpileup

samtools mpileup -B -f $genome results/$control_patient/$file.bam >
results/$control_patient/CNV/$control_patient.mpileup
```

The on target base ratio was calculated based on the value “ON_TARGET_BASES” in the *.HSMetrices from Picard. In detail, number of on target bases from the control patient was divided by the number of on target bases from the test patient. The result serves as the normalization factor for the parameter —data-ration of VarScan copynumber tool.

```
on_target_bases_ratio=`get_on_target_bases_ratio
results/$control_patient/$control_patient.HSMetrices
results/$test_patient/$test_patient.HSMetrices`

java -Xmx10G -jar VarScan.jar copynumber
results/$control_patient/CNV/$control_patient.mpileup
results/$test_patient/CNV/$test_patient.mpileup
results/$test_patient/CNV/${test_patient}_${control_patient} --max-
segment-size 300 --data-ratio $on_target_bases_ratio

java -Xmx10G -jar VarScan.jar copyCaller
results/$test_patient/CNV/${test_patient}_${control_patient}.copynum
ber --min-tumor-coverage 11 --max-homdel-coverage 10 --output-file
results/$test_patient/CNV/${test_patient}_${control_patient}.called
--output-homdel-file
results/$test_patient/CNV/${test_patient}_${control_patient}.called.
homdel
```

ClipCrop [7] program was used as split read method for identification of soft-clipped reads in *PKD1*.

```
clipcrop.js  patient_46.sam  ~/grch37.70.fa.chr  --bases_around_break
1000
```

The Smith-Waterman alignment of breakpoint read sequences was performed versus the entire DNA sequence of *PKD1*, and, as suggested by coverage-based CNV analysis, the best match for the breakpoint at 16:2161589 in patient 46 was found in the intronic region between exons 21 and 22.

```
# obtain full split read sequence from original sam (fastq file emitted by clipcrop contains only the unaligned (split) part of reads)
# the following code has to be run in an R shell

splitreads <- grep('^@', readLines('mapped.sam'), invert = TRUE, value = TRUE)
splitreads <- do.call(rbind, lapply(strsplit(splitreads, split = '::'), function(x) x[1:3]))
splitreads <- unlist(lapply(
        splitreads[, 1],
        function(ids.to.find) {
          system(paste("grep ", paste(" -e '", ids.to.find, "' ", collapse = '', sep = ''), " patient_46.sam", sep = ''), intern = TRUE)
        }
))
splitreads.df <- do.call(rbind, lapply(splitreads, function(x) strsplit(x, split = '\t')[[1]][1:10]))

# realign longest split read at breakpoint in question
bp <- 2161589
chr <- 16

reads.df <- splitreads.df[splitreads.df[, 3] == chr &
splitreads.df[, 4] == bp, ]

# get 4 MB window of reference genome to align to
require(seqinr)
align.ref <- paste(system(paste('samtools faidx ./data/grch37.70.fa ', chr, ':', bp - 2000000, '-', bp + 2000000, sep = ''), intern = TRUE), collapse = '')
split.readstart.idx <- which(regexpr('^\\d+S', reads.df[, 6]) == 1)
```

```
splitlength.readstart <- sub("S", '', regmatches(reads.df[, 6],
regexpr('^\\d+S', reads.df[, 6])))
split.readend.idx <- which(regexpr('\\d+S$', reads.df[, 6]) == 1)
splitlength.readend <- sub("S", '', regmatches(reads.df[, 6],
regexpr('\\d+S$', reads.df[, 6])))
# find split read with the longest unaligned split part and align it
if(length(splitlength.readstart) > length(splitlength.readend) ) {
  reads.splitpart <- substr(reads.df[split.readstart.idx, 10], 1,
splitlength.readstart)
} else  {
  reads.splitpart <- substr(reads.df[split.readend.idx, 10],
nchar(reads.df[, 10]) - as.numeric(splitlength.readend),
nchar(reads.df[, 10]))
}
require(Biostrings)
globalAlign <- pairwiseAlignment(align.ref,
reads.splitpart[which.max(nchar(reads.splitpart))], type = 'local',
gapOpening = -2000, gapExtension = -10)
writePairwiseAlignments(globalAlign, paste(chr, '-', bp, '.align',
sep = ''))
```

To further unravel the genomic structure at the CNV breakpoint, we applied the GATA tool [8] facilitating a more general motif search. Application of the GATA tool decomposed the read sequence to short fragments in a sliding window approach (using windows of 20 bases) and subsequently mapped all fragments to the *PKD1* sequence via BLASTN.

```
# read with the longest split part to be aligned in GATA (derived
from the previous analysis)
>HWI-C00116:32:H8LGAADXX:2:2104:8694:69676 119S32M
GAAGGAGGGGGAGGGGAGGAAAGGAGGAGGGAAGGACAGGAGGGGGAGGGGAGGAGAGAGGAGGGGGA
TGAGGGGAGGAGAGGAGGGGGGAGGAGAGGAGTGGGGAGGAGAGGGGTGGGGACCTCCAGGCGCACGG
GGTAGGTGCCCCTCG
```

```
# the parameters of the graphical GATA user interface
```

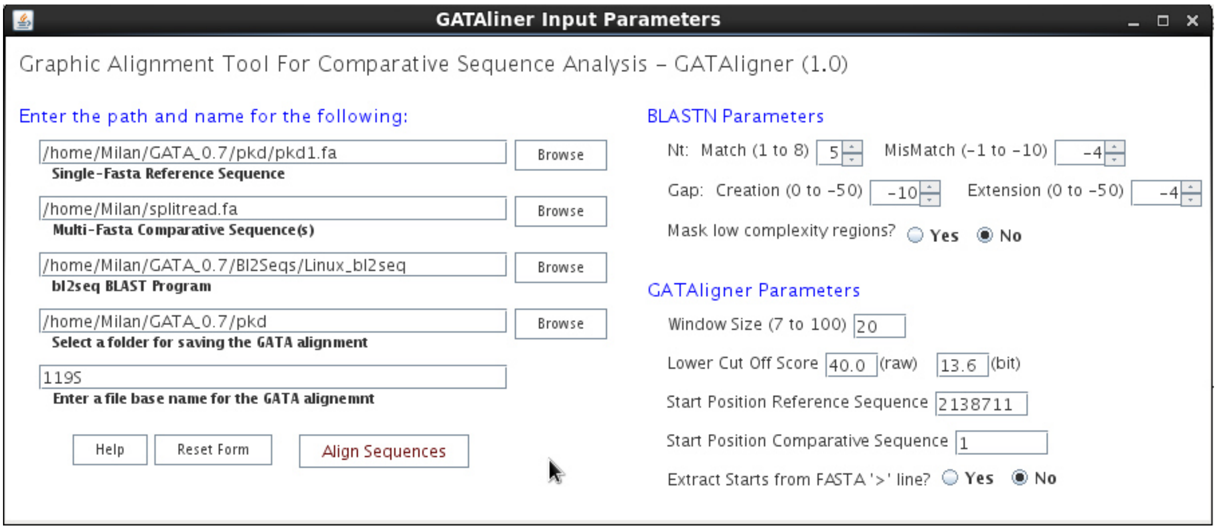

Read simulation

In a first step, FASTA files for *PKD1* exons 1-33 where extracted from the hg19 reference genome using exon coordinates from the RefSeq gene file (ftp://hgdownload.cse.ucsc.edu/ goldenPath/hg19/database/refGene.txt.gz) as well as the FASTA files for *PKD1* pseudogenes *PKD1P1-6* using hg19 coordinates from GeneCards (e.g. http://www.genecards.org/cgi-bin/carddisp.pl?gene=PKD1P1) [9]. For all locations we added 20 bp of flanking sequences. Next, we calculated the length for every location and the amount of simulated reads to achieve an average coverage which will be equal to those of a MiSeq run under the assumption that we need 1000X coverage for every sequencing fragment with a length of 200 bp (Figure S3). After this, we simulated the 2 x 150 bp paired end reads in FASTQ format with Wgsim from the SAMtools package (Li, H. wgsim - read simulator for next generation sequencing). For the parameter of Wgsim we used a base error rate of 0.001, an outer distance between the two ends of 200 bp and a length of the first and second read of 150 bp for all locations. For the reads of *PKD1* exons 1-33 we simulated a mutation rate of 0.02 and for *PKD1P1-6* a rate of 0 which simulates wild-type reads with a base error rate only and no mutations. The number of simulated paired end reads was adjusted to the appropriate location. All other parameters were used with standard settings. After all reads had been simulated, we merged all simulated FASTQ files into one file for R1 and R2 respectively and analyzed them with our in-house bioinformatic pipeline (see Bioinformatic data analysis).

```
# Exemplary simulation of reads for PKD1 exon 1
wgsim -e 0.001 -d 200 -N 1000 -1 150 -2 150 -r 0.02 PKD1_Exon1-33_FASTA/PKD1.E1.fa PKD1_Exon1-33_SimReads/PKD1.E1.R1.fq PKD1_Exon1-33_SimReads/PKD1.E.R2.fq

# Exemplary simulation of reads for PKD1P1 exon 1
wgsim -e 0.001 -d 200 -N 165900 -1 150 -2 150 -r 0
Pseudogenes_FASTA/PKD1P1.fa PKD1P1-6_SimReads/PKD1P1.R1.fq PKD1P1-6_SimReads/PKD1P1.R2.fq
```

Sanger sequencing of *PKD1* exons 10 and 11

For reassessment of the recurring false positive calls in exon 10 and 11 by LR-PCR Sanger sequencing alternative primer pairs were used, described in [10,11,12].

Long-range PCR (LR-PCR) primer		
**exon**	**primer sequence**	**reference**
2-12	F: 5´-CCAGCTCTCTGTCTACTCACCTCCGCATC-3´	10,11
2-12	R: 5´-CCACGGTTACGTTGTAGTTCACGGTGACG-3´	10
2-12	R: 5´-CTGCATCCTGTTCATCCGCTCCACGGTTAC-3´	11
6-11	F: 5´-GCTGCCCACTCCCCTTCCTC-3´	12
6-11	R: 5´-GGAATGAGTTAGCGGAGCCA-3´	12
Sequencing Primers		
10	F1: 5´-GTCCTCACAGCAAGGCCAGGATT-3´	-
11	F1a: 5´-GTGTCCACCCTCATCCGTCGTGCG-3´	-
11	F1b: 5´-CCGGCTGCCCCTGGGAGACCAAC-3´	-
11	R1: 5´-CAGTGGAATGAGTTAGCGGAGCCA-3´	-
10	F2: 5´-GCTGGGAGTGCTGCCCAGGT-3´	12
10	R: 5´-TGCAGGCTGGGTGTGTCTGG-3´	12
11	F2: 5´-AGCACTGTGGGGAGGCTCCT-3´	12
11	R2: 5´-TAAAGCCCACCAGGTAGCCC-3´	12

References

1. Li H, Durbin R (2009) Fast and accurate short read alignment with Burrows-Wheeler transform. Bioinformatics 25: 1754-1760.
2. Li H, Handsaker B, Wysoker A, Fennell T, Ruan J, et al. (2009) The Sequence Alignment/Map format and SAMtools. Bioinformatics 25: 2078-2079.
3. McKenna A, Hanna M, Banks E, Sivachenko A, Cibulskis K, et al. (2010) The Genome Analysis Toolkit: a MapReduce framework for analyzing next-generation DNA sequencing data. Genome Res 20: 1297-1303.
4. Eisenberger T, Neuhaus C, Khan AO, Decker C, Preising MN, et al. (2013) Increasing the yield in targeted next-generation sequencing by implicating CNV analysis, non-coding exons and the overall variant load: the example of retinal dystrophies. PLoS One 8: e78496.
5. Eisenberger T, Di Donato N, Baig SM, Neuhaus C, Beyer A, et al. (2014) Targeted and genomewide NGS data disqualify mutations in MYO1A, the "DFNA48 gene", as a cause of deafness. Hum Mutat 35: 565-570.
6. Koboldt DC, Zhang Q, Larson DE, Shen D, McLellan MD, et al. (2012) VarScan 2: somatic mutation and copy number alteration discovery in cancer by exome sequencing. Genome Res 22: 568-576.
7. Suzuki S, Yasuda T, Shiraishi Y, Miyano S, Nagasaki M (2011) ClipCrop: a tool for detecting structural variations with single-base resolution using soft-clipping information. BMC Bioinformatics 12 Suppl 14: S7.
8. Nix DA, Eisen MB (2005) GATA: a graphic alignment tool for comparative sequence analysis. BMC Bioinformatics 6: 9.
9. Saran M, Dalah I, Alexander J, Rosen N, Iny Stein T, et al. (2010) GeneCards Version 3: the human gene integrator. Database (Oxford) 2010: baq020.
10. Rossetti S, Chauveau D, Walker D, Saggar-Malik A, Winearls CG, et al. (2002) A complete mutation screen of the ADPKD genes by DHPLC. Kidney international 61: 1588-1599.
11. Rossetti S, Hopp K, Sikkink RA, Sundsbak JL, Lee YK, et al. (2012) Identification of gene mutations in autosomal dominant polycystic kidney disease through targeted resequencing. J Am Soc Nephrol 23: 915-933.
12. Audrezet MP, Cornec-Le Gall E, Chen JM, Redon S, Quere I, et al. (2012) Autosomal dominant polycystic kidney disease: comprehensive mutation analysis of PKD1 and PKD2 in 700 unrelated patients. Hum Mutat 33: 1239-1250.
