# Supplementary material for: An Efficient and Comprehensive Strategy for Genetic Diagnostics of Polycystic Kidney Disease
Source: PLoS One. 2015 Feb 3;10(2):e0116680. doi: 10.1371/journal.pone.0116680 (PMC4315576; doi:10.1371/journal.pone.0116680)
Supplement: S1 Fig — (PDF) [file pone.0116680.s002.pdf]

# Figure S1

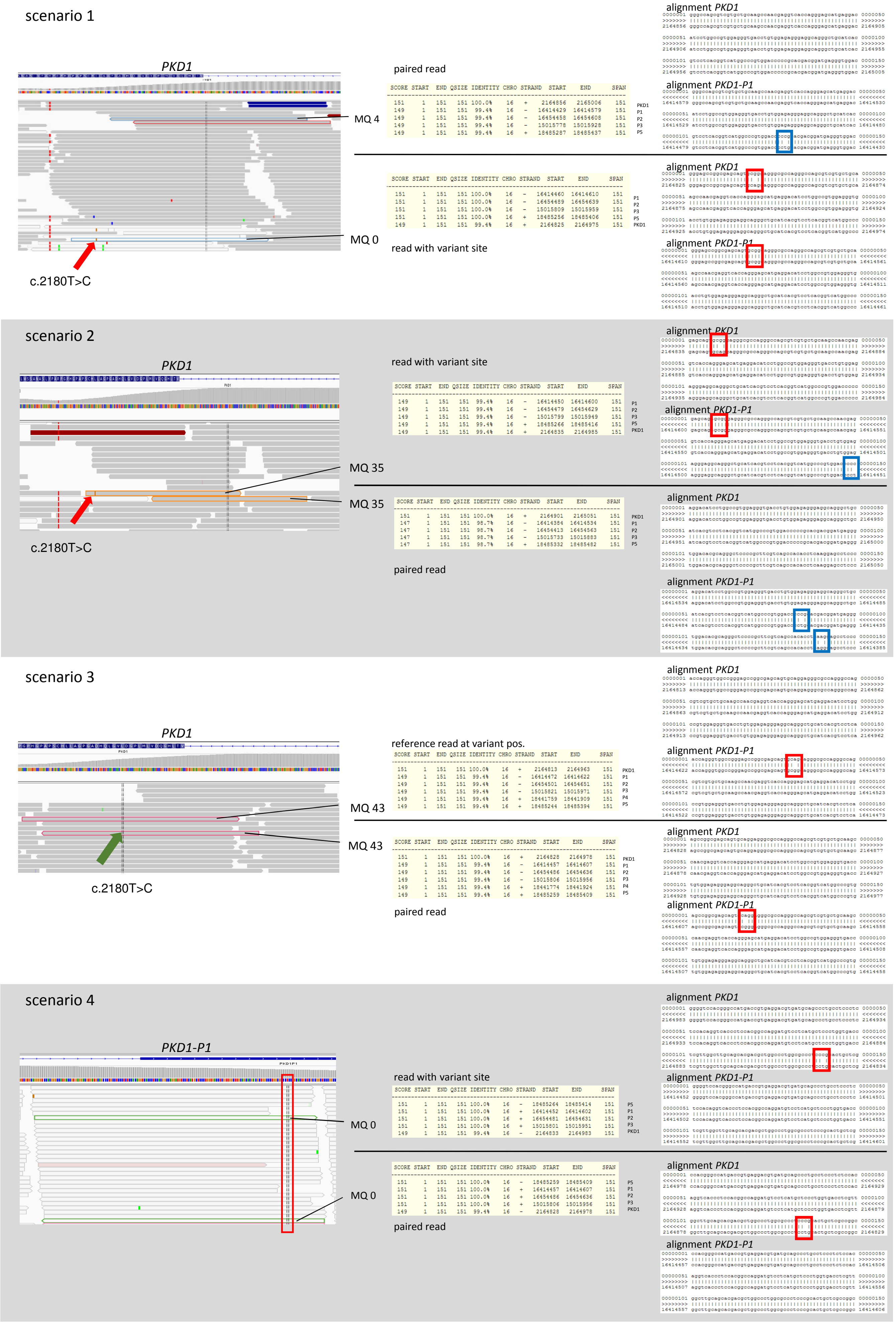

scenario 1
PKD1
MQ 4
MQ 0
c.2180T>C
paired read
read with variant site
alignment PKD1
alignment PKD1-P1
alignment PKD1
alignment PKD1-P1
scenario 2
PKD1
MQ 35
MQ 35
c.2180T>C
read with variant site
paired read
alignment PKD1
alignment PKD1-P1
alignment PKD1
alignment PKD1-P1
scenario 3
PKD1
MQ 43
MQ 43
c.2180T>C
reference read at variant pos.
paired read
alignment PKD1
alignment PKD1-P1
alignment PKD1
alignment PKD1-P1
scenario 4
PKD1-P1
MQ 0
MQ 0
read with variant site
paired read
alignment PKD1
alignment PKD1-P1
alignment PKD1
alignment PKD1-P1

**Figure S1. Challenging discriminative mapping between master gene and pseudogene regions at one critical site.**

Inspection of the alignment at the site c.2180T>C (p.Leu727Pro), which could in one case not been detected even with relaxed filter criteria. The variant at this site is present in several pseudogenes (*PKD1-P1,-P2,-P3,-P5*) favoring mapping to these sites for reads without additional sequence divergence between *PKD1* and the homologous regions. However, few reads are present in the alignment that map to the master gene due to discrimination enabled by the second read of this pair. Relaxing filter criteria to include these reads led to detection of all but one critical sites as candidate for further validation. Read pairs were identified in the IGV and read sequences analyzed by Blat [middle: alignment statistics; right: site-by-site alignment of the read (upper line) to the *PKD1* and *PKD1-P1* region (lower line). Alignments to *PKD1* are shown as reverse complementary sequence.] MQ- mapping quality.

Different scenarios are presented to illustrate challenges for any mapping algorithm:

<u>Scenario 1</u>: On the left alignment at the *PKD1* locus (IGV) is displayed with inspection of the blue labelled read pair. The read with the variant site of interest (red box on the right) shows 100% sequence identity to four pseudogenes resulting in zero mapping quality. However, the second read of the pair shows one sequence divergence (blue box) between *PKD1* and the homologous regions, so that the algorithm decides to align this pair to the genuine gene despite low mapping quality. Therefore, relaxing filter criteria to include these low frequent read pairs enabled detection of most of these critical sites. Filtering total reads against low mapping quality was not appropriate at this site. Increasing the fragment size to gain more distant read pairs might enlarge the chance of finding a second read pair with at least one sequence divergence between *PKD1* and all homologous regions, depending on surrounding sequence composition.

<u>Scenario 2</u>: On the left alignment at the *PKD1* locus (IGV) is displayed with inspection of the orange labelled read pair. The read with the variant site of interest (red box) equally maps to *PKD1* and four pseudogenes as there is one additional sequence divergence between master gene and homologous regions (blue box). As the second pair introduces specificity of mapping to the *PKD1* locus – two sequence divergences – the pair gains higher mapping quality, but this situations seems to be underrepresented at critical sites. A similar scenario with the variant of interest not being present in one homologous region might enable variant detection at most sites evaluated due to unambiguous mapping of read pairs to the master gene.

<u>Scenario 3</u>: On the left alignment at the *PKD1* locus (IGV) is displayed with inspection of the pink labelled read pair. One read pair not carrying the variant of interest shows 100% sequence identity only to the *PKD1* locus resulting in high mapping quality in the alignment to the genuine gene. This situation is overrepresented at this site requiring lower detection threshold to identify

critical variants. The red box illustrates sequence divergence at the site of interest in the *PKD1-P1* gene.

Scenario 4: On the left alignment at the *PKD1-P1* locus (IGV) is displayed with inspection of the green labelled read pair. As the variant of interest is present in *PKD1-P1* this pair shows 100% identity to this region and cannot be mapped to *PKD1* substracting this read from the total reads at the site in the genuine gene. High sequence homology of *PKD1-P1* to other pseudogenes results in zero mapping quality (reads in white) as seen for most reads in the pseudogene. Therefore, read simulation of mutations in pseudogenes in highly homologous regions failed (data not shown) as unambiguous mapping of read pairs (MQ =0) to one specific pseudogene is problematic maybe due to higher sequence homology among pseudogenes compared to *PKD1* as seen here.
