# Supplementary material for: An Efficient and Comprehensive Strategy for Genetic Diagnostics of Polycystic Kidney Disease
Source: PLoS One. 2015 Feb 3;10(2):e0116680. doi: 10.1371/journal.pone.0116680 (PMC4315576; doi:10.1371/journal.pone.0116680)
Supplement: S2 Fig — (PDF) [file pone.0116680.s003.pdf]

Figure S2

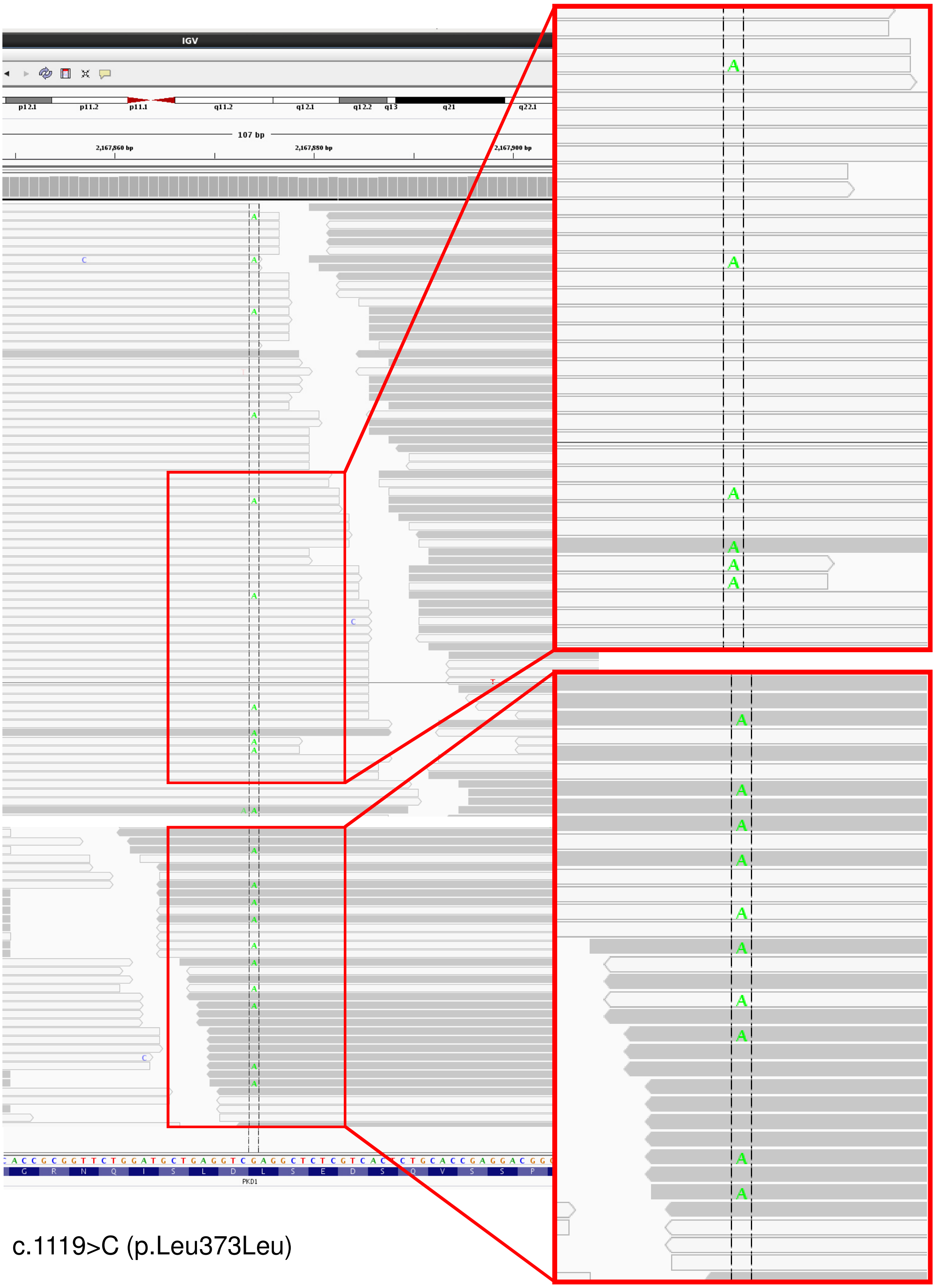

Figure S2. Critical sites with positive effect when filtering total reads against mapping quality.
The alignment (IGV) at position c.1119>C (p.Leu373Leu) in exon 5 shows a high level of reads with low to zero mapping quality (reads in white). This variant is not present in any pseudogene with sequence identity between *PKD1* and the pseudogenes at the site of interest. Although there are reads with low mapping quality carrying the variant, filtering of total reads against their mapping quality resulted in better results (higher percentage of alternative reads, data not shown). The proportion of pairs with higher mapping quality carrying the variant is higher at these sites presumably due to sequence divergence around the site exploited by the second pair of the read. However, in most of the critical regions filtering against MQ did not elevate the read ratio towards more alternative reads.
