# Supplementary material for: An Efficient and Comprehensive Strategy for Genetic Diagnostics of Polycystic Kidney Disease
Source: PLoS One. 2015 Feb 3;10(2):e0116680. doi: 10.1371/journal.pone.0116680 (PMC4315576; doi:10.1371/journal.pone.0116680)
Supplement: S3 Fig — (PDF) [file pone.0116680.s004.pdf]

Figure S3

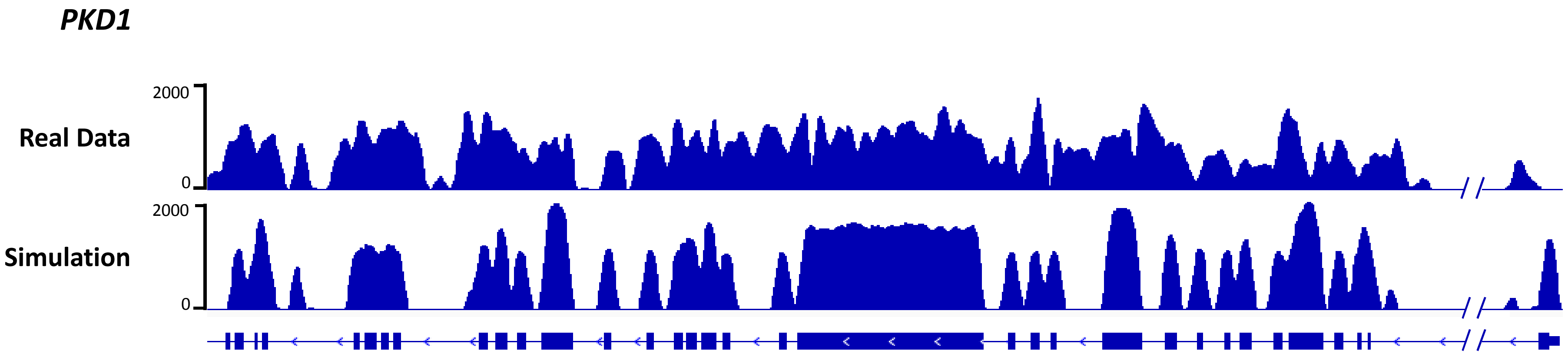

Figure S3. Coverage plots for real *PKD1* data and for variant simulation data by Wgsim in the duplicated region (exons 1-33).
In the real dataset (upper plot) also intronic regions targeted by the probe design are partially covered, whereas read simulation (lower plot) was only performed for exonic regions in the duplicated *PKD1* region. Some spurious alignment from pseudogene regions is visible in the large intron 1 maybe due to repetitive sequence segments. *PKD1* is displayed from the right to the left.
