# Supplementary material for: An Efficient and Comprehensive Strategy for Genetic Diagnostics of Polycystic Kidney Disease
Source: PLoS One. 2015 Feb 3;10(2):e0116680. doi: 10.1371/journal.pone.0116680 (PMC4315576; doi:10.1371/journal.pone.0116680)
Supplement: S4 Fig — (PDF) [file pone.0116680.s005.pdf]

Figure S4

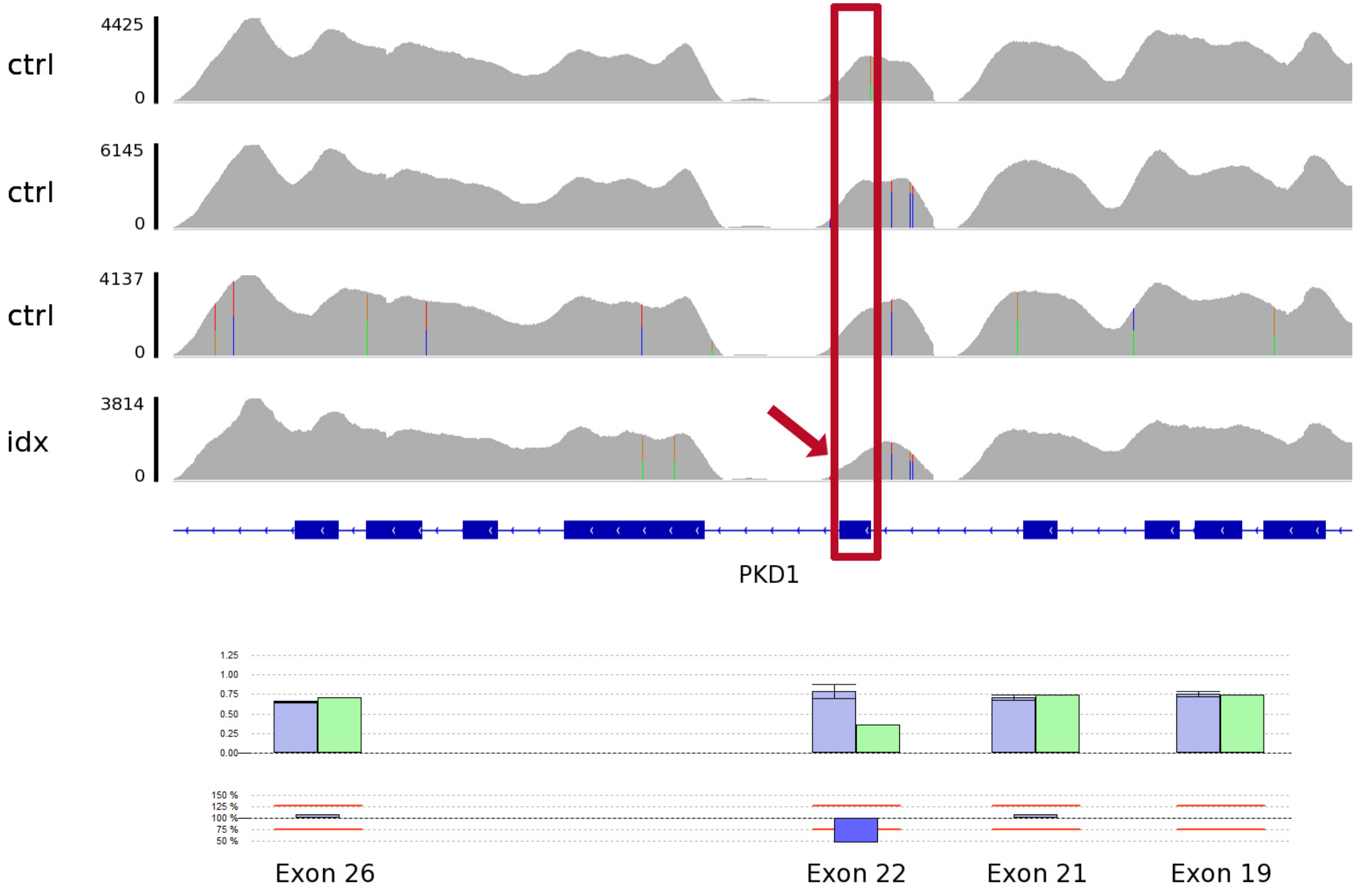

Figure S4. CNV detection in exon 22 for patient 30.

Coverage plots (IGV) of three control (ctrl) patients vs. the index (idx) sample illustrate the statistical readout with a drop in coverage in exon 22 for the index (red arrow) compared to controls indicating a deletion of this exon. Underneath, the result from MLPA analysis is displayed by the MLPA module in the JSI SeqPilot software. The relative peak area (RPA) of the patient result file (green) and of the control result file (blue) with standard deviation (error bar) is shown. The ratio RPA (lower panel) was calculated as RPA of the patient versus controls. Deletions are indicated if the ratio RPA falls below 75% (red line).
