# Supplementary material for: An Efficient and Comprehensive Strategy for Genetic Diagnostics of Polycystic Kidney Disease
Source: PLoS One. 2015 Feb 3;10(2):e0116680. doi: 10.1371/journal.pone.0116680 (PMC4315576; doi:10.1371/journal.pone.0116680)
Supplement: S5 Fig — (PDF) [file pone.0116680.s006.pdf]

Figure S5

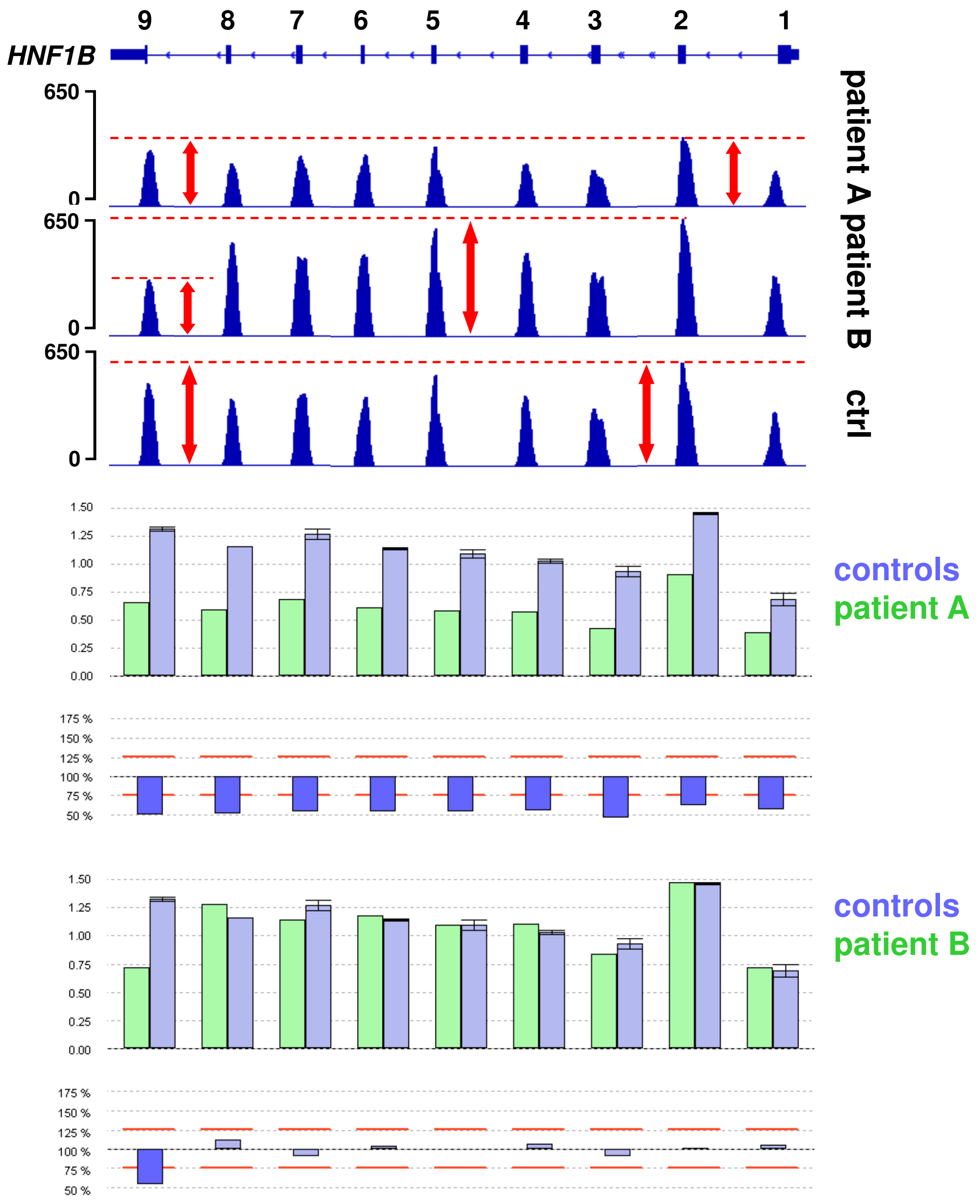

Figure S5. CNV detection of *HNF1B* by sequence capture-based NGS approach.

Coverage plots (IGV) of a control sample (ctrl) vs. patient A (complete *HNF1B* deletion in heterozygous state) or patient B (heterozygous deletion of exon 9) from previous sequence capture-based gene-panel sequencing runs are shown. The statistical readout is illustrated with a lower coverage of all exons or exon 9 (red arrows), respectively, compared to the control sample indicating a deletion of these exons. Below, the results from CNV analyses by the SeqNext module in the JSI SeqPilot software are displayed for each patient calculating and comparing the normalized relative coverage of the samples (patients in green, controls in blue bars). The *HNF1B* gene is displayed from right to left.
