# Supplementary material for: An Efficient and Comprehensive Strategy for Genetic Diagnostics of Polycystic Kidney Disease
Source: PLoS One. 2015 Feb 3;10(2):e0116680. doi: 10.1371/journal.pone.0116680 (PMC4315576; doi:10.1371/journal.pone.0116680)
Supplement: S6 Fig — (PDF) [file pone.0116680.s007.pdf]

Figure S6

Patient 1 Patient 3

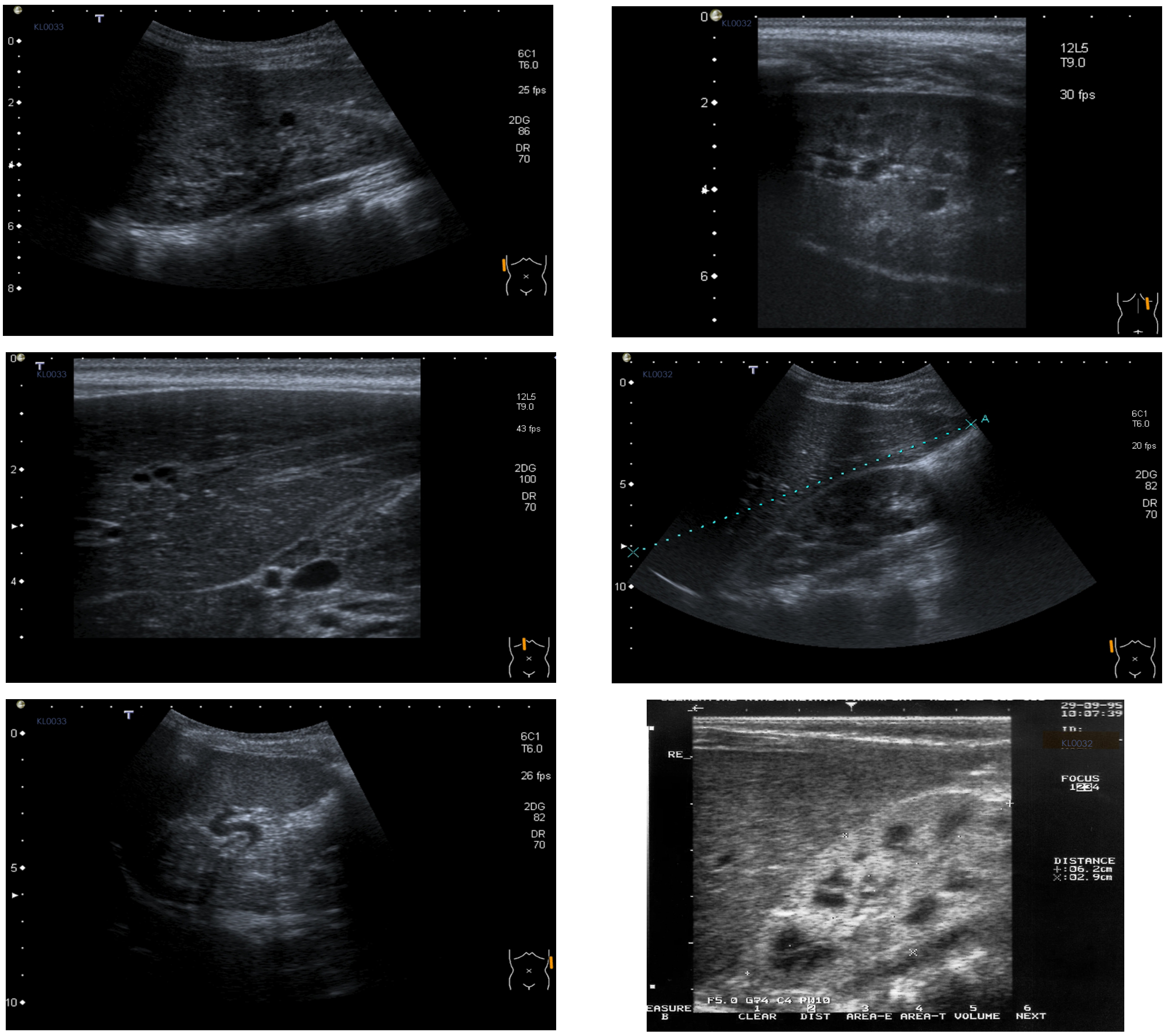

Figure S6. Differential diagnosis can be challenging in patients with polycystic kidney disease.

Patient 1 (left): In this 7-year-old girl with early and severe disease and negative family history ARPKD was initially suspected. Without knowing the *PKD1* genotype, detection of one pathogenic *PKHD1* mutation may have been misleading and indicative for *PKHD1* as the main disease locus. However, PKD in this patient is clearly related to *PKD1* (for details see Table 2). Both kidneys have been enlarged from early on. Cortical echogenicity is shown to be normal containing a few visible cysts (top and middle left). The medulla displays scattered echogenic reflexes suggesting many small tubular cysts. The liver of the infant shows increased echogenic borders of the portal area compatible with mild liver fibrosis (bottom left). The tortuous splenic vein is in accordance with the appearance of an altered liver structure.

Patient 3 (right): This 19-year-old female patient was also initially assumed to be affected with ARPKD because of the ultrasonographic pattern and a negative family history. Initial ultrasound (bottom right) shortly after birth revealed markedly enlarged kidneys with increased echogenicity. Single cysts could not be discerned suggesting ARPKD. Follow-up examinations demonstrated enlarged kidneys with decreased cortico-medullary differentiation, moderately increased echogenicity and a few visible cysts (top and middle right). The liver still appears unremarkable. *PKHD1* mutation analysis previously performed by conventional Sanger sequencing did not show any pathogenic change. Only now with the multi-gene NGS panel available we were able to identify *PKD1* as putatively major disease locus in this patient.
