# Supplementary material for: An Efficient and Comprehensive Strategy for Genetic Diagnostics of Polycystic Kidney Disease
Source: PLoS One. 2015 Feb 3;10(2):e0116680. doi: 10.1371/journal.pone.0116680 (PMC4315576; doi:10.1371/journal.pone.0116680)
Supplement: S7 Fig — (PDF) [file pone.0116680.s008.pdf]

Figure S7

PKD1 Exome coverage

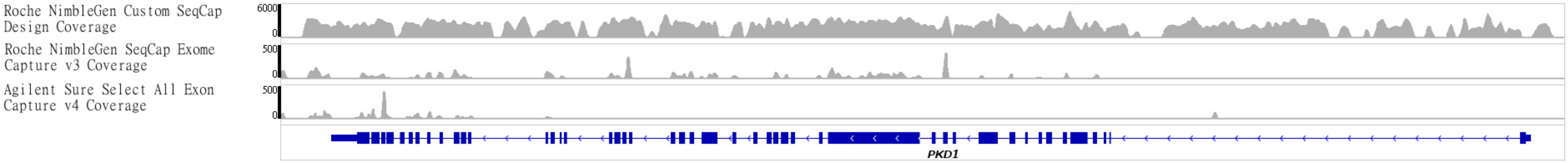

Figure S7. Coverage achieved by our sequence capture approach compared with available exome capture data.

Higher and more complete coverage is achieved by the described gene panel based sequence capture setup in comparison to data available for established exome capture kits which do not completely address these regions in probe design.
