# Supplementary material for: An Efficient and Comprehensive Strategy for Genetic Diagnostics of Polycystic Kidney Disease
Source: PLoS One. 2015 Feb 3;10(2):e0116680. doi: 10.1371/journal.pone.0116680 (PMC4315576; doi:10.1371/journal.pone.0116680)
Supplement: S8 Fig — (PDF) [file pone.0116680.s009.pdf]

Figure S8

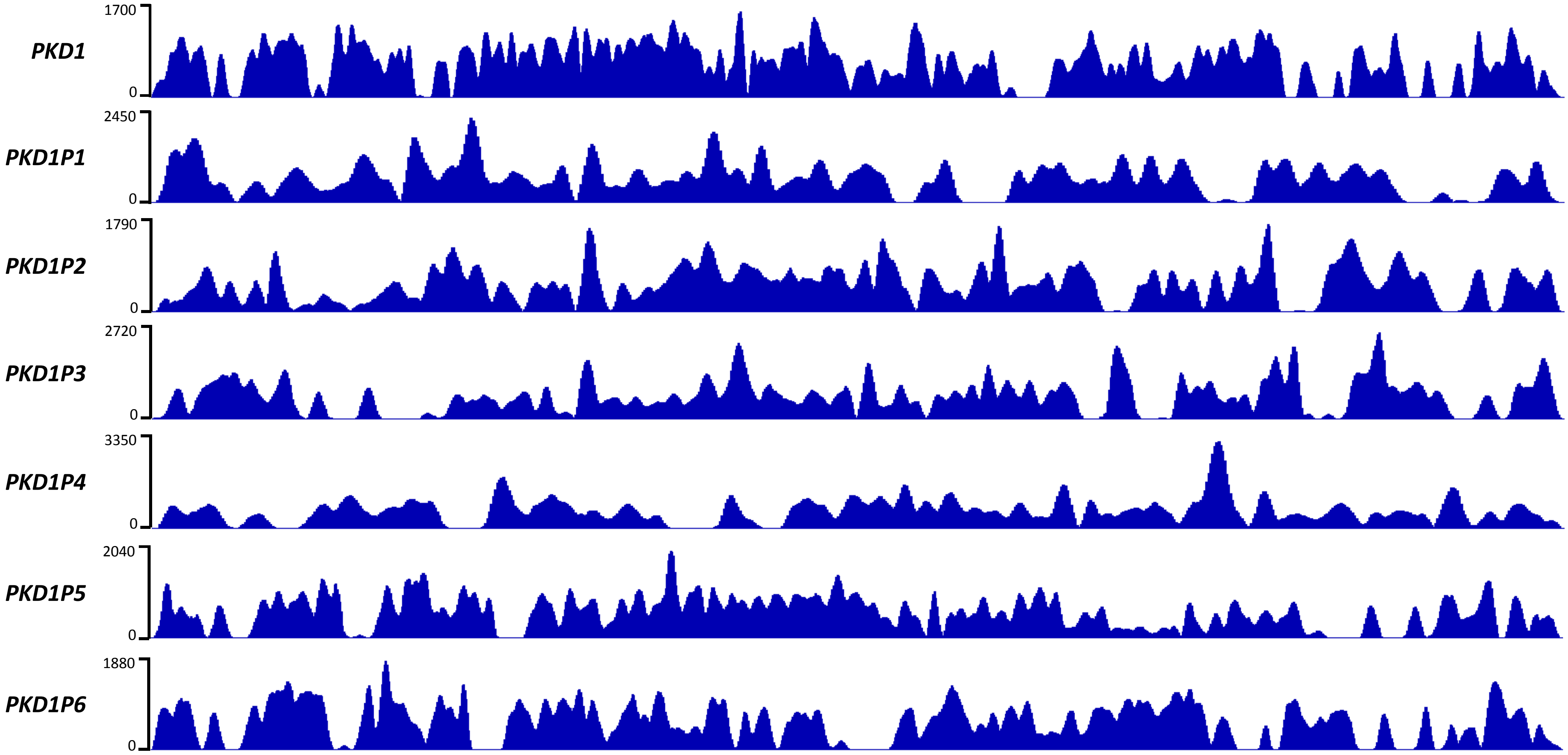

Figure S8. Specific alignment of reads from the genuine *PKD1* gene and duplicated regions to their correct locations.

Example coverage plots for one patient sequenced on the MiSeq platform display equally covered regions in the duplicated *PKD1* region and the pseudogene locations *PKD1P1-P6* (according to GeneCards annotation).
