# Supplementary material for: An Efficient and Comprehensive Strategy for Genetic Diagnostics of Polycystic Kidney Disease
Source: PLoS One. 2015 Feb 3;10(2):e0116680. doi: 10.1371/journal.pone.0116680 (PMC4315576; doi:10.1371/journal.pone.0116680)
Supplement: S9 Fig — (PDF) [file pone.0116680.s010.pdf]

Figure S9

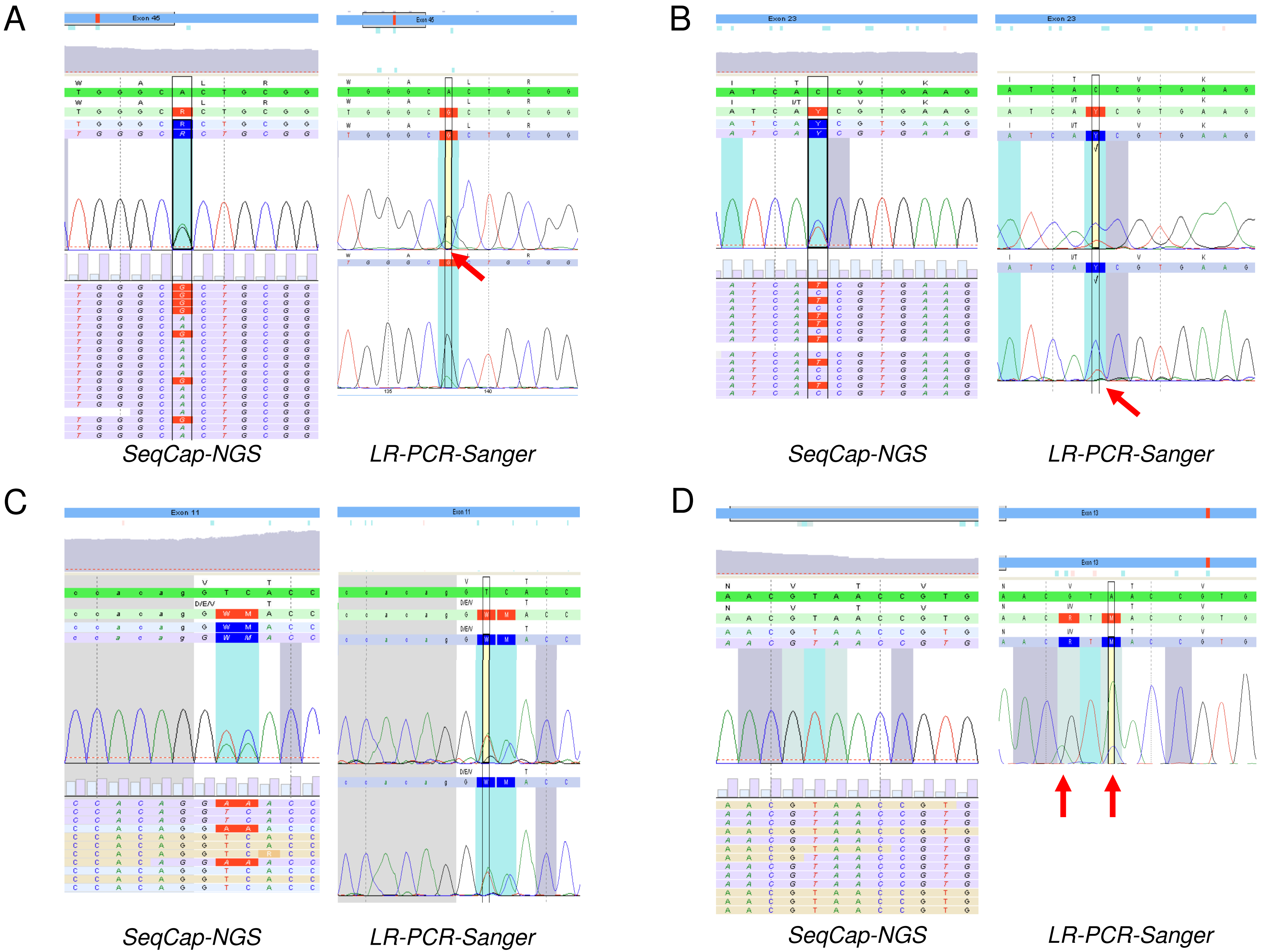

A
B
C
D
SeqCap-NGS
LR-PCR-Sanger
SeqCap-NGS
LR-PCR-Sanger
SeqCap-NGS
LR-PCR-Sanger
SeqCap-NGS
LR-PCR-Sanger

**Figure S9. Examples for superior performance of our NGS based approach versus LR-PCR-Sanger sequencing.**

**A** The nucleotide exchange c.12276A>G (p.Ala4092Ala) in exon 45 in the unique *PKD1* region was clearly detected in the NGS setup with equal read count as seen in the pseudo-electropherogram displayed in the JSI SeqNext software. In contrast, a minor signal (red arrow) for the reference allele in the Sanger electropherogram led to a homozygous alternative call at this site. This effect might have been caused by unequal amplification of reference and alternative allele in the LR-PCR setting.

**B** Calling of the variant c.8618C>T (p.Thr2873Ile) in exon 23 was not unambiguous in Sanger due to a minor signal (red arrow) in the electropherogram. Again this variant was clearly detected in the NGS data.

**C** The 2 bp-substitution c.2099_2100delinsAA (p.Val700Glu) in exon 11 could not be resolved in the Sanger electropherogram. As both nucleotide exchanges lie on the same read, the allelic status of the variant at this position becomes only obvious in the NGS data.

**D** A minor signal at two positions (c.3031G>A and c.3033A>C, red arrows) resulted in erroneous variant calling at these sites in the Sanger electropherogram, but not in the NGS data. The Sanger signal might have resulted from spurious amplification of pseudogene location as the mutated sequence with both alterations unambiguously maps to five duplicated regions, whereas only the wildtype sequence maps to the genuine gene.
