# Supplementary material for: An Efficient and Comprehensive Strategy for Genetic Diagnostics of Polycystic Kidney Disease
Source: PLoS One. 2015 Feb 3;10(2):e0116680. doi: 10.1371/journal.pone.0116680 (PMC4315576; doi:10.1371/journal.pone.0116680)
Supplement: S1 Table — (PDF) [file pone.0116680.s011.pdf]

Table S1. List of all genes targeted by the NimbleGen SeqCap EZ choice library.

**Gene**
ACE
AGT
AGTR1
ANKS6
BBS1
BBS2
BBS7
BBS10
BBS12
BICC1
CEP164
CEP290
CYS1
FAN1
GLIS2
GLIS3
HNF1B
INVS
IQCB1
KIF12
MKKS
MRE11A
MUC1
NEK8
NPHP1
NPHP3
NPHP4
PKD1
PKD2
PKHD1
REN
RPGRIP1L
SDCCAG8
SLC41A1
TMEM67
TTC21B
UMOD
WDR19
XPNPEP3
ZNF423
