# Supplementary material for: An Efficient and Comprehensive Strategy for Genetic Diagnostics of Polycystic Kidney Disease
Source: PLoS One. 2015 Feb 3;10(2):e0116680. doi: 10.1371/journal.pone.0116680 (PMC4315576; doi:10.1371/journal.pone.0116680)
Supplement: S2 Table — (PDF) [file pone.0116680.s012.pdf]

Table S2. Coverage statistics of all target regions and the *PKD1* locus.

Bioinformatic calculation of the coverage statistics has been performed by GATK. The average coverage of all target regions and detailed coverage statistics of the genomic and coding regions in the *PKD1* locus as well as of exon 1 have been calculated. The mean coverage of all pseudogenes (PG) is also listed. For a diagnostic evaluation of the data we require the regions of interest to be covered at least 20x which is achieved in 97% of all target regions and for up to 99% of the *PKD1* coding sequence (not fulfilled only for about half of exon 1). The small box (below left) displays run parameters for both NGS platforms used as well as mapping metrics.

		**All regions coverage**					***PKD1* coverage genomic region**					***PKD1* coverage exonic**					***PKD1* coverage exon 1**					**Mean PG coverage**					
Sample	Instrument	Mean target coverage	% bases >5x	% bases >10x	% bases >15x	% bases >20x	Mean Coverage	% bases >5x	% bases >10x	% bases >15x	% bases >20x	Mean Coverage	% bases >5x	% bases >10x	% bases >15x	% bases >20x	Mean Coverage	% bases >5x	% bases >10x	% bases >15x	% bases >20x	*PKD1P1*	*PKD1P2*	*PKD1P3*	*PKD1P4*	*PKD1P5*	*PKD1P6*
1	MiSeq	753	97.4	97	96.5	96.1	1219	93.5	92.8	92.1	91.5	1708	98.9	98.9	98.8	98.8	20	40	34.9	30.9	29.5	805.7	1819.3	1318.8	1384.5	805.9	1053.2
2	MiSeq	527	97.6	97.2	96.8	96.4	688	93.6	92.8	92.2	91.7	866	99	98.7	98.6	98.6	13	42.5	29.8	26.2	25.1	331.6	718.8	673.5	674.0	411.5	588.7
3	MiSeq	534	97.8	97.5	97.2	96.9	654	94	93.3	92.8	92.1	807	98.7	98.6	98.5	98.3	4	26.5	21.8	16.4	5.8	389.1	860.9	908.5	778.0	492.6	584.6
4	MiSeq	705	97.7	97.3	97	96.7	877	93.6	92.7	92.1	91.6	1126	98.8	98.8	98.7	98.7	19	33.5	30.9	29.5	28.7	463.2	955.6	1047.0	764.4	464.5	797.0
5	MiSeq	542	97.6	97.2	96.9	96.5	794	93.5	92.7	92.1	91.4	1045	98.8	98.7	98.5	98.4	6	30.5	24.7	18.9	10.9	441.7	820.8	960.5	833.2	501.4	697.9
6	MiSeq	526	97.4	97	96.6	96.3	684	93.4	92.5	91.8	91.3	877	99	98.9	98.7	98.7	17	41.1	39.3	31.3	30.2	355.0	580.6	702.0	681.0	427.2	585.7
7	MiSeq	806	97.9	97.7	97.5	97.3	1029	94.2	93.7	93.3	93	1271	99	98.8	98.7	98.7	11	42.9	30.5	26.2	25.1	628.6	1388.9	1414.7	1176.3	691.2	927.8
8	MiSeq	825	97.6	97.2	96.9	96.5	1274	93.5	92.9	92.3	91.8	1758	98.8	98.8	98.8	98.7	22	32	31.6	30.5	30.2	840.7	1999.9	1577.3	1588.7	866.2	1115.1
9	MiSeq	555	97.5	97	96.6	96.2	802	93.3	92.6	91.7	91.2	1079	98.7	98.6	98	97.9	7	28.4	25.1	22.9	20.7	472.8	1153.7	1007.5	922.4	626.8	708.7
10	MiSeq	724	97.8	97.5	97.3	97	1114	94	93.6	93	92.7	1440	98.8	98.8	98.8	98.7	18	33.1	30.2	29.1	28.4	647.5	1216.7	1302.3	1122.2	632.8	939.8
**Mean MiSeq**	**MiSeq**	**650**	**97.6**	**97.3**	**96.9**	**96.6**	**914**	**93.7**	**93.0**	**92.3**	**91.8**	**1198**	**98.9**	**98.8**	**98.6**	**98.6**	**14**	**35.1**	**29.9**	**26.2**	**23.5**	**537.6**	**1151.5**	**1091.2**	**992.4**	**592.0**	**799.8**
11	HiSeq	1786	98	97.7	97.5	97.4	2356	94.1	93.6	93.3	93	2972	99.3	99.3	99.2	99.2	389	58.9	57.5	56.7	56	1825.7	1825.7	3571.9	3258.1	1954.1	2217.1
12	HiSeq	1109	98.1	97.8	97.6	97.4	1418	94.1	93.7	93.3	93	1797	99.2	99.1	99	99	159	52.7	47.6	46.5	43.6	947.7	947.7	1998.8	2118.3	1341.0	1341.8
13	HiSeq	1554	98	97.7	97.4	97.2	2304	94.2	93.6	93.3	92.9	3091	99.3	99.2	99.2	99.2	273	57.5	55.3	54.9	54.2	1459.7	1459.7	2438.0	2683.7	1662.5	2026.3
14	HiSeq	1773	98	97.7	97.4	97.3	2673	94.1	93.6	93.2	92.9	3616	99.2	99.2	99.2	99.2	314	56.7	56.7	54.9	54.2	1934.2	1934.2	3719.5	3734.6	2329.1	2458.1
15	HiSeq	1206	98.1	97.8	97.5	97.4	1797	94.2	93.7	93.3	93	2431	99.2	99.2	99.1	99.1	184	54.9	53.1	50.9	48.7	1435.0	1435.0	2866.6	2564.9	1504.2	1654.4
16	HiSeq	1658	98	97.8	97.6	97.3	2249	94	93.5	93.1	92.8	2879	99.3	99.2	99.2	99.2	346	57.5	54.5	53.8	53.5	1528.8	1528.8	3019.5	2686.7	1597.4	2146.6
17	HiSeq	1734	98.1	97.8	97.6	97.4	2574	94.2	93.8	93.5	93.2	3433	99.3	99.3	99.2	99.2	359	58.5	58.2	55.6	54.5	1706.7	4431.7	3271.4	3801.3	2145.0	2483.7
18	HiSeq	1508	98	97.7	97.5	97.3	2259	94.1	93.6	93.3	92.8	3012	99.3	99.2	99.2	99.2	231	57.5	55.6	54.2	53.1	1562.2	2951.0	2623.7	2620.1	1565.4	2038.6
19	HiSeq	1436	97.9	97.5	97.2	97	2279	93.9	93.3	92.8	92.5	3145	99.2	99.2	99.2	99.2	323	56.4	55.3	54.5	54.2	1912.7	3563.2	3255.6	3619.2	2223.7	2168.0
20	HiSeq	1275	98.2	97.9	97.8	97.6	1496	94.3	93.8	93.6	93.3	1849	99.2	99.2	99.2	99.1	168	56.4	55.3	52	51.3	999.3	2015.9	2121.3	1867.5	1195.2	1354.5
21	HiSeq	1123	97.8	97.5	97.2	96.9	1587	93.6	92.9	92.5	92.1	2055	99.3	99.2	99.2	99.1	249	57.5	55.6	54.9	53.5	1290.7	2637.9	2476.5	2445.7	1542.3	1558.7
22	HiSeq	1979	98.3	98.1	98	97.9	2542	94.5	94.3	94.1	93.9	3181	99.3	99.3	99.2	99.2	347	60.7	57.5	56.7	56.4	1873.3	4188.4	3482.0	3516.8	2229.4	2416.3
23	HiSeq	1220	98	97.7	97.5	97.3	1622	93.7	93.3	93	92.8	2068	99	99	99	98.9	111	43.6	42.2	41.5	40.7	1052.9	2088.3	1840.4	1643.5	967.1	1549.9
24	HiSeq	1415	98	97.7	97.4	97.2	1985	94	93.5	92.9	92.5	2625	99.2	99.2	99.2	99.1	220	56.4	54.9	54.2	53.1	1310.2	3184.7	2428.1	2738.6	1894.1	1857.8
25	HiSeq	1506	98.1	97.8	97.6	97.5	2130	94.1	93.7	93.4	93.1	2740	99.2	99.2	99.1	99	157	54.9	54.5	50.5	44.7	1423.6	4367.9	2387.9	2955.5	1657.4	1928.2
26	HiSeq	1569	98.1	97.8	97.6	97.4	2289	94	93.7	93.4	93.1	2987	99.2	99.2	99.2	99.2	235	56.7	55.3	54.9	54.2	1628.8	3368.6	2953.3	2916.8	1712.0	2044.9
27	HiSeq	1394	98	97.8	97.6	97.4	1984	94.1	93.7	93.4	93.1	2569	99.2	99.2	99.2	99.2	176	56.4	54.9	53.1	52	1321.0	2957.4	2290.6	2338.2	1387.8	1880.3
28	HiSeq	1533	98.1	97.8	97.6	97.4	2251	94.2	93.7	93.4	93.2	2963	99.2	99.2	99.2	99.2	255	55.3	54.5	52.4	52	1656.0	2904.6	3477.3	3057.2	1892.6	2081.5
29	HiSeq	1406	98	97.6	97.4	97.2	2026	93.9	93.4	92.9	92.6	2731	99.3	99.2	99.2	99.2	229	57.5	56	55.6	53.5	1738.2	3373.3	2917.8	2877.0	1658.6	1862.4
30	HiSeq	1360	98.1	97.8	97.6	97.5	1707	94	93.5	93.2	93	2122	99.2	99.2	99.1	99.1	167	54.5	51.6	51.3	50.9	1425.9	3537.6	2468.4	2661.3	1520.4	1631.1
31	HiSeq	1667	98	97.7	97.5	97.3	2448	94.1	93.7	93.4	93.2	3249	99.2	99.2	99.2	99.2	222	56.7	55.3	53.5	52.7	1671.4	2510.7	3212.3	2738.3	1777.8	2198.3
32	HiSeq	712	98	97.7	97.5	97.4	836	93.8	93.3	92.8	92.4	1045	99.1	99	98.9	98.9	73	49.5	45.1	43.3	41.5	558.3	1314.7	1172.0	1212.5	721.4	776.7
33	HiSeq	1808	98	97.7	97.5	97.3	2801	94.1	93.7	93.4	92.9	3829	99.2	99.2	99.2	99.2	283	56.7	55.6	54.9	54.2	2035.6	3820.3	3915.7	3814.8	2379.4	2624.0
34	HiSeq	1249	98	97.8	97.5	97.4	1660	94	93.6	93.2	92.9	2140	99.2	99.1	99.1	99.1	182	56.4	52.4	51.6	51.3	1247.7	1247.7	2281.7	2623.2	1757.3	1655.1
35	HiSeq	1305	98.1	97.8	97.5	97.4	1686	94	93.7	93.3	92.9	2138	99.2	99.2	99.2	99.1	157	55.3	52.7	52	51.3	1139.5	1139.5	2164.2	2012.5	1232.2	1534.6
36	HiSeq	1491	98.1	97.9	97.6	97.4	2021	94.1	93.7	93.4	93.1	2590	99.2	99.2	99.2	99.1	214	54.9	53.1	51.6	51.3	1394.3	1394.3	2444.1	2514.3	1658.9	1886.0
37	HiSeq	1351	98.1	97.9	97.6	97.5	1769	94.1	93.7	93.4	93	2210	99.2	99.2	99.1	99	179	53.1	51.6	47.6	46.5	1304.3	2373.9	2656.2	2213.3	1302.9	1667.7
38	HiSeq	1693	98.1	97.8	97.5	97.4	2383	94.1	93.6	93.3	93	3126	99.2	99.2	99.2	99.2	303	56.4	54.9	53.5	52.4	1782.3	3462.2	3518.4	3301.1	2253.0	2209.1
39	HiSeq	1465	98.1	97.8	97.7	97.5	1892	94.1	93.7	93.4	93.2	2370	99.2	99.2	99.2	99.2	211	54.2	53.1	52.4	52	1489.2	2788.7	2822.6	2488.4	1541.1	1815.3
40	HiSeq	1619	98.1	97.8	97.6	97.4	2195	94.1	93.7	93.3	93	2794	99.2	99.2	99.2	99.2	292	56	54.5	53.8	52.4	1376.2	3544.5	3269.1	2838.7	1715.1	2077.1
41	HiSeq	1269	98.1	97.8	97.5	97.3	1767	94.1	93.6	93.2	92.9	2268	99.2	99.2	99.2	99.2	201	55.3	54.2	53.5	52.7	1297.5	3349.2	2214.8	2486.2	1489.7	1653.6
42	HiSeq	1231	98	97.8	97.6	97.4	1503	94	93.5	93.1	92.8	1862	99.2	99.2	99.2	99.1	206	55.3	53.1	53.1	52	1122.4	2358.6	2105.3	1934.1	1175.9	1458.7
43	HiSeq	1456	98	97.7	97.5	97.3	1969	94	93.3	92.9	92.6	2554	99.2	99.2	99.2	99.2	222	56.4	54.2	53.1	52	1262.8	2193.8	2126.1	2037.1	1386.7	1741.9
44	HiSeq	991	98.1	97.8	97.6	97.4	1253	94.1	93.6	93.2	92.8	1608	99.2	99.2	99.1	99.1	133	55.3	54.9	53.1	52.7	973.2	1859.4	2047.9	1756.9	1082.8	1141.7
45	HiSeq	876	97.9	97.7	97.5	97.3	1034	93.8	93.4	92.8	92.4	1288	99	98.9	98.9	98.6	30	45.1	40.7	39.6	32.4	678.3	1340.1	1543.3	1252.9	741.9	937.2
46	HiSeq	1399	98	97.8	97.6	97.4	1633	94	93.5	93.2	92.9	1854	99.1	99	98.9	98.9	56	50.2	45.5	41.5	38.5	1189.9	2795.5	2429.4	2197.4	1250.3	1668.2
47	HiSeq	1426	98	97.8	97.6	97.5	2004	94.1	93.8	93.4	93.2	2600	99.2	99.1	99	99	137	54.2	50.9	44	42.5	1396.1	2370.8	2882.3	2297.5	1366.3	1846.7
48	HiSeq	1631	98	97.8	97.6	97.5	2172	94.2	93.8	93.4	93	2751	99.3	99.2	99.2	99.1	191	57.5	55.3	52.4	51.3	1490.7	3646.0	2623.2	2766.7	1830.3	2029.5
49	HiSeq	1963	98.1	97.9	97.6	97.5	2918	94	93.7	93.4	93.1	3818	99.2	99.2	99.2	99.1	265	56	54.9	53.8	53.1	1927.9	4370.5	3933.1	3929.8	2962.1	2618.9
50	HiSeq	1571	98	97.8	97.5	97.4	2292	94.1	93.7	93.4	93.1	3069	99.2	99.2	99.2	99.1	210	56.7	53.8	53.1	51.6	1563.0	2424.5	3193.5	2917.0	1759.6	2046.3
51	HiSeq	1568	98.1	97.9	97.6	97.4	2272	94.1	93.7	93.3	93	3020	99.3	99.2	99.2	99.2	261	57.5	55.6	53.8	53.1	1865.2	4917.4	3369.3	3606.2	2133.6	2215.8
52	HiSeq	1680	98.2	98	97.7	97.5	2373	94.2	93.9	93.6	93.4	3048	99.2	99.2	99.2	99.1	246	57.1	54.5	52.4	52	1699.2	3880.4	3370.4	3106.4	1645.7	2157.4
53	HiSeq	680	98	97.6	97.4	97.2	717	93.9	93.3	92.8	92.4	850	99.3	99.2	99.1	99	94	57.5	54.2	49.8	45.8	479.8	1191.3	949.9	946.0	545.0	665.1
54	HiSeq	1478	98.2	98	97.8	97.6	1810	94.3	93.9	93.6	93.4	2228	99.3	99.2	99.2	99.2	242	57.5	56	55.3	54.5	1262.1	2639.3	2504.4	2440.7	1572.6	1682.0
55	HiSeq	1831	98.1	97.8	97.6	97.4	2766	94.1	93.7	93.5	93.2	3691	99.2	99.2	99.2	99.2	299	56.4	54.9	54.2	53.1	1879.8	3725.5	3903.7	2917.2	1758.8	2466.0
**Mean HiSeq**	**HiSeq**	**1443**	**98.1**	**97.8**	**97.6**	**97.4**	**1993**	**94.1**	**93.6**	**93.3**	**92.9**	**2584**	**99.2**	**99.2**	**99.2**	**99.1**	**218**	**55.5**	**53.5**	**51.9**	**50.6**	**1424.9**	**2696.9**	**2716.9**	**2632.3**	**1622.7**	**1855.0**

**Run parameter:**	
**MiSeq**	10 samples
PE Reads	44.40 M
PF PE Reads	39.36 M
% aligned PF Reads (average)	98.90%
**HiSeq**	22 or 23 samples/lane
Lane 1	
PE Reads	420.92 M
PF PE Reads	367.28 M
% aligned PF Reads (average)	98.60%
Lane 2	
PE Reads	413.06 M
PF PE Reads	362.58 M
% aligned PF Reads (average)	98.70%
