# Supplementary material for: An Efficient and Comprehensive Strategy for Genetic Diagnostics of Polycystic Kidney Disease
Source: PLoS One. 2015 Feb 3;10(2):e0116680. doi: 10.1371/journal.pone.0116680 (PMC4315576; doi:10.1371/journal.pone.0116680)
Supplement: S3 Table — (PDF) [file pone.0116680.s013.pdf]

Table S3. Coverage statistics of all target genes sequenced and analysed in parallel to *PKD1* .
The table displays average coverage (Av.Cov.) as well as percentage of the coding regions, for which at least a 15x or 20x coverage was achieved as a prerequisite for diagnostic evaluation of the data. Statistics are listed for all 39 genes sequenced and analysed in parallel to *PKD1* . Bioinformatic calculation of the coverage statistics has been performed by GATK. Under comments critical regions in distinct genes (transcripts) are listed that are in general not properly covered.

	NPHP4			*MUC1*			*REN*			*SLC41A1*			*AGT*		
Patient	Av. Cov.	% > 15x	% > 20x	Av. Cov.	% > 15x	% > 20x	Av. Cov.	% > 15x	% > 20x	Av. Cov.	% > 15x	% > 20x	Av. Cov.	% > 15x	% > 20x
1	1610.85	100	100	1245.52	100	100	1327.64	100	100	1579.15	100	100	1634.08	100	100
2	909.68	100	100	782.49	100	100	833.74	100	100	943.04	100	100	949.06	100	100
3	885.93	100	100	767.73	100	100	804.76	100	100	884.52	100	100	915.12	100	100
4	1255.05	100	100	1021.42	100	100	1131.61	100	100	1247.91	100	100	1311.56	100	100
5	1108.35	100	100	866.14	100	100	962.62	100	100	1084.85	100	100	1192.2	100	100
6	959.32	100	100	761.54	100	100	837.36	100	100	961.16	100	100	984.25	100	100
7	1373.81	100	100	1220.06	100	100	1271.12	100	100	1404.43	100	100	1424.24	100	100
8	1683.86	100	100	1329.98	100	100	1474.02	100	100	1644.51	100	100	1771.9	100	100
9	1098.99	100	100	850.79	100	100	952.84	100	100	1098.79	100	100	1174.63	100	100
10	1380.84	100	100	1159.72	100	100	1216.36	100	100	1359.3	100	100	1452.63	100	100
11	2967.54	100	100	2688.41	100	100	2740.01	100	100	3017.93	100	100	3154.28	100	100
12	1894.25	100	100	1496.41	100	100	1645.69	100	100	1838.74	100	100	2007.36	100	100
13	2982.41	100	100	2439.44	100	100	2588.66	100	100	2964.29	100	100	3205.98	100	100
14	3448.35	100	100	2759.79	100	100	3022.65	100	100	3386.42	100	100	3606.91	100	100
15	2219.55	100	100	1762.96	100	100	1938.94	100	100	2168.84	100	100	2325.49	100	100
16	3015.71	100	100	2424.13	100	100	2642.7	100	100	2872.32	100	100	3145.85	100	100
17	3247.05	100	100	2600.86	100	100	2902.69	100	100	3250.89	100	100	3530.92	100	100
18	2888.31	100	100	2252.52	100	100	2545.49	100	100	2876	100	100	3127.99	100	100
19	2887.15	100	100	2232.6	100	100	2436.08	100	100	2827.83	100	100	3072.21	100	100
20	1970.43	100	100	1817.86	100	100	1811.44	100	100	2012.72	100	100	2060.96	100	100
21	2019.81	100	100	1665.05	100	100	1815.66	100	100	2036.43	100	100	2096.91	100	100
22	3260.79	100	100	2898.39	100	100	2941.6	100	100	3216.37	100	100	3354.97	100	100
23	2174.87	100	100	1798.28	100	100	1975.32	100	100	2199.01	100	100	2317.92	100	100
24	2556.83	100	100	2075.59	100	100	2254.81	100	100	2578.66	100	100	2774.72	100	100
25	2726.81	100	100	2243.21	100	100	2445.8	100	100	2747.08	100	100	2892.28	100	100
26	2922.57	100	100	2377.6	100	100	2615.52	100	100	2921.85	100	100	3158.62	100	100
27	2588.02	100	100	2119.92	100	100	2293.74	100	100	2618.32	100	100	2791.47	100	100
28	2902.78	100	100	2314.17	100	100	2544.05	100	100	2823.4	100	100	3026.56	100	100
29	2706.92	100	100	2096.83	100	100	2324.28	100	100	2657.22	100	100	2873.93	100	100
30	2269.43	100	100	1815.75	100	100	2036.07	100	100	2258.94	100	100	2388.26	100	100
31	3130.54	100	100	2576.87	100	100	2806.29	100	100	3137.38	100	100	3350.45	100	100
32	1076.6	100	100	882.49	100	100	988.05	100	100	1049.83	100	100	1147.53	100	100
33	3588.22	100	100	2871.93	100	100	3101.85	100	100	3569.93	100	100	3870.85	100	100
34	2164.83	100	100	1773	100	100	1987.76	100	100	2172.75	100	100	2332.44	100	100
35	2175.47	100	100	1811.17	100	100	2001.8	100	100	2145.75	100	100	2316.03	100	100
36	2551.21	100	100	2105.23	100	100	2361.75	100	100	2528.92	100	100	2728.62	100	100
37	2274.16	100	100	1879.76	100	100	2071.83	100	100	2289.13	100	100	2445.86	100	100
38	3108.72	100	100	2457.7	100	100	2782.09	100	100	3054.58	100	100	3342.58	100	100
39	2473.75	100	100	2053.05	100	100	2229.7	100	100	2430.14	100	100	2596.77	100	100
40	2797.46	100	100	2277.63	100	100	2563.28	100	100	2778.91	100	100	2998.42	100	100
41	2269.65	100	100	1816.3	100	100	2033.42	100	100	2269.22	100	100	2451.87	100	100
42	2066.02	100	100	1695.12	100	100	1853.46	100	100	2045.25	100	100	2199.25	100	100
43	2597.75	100	100	2112.77	100	100	2314.08	100	100	2612.82	100	100	2761.64	100	100
44	1654.86	100	100	1342.01	100	100	1432.87	100	100	1638.69	100	100	1759.68	100	100
45	1345.61	100	100	1122.18	100	100	1221.75	100	100	1366.57	100	100	1458.24	100	100
46	2321.61	100	100	1931.96	100	100	2126.63	100	100	2310.58	100	100	2478.76	100	100
47	2658.28	100	100	2112.33	100	100	2364.51	100	100	2662.91	100	100	2866.66	100	100
48	2914.62	100	100	2235.9	100	100	2563.56	100	100	2888.34	100	100	3108.76	100	100
49	3697.4	100	100	3001.92	100	100	3290.45	100	100	3665.99	100	100	3985.92	100	100
50	2972.39	100	100	2387	100	100	2549.47	100	100	2974.1	100	100	3169.77	100	100
51	2991.3	100	100	2345.63	100	100	2641.38	100	100	2984.14	100	100	3293.66	100	100
52	3022.37	100	100	2531.09	100	100	2712.24	100	100	3071.31	100	100	3220.76	100	100
53	903.31	100	100	880.07	100	100	874.59	100	100	975.7	100	100	948.94	100	100
54	2264.45	100	100	2009.03	100	100	2118.17	100	100	2317.21	100	100	2378.21	100	100
55	3493.84	100	100	2870.6	100	100	3059.44	100	100	3511.93	100	100	3749.83	100	100
**Max**		**100**	**100**		**100**	**100**		**100**	**100**		**100**	**100**		**100**	**100**
**Mean**		**100**	**100**		**100**	**100**		**100**	**100**		**100**	**100**		**100**	**100**
**Min**		**100**	**100**		**100**	**100**		**100**	**100**		**100**	**100**		**100**	**100**
**Comments**															

	SDCCAG8			*CYS1*			*NPHP1*			*TTC21B*			*IQCB1*			*NPHP3*			*AGTR1*			*WDR19*		
Patient	Av. Cov.	% > 15x	% > 20x	Av. Cov.	% > 15x	% > 20x	Av. Cov.	% > 15x	% > 20x	Av. Cov.	% > 15x	% > 20x	Av. Cov.	% > 15x	% > 20x	Av. Cov.	% > 15x	% > 20x	Av. Cov.	% > 15x	% > 20x	Av. Cov.	% > 15x	% > 20x
1	518.69	100	100	299.88	37.1	37.1	518.11	100	100	289.62	100	100	361.11	100	100	404.51	100	100	593.37	100	100	494.06	99.9	99.9
2	468.79	100	100	238.04	37.1	37.1	443.34	100	100	309.39	100	100	349.2	100	100	369.86	100	100	466	100	100	424.38	99.9	99.5
3	505.49	100	100	239.99	37.1	37.1	253.74	100	100	372.09	100	100	385.99	100	100	433.7	100	100	524.18	100	100	444.52	100	100
4	634.87	100	100	303.16	37.1	37.1	628.72	100	100	435.53	100	100	470.37	100	100	511.87	100	100	684.39	100	100	586.43	100	100
5	403.6	100	100	258.64	37.1	37.1	413.03	100	100	241.97	100	100	290.6	100	100	317.8	100	100	465.51	100	100	366.95	100	99.9
6	463.42	100	100	235.23	37.1	37.1	456.5	100	100	322.7	100	100	349.9	100	100	383.3	100	100	482.43	100	100	432.41	100	99.9
7	732.14	100	100	366.32	37.1	37.1	702.99	100	100	513.13	100	100	553.28	100	100	613.58	100	100	738.17	100	100	657.63	100	100
8	631.43	100	100	343.75	37.1	37.1	612.06	100	100	373.61	100	100	438.58	100	100	487.02	100	100	682.31	100	100	577.16	100	99.9
9	431.91	100	100	272.47	37.1	37.1	437.58	100	100	261.99	100	100	302.46	100	100	338.23	100	100	467.02	100	100	393.58	100	100
10	555.97	100	100	386.08	37.1	37.1	522.85	100	100	323.72	100	100	404.04	100	100	440.67	100	100	555.79	100	100	477.99	100	100
11	1624.92	100	100	790.97	98.5	95.5	1607.08	100	100	1126.93	100	100	1250.77	100	100	1393.64	100	100	1613.23	100	100	1521.92	100	100
12	1000.42	100	100	501.22	63.5	55.3	963.97	100	100	704.11	100	100	746.97	100	100	849.4	100	100	1037.05	100	100	879.52	100	100
13	1224.89	100	100	694.87	66.3	65.2	1197.91	100	100	714.35	100	100	859.14	100	100	981.62	100	100	1247.63	100	100	1081.95	100	100
14	1379.13	100	100	798.22	73.4	66.1	1354.63	100	100	817.88	100	100	998.04	100	100	1126.73	100	100	1509.73	100	100	1235.01	100	100
15	967.82	100	100	512.7	63.9	58.7	959.42	100	100	624.11	100	100	738.85	100	100	816.28	100	100	1039.51	100	100	916.98	100	100
16	1407.32	100	100	770.24	97.2	92.6	1413.48	100	100	957.28	100	100	1049.13	100	100	1215.54	100	100	1502.71	100	100	1275.71	100	100
17	1378.54	100	100	832.36	100	92.4	1348.78	100	100	854.18	100	100	1007.18	100	100	1132.75	100	100	1448.24	100	100	1249.14	100	100
18	1159.8	100	100	705.62	66.5	65.7	1167.72	100	100	695.8	100	100	844.17	100	100	968.85	100	100	1302.98	100	100	1057.56	100	100
19	1086.83	100	100	634.88	67.2	66.1	1102.32	100	100	636.25	100	100	779.18	100	100	875.01	100	100	1198.22	100	100	1018.12	100	100
20	1265.47	100	100	524.26	64.2	62.2	1232.29	100	100	939.44	100	100	996.24	100	100	1106.3	100	100	1262.08	100	100	1141.86	100	100
21	972.4	100	100	516.29	74.1	73.9	984.78	100	100	666.97	100	100	730.72	100	100	834.94	100	100	1005.38	100	100	909.15	100	100
22	1813.31	100	100	891.59	90.5	85.5	1716.83	100	100	1246.27	100	100	1384.64	100	100	1543.61	100	100	1790.77	100	100	1586.13	100	100
23	1044.08	100	100	564.57	45.3	44.5	1011.88	100	100	664.6	100	100	768.12	100	100	856.11	100	100	1074.41	100	100	929.8	100	100
24	1172.38	100	100	656.66	66.7	64.1	1176.61	100	100	760.72	100	100	897	100	100	960.19	100	100	1266.58	100	100	1076.08	100	100
25	1222.11	100	100	696.63	65.5	60.5	1704.79	100	100	744.83	100	100	887.24	100	100	1003.66	100	100	1277.26	100	100	1079.93	100	100
26	1281.44	100	100	700.55	66.7	63.7	1255.74	100	100	791.51	100	100	922.15	100	100	1051.52	100	100	1354.59	100	100	1151.3	100	100
27	1148.93	100	100	637.9	62.6	60.9	1118.97	100	100	692.59	100	100	829.26	100	100	929.4	100	100	1191.73	100	100	1010.44	100	100
28	1246.73	100	100	676.58	68.3	62.8	1242.85	100	100	773.21	100	100	920.27	100	100	1002.77	100	100	1293.28	100	100	1114.29	100	100
29	1138.23	100	100	610.47	68.2	65.9	1122.1	100	100	711.12	100	100	809.25	100	100	924.67	100	100	1225.5	100	100	1036.32	100	100
30	1266.35	100	100	665.08	60.7	49.3	1197.16	100	100	896.18	100	100	972.81	100	100	1078.3	100	100	1288.87	100	100	1133.75	100	100
31	1342.24	100	100	778.14	72.8	71.5	1301.59	100	100	819.9	100	100	972.14	100	100	1108.98	100	100	1406	100	100	1199.55	100	100
32	691.67	100	100	299	61.8	57.4	665.46	100	100	547.7	100	100	550.06	100	100	612.33	100	100	761.05	100	100	639.99	100	100
33	1385.44	100	100	795.82	61.5	60.7	1366.51	100	100	796.33	100	100	962.25	100	100	1108.5	100	100	1527.84	100	100	1227.5	100	100
34	1107.94	100	100	595.29	63.9	63.5	1053.6	100	100	726.56	100	100	833.5	100	100	900.67	100	100	1137.97	100	100	968.98	100	100
35	1207.39	100	100	641.78	66.5	64.6	1127.69	100	100	835.08	100	100	917.87	100	100	968.33	100	100	1225.47	100	100	1057.93	100	100
36	1262.95	100	100	736.85	74.1	73.2	1250.15	100	100	885.3	100	100	976.57	100	100	1073.76	100	100	1352.91	100	100	1172.89	100	100
37	1222.61	100	100	625.23	71.5	64.2	1160.71	100	100	839.78	100	100	913.46	100	100	1033.42	100	100	1257.81	100	100	1079.66	100	100
38	1434.32	100	100	796.23	73.7	71.3	1416	100	100	931.21	100	100	1059.49	100	100	1184.27	100	100	1485.31	100	100	1324.01	100	100
39	1375.32	100	100	699.41	73.4	73	1289.65	100	100	948.56	100	100	1028.71	100	100	1160.12	100	100	1362.96	100	100	1205.41	100	100
40	1389.02	100	100	754.91	68.7	67.2	1372.03	100	100	935.68	100	100	1062.92	100	100	1192.81	100	100	1456.95	100	100	1263.46	100	100
41	1090.89	100	100	576.72	74.7	62.2	1069.13	100	100	724.83	100	100	813.33	100	100	906.2	100	100	1154.03	100	100	974.81	100	100
42	1151.24	100	100	562.07	48	46.7	1117.01	100	100	827.68	100	100	897.4	100	100	979.29	100	100	1178.1	100	100	1026.63	100	100
43	1252.91	100	100	622.01	72.4	71.5	1233.09	100	100	834.8	100	100	926.12	100	100	1049.42	100	100	1300.02	100	100	1147.66	100	100
44	919.73	100	100	408.91	57	48.8	897.6	100	100	685.45	100	100	737.13	100	100	792.77	100	100	973.23	100	100	855.7	100	100
45	847.52	100	100	365.94	37.1	37.1	815.1	100	100	644.46	100	100	663.71	100	100	714.65	100	100	878.2	100	100	778.14	100	100
46	1306.05	100	100	648.38	37.1	37.1	1256.2	100	100	955.74	100	100	1039.99	100	100	1115.84	100	100	1318.96	100	100	1203.16	100	100
47	1170.43	100	100	674.38	40.6	39.5	1146.22	100	100	722.51	100	100	830.94	100	100	942.34	100	100	1228.01	100	100	1006.9	100	99.9
48	1407.08	100	100	767.18	46.6	45.6	1366	100	100	911.12	100	100	1035	100	100	1158.91	100	100	1448.91	100	100	1218.25	100	100
49	1578.13	100	100	974.53	73.7	73.2	1501.2	100	100	935.04	100	100	1157.1	100	100	1270.51	100	100	1617.18	100	100	1380.99	100	100
50	1257.38	100	100	699.43	46.6	45.8	1231.5	100	100	769.86	100	100	900.77	100	100	1017.55	100	100	1345.79	100	100	1109.65	100	100
51	1294.78	100	100	714.19	68.9	68	1245.82	100	100	797.23	100	100	934.67	100	100	1063.45	100	100	1390.31	100	100	1170.51	100	100
52	1444.19	100	100	771.7	82.9	65.4	1380.74	100	100	895.12	100	100	1051.77	100	100	1188.41	100	100	1449.98	100	100	1295.25	100	100
53	722.55	100	100	282.96	42.6	41.3	693.87	100	100	598.03	100	100	622.74	100	100	654.36	100	100	727.1	100	100	691.88	100	100
54	1444.11	100	100	680.91	80.6	63.5	1370.23	100	100	1093.75	100	100	1133.6	100	100	1249.83	100	100	1391.02	100	100	1299.3	100	100
55	1456.38	100	100	838.36	66.9	63.3	1402.51	100	100	877.13	100	100	1030.28	100	100	1163.68	100	100	1503.82	100	100	1285.93	100	100
**Max**		**100**	**100**		**100**	**95.5**		**100**	**100**		**100**	**100**		**100**	**100**		**100**	**100**		**100**	**100**		**100**	**100**
**Mean**		**100**	**100**		**61.1**	**58.0**		**100**	**100**		**100**	**100**		**100**	**100**		**100**	**100**		**100**	**100**		**100.0**	**100.0**
**Min**		**100**	**100**		**37.1**	**37.1**		**100**	**100**		**100**	**100**		**100**	**100**		**100**	**100**		**100**	**100**		**99.9963**	**99.9**
**Comments**				*CYS1* (NM_001037160): exon 1 not complete covered.																				

	PKD2			*BBS7*			*BBS12*			*PKHD1*			*TMEM67*			*GLIS3*		
Patient	Av. Cov.	% > 15x	% > 20x	Av. Cov.	% > 15x	% > 20x	Av. Cov.	% > 15x	% > 20x	Av. Cov.	% > 15x	% > 20x	Av. Cov.	% > 15x	% > 20x	Av. Cov.	% > 15x	% > 20x
1	646.27	97.8	97.6	242.83	99.6	99.5	613.23	100	100	830.51	100	100	293.64	96.9	96.7	1127.85	100	100
2	499.79	97.8	97.5	257.01	99.5	98.9	566.37	100	100	636.35	100	100	257.62	100	99.9	746.07	100	100
3	496.9	97.6	97.4	323.83	100	99.9	572.36	100	100	643.12	100	100	329.61	100	100	766.12	100	100
4	673.06	98	97.7	372.1	100	99.8	748.22	100	100	854.77	100	100	389.92	100	99.8	1035.28	100	100
5	478.02	97.5	97.1	207.86	100	99.9	495.57	100	100	603.48	100	100	245.78	100	99.9	832.08	100	100
6	505.43	97.8	97.7	281.04	99.8	99.6	572.45	100	100	644.84	100	100	283.34	99.9	99.8	780.37	100	100
7	764.64	98.2	98	455.25	100	100	814.67	100	100	952.35	100	100	441.36	100	100	1136.12	100	100
8	717.27	98.1	97.8	308.44	99.5	99.2	751.04	100	100	953.36	100	100	351.16	99.6	99.6	1228.26	100	100
9	495.3	98	97.7	208.23	99.1	99	522.44	100	100	665.73	100	100	258.38	99.4	99.1	884.89	100	100
10	628.98	98	97.9	279.76	99.8	99.6	627.57	100	100	769.11	100	100	311.28	100	99.8	1071.38	100	100
11	1824.54	100	100	1020.9	100	100	1909.52	100	100	2135.09	100	100	1000.17	100	100	2515.38	100	100
12	1103.94	100	100	607.6	100	99.9	1218.35	100	100	1335.22	100	100	587.28	100	100	1611.08	100	100
13	1478.2	100	100	594.22	99.8	99.8	1477.85	100	100	1789.25	100	100	669.41	100	100	2366.24	100	100
14	1630.94	100	100	688.89	100	100	1646.55	100	100	2055.66	100	100	769.16	100	100	2734.63	100	100
15	1149.1	100	100	552.1	99.8	99.7	1229.62	100	100	1391.06	100	100	578.91	99.8	99.8	1713.74	100	100
16	1649.43	100	100	832.34	100	99.9	1725.81	100	100	1991.65	100	100	836.49	100	100	2468.34	100	100
17	1646.75	100	100	727.3	100	99.9	1672.2	100	100	1991.13	100	100	802.54	100	100	2605.84	100	100
18	1405.79	100	99.9	583.94	99.9	99.8	1479.92	100	100	1743.84	100	100	650.29	99.9	99.8	2294.08	100	100
19	1330.59	100	99.8	528.07	100	100	1321.2	100	100	1668.85	100	100	598.82	99.8	99.4	2169.15	100	100
20	1306.49	100	100	846.46	100	100	1434.43	100	100	1549.64	100	100	820.96	100	100	1744.21	100	100
21	1112.96	100	100	578.16	99.9	99.8	1162.01	100	100	1333.52	100	100	590.62	100	99.9	1627.02	100	100
22	1976.3	100	100	1130.86	100	100	2100.15	100	100	2292.84	100	100	1097.28	100	100	2771.8	100	100
23	1200.31	99	98.8	554.11	99.9	99.8	1264.57	100	100	1433.7	100	100	575.97	100	100	1767.56	100	100
24	1371.52	100	100	645.23	100	100	1448.82	100	100	1663.57	100	100	697.55	100	100	2080.35	100	100
25	1417.26	100	100	643.37	99.8	99.7	1485.17	100	100	1733.84	100	100	660.13	100	100	2235.73	100	100
26	1502.93	100	100	656.66	100	99.8	1584.12	100	100	1828.2	100	100	711.76	100	99.9	2355.42	100	100
27	1347.57	99.8	99.4	593.43	100	99.8	1370.87	100	100	1628.82	100	100	612.04	100	99.9	2081.86	100	100
28	1468.51	100	99.7	654.55	100	100	1503.57	100	100	1786.69	100	100	672.7	100	99.9	2294.39	100	100
29	1339.74	100	100	598.9	100	100	1381.13	100	100	1660.44	100	100	676.41	100	100	2098.64	100	100
30	1348.04	100	100	785.53	100	100	1526.2	100	100	1662.65	100	100	753.34	100	100	1946.18	100	100
31	1601.1	100	100	690.35	99.9	99.8	1630.4	100	100	1937.1	100	100	738.14	100	100	2505.63	100	100
32	707.26	99.9	99.6	492.68	100	100	839.32	100	100	847.79	100	100	483.57	100	100	919.03	100	100
33	1676.92	100	100	672.95	100	99.8	1681.49	100	100	2072.31	100	100	759.55	100	99.8	2785.49	100	100
34	1243.57	100	100	638.23	100	99.9	1363.63	100	100	1501.49	100	100	631.32	100	100	1847.78	100	100
35	1311.72	100	100	696.87	100	100	1396.54	100	100	1582	100	100	689.35	100	100	1896.53	100	100
36	1443.75	100	100	764.86	100	99.9	1587.1	100	100	1759.98	100	100	767.7	100	99.9	2159.84	100	100
37	1329.92	100	100	755.01	100	100	1492.88	100	100	1631.64	100	100	717.66	100	100	1966.32	100	100
38	1627.62	100	100	806.9	100	100	1749.75	100	100	2022.8	100	100	844.44	100	100	2569.16	100	100
39	1465.51	100	100	852.18	100	100	1590.43	100	100	1765.37	100	100	817.7	100	100	2125.18	100	100
40	1599.53	100	99.8	818.71	100	100	1736.69	100	100	1911.6	100	100	825	100	100	2413.73	100	100
41	1254.72	100	100	617.5	100	99.8	1341.05	100	100	1499.84	100	100	648.56	100	100	1880.4	100	100
42	1241.93	100	99.7	729.24	100	100	1398.37	100	100	1520.34	100	100	689.46	100	100	1775.54	100	100
43	1408.94	100	100	740.62	100	100	1514.09	100	100	1727.75	100	100	728.8	100	100	2094.23	100	100
44	981.99	100	99.9	623.74	100	100	1071.2	100	100	1187.06	100	100	634.87	100	100	1369.63	100	100
45	855.5	98.4	98.1	571.47	100	100	1053.44	100	100	1055.44	100	100	544.86	100	100	1164.23	100	100
46	1385.94	98.4	98.3	843.51	100	100	1512.65	100	100	1677.23	100	100	803.92	100	100	1932.28	100	100
47	1332.31	99.3	99.1	597.49	99.8	99.6	1467.47	100	100	1681.58	100	100	635.5	100	100	2146.81	100	100
48	1566.6	99.8	99.5	794.47	100	100	1707.6	100	100	1956.6	100	100	797.36	100	100	2415.75	100	100
49	1851.45	100	100	787.08	100	99.9	1898.01	100	100	2292.46	100	100	834.73	100	100	3016.51	100	100
50	1475.41	100	99.6	632.11	100	100	1494.64	100	100	1797.31	100	100	706.95	100	100	2354.85	100	100
51	1490.77	100	100	683.84	100	100	1542.65	100	100	1854.99	100	100	755.88	100	100	2360.72	100	100
52	1677.44	100	100	754.75	99.9	99.7	1793.02	100	100	1999.24	100	100	741.34	100	100	2503.23	100	100
53	718.74	100	100	611.44	100	100	827.66	100	100	795.87	100	100	592.15	100	100	852.52	100	100
54	1487.91	100	100	965.05	100	100	1649.29	100	100	1744.91	100	100	926.95	100	100	2033.76	100	100
55	1740.69	100	100	738.86	100	99.9	1800.48	100	100	2078.11	100	100	816.41	99.9	99.8	2753.27	100	100
**Max**		**100**	**100**		**100**	**100**		**100**	**100**		**100**	**100**		**100**	**100**		**100**	**100**
**Mean**		**99.5**	**99.4**		**99.9**	**99.8**		**100**	**100**		**100**	**100**		**99.9**	**99.9**		**100**	**100**
**Min**		**97.5**	**97.1**		**99.1**	**99**		**100**	**100**		**100**	**100**		**99.4**	**99.1**		**100**	**100**
**Comments**	*PKD2* (NM_000297): exon 1 not complete covered.																	

	ANKS6			*INVS*			*KIF12*			*BICC1*			*BBS1*			*MRE11A*			*CEP164*		
Patient	Av. Cov.	% > 15x	% > 20x	Av. Cov.	% > 15x	% > 20x	Av. Cov.	% > 15x	% > 20x	Av. Cov.	% > 15x	% > 20x	Av. Cov.	% > 15x	% > 20x	Av. Cov.	% > 15x	% > 20x	Av. Cov.	% > 15x	% > 20x
1	1127.2	94.6	94.5	791.15	100	100	1321.41	100	100	605.25	100	100	1187.51	100	100	343.71	100	100	1195.02	100	100
2	714.27	94.3	94.2	617.1	100	100	814.47	100	100	511.38	100	100	799.98	100	100	328.7	100	100	780.65	100	100
3	675.21	94.4	94.3	620.63	100	100	773.66	100	100	536.92	100	100	736.63	100	100	392.74	100	100	749.28	100	100
4	952.48	94.5	94.4	830.86	100	100	1048.7	100	100	688.48	100	100	1023.1	100	100	466.11	100	100	999.88	100	100
5	819.91	94.2	94.1	585.97	100	100	933.54	100	100	463.61	100	100	855.48	100	100	265.81	100	100	859.93	100	100
6	726.94	94.5	94.3	624.04	100	100	806.07	100	100	513.84	100	100	778.12	100	100	347.7	100	100	773.11	100	100
7	1070.1	94.6	94.4	912.63	100	100	1221.78	100	100	755.01	100	100	1158.96	100	100	543.1	100	100	1190.82	100	100
8	1219.6	94.4	94.3	903.29	100	100	1393.89	100	100	707.59	100	100	1258.44	100	100	412.52	100	100	1279.18	100	100
9	812.38	94.2	94.1	644.62	100	100	939.07	100	100	495.86	100	100	806.36	100	100	291.65	100	100	837.8	100	100
10	1066.7	94.4	94.4	761.35	100	100	1240.07	100	100	600.68	100	100	1158.38	100	100	371.12	100	100	1139.32	100	100
11	2328.8	99.2	99.1	2118.42	100	100	4840.35	100	100	1812.64	100	100	2621.4	100	100	1208.26	100	100	2559.54	100	100
12	1512.3	95.1	95.1	1295.68	100	100	1647.79	100	100	1122.15	100	100	1523.36	100	100	751.8	100	100	1548.04	100	100
13	2246.4	95.4	95.4	1723.59	100	100	2539.24	100	100	1385.39	100	100	2396.9	100	100	827.88	100	100	2338.65	100	100
14	2562	95.2	95.2	1975.43	100	100	2939.9	100	100	1597.51	100	100	2657.69	100	100	937.49	100	100	2635.53	100	100
15	1632.6	95.5	95.5	1339.1	100	100	1939.21	100	100	1125.36	100	100	1756.03	100	100	715.65	100	100	1755.58	100	100
16	2333.2	99.3	97.3	1944.27	100	100	2671.02	100	100	1622.36	100	100	2433.23	100	100	1014.64	100	100	2426.87	100	100
17	2459.9	95.6	95.5	1949.01	100	100	2899.6	100	100	1574.08	100	100	2638.38	100	100	952.52	100	100	2593.14	100	100
18	2192.3	95.5	95.5	1704	100	100	2490.79	100	100	1365.58	100	100	2336.13	100	100	804.96	100	100	2262.3	100	100
19	2094.7	95.4	95.3	1607.11	100	100	2438.39	100	100	1283.68	100	100	2255.7	100	100	745.1	100	100	2215.44	100	100
20	1601.6	95.4	95.2	1516.37	100	100	1756.92	100	100	1326.74	100	100	1676.23	100	100	1014.17	100	100	1754.92	100	100
21	1536.5	98	95.1	1310.04	100	100	1783.88	100	100	1105.7	100	100	1701.4	100	100	716.04	100	100	1648.13	100	100
22	2615.6	95.4	95.3	2201.76	100	100	2931.43	100	100	1929.63	100	100	2785.1	100	100	1394.7	100	100	2798.2	100	100
23	1691	95.1	95	1376.32	100	100	1900.42	100	100	1135.11	100	100	1830.76	100	100	738.32	100	100	1823.65	100	100
24	1996.6	95.3	95.3	1643.24	100	100	2212.54	100	100	1348.13	100	100	2068.76	100	100	857.62	100	100	2028.72	100	100
25	2119.9	95	95	1695.76	100	100	2389.41	100	100	1368.57	100	100	2274.48	100	100	863.7	100	100	2236.09	100	100
26	2288.9	97.1	95	1787.75	100	100	2545.23	100	100	1444.79	100	100	2393.25	100	100	904.34	100	100	2356.36	100	100
27	1987.5	95.1	95	1580.85	100	100	2258.1	100	100	1262.12	100	100	2164.64	100	100	788.79	100	100	2123.14	100	100
28	2217.7	95.4	95.3	1749.78	100	100	2499.64	100	100	1385	100	100	2347.01	100	100	870.31	100	100	2360.59	100	100
29	1993.7	95.2	95.1	1591.86	100	100	2237.05	100	100	1285.17	100	100	2043.87	100	100	806.63	100	100	2046.88	100	100
30	1808.8	95	95	1607.55	100	100	2026.96	100	100	1412.04	100	100	1864.61	100	100	966.33	100	100	1882.94	100	100
31	2414.6	95.4	95.3	1886.2	100	100	2739.12	100	100	1536.37	100	100	2584.11	100	100	921.79	100	100	2529.93	100	100
32	889.23	94.9	94.9	823	100	100	996.38	100	100	729.99	100	100	907.19	100	100	577.97	100	100	937.04	100	100
33	2688.8	95.1	95.1	1970.82	100	100	3043.27	100	100	1569.99	100	100	2838.97	100	100	933.26	100	100	2806.57	100	100
34	1723.9	95.3	95.3	1461.56	100	100	1964.96	100	100	1225	100	100	1817.21	100	100	811.63	100	100	1843.65	100	100
35	1733.9	95.1	95	1519.12	100	100	1890.66	100	100	1321.03	100	100	1824.5	100	100	881.57	100	100	1776.49	100	100
36	2021.7	98.3	96.9	1704.85	100	100	2270.36	100	100	1457.96	100	100	2154.56	100	100	956.24	100	100	2088.25	100	100
37	1835.6	95.3	95.2	1557.67	100	100	2050.01	100	100	1349.26	100	100	1889.36	100	100	884.01	100	100	1919.99	100	100
38	2356.7	95.1	95.1	1978.77	100	100	2723.37	100	100	1617.8	100	100	2447	100	100	1021.38	100	100	2478.71	100	100
39	1985.5	98.1	95.1	1722.48	100	100	2227.96	100	100	1479.19	100	100	2074.64	100	100	1018.62	100	100	2093.53	100	100
40	2202.4	95.1	95.1	1884.71	100	100	2516.98	100	100	1584.66	100	100	2359.1	100	100	1024.55	100	100	2329.66	100	100
41	1779.4	95.3	95.2	1460.03	100	100	1967	100	100	1214.59	100	100	1887.23	100	100	775.33	100	100	1853.06	100	100
42	1652.8	95.3	95.2	1473.85	100	100	1816.87	100	100	1272.97	100	100	1692.02	100	100	904.19	100	100	1748.55	100	100
43	1966.6	95.2	95.1	1690.3	100	100	2265.48	100	100	1397.9	100	100	2127.9	100	100	953.79	100	100	2113.23	100	100
44	1276.6	94.9	94.9	1146.56	100	100	1406.45	100	100	981.46	100	100	1322.54	100	100	749.99	100	100	1357.79	100	100
45	1099.6	94.5	94.4	1037.9	100	100	1214.4	100	100	896.95	100	100	1170.54	100	100	673.9	100	100	1167.07	100	100
46	1825.4	94.9	94.8	1661.46	100	100	2086.38	100	100	1390.3	100	100	2036.38	100	100	1006.06	100	100	1996.81	100	100
47	2073.9	95	94.9	1626.21	100	100	2319.29	100	100	1301.47	100	100	2167.09	100	100	818.28	100	100	2178.76	100	100
48	2260.6	95.3	95.2	1875.24	100	100	2525.17	100	100	1550.8	100	100	2367.18	100	100	1021.09	100	100	2383.87	100	100
49	2853.1	95.1	95.1	2227.13	100	100	3276.36	100	100	1795.38	100	100	3036.92	100	100	1058.19	100	100	3017.06	100	100
50	2258	95.4	95.3	1754.54	100	100	2516.25	100	100	1389.12	100	100	2375.54	100	100	878.77	100	100	2354.79	100	100
51	2303.5	95.5	95.4	1782.24	100	100	2542.55	100	100	1437.52	100	100	2385.1	100	100	895.45	100	100	2339.43	100	100
52	2366.1	97.5	95.3	1964.68	100	100	2681.35	100	100	1612.27	100	100	2576.45	100	100	998.5	100	100	2528.54	100	100
53	777.4	95.3	95.2	794.83	100	100	851.2	100	100	751.54	100	100	842.23	100	100	634.83	100	100	869.25	100	100
54	1854.8	95.3	95.3	1720.6	100	100	2047.77	100	100	1511.11	100	100	1951.72	100	100	1139.36	100	100	1940.33	100	100
55	2657.8	96.9	95.4	2048.49	100	100	2998.47	100	100	1607.88	100	100	2868.49	100	100	973.32	100	100	2787.52	100	100
**Max**		**99.3**	**99.1**		**100**	**100**		**100**	**100**		**100**	**100**		**100**	**100**		**100**	**100**		**100**	**100**
**Mean**		**95.5**	**95.1**		**100**	**100**		**100**	**100**		**100**	**100**		**100**	**100**		**100**	**100**		**100**	**100**
**Min**		**94.2**	**94.1**		**100**	**100**		**100**	**100**		**100**	**100**		**100**	**100**		**100**	**100**		**100**	**100**
**Comments**	*ANKS6* (NM_173551): exon 1 not complete covered.																				

	BBS10			*CEP290*			*FAN1*			*GLIS2*			*UMOD*			*ZNF423*		
Patient	Av. Cov.	% > 15x	% > 20x	Av. Cov.	% > 15x	% > 20x	Av. Cov.	% > 15x	% > 20x	Av. Cov.	% > 15x	% > 20x	Av. Cov.	% > 15x	% > 20x	Av. Cov.	% > 15x	% > 20x
1	456.67	100	100	182.52	98.8	97.5	743.36	100	100	1346.59	100	98.3	1794.56	100	100	2147.87	100	100
2	467.95	100	100	190.16	98.9	98.1	592.78	100	100	807.05	100	100	898.2	100	100	1104.03	100	100
3	498.99	100	100	285.09	100	99.9	607.31	100	100	751.8	100	100	871.6	100	100	1057.7	100	100
4	647.31	100	100	301.6	100	99.9	813.27	100	100	1066.33	98.1	97.7	1271.67	100	100	1511.66	100	100
5	387.23	100	100	170.12	99.8	99.5	599.59	100	100	918.69	100	100	1152.83	100	100	1384.14	100	100
6	470.54	100	100	220.4	99.6	98.8	616.35	100	100	776.86	97.9	97.7	941.17	100	100	1132.84	100	100
7	709.78	100	100	384.19	100	99.9	895.49	100	100	1183.15	100	100	1413.62	100	100	1657.71	100	100
8	583.03	100	100	237.75	99.5	99.1	851.75	100	100	1423.31	100	100	1898.15	100	100	2265.91	100	100
9	424.3	100	100	170.59	99.1	97.9	617.25	100	100	919.9	100	99.7	1130.07	100	100	1443.35	100	100
10	533.75	100	100	226.87	99.5	99.3	736.74	100	100	1283.74	100	100	1443.77	100	100	1645.98	100	100
11	1600.51	100	100	791.43	100	100	2016.38	100	100	2595.47	100	100	3078.13	100	100	3430.15	100	100
12	1035.54	100	100	496.06	99.8	99.6	1267.97	100	100	1580.8	100	100	1981.8	100	100	2209.82	100	100
13	1149.32	100	100	459.36	99.7	99.4	1663.19	100	100	2597.5	100	100	3257.08	100	100	3734.92	100	100
14	1296.95	100	100	523.63	99.8	99.7	1937.62	100	100	2942.11	100	100	3759.61	100	100	4297.37	100	100
15	952.06	100	100	440.87	100	99.8	1287.39	100	100	1927.98	100	100	2373.92	100	100	2767.84	100	100
16	1393.84	100	100	645.61	100	99.9	1850.18	100	100	2570.04	100	100	3111.7	100	100	3513.94	100	100
17	1354.3	100	100	568.68	100	100	1878.57	100	100	2840.63	100	100	3438.04	100	100	3973.85	100	100
18	1128.62	100	100	451.05	99.9	99.8	1627.62	100	100	2494.71	100	100	3175.03	100	100	3617.02	100	100
19	1024	100	100	403.36	99.8	99.3	1555.31	100	100	2497.23	100	100	3138.56	100	100	3653.25	100	100
20	1270.81	100	100	742.13	100	100	1495.87	100	100	1640.36	100	100	2052.84	100	100	2276.12	100	100
21	974.37	100	100	445.36	100	100	1259	100	100	1733.32	100	100	2104.35	100	100	2345.89	100	100
22	1826.3	100	100	945.6	100	100	2221.74	100	100	2896.09	100	100	3435.52	100	100	3805.51	100	100
23	1029.66	100	100	446.2	99.8	99.6	1351.54	100	100	1864.55	100	100	2297.13	100	100	2604.97	100	100
24	1206.91	100	100	515.48	99.9	99.9	1605.46	100	100	2198.18	100	100	2785.14	100	100	3123.38	100	100
25	1187.01	100	100	488.92	99.8	99.6	1642.56	100	100	2341.44	100	100	2899.77	100	100	3283.71	100	100
26	1241.33	100	100	506.13	100	100	1712.9	100	100	2532.7	100	100	3117.99	100	100	3583.91	100	100
27	1118.62	100	100	461.17	99.9	99.6	1526.17	100	100	2278.34	100	100	2760.55	100	100	3159.68	100	100
28	1194.9	100	100	489.35	100	99.8	1651.31	100	100	2517.01	100	100	3066.66	100	100	3529.35	100	100
29	1101.37	100	100	476.64	100	99.9	1544.57	100	100	2238.98	100	100	2986.55	100	100	3421.76	100	100
30	1318.74	100	100	649.58	100	99.9	1600.16	100	100	1919.24	100	100	2276.66	100	100	2562.95	100	100
31	1307.29	100	100	531.96	99.9	99.6	1816.57	100	100	2770.2	100	100	3345.72	100	100	3836.81	100	100
32	701.35	100	100	429.97	100	100	817.39	100	100	902.55	100	100	1163.72	100	100	1273.75	100	100
33	1286.46	100	100	502.96	99.9	99.7	1921.97	100	100	3103.16	100	100	4067.2	100	100	4612.55	100	100
34	1112.74	100	100	514.22	100	99.9	1420.7	100	100	1862.51	100	100	2285.7	100	100	2562.83	100	100
35	1215.14	100	100	586.03	99.9	99.8	1542.02	100	100	1885.25	100	100	3138.8	100	100	2534.23	100	100
36	1342.91	100	100	606.24	100	100	1662.94	100	100	2257.14	100	100	2651.09	100	100	3052.47	100	100
37	1253.53	100	100	619.44	100	100	1541.67	100	100	1978.93	100	100	2397.83	100	100	2646.6	100	100
38	1448.27	100	100	640.96	100	99.9	1934.4	100	100	2654.34	100	100	3225.52	100	100	3772.82	100	100
39	1378.78	100	100	677.28	100	100	1691.7	100	100	2150.34	100	100	2549.99	100	100	2822.13	100	100
40	1416.3	100	100	639.93	100	99.8	1833.94	100	100	2460.51	100	100	2926.96	100	100	3294.44	100	100
41	1097.96	100	100	503.29	100	100	1440.21	100	100	1980.17	100	100	2391.35	100	100	2721.29	100	100
42	1207.82	100	100	592.56	100	100	1445.38	100	100	1739.74	100	100	2101.39	100	100	2358.07	100	100
43	1267.49	100	100	573.47	100	99.9	1638.7	100	100	2249.49	100	100	2718.65	100	100	3095.06	100	100
44	935.88	100	100	549.55	100	100	1140.9	100	100	1402.23	100	100	1843.49	100	100	2059.66	100	100
45	927.79	100	100	489.55	100	100	1033.66	100	100	1133.26	100	100	1460.69	100	100	1576.29	100	100
46	1357.05	100	100	699.64	100	100	1617.94	100	100	2023.4	100	100	2388.28	100	100	2655.39	100	100
47	1149.86	100	100	473.16	100	99.9	1555.91	100	100	2264.36	100	100	2794.51	100	100	3235.91	100	100
48	1384.06	100	100	627.24	100	100	1832.25	100	100	2468.34	100	100	2992.01	100	100	3440.94	100	100
49	1571.36	100	100	604.98	100	99.7	2150.76	100	100	3241.85	100	100	3871.86	100	100	4462.24	100	100
50	1214	100	100	500.57	100	100	1681.89	100	100	2552.46	100	100	3267.04	100	100	3698.34	100	100
51	1263.99	100	100	541.91	100	100	1740.23	100	100	2611	100	100	3343.31	100	100	3805.03	100	100
52	1387.71	100	100	523.92	99.9	99.7	1883.04	100	100	2671.66	100	100	3172.39	100	100	3607.93	100	100
53	778.05	100	100	531.36	100	100	776.73	100	100	806.92	100	100	926.99	100	100	990.21	100	100
54	1508.83	100	100	823.99	100	100	1728.57	100	100	1974.52	100	100	2283.88	100	100	2574.54	100	100
55	1388.81	100	100	563.78	100	100	1946.79	100	100	3118.81	100	100	3807.6	100	100	4413.92	100	100
**Max**		**100**	**100**		**100**	**100**		**100**	**100**		**100**	**100**		**100**	**100**		**100**	**100**
**Mean**		**100**	**100**		**99.9**	**99.7**		**100**	**100**		**99.9**	**99.9**		**100**	**100**		**100**	**100**
**Min**		**100**	**100**		**99.1**	**97.9**		**100**	**100**		**97.9**	**97.7**		**100**	**100**		**100**	**100**
**Comments**				*CEP290* (NM_025114): flanking sequence of exon 32, exon 7 and exon 5 not complete covered.														

	RPGRIP1L			*BBS2*			*NEK8*			*HNF1B*			*ACE*			*MKKS*			*XPNPEP3*		
Patient	Av. Cov.	% > 15x	% > 20x	Av. Cov.	% > 15x	% > 20x	Av. Cov.	% > 15x	% > 20x	Av. Cov.	% > 15x	% > 20x	Av. Cov.	% > 15x	% > 20x	Av. Cov.	% > 15x	% > 20x	Av. Cov.	% > 15x	% > 20x
1	422.9	98.6	98.4	710.65	100	100	1787.34	100	100	1276.68	100	100	1513.15	99.4	99.4	809.61	100	100	831.5	93.3	93.3
2	368.19	98.9	98.8	563.74	100	100	992.57	100	100	815.01	100	100	863.15	99.4	99.4	627.51	100	100	637.49	93.3	93.3
3	420.95	99	98.8	587.06	100	100	921.11	100	100	810.05	100	100	818.37	99.5	99.4	625.17	100	100	639.64	93.3	93.3
4	521.9	98.9	98.7	792.21	100	100	1343.2	100	100	1098.53	100	100	1152.58	99.6	99.5	865.21	100	100	862.06	93.3	93.3
5	322.61	99	98.9	523.56	100	100	1193.29	100	100	910.23	100	100	1027.63	99.4	99.3	578.17	100	100	629.33	93.3	93.3
6	398.65	98.8	98.3	576.04	100	100	1024.04	100	100	826.24	100	100	872.51	99.5	99.4	662.98	100	100	665.51	93.3	93.3
7	595.95	99.4	99.2	881.6	100	100	1468.22	100	100	1238.62	100	100	1268.93	99.6	99.5	923.46	100	100	981.26	93.3	93.3
8	485.43	99	98.8	805.69	100	100	1841.9	100	100	1358.91	100	100	1565	99.3	99.3	913.72	100	100	940.98	93.3	93.3
9	347.82	99	98.9	562.11	100	100	1132.15	100	100	921.41	100	100	1020.51	99.6	99.6	640.87	100	100	655.65	93.3	93.3
10	412.68	99.2	99.1	676.27	100	100	1491.46	100	100	1202.82	100	100	1314.67	99.6	99.5	721.73	100	100	815.21	93.3	93.3
11	1363.1	98.8	98.7	2045.86	100	100	3222.28	100	100	2747.03	100	100	2841.48	100	100	2114.3	100	100	2195.79	93.3	93.3
12	827.96	99.4	99.4	1206.01	100	100	1924.53	100	100	1687.11	100	100	1730.34	100	100	1346.3	100	100	1291.57	93.3	93.3
13	937.35	99.1	99	1585.71	100	100	3280.65	100	100	2577.62	100	100	2828.18	100	100	1783.86	100	100	1789.82	93.3	93.3
14	1080.9	99	98.9	1811.74	100	100	3751.93	100	100	2883.01	100	100	3229.16	100	100	2080.05	100	100	2071.72	93.3	93.3
15	812.35	99.5	99.4	1241.78	100	100	2356.1	100	100	1862.51	100	100	2104.59	100	100	1384.7	100	100	1402.6	93.3	93.3
16	1160.2	99.1	99.1	1800.55	100	100	3178.52	100	100	2633.34	100	100	2784.45	100	100	1994.79	100	100	1960.8	93.3	93.3
17	1091.6	99.1	99	1774.97	100	100	3473.51	100	100	2825.45	100	100	3119.85	100	100	1961.1	100	100	2020.39	93.3	93.3
18	917.44	99.1	99.1	1535.11	100	100	3174.68	100	100	2497.57	100	100	2745.05	100	100	1758.51	100	100	1811.24	93.3	93.3
19	844.98	98.6	98.4	1463.09	100	100	3170.34	100	100	2368.13	100	100	2756.41	100	100	1677.91	100	100	1708.49	93.3	93.3
20	1070.6	99.4	99.3	1477.77	100	100	2067.59	100	100	1843.59	100	100	1824.68	100	100	1556.28	100	100	1528.62	93.3	93.3
21	802.36	98.6	98.4	1244.45	100	100	2171.64	100	100	1760.9	100	100	1904.34	100	100	1343.08	100	100	1374.37	93.3	93.3
22	1478.9	99.5	99.4	2204.07	100	100	3484.77	100	100	2949.42	100	100	3101.02	100	100	2288.79	100	100	2285.68	93.3	93.3
23	823.88	99.3	99.3	1302.2	100	100	2349.16	100	100	1914.11	100	100	2059.65	100	100	1433.92	100	100	1452.48	93.3	93.3
24	953.62	99.1	99	1516.38	100	100	2768.18	100	100	2241.65	100	100	2411.91	100	100	1731.25	100	100	1705.89	93.3	93.3
25	946.14	99.4	99.2	1577.11	100	100	2984.54	100	100	2389.34	100	100	2578.21	100	100	1715.56	100	100	1782.75	93.3	93.3
26	1002.1	99.1	99.1	1636.08	100	100	3151.65	100	100	2546.33	100	100	2785.2	100	100	1806.32	100	100	1877.73	93.3	93.3
27	881.53	99.4	99.4	1454.77	100	100	2832.59	100	100	2252.31	100	100	2454.66	100	100	1627.72	100	100	1668.03	93.3	93.3
28	983.69	99.5	99.4	1620.1	100	100	3124.83	100	100	2490.33	100	100	2738.03	100	100	1758.21	100	100	1779.45	93.3	93.3
29	903.23	99	98.8	1444.38	100	100	2939.8	100	100	2247.07	100	100	2502.3	100	100	1697.33	100	100	1654.97	93.3	93.3
30	1058.7	99.5	99.3	1500.02	100	100	2260.05	100	100	2025.16	100	100	2074.78	100	100	1663.88	100	100	1611.66	93.3	93.3
31	1052.6	99.1	99	1714.8	100	100	3434.43	100	100	2670.7	100	100	2980.11	100	100	1952.25	100	100	1973.19	93.3	93.3
32	602.65	99.3	99.2	808.98	100	100	1115.59	100	100	986.03	100	100	1000.88	100	100	856.09	100	100	802.29	93.3	93.3
33	1061.8	99.2	99.2	1817.83	100	100	4040.71	100	100	3036.08	100	100	3424.79	100	100	2054.54	100	100	2107.89	93.3	93.3
34	900.53	99.1	99.1	1340.68	100	100	2313.77	100	100	1926.67	100	100	2050.02	100	100	1536.42	100	100	1497.22	93.3	93.3
35	981.99	99.1	99.1	1409.29	100	100	2196.04	100	100	1919.98	100	100	1991.85	100	100	1629.74	100	100	1568.48	93.3	93.3
36	1042.6	99.2	99.1	1595.8	100	100	2691.63	100	100	2218.86	100	100	2405.92	100	100	1782.55	100	100	1738.72	93.3	93.3
37	997.24	99.3	99.2	1447.31	100	100	2373.49	100	100	2019.4	100	100	2113.54	100	100	1620.36	100	100	1570.65	93.3	93.3
38	1171.6	99.1	99	1838.61	100	100	3254.82	100	100	2650.51	100	100	2899.28	100	100	2057.45	100	100	2048.33	93.3	93.3
39	1117.1	99.4	99.3	1629.09	100	100	2550.95	100	100	2216.4	100	100	2293	100	100	1769.5	100	100	1742.06	93.3	93.3
40	1121.5	99.3	99.1	1737.26	100	100	2989.58	100	100	2481.94	100	100	2647.5	100	100	1946.38	100	100	1914.27	93.3	93.3
41	888.49	99.3	99.1	1368.57	100	100	2413.54	100	100	2009.79	100	100	2146.9	100	100	1540.59	100	100	1501.67	93.3	93.3
42	966.62	99.3	99.2	1373.95	100	100	2120.11	100	100	1862.02	100	100	1906	100	100	1560.83	100	100	1473.89	93.3	93.3
43	1016.4	99.2	99.1	1587.25	100	100	2796.99	100	100	2290.25	100	100	2469.85	100	100	1744.45	100	100	1721.53	93.3	93.3
44	812.22	99.1	99.1	1110.52	100	100	1761.11	100	100	1467.12	100	100	1515.3	100	100	1222	100	100	1196.18	93.3	93.3
45	731.44	99.4	99.4	1000.57	100	100	1442.92	100	100	1208.58	100	100	1245.84	99.7	99.6	1115.22	100	100	1046.8	93.3	93.3
46	1089.1	99.5	99.4	1583.02	100	100	2439.26	100	100	2063.37	100	100	2159.97	99.8	99.6	1656.38	100	100	1688.5	93.3	93.3
47	913.55	99.4	99.3	1445.74	100	100	2833.56	100	100	2274.43	100	100	2490.67	100	100	1666.82	100	100	1627.69	93.3	93.3
48	1124	99.4	99.3	1726.25	100	100	3090.85	100	100	2559.4	100	100	2742.81	100	100	1978.94	100	100	1926.04	93.3	93.3
49	1199.8	99.4	99.3	1976.21	100	100	4010.34	100	100	3203.12	100	100	3491.16	100	100	2233.39	100	100	2334.99	93.3	93.3
50	992.25	99.1	99.1	1597.87	100	100	3258.13	100	100	2555.62	100	100	2801.39	100	100	1823.73	100	100	1837.77	93.3	93.3
51	1006.7	99.1	99	1637.46	100	100	3354.04	100	100	2568.02	100	100	2832.32	100	100	1817.62	100	100	1848.71	93.3	93.3
52	1114.5	99.3	99.1	1793.3	100	100	3246.16	100	100	2672.65	100	100	2858.11	100	100	1978.92	100	100	1996.4	93.3	93.3
53	645.61	99.2	98.9	790.91	100	100	1002.02	100	100	875.01	100	100	894.36	100	100	845.7	100	100	809.68	93.3	93.3
54	1231.4	99.2	99.1	1674.67	100	100	2408.5	100	100	2121.2	100	100	2109.76	100	100	1792.7	100	100	1727.23	93.3	93.3
55	1115.3	99.3	99.2	1844.74	100	100	3847.84	100	100	3008.41	100	100	3356.47	100	100	2046.37	100	100	2148.68	93.3	93.3
**Max**		**99.5**	**99.4**		**100**	**100**		**100**	**100**		**100**	**100**		**100**	**100**		**100**	**100**		**93.3**	**93.3**
**Mean**		**99.2**	**99.1**		**100**	**100**		**100**	**100**		**100**	**100**		**99.9**	**99.9**		**100**	**100**		**93.3**	**93.3**
**Min**		**98.6**	**98.3**		**100**	**100**		**100**	**100**		**100**	**100**		**99.3**	**99.3**		**100**	**100**		**93.3**	**93.3**
**Comments**	*RPGRIP1L* (NM_015272): exon 23 not complete covered.																		*XPNPEP3* (NM_001204827): exon 2 included in design but not enriched.		
