# Supplementary material for: An Efficient and Comprehensive Strategy for Genetic Diagnostics of Polycystic Kidney Disease
Source: PLoS One. 2015 Feb 3;10(2):e0116680. doi: 10.1371/journal.pone.0116680 (PMC4315576; doi:10.1371/journal.pone.0116680)
Supplement: S4 Table — (PDF) [file pone.0116680.s014.pdf]

Table S4. List of all variants included in this study as detected by conventional LR-PCR-Sanger sequencing and by our NGS approach.
All variants mentioned in this study with their exact positions, read depth, allele distribution, annotation in databases and classifications are listed. Pat-no – patient number; c.-pos – coding position; p.-pos - amino acid position; 1kG - allele frequency in the 1000 Genomes project; ESP – allele frequency in the exome sequencing project; m – match (correct detection of variant; 100% concordance of Sanger and NGS result); d - detected (below 20% reads-threshold, different zygosity); n – not detected; p – only detected in bioinformatic NGS pipeline; zygosity – incorrectly called zygosity in NGS data, Sanger result set as correct; FP- false positive; NA - not available; R – reference alleles; A - alternative alleles; mutation? - putatively pathogenic variant (based on bioinformatic prediction or variant of indefinite nature or being private to the patient) (Table S6).
Below: exact validation statistics in the *PKD1* patient cohort.

Pat-no	exon	hg19 position	c.-pos	p.-pos	zygosity	rs-number	PKD DB entry	NGS	PubMed-ID	1kG	ESP	classification	reads	% reads	Instrument	Statistics	TNs per sample	Comment
1	5	chr16:2167970	c.1023C>T	p.Ala341Ala	het	NA	likely neutral	m	NA	0.00%	0.00%		1072 R | 667 A	38%	MiSeq	TP		
1	5	chr16:2167874	c.1119C>T	p.Leu373Leu	het	rs199685642	likely neutral	m	NA	0.00%	0.00%		1772 R | 461 A	21%	MiSeq	TP		
1	11	chr16:2164808	c.2216G>A	p.Arg739Gln	hom	rs40433	likely neutral	m	NA	91.00%	0.00%		20 R | 958 A	98%	MiSeq	TP		
1	13	chr16:2162984	c.2986-20A>C	NA	het	NA	NA	m	NA	0.00%	0.00%		1881 R | 1528 A	45%	MiSeq	TP		
1	15	chr16:2160154_2160153	c.5014_5015delAG	p.Arg1672Glyfs*98	het	NA	definitely pathogenic	m	10577909	0.00%	0.00%	mutation	816 R | 979 A	55%	MiSeq	TP		
1	22	chr16:2154478	c.8161+21T>C	NA	het	rs4786209	likely neutral	m	NA	56.00%	0.00%		261 R | 313 A	55%	MiSeq	TP		
1	44	chr16:2140794	c.12019C>T	p.Arg4007Cys	het	NA	highly likely pathoger	m	NA	0.00%	0.00%	mutation?	878 R | 838 A	49%	MiSeq	TP	153	
2	5	chr16:2168022	c.971G>T	p.Arg324Leu	het	rs199476099	likely neutral	m	10364515	0.00%	0.16%		491 R | 283 A	37%	MiSeq	TP		
2	5	chr16:2167970	c.1023C>T	p.Ala341Ala	het	NA	likely neutral	m	NA	0.00%	0.00%		717 R | 336 A	32%	MiSeq	TP		
2	5	chr16:2167874	c.1119C>T	p.Leu373Leu	hom	rs199685642	likely neutral	d	NA	0.00%	0.00%		776 R | 463 A	37%	MiSeq	TP		zygosity
2	11	chr16:2164808	c.2216G>A	p.Arg739Gln	hom	rs40433	likely neutral	m	NA	91.00%	0.00%		21 R | 459 A	96%	MiSeq	TP		
2	15	chr16:2159128	c.6040C>T	p.Gln2014*	het	NA	definitely pathogenic	m	12007219	0.00%	0.00%	mutation	562 R | 496 A	47%	MiSeq	TP		
2	22	chr16:2154478	c.8161+21T>C	NA	hom	rs4786209	likely neutral	m	NA	56.00%	0.00%		6 R | 267 A	98%	MiSeq	TP		
2	11	chr16:2164211	c.2813C>T	p.Thr938Met	NA	rs148709380	NA	p	NA	5.00%	0.00%		693 R | 193 A	22%	MiSeq	FP	154	
3	5	chr16:2167874	c.1119C>T	p.Leu373Leu	hom	rs199685642	likely neutral	d	NA	0.00%	0.00%		847 R | 382 A	31%	MiSeq	TP		zygosity
3	8	chr16:2166672	c.1607-27C>T	NA	het	rs4787158	likely neutral	m	NA	4.00%	2.87%		219 R | 203 A	48%	MiSeq	TP		
3	11	chr16:2164808	c.2216G>A	p.Arg739Gln	hom	rs40433	likely neutral	m	NA	91.00%	0.00%		5 R | 370 A	99%	MiSeq	TP		
3	18	chr16:2156543_2156532	c.7345_7356del	p.Thr2449_Gly2452del	het	NA	NA	m	NA	0.00%	0.00%	mutation?	401 R | 204 A	34%	MiSeq	TP		
3	22	chr16:2154478	c.8161+21T>C	NA	het	rs4786209	likely neutral	m	NA	56.00%	0.00%		103 R | 111 A	52%	MiSeq	TP		
3	22	chr16:2154470	c.8161+29G>A	NA	het	rs375810914	likely neutral	m	NA	0.84%	0.00%		101 R | 83 A	45%	MiSeq	TP		
3	35	chr16:2144182	c.10529C>T	p.Thr3510Met	het	rs45478794	likely neutral	m	11558899	2.00%	0.52%		385 R | 375 A	49%	MiSeq	TP	153	
4	5	chr16:2167874	c.1119C>T	p.Leu373Leu	hom	rs199685642	likely neutral	d	NA	0.00%	0.00%		640 R | 515 A	45%	MiSeq	TP		zygosity
4	9	chr16:2165965_2165959	c.1849+28_1849+34del	NA	hom	rs56173969	likely neutral	m	NA	9.00%	0.00%		11 R | 508 A	98%	MiSeq	TP		
4	10	chr16:2165630	c.1850-4A>G	NA	hom	rs35929659	likely neutral	m	NA	21.00%	22.78%		61 R | 853 A	93%	MiSeq	TP		
4	11	chr16:2164808	c.2216G>A	p.Arg739Gln	hom	rs40433	likely neutral	m	NA	91.00%	0.00%		13 R | 510 A	98%	MiSeq	TP		
4	11	chr16:2164324	c.2700G>A	p.Pro900Pro	hom	rs35667726	likely neutral	m	NA	3.00%	5.31%		113 R | 1202 A	91%	MiSeq	TP		
4	11	chr16:2164294	c.2730C>T	p.Asp910Asp	hom	rs35965348	likely neutral	m	NA	3.00%	5.33%		99 R | 1147 A	92%	MiSeq	TP		
4	15	chr16:2160503	c.4665A>C	p.Ala1555Ala	hom	rs71385734	likely neutral	m	NA	21.00%	28.26%		129 R | 1534 A	92%	MiSeq	TP		
4	15	chr16:2159996	c.5172C>T	p.Ala1724Ala	hom	rs9935526	likely neutral	m	NA	20.00%	27.23%		110 R | 1342 A	92%	MiSeq	TP		
4	15	chr16:2159405	c.5763G>A	p.Leu1921Leu	het	rs2575313	likely neutral	m	NA	2.00%	3.44%		563 R | 491 A	47%	MiSeq	TP		
4	17	chr16:2156850	c.7165T>C	p.Leu2389Leu	hom	rs2457533	likely neutral	d	NA	15.00%	0.00%		75 R | 91 A	55%	MiSeq	TP		zygosity
4	18	chr16:2156570	c.7303_7317dup	p.Arg2435_Arg2439dup	het	NA	NA	m	NA	0.00%	0.00%	mutation?	633 R | 198 A	24%	MiSeq	TP		
4	18	chr16:2156447	c.7441C>T	p.Leu2481Leu	hom	rs2003782	likely neutral	m	NA	21.00%	20.68%		89 R | 783 A	90%	MiSeq	TP		
4	21	chr16:2155297	c.8016+26T>C	NA	hom	rs9934488	likely neutral	m	NA	20.00%	0.00%		102 R | 1113 A	92%	MiSeq	TP		
4	22	chr16:2154478	c.8161+21T>C	NA	het	rs4786209	likely neutral	m	NA	56.00%	0.00%		115 R | 162 A	58%	MiSeq	TP		
4	25	chr16:2152651	c.8949-17A>G	NA	hom	rs9928278	likely neutral	m	NA	21.00%	27.84%		91 R | 1193 A	93%	MiSeq	TP		
4	25	chr16:2152388	c.9195G>C	p.Val3065Val	hom	rs9935834	NA	m	NA	19.00%	16.44%		106 R | 1182 A	92%	MiSeq	TP		
4	25	chr16:2152387	c.9196T>C	p.Phe3066Leu	hom	rs77028972	likely neutral	m	NA	19.00%	16.13%		107 R | 1175 A	92%	MiSeq	TP		
4	26	chr16:2152129	c.9330T>C	p.Pro3110Pro	hom	rs144582212	likely neutral	d	NA	11.00%	0.00%		95 R | 277 A	74%	MiSeq	TP		zygosity
4	28	chr16:2150323	c.9569-13T>C	NA	het	rs11248911	likely neutral	m	NA	11.00%	0.00%		796 R | 589 A	43%	MiSeq	TP		
4	35	chr16:2144176	c.10535C>T	p.Ala3512Val	het	rs34197769	likely neutral	m	NA	7.00%	9.61%		475 R | 486 A	51%	MiSeq	TP		
4	42	chr16:2141396	c.11712+28G>C	NA	hom	rs11866494	likely neutral	m	NA	26.00%	0.00%		6 R | 37 A	86%	MiSeq	TP		
4	42	chr16:2141395	c.11712+29C>T	NA	het	NA	likely neutral	m	NA	0.00%	0.00%		18 R | 25 A	58%	MiSeq	TP		
4	44	chr16:2140680	c.12133A>G	p.Ile4045Val	hom	rs10960	likely neutral	m	NA	24.00%	31.10%		102 R | 1321 A	93%	MiSeq	TP		
4	45	chr16:2140554	c.12176C>T	p.Ala4059Val	het	rs3209986	likely neutral	m	NA	4.00%	5.82%		871 R | 746 A	46%	MiSeq	TP		
4	45	chr16:2140454	c.12276A>G	p.Ala4092Ala	hom	rs3087632	likely neutral	m	NA	26.00%	32.61%		72 R | 1021 A	93%	MiSeq	TP		
4	45	chr16:2140321	c.12409C>T	p.Leu4137Leu	het	rs79899502	likely neutral	m	NA	4.00%	5.84%		509 R | 431 A	46%	MiSeq	TP		
4	46	chr16:2140010	c.12630T>C	p.Pro4210Pro	hom	rs7203729	likely neutral	m	NA	26.00%	33.03%		91 R | 1247 A	93%	MiSeq	TP	133	
5	27	chr16:2150481	c.9484C>T	p.Arg3162Cys	het	NA	likely hypomorphic	m	22034641	0.00%	0.00%	mutation?	880 R | 703 A	44%	MiSeq	TP	NA	only single exon analysis Sanger
6	1	chr16:2185595	c.96C>T	p.Pro32Pro	het	NA	NA	n	NA	0.00%	0.00%		NA	NA	MiSeq	FN		no coverage exon 1
6	2	chr16:2169319	c.276G>A	p.Ala92Ala	het	rs374518168	likely neutral	m	NA	0.00%	0.00%		352 R | 111 A	24%	MiSeq	TP		
6	11	chr16:2164808	c.2216G>A	p.Arg739Gln	het	rs40433	likely neutral	m	NA	91.00%	0.00%		399 R | 203 A	34%	MiSeq	TP		
6	15	chr16:2160494	c.4674G>A	p.Thr1558Thr	het	rs79884128	likely neutral	m	NA	8.00%	5.41%		677 R | 559 A	45%	MiSeq	TP		
6	21	chr16:2155352	c.7987C>T	p.Gln2663*	het	NA	definitely pathogenic	m	22508176	0.00%	0.00%	mutation	590 R | 407 A	41%	MiSeq	TP		
6	22	chr16:2154478	c.8161+21T>C	NA	hom	rs4786209	likely neutral	m	NA	56.00%	0.00%		16 R | 213 A	93%	MiSeq	TP		
6	25	chr16:2152549	c.9034A>G	p.Thr3012Ala	het	NA	NA	m	NA	0.00%	0.00%		635 R | 496 A	44%	MiSeq	TP		
6	37	chr16:2143689	c.10872G>A	p.Pro3624Pro	het	rs201417193	likely neutral	m	NA	0.05%	0.00%		369 R | 393 A	52%	MiSeq	TP	153	
7	5	chr16:2167874	c.1119C>T	p.Leu373Leu	hom	rs199685642	likely neutral	d	NA	0.00%	0.00%		1026 R | 663 A	39%	MiSeq	TP		zygosity
7	11	chr16:2164808	c.2216G>A	p.Arg739Gln	hom	rs40433	likely neutral	m	NA	91.00%	0.00%		25 R | 627 A	96%	MiSeq	TP		
7	13	chr16:2162839	c.3111A>G	p.Leu1037Leu	het	rs2099534	likely neutral	m	NA	2.00%	4.13%		1007 R | 765 A	43%	MiSeq	TP		
7	15	chr16:2160673	c.4495C>T	p.Leu1499Leu	het	rs142002333	likely neutral	m	NA	0.23%	0.38%		824 R | 601 A	42%	MiSeq	TP		
7	15	chr16:2160547	c.4621A>C	p.Asn1541His	het	NA	NA	m	NA	0.00%	0.00%	mutation?	958 R | 748 A	44%	MiSeq	TP		
7	18	chr16:2156369	c.7489+30C>T	NA	het	rs142761413	NA	m	NA	2.00%	1.60%		744 R | 643 A	46%	MiSeq	TP		
7	22	chr16:2154478	c.8161+21T>C	NA	het	rs4786209	likely neutral	m	NA	56.00%	0.00%		201 R | 264 A	57%	MiSeq	TP	153	
8	5	chr16:2167874	c.1119C>T	p.Leu373Leu	hom	rs199685642	likely neutral	d	NA	0.00%	0.00%		1298 R | 882 A	40%	MiSeq	TP		zygosity
8	11	chr16:2164844	c.2180T>C	p.Leu727Pro	het	NA	NA	d	17582161	0.00%	0.00%	mutation?	1300 R | 138 A	10%	MiSeq	TP		
8	11	chr16:2164808	c.2216G>A	p.Arg739Gln	hom	rs40433	likely neutral	m	NA	91.00%	0.00%		26 R | 740 A	97%	MiSeq	TP		
8	17	chr16:2156850	c.7165T>G	p.Leu2389Val	het	NA	NA	m	NA	0.00%	0.00%		809 R | 614 A	43%	MiSeq	TP		
8	19	chr16:2156112	c.7683C>T	p.Ala2561Ala	het	NA	likely neutral	m	NA	0.00%	0.00%		1157 R | 853 A	42%	MiSeq	TP		

<table>
<tr><td>8</td><td>22</td><td>chr16:2154478</td><td>c.8161+21T>C</td><td>NA</td><td>het</td><td>rs4786209</td><td>likely neutral</td><td>m</td><td>NA</td><td>56.00%</td><td>0.00%</td><td></td><td>227 R | 266 A</td><td>54%</td><td>MiSeq</td><td>TP</td><td></td><td></td></tr>
<tr><td>8</td><td>43</td><td>chr16:2141018</td><td>c.11870G>A</td><td>p.Gly3957Asp</td><td>het</td><td>NA</td><td>likely neutral</td><td>m</td><td>NA</td><td>0.00%</td><td>0.00%</td><td></td><td>695 R | 691 A</td><td>50%</td><td>MiSeq</td><td>TP</td><td>153</td><td></td></tr>
<tr><td>9</td><td>5</td><td>chr16:2167874</td><td>c.1119C>T</td><td>p.Leu373Leu</td><td>het</td><td>rs199685642</td><td>likely neutral</td><td>d</td><td>NA</td><td>0.00%</td><td>0.00%</td><td></td><td>1298 R | 247 A</td><td>16%</td><td>MiSeq</td><td>TP</td><td></td><td></td></tr>
<tr><td>9</td><td>9</td><td>chr16:2165965_2165959</td><td>c.1849+28_1849+34del</td><td>NA</td><td>het</td><td>rs56173969</td><td>likely neutral</td><td>m</td><td>NA</td><td>9.00%</td><td>0.00%</td><td></td><td>61 R | 239 A</td><td>80%</td><td>MiSeq</td><td>TP</td><td></td><td></td></tr>
<tr><td>9</td><td>10</td><td>chr16:2165630</td><td>c.1850-4A>G</td><td>NA</td><td>het</td><td>rs35929659</td><td>likely neutral</td><td>m</td><td>NA</td><td>21.00%</td><td>22.78%</td><td></td><td>441 R | 448 A</td><td>50%</td><td>MiSeq</td><td>TP</td><td></td><td></td></tr>
<tr><td>9</td><td>11</td><td>chr16:2164808</td><td>c.2216G>A</td><td>p.Arg739Gln</td><td>het</td><td>rs40433</td><td>likely neutral</td><td>m</td><td>NA</td><td>91.00%</td><td>0.00%</td><td></td><td>585 R | 343 A</td><td>37%</td><td>MiSeq</td><td>TP</td><td></td><td></td></tr>
<tr><td>9</td><td>11</td><td>chr16:2164324</td><td>c.2700G>A</td><td>p.Pro900Pro</td><td>het</td><td>rs35667726</td><td>likely neutral</td><td>m</td><td>NA</td><td>3.00%</td><td>5.31%</td><td></td><td>663 R | 554 A</td><td>46%</td><td>MiSeq</td><td>TP</td><td></td><td></td></tr>
<tr><td>9</td><td>11</td><td>chr16:2164294</td><td>c.2730C>T</td><td>p.Asp910Asp</td><td>het</td><td>rs35965348</td><td>likely neutral</td><td>m</td><td>NA</td><td>3.00%</td><td>5.33%</td><td></td><td>669 R | 547 A</td><td>45%</td><td>MiSeq</td><td>TP</td><td></td><td></td></tr>
<tr><td>9</td><td>15</td><td>chr16:2160503</td><td>c.4665A>C</td><td>p.Ala1555Ala</td><td>het</td><td>rs71385734</td><td>likely neutral</td><td>m</td><td>NA</td><td>21.00%</td><td>28.26%</td><td></td><td>913 R | 726 A</td><td>44%</td><td>MiSeq</td><td>TP</td><td></td><td></td></tr>
<tr><td>9</td><td>15</td><td>chr16:2160154_2160153</td><td>c.5014_5015delAG</td><td>p.Arg1672Glyfs*98</td><td>het</td><td>NA</td><td>definitely pathogenic</td><td>m</td><td>10577909</td><td>0.00%</td><td>0.00%</td><td>mutation</td><td>675 R | 664 A</td><td>50%</td><td>MiSeq</td><td>TP</td><td></td><td></td></tr>
<tr><td>9</td><td>15</td><td>chr16:2159996</td><td>c.5172C>T</td><td>p.Ala1724Ala</td><td>het</td><td>rs9935526</td><td>likely neutral</td><td>m</td><td>NA</td><td>20.00%</td><td>27.23%</td><td></td><td>751 R | 671 A</td><td>47%</td><td>MiSeq</td><td>TP</td><td></td><td></td></tr>
<tr><td>9</td><td>15</td><td>chr16:2159405</td><td>c.5763G>A</td><td>p.Leu1921Leu</td><td>het</td><td>rs2575313</td><td>likely neutral</td><td>m</td><td>NA</td><td>2.00%</td><td>3.44%</td><td></td><td>516 R | 391 A</td><td>43%</td><td>MiSeq</td><td>TP</td><td></td><td></td></tr>
<tr><td>9</td><td>17</td><td>chr16:2156850</td><td>c.7165T>C</td><td>p.Leu2389Leu</td><td>het</td><td>rs2457533</td><td>likely neutral</td><td>d</td><td>NA</td><td>15.00%</td><td>0.00%</td><td></td><td>532 R | 58 A</td><td>10%</td><td>MiSeq</td><td>TP</td><td></td><td></td></tr>
<tr><td>9</td><td>18</td><td>chr16:2156447</td><td>c.7441C>T</td><td>p.Leu2481Leu</td><td>het</td><td>rs2003782</td><td>likely neutral</td><td>m</td><td>NA</td><td>21.00%</td><td>20.68%</td><td></td><td>435 R | 390 A</td><td>47%</td><td>MiSeq</td><td>TP</td><td></td><td></td></tr>
<tr><td>9</td><td>22</td><td>chr15:2154537</td><td>c.8123C>T</td><td>p.Thr2708Met</td><td>het</td><td>rs147350387</td><td>likely neutral</td><td>m</td><td>NA</td><td>0.00%</td><td>1.14%</td><td></td><td>343 R | 265 A</td><td>44%</td><td>MiSeq</td><td>TP</td><td></td><td></td></tr>
<tr><td>9</td><td>22</td><td>chr16:2154478</td><td>c.8161+21T>C</td><td>NA</td><td>hom</td><td>rs4786209</td><td>likely neutral</td><td>m</td><td>NA</td><td>56.00%</td><td>0.00%</td><td></td><td>3 R | 300 A</td><td>99%</td><td>MiSeq</td><td>TP</td><td></td><td></td></tr>
<tr><td>9</td><td>25</td><td>chr16:2152651</td><td>c.8949-17A>G</td><td>NA</td><td>het</td><td>rs9928278</td><td>likely neutral</td><td>m</td><td>NA</td><td>21.00%</td><td>27.84%</td><td></td><td>684 R | 510 A</td><td>43%</td><td>MiSeq</td><td>TP</td><td></td><td></td></tr>
<tr><td>9</td><td>25</td><td>chr16:2152388</td><td>c.9195G>C</td><td>p.Val3065Val</td><td>het</td><td>rs9935834</td><td>NA</td><td>m</td><td>NA</td><td>19.00%</td><td>16.44%</td><td></td><td>795 R | 582 A</td><td>42%</td><td>MiSeq</td><td>TP</td><td></td><td></td></tr>
<tr><td>9</td><td>25</td><td>chr16:2152387</td><td>c.9196T>C</td><td>p.Phe3066Leu</td><td>het</td><td>rs77028972</td><td>likely neutral</td><td>m</td><td>NA</td><td>19.00%</td><td>16.13%</td><td></td><td>797 R | 578 A</td><td>42%</td><td>MiSeq</td><td>TP</td><td></td><td></td></tr>
<tr><td>9</td><td>26</td><td>chr16:2152129</td><td>c.9330T>C</td><td>p.Pro3110Pro</td><td>het</td><td>rs144582212</td><td>likely neutral</td><td>m</td><td>NA</td><td>11.00%</td><td>0.00%</td><td></td><td>567 R | 133 A</td><td>19%</td><td>MiSeq</td><td>TP</td><td></td><td></td></tr>
<tr><td>9</td><td>28</td><td>chr16:2150323</td><td>c.9569-13T>C</td><td>NA</td><td>het</td><td>rs11248911</td><td>likely neutral</td><td>m</td><td>NA</td><td>11.00%</td><td>0.00%</td><td></td><td>779 R | 543 A</td><td>41%</td><td>MiSeq</td><td>TP</td><td></td><td></td></tr>
<tr><td>9</td><td>35</td><td>chr16:2144176</td><td>c.10535C>T</td><td>p.Ala3512Val</td><td>het</td><td>rs34197769</td><td>likely neutral</td><td>m</td><td>NA</td><td>7.00%</td><td>9.61%</td><td></td><td>477 R | 460 A</td><td>49%</td><td>MiSeq</td><td>TP</td><td></td><td></td></tr>
<tr><td>9</td><td>42</td><td>chr16:2141396</td><td>c.11712+28G>C</td><td>NA</td><td>het</td><td>rs11866494</td><td>likely neutral</td><td>m</td><td>NA</td><td>26.00%</td><td>0.00%</td><td></td><td>4 R | 6 A</td><td>60%</td><td>MiSeq</td><td>TP</td><td></td><td></td></tr>
<tr><td>9</td><td>42</td><td>chr16:2141395</td><td>c.11712+29C>T</td><td>NA</td><td>het</td><td>NA</td><td>likely neutral</td><td>m</td><td>NA</td><td>0.00%</td><td>0.00%</td><td></td><td>4 R | 6 A</td><td>60%</td><td>MiSeq</td><td>TP</td><td></td><td></td></tr>
<tr><td>9</td><td>44</td><td>chr16:2140680</td><td>c.12133A>G</td><td>p.Ile4045Val</td><td>het</td><td>rs10960</td><td>likely neutral</td><td>m</td><td>NA</td><td>24.00%</td><td>31.10%</td><td></td><td>725 R | 699 A</td><td>49%</td><td>MiSeq</td><td>TP</td><td></td><td></td></tr>
<tr><td>9</td><td>45</td><td>chr16:2140554</td><td>c.12176C>T</td><td>p.Ala4059Val</td><td>het</td><td>rs3209986</td><td>likely neutral</td><td>m</td><td>NA</td><td>4.00%</td><td>5.82%</td><td></td><td>744 R | 685 A</td><td>48%</td><td>MiSeq</td><td>TP</td><td></td><td></td></tr>
<tr><td>9</td><td>45</td><td>chr16:2140454</td><td>c.12276A>G</td><td>p.Ala4092Ala</td><td>het</td><td>rs3087632</td><td>likely neutral</td><td>m</td><td>NA</td><td>26.00%</td><td>32.61%</td><td></td><td>511 R | 435 A</td><td>46%</td><td>MiSeq</td><td>TP</td><td></td><td></td></tr>
<tr><td>9</td><td>45</td><td>chr16:2140321</td><td>c.12409C>T</td><td>p.Leu4137Leu</td><td>het</td><td>rs79899502</td><td>likely neutral</td><td>m</td><td>NA</td><td>4.00%</td><td>5.84%</td><td></td><td>399 R | 391 A</td><td>49%</td><td>MiSeq</td><td>TP</td><td></td><td></td></tr>
<tr><td>9</td><td>46</td><td>chr16:2140010</td><td>c.12630T>C</td><td>p.Pro4210Pro</td><td>het</td><td>rs7203729</td><td>likely neutral</td><td>m</td><td>NA</td><td>26.00%</td><td>33.03%</td><td></td><td>705 R | 510 A</td><td>42%</td><td>MiSeq</td><td>TP</td><td></td><td></td></tr>
<tr><td>9</td><td>10</td><td>chr16:2165395</td><td>c.2081C>T</td><td>p.Pro694Leu</td><td>NA</td><td>rs138575342</td><td>NA</td><td>p</td><td>NA</td><td>0.00%</td><td>0.00%</td><td></td><td>719 R | 197 A</td><td>22%</td><td>MiSeq</td><td>FP</td><td></td><td></td></tr>
<tr><td>9</td><td>21</td><td>chr16:2155297</td><td>c.8016+26T>C</td><td>NA</td><td>het</td><td>rs9934488</td><td>likely neutral</td><td>m</td><td>NA</td><td>20.00%</td><td>0.00%</td><td></td><td>645 R | 512 A</td><td>44%</td><td>MiSeq</td><td>TP</td><td>132</td><td></td></tr>
<tr><td>10</td><td>5</td><td>chr16:2167874</td><td>c.1119C>T</td><td>p.Leu373Leu</td><td>hom</td><td>rs199685642</td><td>likely neutral</td><td>d</td><td>NA</td><td>0.00%</td><td>0.00%</td><td></td><td>1048 R | 816 A</td><td>44%</td><td>MiSeq</td><td>TP</td><td></td><td>zygosity</td></tr>
<tr><td>10</td><td>11</td><td>chr16:2164808</td><td>c.2216G>A</td><td>p.Arg739Gln</td><td>hom</td><td>rs40433</td><td>likely neutral</td><td>m</td><td>NA</td><td>91.00%</td><td>0.00%</td><td></td><td>18 R | 909 A</td><td>98%</td><td>MiSeq</td><td>TP</td><td></td><td></td></tr>
<tr><td>10</td><td>22</td><td>chr16:2154478</td><td>c.8161+21T>C</td><td>NA</td><td>het</td><td>rs4786209</td><td>likely neutral</td><td>m</td><td>NA</td><td>56.00%</td><td>0.00%</td><td></td><td>196 R | 230 A</td><td>54%</td><td>MiSeq</td><td>TP</td><td></td><td></td></tr>
<tr><td>10</td><td>23</td><td>chr16:2153765</td><td>c.8293C>T</td><td>p.Arg2765Cys</td><td>het</td><td>rs144979397</td><td>likely hypomorphic</td><td>m</td><td>19165178</td><td>0.46%</td><td>0.58%</td><td>mutation?</td><td>810 R | 684 A</td><td>46%</td><td>MiSeq</td><td>TP</td><td></td><td></td></tr>
<tr><td>10</td><td>25</td><td>chr16:2152516</td><td>c.9067A>G</td><td>p.Met3023Val</td><td>het</td><td>rs17135779</td><td>NA</td><td>m</td><td>NA</td><td>0.00%</td><td>0.00%</td><td></td><td>886 R | 713 A</td><td>45%</td><td>MiSeq</td><td>TP</td><td></td><td></td></tr>
<tr><td>10</td><td>33</td><td>chr16:2147362</td><td>c.10363C>T</td><td>p.Leu3455Leu</td><td>het</td><td>rs114102239</td><td>likely neutral</td><td>m</td><td>NA</td><td>0.05%</td><td>0.03%</td><td></td><td>723 R | 727 A</td><td>50%</td><td>MiSeq</td><td>TP</td><td>154</td><td></td></tr>
<tr><td>11</td><td>11</td><td>chr16:2164808</td><td>c.2216G>A</td><td>p.Arg739Gln</td><td>hom</td><td>rs40433</td><td>likely neutral</td><td>m</td><td>NA</td><td>91.00%</td><td>0.00%</td><td></td><td>31 R | 1216 A</td><td>98%</td><td>HiSeq</td><td>TP</td><td></td><td></td></tr>
<tr><td>11</td><td>13</td><td>chr16:2162839</td><td>c.3111A>G</td><td>p.Leu1037Leu</td><td>het</td><td>rs2099534</td><td>likely neutral</td><td>m</td><td>NA</td><td>0.00%</td><td>0.00%</td><td></td><td>NA</td><td>NA</td><td>HiSeq</td><td>TN</td><td></td><td>PG specific? TN</td></tr>
<tr><td>11</td><td>13</td><td>chr16:2162917</td><td>c.3033A>C</td><td>p.Val1011Ile</td><td>het</td><td>rs2369067</td><td>NA</td><td>m</td><td>NA</td><td>0.00%</td><td>0.00%</td><td></td><td>NA</td><td>NA</td><td>HiSeq</td><td>TN</td><td></td><td>PG specific? TN</td></tr>
<tr><td>11</td><td>13</td><td>chr16:2162919</td><td>c.3031G>A</td><td>p.Val1011Ile</td><td>het</td><td>rs2369066</td><td>NA</td><td>m</td><td>NA</td><td>0.00%</td><td>0.00%</td><td></td><td>NA</td><td>NA</td><td>HiSeq</td><td>TN</td><td></td><td>PG specific? TN</td></tr>
<tr><td>11</td><td>15</td><td>chr16:2160494</td><td>c.4674G>A</td><td>p.Thr1558Thr</td><td>het</td><td>rs79884128</td><td>likely neutral</td><td>m</td><td>NA</td><td>8.00%</td><td>5.41%</td><td></td><td>2039 R | 1693 A</td><td>45%</td><td>HiSeq</td><td>TP</td><td></td><td></td></tr>
<tr><td>11</td><td>15</td><td>chr16:2160904</td><td>c.4264G>A</td><td>p.Ala1422Thr</td><td>het</td><td>rs140980374</td><td>likely neutral</td><td>m</td><td>NA</td><td>0.37%</td><td>0.62%</td><td></td><td>1652 R | 1478 A</td><td>47%</td><td>HiSeq</td><td>TP</td><td></td><td></td></tr>
<tr><td>11</td><td>38</td><td>chr16:2143015_2143007</td><td>c.11096_11104del</td><td>p.Tyr3699*</td><td>het</td><td>NA</td><td>NA</td><td>m</td><td>NA</td><td>0.00%</td><td>0.00%</td><td>mutation</td><td>1874 R | 1089 A</td><td>37%</td><td>HiSeq</td><td>TP</td><td>156</td><td></td></tr>
<tr><td>12</td><td>5</td><td>chr16:2167874</td><td>c.1119C>T</td><td>p.Leu373Leu</td><td>het</td><td>rs199685642</td><td>likely neutral</td><td>d</td><td>NA</td><td>0.00%</td><td>0.00%</td><td></td><td>2755 R | 556 A</td><td>17%</td><td>HiSeq</td><td>TP</td><td></td><td></td></tr>
<tr><td>12</td><td>8</td><td>chr16:2166672</td><td>c.1607-27C>T</td><td>NA</td><td>het</td><td>rs4787158</td><td>likely neutral</td><td>m</td><td>NA</td><td>4.00%</td><td>2.87%</td><td></td><td>469 R | 507 A</td><td>52%</td><td>HiSeq</td><td>TP</td><td></td><td></td></tr>
<tr><td>12</td><td>11</td><td>chr16:2164808</td><td>c.2216G>A</td><td>p.Arg739Gln</td><td>hom</td><td>rs40433</td><td>likely neutral</td><td>m</td><td>NA</td><td>91.00%</td><td>0.00%</td><td></td><td>20 R | 870 A</td><td>98%</td><td>HiSeq</td><td>TP</td><td></td><td></td></tr>
<tr><td>12</td><td>22</td><td>chr16:2154470</td><td>c.8161+29G>A</td><td>NA</td><td>het</td><td>rs375810914</td><td>likely neutral</td><td>m</td><td>NA</td><td>0.84%</td><td>0.00%</td><td></td><td>291 R | 148 A</td><td>34%</td><td>HiSeq</td><td>TP</td><td></td><td></td></tr>
<tr><td>12</td><td>22</td><td>chr16:2154478</td><td>c.8161+21T>C</td><td>NA</td><td>hom</td><td>rs4786209</td><td>likely neutral</td><td>m</td><td>NA</td><td>56.00%</td><td>0.00%</td><td></td><td>29 R | 516 A</td><td>95%</td><td>HiSeq</td><td>TP</td><td></td><td></td></tr>
<tr><td>12</td><td>23</td><td>chr16:2153532_2153530</td><td>c.8526_8528delCAC</td><td>p.Tyr2842_Thr2843delinsT</td><td>het</td><td>NA</td><td>NA</td><td>m</td><td>NA</td><td>0%</td><td>0.00%</td><td>mutation?</td><td>972 R | 740 A</td><td>43%</td><td>HiSeq</td><td>TP</td><td>154</td><td></td></tr>
<tr><td>13</td><td>4</td><td>chr16:2168742</td><td>c.464G>A</td><td>p.Cys155Tyr</td><td>het</td><td>NA</td><td>highly likely pathoger</td><td>m</td><td>22508176</td><td>0.00%</td><td>0.00%</td><td>mutation?</td><td>2305 R | 1372 A</td><td>37%</td><td>HiSeq</td><td>TP</td><td></td><td></td></tr>
<tr><td>13</td><td>5</td><td>chr16:2167874</td><td>c.1119C>T</td><td>p.Leu373Leu</td><td>het</td><td>rs199685642</td><td>likely neutral</td><td>m</td><td>NA</td><td>0.00%</td><td>0.00%</td><td></td><td>3589 R | 958 A</td><td>21%</td><td>HiSeq</td><td>TP</td><td></td><td></td></tr>
<tr><td>13</td><td>11</td><td>chr16:2164808</td><td>c.2216G>A</td><td>p.Arg739Gln</td><td>hom</td><td>rs40433</td><td>likely neutral</td><td>m</td><td>NA</td><td>91.00%</td><td>0.00%</td><td></td><td>17 R | 1786 A</td><td>99%</td><td>HiSeq</td><td>TP</td><td></td><td></td></tr>
<tr><td>13</td><td>16</td><td>chr16:2158022</td><td>c.6927C>T</td><td>p.Gly2309Gly</td><td>het</td><td>rs189277711</td><td>likely neutral</td><td>m</td><td>NA</td><td>1.00%</td><td>1.20%</td><td></td><td>1216 R | 1218 A</td><td>50%</td><td>HiSeq</td><td>TP</td><td></td><td></td></tr>
<tr><td>13</td><td>22</td><td>chr16:2154478</td><td>c.8161+21T>C</td><td>NA</td><td>het</td><td>rs4786209</td><td>likely neutral</td><td>m</td><td>NA</td><td>56.00%</td><td>0.00%</td><td></td><td>471 R | 553 A</td><td>54%</td><td>HiSeq</td><td>TP</td><td></td><td></td></tr>
<tr><td>13</td><td>32</td><td>chr16:2148010</td><td>c.10051-25G>A</td><td>NA</td><td>het</td><td>rs150399422</td><td>likely neutral</td><td>m</td><td>NA</td><td>2.00%</td><td>0.73%</td><td></td><td>1997 R | 1814 A</td><td>48%</td><td>HiSeq</td><td>TP</td><td>154</td><td></td></tr>
<tr><td>14</td><td>5</td><td>chr16:g.2168137_2168131</td><td>c.856_862del</td><td>p.Ser286Serfs*2</td><td>het</td><td>NA</td><td>definitely pathogenic</td><td>m</td><td>21694639</td><td>0.00%</td><td>0.00%</td><td>mutation</td><td>567 R | 549 A</td><td>49%</td><td>HiSeq</td><td>TP</td><td></td><td></td></tr>
<tr><td>14</td><td>10</td><td>chr16:2165395</td><td>c.2081C>T</td><td>p.Pro694Leu</td><td>NA</td><td>rs138575342</td><td>NA</td><td>p</td><td>NA</td><td>0.00%</td><td>0.00%</td><td></td><td>2620 R | 700 A</td><td>21%</td><td>HiSeq</td><td>FP</td><td></td><td></td></tr>
<tr><td>14</td><td>11</td><td>chr16:2164211</td><td>c.2813C>T</td><td>p.Thr938Met</td><td>NA</td><td>rs148709380</td><td>NA</td><td>p</td><td>NA</td><td>5.00%</td><td>0.00%</td><td></td><td>3333 R | 895 A</td><td>21%</td><td>HiSeq</td><td>FP</td><td></td><td></td></tr>
<tr><td>14</td><td>11</td><td>chr16:2164808</td><td>c.2216G>A</td><td>p.Arg739Gln</td><td>hom</td><td>rs40433</td><td>likely neutral</td><td>m</td><td>NA</td><td>91.00%</td><td>0.00%</td><td></td><td>41 R | 2151 A</td><td>98%</td><td>HiSeq</td><td>TP</td><td></td><td></td></tr>
<tr><td>14</td><td>13</td><td>chr16:2162839</td><td>c.3111A>G</td><td>p.Leu1037Leu</td><td>het</td><td>rs2099534</td><td>likely neutral</td><td>m</td><td>NA</td><td>2.00%</td><td>4.13%</td><td></td><td>2691 R | 2189 A</td><td>45%</td><td>HiSeq</td><td>TP</td><td></td><td></td></tr>
<tr><td>14</td><td>22</td><td>chr16:2154640</td><td>c.8020C>T</td><td>p.Pro2674Ser</td><td>het</td><td>rs144557371</td><td>likely neutral</td><td>m</td><td>NA</td><td>0.00%</td><td>0.58%</td><td></td><td>2234 R | 1262 A</td><td>36%</td><td>HiSeq</td><td>TP</td><td></td><td></td></tr>
<tr><td>14</td><td>23</td><td>chr16:2153618</td><td>c.8440G>A</td><td>p.Gly2814Arg</td><td>het</td><td>rs149151043</td><td>likely neutral</td><td>m</td><td>NA</td><td>0.37%</td><td>0.59%</td><td></td><td>1656 R | 1544 A</td><td>48%</td><td>HiSeq</td><td>TP</td><td></td><td></td></tr>
<tr><td>14</td><td>22</td><td>chr16:2154478</td><td>c.8161+21T>C</td><td>NA</td><td>hom</td><td>rs4786209</td><td>likely neutral</td><td>m</td><td>NA</td><td>56.00%</td><td>0.00%</td><td></td><td>21 R | 1239 A</td><td>98%</td><td>HiSeq</td><td>TP</td><td>154</td><td></td></tr>
<tr><td>15</td><td>5</td><td>chr16:2167874</td><td>c.1119C>T</td><td>p.Leu373Leu</td><td>hom</td><td>rs199685642</td><td>likely neutral</td><td>d</td><td>NA</td><td>0.00%</td><td>0.00%</td><td></td><td>3151 R | 1468 A</td><td>32%</td><td>HiSeq</td><td>TP</td><td></td><td>zygosity</td></tr>
<tr><td>15</td><td>11</td><td>chr16:2164808</td><td>c.2216G>A</td><td>p.Arg739Gln</td><td>hom</td><td>rs40433</td><td>likely neutral</td><td>m</td><td>NA</td><td>91.00%</td><td>0.00%</td><td></td><td>19 R | 1629 A</td><td>99%</td><td>HiSeq</td><td>TP</td><td></td><td></td></tr>
<tr><td>15</td><td>22</td><td>chr16:2154478</td><td>c.8161+21T>C</td><td>NA</td><td>het</td><td>rs4786209</td><td>likely neutral</td><td>m</td><td>NA</td><td>56.00%</td><td>0.00%</td><td></td><td>368 R | 386 A</td><td>51%</td><td>HiSeq</td><td>TP</td><td></td><td></td></tr>
<tr><td>15</td><td>23</td><td>chr16:2153747</td><td>c.8311G>A</td><td>p.Glu2771Lys</td><td>het</td><td>NA</td><td>highly likely pathoger</td><td>m</td><td>11115377</td><td>0.00%</td><td>0.00%</td><td>mutation</td><td>1419 R | 1075 A</td><td>43%</td><td>HiSeq</td><td>TP</td><td></td><td></td></tr>
<tr><td>15</td><td>23</td><td>chr16:2153765</td><td>c.8293C>T</td><td>p.Arg2765Cys</td><td>het</td><td>rs144979397</td><td>likely hypomorphic</td><td>m</td><td>19165178</td><td>0.46%</td><td>0.58%</td><td>mutation?</td><td>1441 R | 1232 A</td><td>46%</td><td>HiSeq</td><td>TP</td><td></td><td></td></tr>
<tr><td>15</td><td>25</td><td>chr16:2152396</td><td>c.9187C>T</td><td>p.Arg3063Cys</td><td>het</td><td>rs145906459</td><td>likely neutral</td><td>m</td><td>NA</td><td>0.00%</td><td>0.03%</td><td></td><td>1618 R | 1092 A</td><td>40%</td><td>HiSeq</td><td>TP</td><td>154</td><td></td></tr>
<tr><td>16</td><td>5</td><td>chr16:2167874</td><td>c.1119C>T</td><td>p.Leu373Leu</td><td>het</td><td>rs199685642</td><td>likely neutral</td><td>d</td><td>NA</td><td>0.00%</td><td>0.00%</td><td></td><td>3885 R | 814 A</td><td>17%</td><td>HiSeq</td><td>TP</td><td></td><td></td></tr>
<tr><td>16</td><td>10</td><td>chr16:2165630</td><td>c.1850-4A>G</td><td>NA</td><td>het</td><td>rs35929659</td><td>likely neutral</td><td>m</td><td>NA</td><td>21.00%</td><td>22.78%</td><td></td><td>1392 R | 1122 A</td><td>45%</td><td>HiSeq</td><td>TP</td><td></td><td></td></tr>
<tr><td>16</td><td>11</td><td>chr16:2164808</td><td>c.2216G>A</td><td>p.Arg739Gln</td><td>het</td><td>rs40433</td><td>likely neutral</td><td>m</td><td>NA</td><td>91.00%</td><td>0.00%</td><td></td><td>1509 R | 933 A</td><td>38%</td><td>HiSeq</td><td>TP</td><td></td><td></td></tr>
<tr><td>16</td><td>13</td><td>chr16:2162887</td><td>c.3063T>C</td><td>p.Gly1021Gly</td><td>het</td><td>rs2369068</td><td>likely neutral</td><td>m</td><td>NA</td><td>16.00%</td><td>22.58%</td><td></td><td>2507 R | 1893 A</td><td>43%</td><td>HiSeq</td><td>TP</td><td></td><td></td></tr>
<tr><td>16</td><td>14</td><td>chr16:2162361</td><td>c.3275T>C</td><td>p.Met1092Thr</td><td>het</td><td>rs2549677</td><td>likely neutral</td><td>m</td><td>NA</td><td>17.00%</td><td>0.00%</td><td></td><td>1737 R | 864 A</td><td>33%</td><td>HiSeq</td><td>TP</td><td></td><td></td></tr>
<tr><td>16</td><td>15</td><td>chr16:2159996</td><td>c.5172C>T</td><td>p.Ala1724Ala</td><td>het</td><td>rs9935526</td><td>likely neutral</td><td>m</td><td>NA</td><td>20.00%</td><td>27.23%</td><td></td><td>1885 R | 1751 A</td><td>48%</td><td>HiSeq</td><td>TP</td><td></td><td></td></tr>
<tr><td>16</td><td>15</td><td>chr16:2160503</td><td>c.4665A>C</td><td>p.Ala1555Ala</td><td>het</td><td>rs71385734</td><td>likely neutral</td><td>m</td><td>NA</td><td>21.00%</td><td>28.26%</td><td></td><td>2071 R | 1548 A</td><td>43%</td><td>HiSeq</td><td>TP</td><td></td><td></td></tr>
<tr><td>16</td><td>15</td><td>chr16:2160973</td><td>c.4195T>C</td><td>p.Trp1399Arg</td><td>het</td><td>rs116092985</td><td>likely neutral</td><td>m</td><td>NA</td><td>5.00%</td><td>0.00%</td><td></td><td>2202 R | 1309 A</td><td>37%</td><td>HiSeq</td><td>TP</td><td></td><td></td></tr>
<tr><td>16</td><td>15</td><td>chr16:2161793</td><td>c.3375C>T</td><td>p.Ser1125Ser</td><td>het</td><td>rs74331768</td><td>likely neutral</td><td>m</td><td>NA</td><td>11.00%</td><td>14.45%</td><td></td><td>1768 R | 1328 A</td><td>43%</td><td>HiSeq</td><td>TP</td><td></td><td></td></tr>
<tr><td>16</td><td>15</td><td>chr16:2161796</td><td>c.3372C>T</td><td>p.Ala1124Ala</td><td>het</td><td>rs75510884</td><td>likely neutral</td><td>m</td><td>NA</td><td>11.00%</td><td>14.61%</td><td></td><td>1734 R | 1326 A</td><td>43%</td><td>HiSeq</td><td>TP</td><td></td><td></td></tr>
</table>

16	17	chr16:2156850	c.7165T>C	p.Leu2389Leu	het	rs2457533	likely neutral	d	NA	15.00%	0.00%		1480 R | 130	8%	HiSeq	TP		
16	18	chr16:2156447	c.7441C>T	p.Leu2481Leu	het	rs2003782	likely neutral	m	NA	21.00%	20.68%		1308 R | 1080 A	45%	HiSeq	TP		
16	20	chr16:2156021	c.7708T>C	p.Leu2570Leu	het	rs28575767	likely neutral	m	NA	15.00%	0.00%		1889 R | 1095 A	37%	HiSeq	TP		
16	21	chr16:2155297	c.8016+26T>C	NA	het	rs9934488	likely neutral	m	NA	20.00%	0.00%		1979 R | 1279 A	39%	HiSeq	TP		
16	21	chr16:2155426	c.7913A>G	p.His2638Arg	het	rs9936785	likely neutral	d	NA	14.00%	0.00%		1945 R | 381 A	16%	HiSeq	TP		
16	22	chr16:2154478	c.8161+21T>C	NA	hom	rs4786209	likely neutral	m	NA	56.00%	0.00%		28 R | 1079 A	97%	HiSeq	TP		
16	25	chr16:2152387	c.9196T>C	p.Phe3066Leu	het	rs77028972	likely neutral	m	NA	19.00%	16.13%		1749 R | 1477 A	46%	HiSeq	TP		
16	25	chr16:2152388	c.9195G>C	p.Val3065Val	het	rs9935834	NA	m	NA	19.00%	16.44%		1745 R | 1486 A	46%	HiSeq	TP		
16	25	chr16:2152651	c.8949-17A>G	NA	het	rs9928278	likely neutral	m	NA	21.00%	27.84%		1667 R | 1398 A	46%	HiSeq	TP		
16	26	chr16:2152129	c.9330T>C	p.Pro3110Pro	het	rs144582212	likely neutral	d	NA	11.00%	0.00%		1734 R | 340 A	16%	HiSeq	TP		
16	39	chr16:2142942	c.11153+13G>A	NA	het	rs142616270	likely neutral	m		1.00%	0.85%		1573 R | 1375 A	47%	HiSeq	TP		
16	40	chr16:g.2142119_2142114	c.11340_11345delTTACGA	p.Tyr3781_Asp3782del	het	NA	likely pathogenic	m	19686598	0.00%	0.00%	mutation	1342 R | 898 A	40%	HiSeq	TP		
16	42	chr16:2141396	c.11712+28G>C	NA	het	rs11866494	likely neutral	m	NA	26.00%	0.00%		98 R | 56 A	36%	HiSeq	TP		
16	44	chr16:2140680	c.12133A>G	p.Ile4045Val	het	rs10960	likely neutral	m	NA	24.00%	31.10%		2033 R | 1669 A	45%	HiSeq	TP		
16	45	chr16:2140454	c.12276A>G	p.Ala4092Ala	het	rs3087632	likely neutral	m	NA	26.00%	32.61%		1525 R | 1246 A	45%	HiSeq	TP		
16	46	chr16:2140010	c.12630T>C	p.Pro4210Pro	het	rs7203729	likely neutral	m	NA	26.00%	33.03%		1802 R | 1379 A	43%	HiSeq	TP	134	
17	5	chr16:2167874	c.1119C>T	p.Leu373Leu	hom	rs199685642	likely neutral	d	NA	0.00%	0.00%		4231 R | 2060 A	33%	HiSeq	TP		zygosity
17	9	chr16:2165965_2165959	c.1849+28_1849+34del	NA	het	rs56173969	likely neutral	m	NA	9.00%	0.00%		214 R | 709 A	77%	HiSeq	TP		
17	10	chr16:2165395	c.2081C>T	p.Pro694Leu	NA	rs138575342	NA	p	NA	0.00%	0.00%		2652 R | 729 A	22%	HiSeq	FP		
17	10	chr16:2165630	c.1850-4A>G	NA	het	rs35929659	likely neutral	m	NA	21.00%	22.78%		1644 R | 1484 A	47%	HiSeq	TP		
17	11	chr16:2164211	c.2813C>T	p.Thr938Met	NA	rs148709380	NA	p	NA	5.00%	0.00%		3339 R | 925 A	22%	HiSeq	FP		
17	11	chr16:2164294	c.2730C>T	p.Asp910Asp	het	rs35965348	likely neutral	m	NA	3.00%	5.33%		2269 R | 1809 A	44%	HiSeq	TP		
17	11	chr16:2164324	c.2700G>A	p.Pro900Pro	het	rs35667726	likely neutral	m	NA	3.00%	5.31%		2243 R | 1863 A	45%	HiSeq	TP		
17	11	chr16:2164808	c.2216G>A	p.Arg739Gln	hom	rs40433	likely neutral	m	NA	91.00%	0.00%		26 R | 1800 A	99%	HiSeq	TP		
17	15	chr16:2159405	c.5763G>A	p.Leu1921Leu	het	rs2575313	likely neutral	m	NA	2.00%	3.44%		1770 R | 1410 A	44%	HiSeq	TP		
17	15	chr16:2159996	c.5172C>T	p.Ala1724Ala	het	rs9935526	likely neutral	m	NA	20.00%	27.23%		2359 R | 2000 A	46%	HiSeq	TP		
17	15	chr16:2160470	c.4697_4698insA	p.Ser1567Glufs*11	het	NA	NA	m	NA	0.00%	0.00%	mutation	2402 R | 1786 A	43%	HiSeq	TP		
17	15	chr16:2160503	c.4665A>C	p.Ala1555Ala	het	rs71385734	likely neutral	m	NA	21.00%	28.26%		2335 R | 1992 A	46%	HiSeq	TP		
17	17	chr16:2156850	c.7165T>C	p.Leu2389Leu	het	rs2457533	likely neutral	d	NA	15.00%	0.00%		1873 R | 198 A	10%	HiSeq	TP		
17	18	chr16:2156447	c.7441C>T	p.Leu2481Leu	het	rs2003782	likely neutral	m	NA	21.00%	20.68%		1675 R | 1464 A	47%	HiSeq	TP		
17	21	chr16:2140554	c.8016+26T>C	NA	het	rs9934488	likely neutral	m	NA	20.00%	0.00%		2386 R | 1926 A	45%	HiSeq	TP		
17	22	chr16:2154640	c.8020C>T	p.Pro2674Ser	het	rs144557371	likely neutral	m	NA	0.00%	0.58%		2319 R | 1224 A	35%	HiSeq	TP		
17	21	chr16:2155297	c.8016+26T>C	NA	het	rs9934488	likely neutral	m	NA	20.00%	0.00%		2292 R | 1756 A	43%	HiSeq	TP		
17	22	chr16:2154478	c.8161+21T>C	NA	hom	rs4786209	likely neutral	m	NA	56.00%	0.00%		24 R | 1272 A	98%	HiSeq	TP		
17	25	chr16:2152387	c.9196T>C	p.Phe3066Leu	het	rs77028972	likely neutral	m	NA	19.00%	16.13%		2150 R | 1733 A	45%	HiSeq	TP		
17	25	chr16:2152388	c.9195G>C	p.Val3065Val	het	rs9935834	NA	m	NA	19.00%	16.44%		2150 R | 1740 A	45%	HiSeq	TP		
17	26	chr16:2152129	c.9330T>C	p.Pro3110Pro	het	rs144582212	likely neutral	d	NA	11.00%	0.00%		2037 R | 433 A	18%	HiSeq	TP		
17	25	chr16:2152651	c.8949-17A>G	NA	het	rs9928278	likely neutral	m	NA	21.00%	27.84%		1834 R | 1670 A	48%	HiSeq	TP		
17	28	chr16:2150323	c.9569-13T>C	NA	het	rs11248911	likely neutral	m	NA	11.00%	0.00%		2182 R | 1828 A	46%	HiSeq	TP		
17	33	chr16:2147421	c.10304G>A	p.Arg3435Gln	het	rs140189010	likely neutral	m	NA	0.05%	0.49%		2256 R | 1815 A	45%	HiSeq	TP		
17	35	chr16:2144176	c.10535C>T	p.Ala3512Val	het	rs34197769	likely neutral	m	NA	7.00%	9.61%		1515 R | 1343 A	47%	HiSeq	TP		
17	42	chr16:2141395	c.11712+29C>T	NA	het	NA	likely neutral	m	NA	0.00%	0.00%		198 R | 94 A	32%	HiSeq	TP		
17	42	chr16:2141396	c.11712+28G>C	NA	hom	rs11866494	likely neutral	m	NA	26.00%	0.00%		200 R | 94 A	32%	HiSeq	TP		
17	44	chr16:2140680	c.12133A>G	p.Ile4045Val	het	rs10960	likely neutral	m	NA	24.00%	31.10%		2335 R | 2041 A	47%	HiSeq	TP		
17	45	chr16:2140321	c.12409C>T	p.Leu4137Leu	het	rs79899502	likely neutral	m	NA	4.00%	5.84%		1244 R | 1092 A	47%	HiSeq	TP		
17	45	chr16:2140454	c.12276A>G	p.Ala4092Ala	het	rs3087632	likely neutral	m	NA	26.00%	32.61%		1702 R | 1467 A	46%	HiSeq	TP		
17	45	chr16:2140554	c.12176C>T	p.Ala4059Val	het	rs3209986	likely neutral	m	NA	4.00%	5.82%		2386 R | 1926 A	45%	HiSeq	TP		
17	46	chr16:2140010	c.12630T>C	p.Pro4210Pro	het	rs7203729	likely neutral	m	NA	26.00%	33.03%		2061 R | 1694 A	45%	HiSeq	TP	130	
18	5	chr16:2167874	c.1119C>T	p.Leu373Leu	hom	rs199685642	likely neutral	d	NA	0.00%	0.00%		2908 R | 1764 A	38%	HiSeq	TP		zygosity
18	11	chr16:2164808	c.2216G>A	p.Arg739Gln	hom	rs40433	likely neutral	m	NA	91.00%	0.00%		22 R | 1801 A	99%	HiSeq	TP		
18	13	chr16:2162839	c.3111A>G	p.Leu1037Leu	het	rs2099534	likely neutral	m	NA	2.00%	4.13%		2247 R | 1686 A	43%	HiSeq	TP		
18	15	chr16:2160494	c.4674G>A	p.Thr1558Thr	het	rs79884128	likely neutral	m	NA	8.00%	5.41%		2264 R | 1804 A	44%	HiSeq	TP		
18	22	chr16:2154478	c.8161+21T>C	NA	hom	rs4786209	likely neutral	m	NA	56.00%	0.00%		36 R | 990 A	96%	HiSeq	TP		
18	45	chr16:2140462	c.12267dupG	p.Leu4090Alafs*67	het	NA	NA	m	NA	0.00%	0.00%	mutation	1583 R | 1216 A	43%	HiSeq	TP	154	
19	5	chr16:2167874	c.1119C>T	p.Leu373Leu	hom	rs199685642	likely neutral	d	NA	0.00%	0.00%		4722 R | 1811 A	28%	HiSeq	TP		zygosity
19	9	chr16:2165965_2165959	c.1849+28_1849+34del	NA	het	rs56173969	likely neutral	m	NA	9.00%	0.00%		119 R | 496 A	81%	HiSeq	TP		
19	10	chr16:2165630	c.1850-4A>G	NA	het	rs35929659	likely neutral	m	NA	21.00%	22.78%		1463 R | 1132 A	44%	HiSeq	TP		
19	11	chr16:2164294	c.2730C>T	p.Asp910Asp	het	rs35965348	likely neutral	m	NA	3.00%	5.33%		2155 R | 1579 A	42%	HiSeq	TP		
19	11	chr16:2164324	c.2700G>A	p.Pro900Pro	het	rs35667726	likely neutral	m	NA	3.00%	5.31%		2234 R | 1677 A	43%	HiSeq	TP		
19	11	chr16:2164808	c.2216G>A	p.Arg739Gln	hom	rs40433	likely neutral	m	NA	91.00%	0.00%		35 R | 1627 A	98%	HiSeq	TP		
19	15	chr16:2159996	c.5172C>T	p.Ala1724Ala	het	rs9935526	likely neutral	m	NA	20.00%	27.23%		2214 R | 1864 A	46%	HiSeq	TP		
19	15	chr16:2160494	c.4674G>A	p.Thr1558Thr	het	rs79884128	likely neutral	m	NA	8.00%	5.41%		2408 R | 1921 A	44%	HiSeq	TP		
19	15	chr16:2160503	c.4665A>C	p.Ala1555Ala	het	rs71385734	likely neutral	m	NA	21.00%	28.26%		2351 R | 1977 A	46%	HiSeq	TP		
19	15	chr16:2161466	c.3702G>A	p.Ala1234Ala	het	NA	NA	m	NA	0.00%	0.00%		2831 R | 2046 A	42%	HiSeq	TP		
19	16	chr16:2157948	c.6994_7000dupGCTGGC	p.Val2334Glyfs*88	het	NA	definitely pathogenic	m	11115377	0.00%	0.00%	mutation	1444 R | 858 A	37%	HiSeq	TP		
19	17	chr16:2156850	c.7165T>C	p.Leu2389Leu	het	rs2457533	likely neutral	d	NA	15.00%	0.00%		1702 R | 156 A	8%	HiSeq	TP		
19	18	chr16:2156447	c.7441C>T	p.Leu2481Leu	het	rs2003782	likely neutral	m	NA	21.00%	20.68%		1431 R | 1023 A	42%	HiSeq	TP		
19	21	chr16:2155297	c.8016+26T>C	NA	het	rs9934488	likely neutral	m	NA	20.00%	0.00%		2520 R | 1728 A	41%	HiSeq	TP		
19	22	chr16:2154478	c.8161+21T>C	NA	hom	rs4786209	likely neutral	m	NA	56.00%	0.00%		44 R | 1210 A	96%	HiSeq	TP		
19	25	chr16:2152387	c.9196T>C	p.Phe3066Leu	het	rs77028972	likely neutral	m	NA	19.00%	16.13%		2309 R | 1483 A	39%	HiSeq	TP		
19	25	chr16:2152388	c.9195G>C	p.Val3065Val	hom	rs9935834	NA	m	NA	19.00%	16.44%		2325 R | 1492 A	39%	HiSeq	TP		
19	25	chr16:2152651	c.8949-17A>G	NA	het	rs9928278	likely neutral	m	NA	21.00%	27.84%		1778 R | 1492 A	46%	HiSeq	TP		
19	26	chr16:2152129	c.9330T>C	p.Pro3110Pro	het	rs144582212	likely neutral	d	NA	11.00%	0.00%		1941 R | 341 A	15%	HiSeq	TP		
19	27	chr16:2150466	c.9499A>T	p.Ile3167Phe	het	rs139945204	likely neutral	m	11967008	0.00%	0.07%		2575 R | 1770 A	41%	HiSeq	TP		
19	28	chr16:2150323	c.9569-13T>C	NA	het	rs11248911	likely neutral	m	NA	11.00%	0.00%		2117 R | 1561 A	42%	HiSeq	TP		
19	35	chr16:2144176	c.10535C>T	p.Ala3512Val	het	rs34197769	likely neutral	m	NA	7.00%	9.61%		1073 R | 1006 A	48%	HiSeq	TP		
19	42	chr16:2141396	c.11712+28G>C	NA	het	rs11866494	likely neutral	m	NA	26.00%	0.00%		237 R | 81 A	25%	HiSeq	TP		
19	44	chr16:2140680	c.12133A>G	p.Ile4045Val	het	rs10960	likely neutral	m	NA	24.00%	31.10%		2037 R | 1861 A	48%	HiSeq	TP		
19	45	chr16:2140321	c.12409C>T	p.Leu4137Leu	het	rs79899502	likely neutral	m	NA	4.00%	5.84%		942 R | 752 A	44%	HiSeq	TP		
19	45	chr16:2140454	c.12276A>G	p.Ala4092Ala	het	rs3087632	likely neutral	m	NA	26.00%	32.61%		1510 R | 1068 A	41%	HiSeq	TP		

<table>
<tr><td>19</td><td>45</td><td>chr16:2140554</td><td>c.12176C>T</td><td>p.Ala4059Val</td><td>het</td><td>rs3209986</td><td>likely neutral</td><td>m</td><td>NA</td><td>4.00%</td><td>5.82%</td><td></td><td>2316 R | 1706 A</td><td>42%</td><td>HiSeq</td><td>TP</td><td></td><td></td></tr>
<tr><td>19</td><td>46</td><td>chr16:2140010</td><td>c.12630T>C</td><td>p.Pro4210Pro</td><td>het</td><td>rs7203729</td><td>likely neutral</td><td>m</td><td>NA</td><td>26.00%</td><td>33.03%</td><td></td><td>1948 R | 1468 A</td><td>43%</td><td>HiSeq</td><td>TP</td><td>132</td><td></td></tr>
<tr><td>20</td><td>5</td><td>chr16:2167874</td><td>c.1119C>T</td><td>p.Leu373Leu</td><td>het</td><td>rs199685642</td><td>likely neutral</td><td>d</td><td>NA</td><td>0.00%</td><td>0.00%</td><td></td><td>1823 R | 993 A</td><td>35%</td><td>HiSeq</td><td>TP</td><td></td><td>zygosity</td></tr>
<tr><td>20</td><td>8</td><td>chr16:2166672</td><td>c.1607-27C>T</td><td>NA</td><td>het</td><td>rs4787158</td><td>likely neutral</td><td>m</td><td>NA</td><td>4.00%</td><td>2.87%</td><td></td><td>478 R | 489 A</td><td>51%</td><td>HiSeq</td><td>TP</td><td></td><td></td></tr>
<tr><td>20</td><td>9</td><td>chr16:2166079</td><td>c.1763delT</td><td>p.Phe588Serfs*197</td><td>het</td><td>NA</td><td>NA</td><td>m</td><td>NA</td><td>0.00%</td><td>0.00%</td><td>mutation</td><td>342 R | 311 A</td><td>48%</td><td>HiSeq</td><td>TP</td><td></td><td></td></tr>
<tr><td>20</td><td>10</td><td>chr16:2165520</td><td>c.1956C>T</td><td>p.Ile652Ile</td><td>het</td><td>NA</td><td>NA</td><td>m</td><td>NA</td><td>0.00%</td><td>0.00%</td><td></td><td>907 R | 718 A</td><td>44%</td><td>HiSeq</td><td>TP</td><td></td><td></td></tr>
<tr><td>20</td><td>11</td><td>chr16:2164808</td><td>c.2216G>A</td><td>p.Arg739Gln</td><td>hom</td><td>rs40433</td><td>likely neutral</td><td>m</td><td>NA</td><td>91.00%</td><td>0.00%</td><td></td><td>2 R | 888 A</td><td>100%</td><td>HiSeq</td><td>TP</td><td></td><td></td></tr>
<tr><td>20</td><td>14</td><td>chr16:2162979</td><td>c.2986-15C>T</td><td>NA</td><td>het</td><td>rs2855349</td><td>likely neutral</td><td>m</td><td>NA</td><td>7.00%</td><td>1.72%</td><td></td><td>1495 R | 1120 A</td><td>43%</td><td>HiSeq</td><td>TP</td><td></td><td></td></tr>
<tr><td>20</td><td>15</td><td>chr16:2158260</td><td>c.6908C>T</td><td>p.Ser2303Leu</td><td>het</td><td>rs143021666</td><td>NA</td><td>m</td><td>NA</td><td>0.00%</td><td>0.01%</td><td>mutation?</td><td>1354 R | 962 A</td><td>42%</td><td>HiSeq</td><td>TP</td><td></td><td></td></tr>
<tr><td>20</td><td>22</td><td>chr16:2154478</td><td>c.8161+21T>C</td><td>NA</td><td>het</td><td>rs4786209</td><td>likely neutral</td><td>m</td><td>NA</td><td>56.00%</td><td>0.00%</td><td></td><td>308 R | 362 A</td><td>54%</td><td>HiSeq</td><td>TP</td><td></td><td></td></tr>
<tr><td>20</td><td>26</td><td>chr16:2152189</td><td>c.9270C>T</td><td>p.Val3090Val</td><td>het</td><td>rs149056734</td><td>likely neutral</td><td>m</td><td>NA</td><td>0.00%</td><td>0.66%</td><td></td><td>1521 R | 908 A</td><td>37%</td><td>HiSeq</td><td>TP</td><td></td><td></td></tr>
<tr><td>20</td><td>35</td><td>chr16:2144182</td><td>c.10529C>T</td><td>p.Thr3510Met</td><td>het</td><td>rs45478794</td><td>likely neutral</td><td>m</td><td>11558899</td><td>2.00%</td><td>0.52%</td><td></td><td>886 R | 767 A</td><td>46%</td><td>HiSeq</td><td>TP</td><td>150</td><td></td></tr>
<tr><td>21</td><td>5</td><td>chr16:2167874</td><td>c.1119C>T</td><td>p.Leu373Leu</td><td>het</td><td>rs199685642</td><td>likely neutral</td><td>d</td><td>NA</td><td>0.00%</td><td>0.00%</td><td></td><td>3135 R | 1078 A</td><td>26%</td><td>HiSeq</td><td>TP</td><td></td><td>zygosity</td></tr>
<tr><td>21</td><td>10</td><td>chr16:2165395</td><td>c.2081C>T</td><td>p.Pro694Leu</td><td>NA</td><td>rs138575342</td><td>NA</td><td>p</td><td>NA</td><td>0.00%</td><td>0.00%</td><td></td><td>1658 R | 419 A</td><td>20%</td><td>HiSeq</td><td>FP</td><td></td><td></td></tr>
<tr><td>21</td><td>11</td><td>chr16:2164211</td><td>c.2813C>T</td><td>p.Thr938Met</td><td>NA</td><td>rs148709380</td><td>NA</td><td>p</td><td>NA</td><td>5.00%</td><td>0.00%</td><td></td><td>1973 R | 689 A</td><td>26%</td><td>HiSeq</td><td>FP</td><td></td><td></td></tr>
<tr><td>21</td><td>11</td><td>chr16:2164808</td><td>c.2216G>A</td><td>p.Arg739Gln</td><td>hom</td><td>rs40433</td><td>likely neutral</td><td>m</td><td>NA</td><td>91.00%</td><td>0.00%</td><td></td><td>15 R | 565 A</td><td>97%</td><td>HiSeq</td><td>TP</td><td></td><td></td></tr>
<tr><td>21</td><td>11</td><td>chr16:2164844</td><td>c.2180T>C</td><td>p.Leu727Pro</td><td>het</td><td>NA</td><td>NA</td><td>n</td><td>17582161</td><td>0.00%</td><td>0.00%</td><td>mutation?</td><td>NA</td><td>NA</td><td>HiSeq</td><td>FN</td><td></td><td>not detected, filtered out during variant calling (see comment 1)</td></tr>
<tr><td>21</td><td>22</td><td>chr16:2154478</td><td>c.8161+21T>C</td><td>NA</td><td>het</td><td>rs4786209</td><td>likely neutral</td><td>m</td><td>NA</td><td>56.00%</td><td>0.00%</td><td></td><td>345 R | 371 A</td><td>52%</td><td>HiSeq</td><td>TP</td><td></td><td></td></tr>
<tr><td>21</td><td>36</td><td>chr16:2143865</td><td>c.10768C>T</td><td>p.Leu3590Leu</td><td>hom</td><td>rs116114803</td><td>likely neutral</td><td>m</td><td>NA</td><td>1.00%</td><td>2.45%</td><td></td><td>178 R | 1664 A</td><td>90%</td><td>HiSeq</td><td>TP</td><td></td><td></td></tr>
<tr><td>21</td><td>43</td><td>chr16:2140972</td><td>c.11916C>T</td><td>p.Arg3972Arg</td><td>hom</td><td>rs77634115</td><td>likely neutral</td><td>m</td><td>NA</td><td>1.00%</td><td>2.04%</td><td></td><td>165 R | 1309 A</td><td>89%</td><td>HiSeq</td><td>TP</td><td></td><td></td></tr>
<tr><td>21</td><td>45</td><td>chr16:2140262</td><td>c.12444+23dupG</td><td>NA</td><td>het</td><td>NA</td><td>likely neutral</td><td>m</td><td>NA</td><td>0.00%</td><td>0.00%</td><td></td><td>600 R | 499 A</td><td>45%</td><td>HiSeq</td><td>TP</td><td></td><td></td></tr>
<tr><td>21</td><td>44</td><td>chr16:2140653</td><td>c.12138+22delG</td><td>NA</td><td>hom</td><td>rs199701927</td><td>likely neutral</td><td>m</td><td>NA</td><td>1.00%</td><td>2.84%</td><td></td><td>311 R | 2202 A</td><td>88%</td><td>HiSeq</td><td>TP</td><td>153</td><td></td></tr>
<tr><td>22</td><td>1</td><td>chr16:2185584</td><td>c.107C>A</td><td>p.Pro36His</td><td>het</td><td>NA</td><td>likely neutral</td><td>m</td><td>NA</td><td>0.00%</td><td>0.00%</td><td></td><td>28 R | 21 A</td><td>43%</td><td>HiSeq</td><td>TP</td><td></td><td></td></tr>
<tr><td>22</td><td>5</td><td>chr16:2167874</td><td>c.1119C>T</td><td>p.Leu373Leu</td><td>hom</td><td>rs199685642</td><td>likely neutral</td><td>d</td><td>NA</td><td>0.00%</td><td>0.00%</td><td></td><td>2486 R | 1850 A</td><td>43%</td><td>HiSeq</td><td>TP</td><td></td><td>zygosity</td></tr>
<tr><td>22</td><td>11</td><td>chr16:2164808</td><td>c.2216G>A</td><td>p.Arg739Gln</td><td>hom</td><td>rs40433</td><td>likely neutral</td><td>m</td><td>NA</td><td>91.00%</td><td>0.00%</td><td></td><td>32 R | 1842 A</td><td>98%</td><td>HiSeq</td><td>TP</td><td></td><td></td></tr>
<tr><td>22</td><td>10</td><td>chr16:2165630</td><td>c.1850-4A>G</td><td>NA</td><td>het</td><td>rs35929659</td><td>likely neutral</td><td>m</td><td>NA</td><td>21.00%</td><td>22.78%</td><td></td><td>1617 R | 1379 A</td><td>46%</td><td>HiSeq</td><td>TP</td><td></td><td></td></tr>
<tr><td>22</td><td>13</td><td>chr16:2162887</td><td>c.3063T>C</td><td>p.Gly1021Gly</td><td>het</td><td>rs2369068</td><td>likely neutral</td><td>m</td><td>NA</td><td>16.00%</td><td>22.58%</td><td></td><td>2513 R | 1999 A</td><td>44%</td><td>HiSeq</td><td>TP</td><td></td><td></td></tr>
<tr><td>22</td><td>14</td><td>chr16:2162361</td><td>c.3275T>C</td><td>p.Met1092Thr</td><td>het</td><td>rs2549677</td><td>likely neutral</td><td>m</td><td>NA</td><td>17.00%</td><td>0.00%</td><td></td><td>1654 R | 1058 A</td><td>39%</td><td>HiSeq</td><td>TP</td><td></td><td></td></tr>
<tr><td>22</td><td>15</td><td>chr16:2159996</td><td>c.5172C>T</td><td>p.Ala1724Ala</td><td>het</td><td>rs9935526</td><td>likely neutral</td><td>m</td><td>NA</td><td>20.00%</td><td>27.23%</td><td></td><td>2068 R | 1779 A</td><td>46%</td><td>HiSeq</td><td>TP</td><td></td><td></td></tr>
<tr><td>22</td><td>15</td><td>chr16:2160503</td><td>c.4665A>C</td><td>p.Ala1555Ala</td><td>het</td><td>rs71385734</td><td>likely neutral</td><td>m</td><td>NA</td><td>21.00%</td><td>28.26%</td><td></td><td>2155 R | 1816 A</td><td>46%</td><td>HiSeq</td><td>TP</td><td></td><td></td></tr>
<tr><td>22</td><td>15</td><td>chr16:2160973</td><td>c.4195T>C</td><td>p.Trp1399Arg</td><td>het</td><td>rs116092985</td><td>likely neutral</td><td>m</td><td>NA</td><td>5.00%</td><td>0.00%</td><td></td><td>2288 R | 1567 A</td><td>41%</td><td>HiSeq</td><td>TP</td><td></td><td></td></tr>
<tr><td>22</td><td>15</td><td>chr16:2161793</td><td>c.4018C>T</td><td>p.Arg1340Trp</td><td>het</td><td>rs143690392</td><td>likely neutral</td><td>m</td><td>11967008</td><td>0.14%</td><td>0.31%</td><td></td><td>2484 R | 1824 A</td><td>42%</td><td>HiSeq</td><td>TP</td><td></td><td></td></tr>
<tr><td>22</td><td>15</td><td>chr16:2161796</td><td>c.3375C>T</td><td>p.Ser1125Ser</td><td>het</td><td>rs74331768</td><td>likely neutral</td><td>m</td><td>NA</td><td>11.00%</td><td>14.45%</td><td></td><td>1820 R | 1375 A</td><td>43%</td><td>HiSeq</td><td>TP</td><td></td><td></td></tr>
<tr><td>22</td><td>15</td><td>chr16:2140564</td><td>c.3372C>T</td><td>p.Ala1124Ala</td><td>het</td><td>rs75510884</td><td>likely neutral</td><td>m</td><td>NA</td><td>11.00%</td><td>14.61%</td><td></td><td>1791 R | 1374 A</td><td>43%</td><td>HiSeq</td><td>TP</td><td></td><td></td></tr>
<tr><td>22</td><td>15</td><td>chr16:2140565</td><td>c.3296-15G>A</td><td>NA</td><td>het</td><td>rs201704201</td><td>likely neutral</td><td>m</td><td>NA</td><td>0.00%</td><td>0.43%</td><td></td><td>1530 R | 1243 A</td><td>45%</td><td>HiSeq</td><td>TP</td><td></td><td></td></tr>
<tr><td>22</td><td>17</td><td>chr16:2156850</td><td>c.7165T>C</td><td>p.Leu2389Leu</td><td>het</td><td>rs2457533</td><td>likely neutral</td><td>d</td><td>NA</td><td>15.00%</td><td>0.00%</td><td></td><td>1723 R | 257 A</td><td>13%</td><td>HiSeq</td><td>TP</td><td></td><td></td></tr>
<tr><td>22</td><td>18</td><td>chr16:2156447</td><td>c.7441C>T</td><td>p.Leu2481Leu</td><td>het</td><td>rs2003782</td><td>likely neutral</td><td>m</td><td>NA</td><td>21.00%</td><td>20.68%</td><td></td><td>1714 R | 1319 A</td><td>43%</td><td>HiSeq</td><td>TP</td><td></td><td></td></tr>
<tr><td>22</td><td>20</td><td>chr16:2156021</td><td>c.7708T>C</td><td>p.Leu2570Leu</td><td>het</td><td>rs28575767</td><td>likely neutral</td><td>m</td><td>NA</td><td>15.00%</td><td>0.00%</td><td></td><td>1902 R | 1148 A</td><td>38%</td><td>HiSeq</td><td>TP</td><td></td><td></td></tr>
<tr><td>22</td><td>21</td><td>chr16:2155426</td><td>c.7913A>G</td><td>p.His2638Arg</td><td>het</td><td>rs9936785</td><td>likely neutral</td><td>m</td><td>NA</td><td>14.00%</td><td>0.00%</td><td></td><td>1927 R | 526 A</td><td>21%</td><td>HiSeq</td><td>TP</td><td></td><td></td></tr>
<tr><td>22</td><td>21</td><td>chr16:2155297</td><td>c.8016+26T>C</td><td>NA</td><td>het</td><td>rs9934488</td><td>likely neutral</td><td>m</td><td>NA</td><td>20.00%</td><td>0.00%</td><td></td><td>2040 R | 1327 A</td><td>39%</td><td>HiSeq</td><td>TP</td><td></td><td></td></tr>
<tr><td>22</td><td>23</td><td>chr16:2153440</td><td>c.8618C>T</td><td>p.Thr2873Ile</td><td>het</td><td>rs148378229</td><td>NA</td><td>m</td><td>NA</td><td>0.00%</td><td>0.03%</td><td></td><td>1705 R | 1335 A</td><td>44%</td><td>HiSeq</td><td>TP</td><td></td><td></td></tr>
<tr><td>22</td><td>22</td><td>chr16:2154478</td><td>c.8161+21T>C</td><td>NA</td><td>hom</td><td>rs4786209</td><td>likely neutral</td><td>m</td><td>NA</td><td>56.00%</td><td>0.00%</td><td></td><td>31 R | 1254 A</td><td>98%</td><td>HiSeq</td><td>TP</td><td></td><td></td></tr>
<tr><td>22</td><td>25</td><td>chr16:2152387</td><td>c.9196T>C</td><td>p.Phe3066Leu</td><td>het</td><td>rs77028972</td><td>likely neutral</td><td>m</td><td>NA</td><td>19.00%</td><td>16.13%</td><td></td><td>1800 R | 1626 A</td><td>47%</td><td>HiSeq</td><td>TP</td><td></td><td></td></tr>
<tr><td>22</td><td>25</td><td>chr16:2152388</td><td>c.9195G>C</td><td>p.Val3065Val</td><td>het</td><td>rs9935834</td><td>NA</td><td>m</td><td>NA</td><td>19.00%</td><td>16.44%</td><td></td><td>1801 R | 1632 A</td><td>48%</td><td>HiSeq</td><td>TP</td><td></td><td></td></tr>
<tr><td>22</td><td>26</td><td>chr16:2152129</td><td>c.9330T>C</td><td>p.Pro3110Pro</td><td>het</td><td>rs144582212</td><td>likely neutral</td><td>d</td><td>NA</td><td>11.00%</td><td>0.00%</td><td></td><td>1912 R | 400 A</td><td>17%</td><td>HiSeq</td><td>TP</td><td></td><td></td></tr>
<tr><td>22</td><td>25</td><td>chr16:2152651</td><td>c.8949-17A>G</td><td>NA</td><td>het</td><td>rs9928278</td><td>likely neutral</td><td>m</td><td>NA</td><td>21.00%</td><td>27.84%</td><td></td><td>1720 R | 1602 A</td><td>48%</td><td>HiSeq</td><td>TP</td><td></td><td></td></tr>
<tr><td>22</td><td>36</td><td>chr16:2143829</td><td>c.10804G>C</td><td>p.Gly3602Arg</td><td>het</td><td>NA</td><td>NA</td><td>m</td><td>NA</td><td>0.00%</td><td>0.00%</td><td>mutation?</td><td>1589 R | 1256 A</td><td>44%</td><td>HiSeq</td><td>TP</td><td></td><td></td></tr>
<tr><td>22</td><td>42</td><td>chr16:2141396</td><td>c.11712+28G>C</td><td>NA</td><td>het</td><td>rs11866494</td><td>likely neutral</td><td>m</td><td>NA</td><td>26.00%</td><td>0.00%</td><td></td><td>316 R | 186 A</td><td>37%</td><td>HiSeq</td><td>TP</td><td></td><td></td></tr>
<tr><td>22</td><td>44</td><td>chr16:2140680</td><td>c.12133A>G</td><td>p.Ile4045Val</td><td>het</td><td>rs10960</td><td>likely neutral</td><td>m</td><td>NA</td><td>24.00%</td><td>31.10%</td><td></td><td>2193 R | 1816 A</td><td>45%</td><td>HiSeq</td><td>TP</td><td></td><td></td></tr>
<tr><td>22</td><td>45</td><td>chr16:2140454</td><td>c.12276A>G</td><td>p.Ala4092Ala</td><td>het</td><td>rs3087632</td><td>likely neutral</td><td>m</td><td>NA</td><td>26.00%</td><td>32.61%</td><td></td><td>1689 R | 1620 A</td><td>49%</td><td>HiSeq</td><td>TP</td><td></td><td></td></tr>
<tr><td>22</td><td>46</td><td>chr16:2140010</td><td>c.12630T>C</td><td>p.Pro4210Pro</td><td>het</td><td>rs7203729</td><td>likely neutral</td><td>m</td><td>NA</td><td>26.00%</td><td>33.03%</td><td></td><td>1789 R | 1418 A</td><td>44%</td><td>HiSeq</td><td>TP</td><td>131</td><td></td></tr>
<tr><td>23</td><td>5</td><td>chr16:2167874</td><td>c.1119C>T</td><td>p.Leu373Leu</td><td>het</td><td>rs199685642</td><td>likely neutral</td><td>m</td><td>NA</td><td>0.00%</td><td>0.00%</td><td></td><td>1314 R | 1281 A</td><td>49%</td><td>HiSeq</td><td>TP</td><td></td><td></td></tr>
<tr><td>23</td><td>10</td><td>chr16:2165630</td><td>c.1850-4A>G</td><td>NA</td><td>hom</td><td>rs35929659</td><td>likely neutral</td><td>m</td><td>NA</td><td>21.00%</td><td>22.78%</td><td></td><td>134 R | 1694 A</td><td>93%</td><td>HiSeq</td><td>TP</td><td></td><td></td></tr>
<tr><td>23</td><td>11</td><td>chr16:2164808</td><td>c.2216G>A</td><td>p.Arg739Gln</td><td>hom</td><td>rs40433</td><td>likely neutral</td><td>m</td><td>NA</td><td>91.00%</td><td>0.00%</td><td></td><td>17 R | 1275 A</td><td>99%</td><td>HiSeq</td><td>TP</td><td></td><td></td></tr>
<tr><td>23</td><td>11</td><td>chr16:2164925_2164924</td><td>c.2099_2100delinsAA</td><td>p.Val700Glu</td><td>het</td><td>NA</td><td>NA</td><td>m</td><td>NA</td><td>0.00%</td><td>0.00%</td><td>mutation?</td><td>2311 R | 1077 A</td><td>32%</td><td>HiSeq</td><td>TP</td><td></td><td></td></tr>
<tr><td>23</td><td>13</td><td>chr16:2162887</td><td>c.3063T>C</td><td>p.Gly1021Gly</td><td>hom</td><td>rs2369068</td><td>likely neutral</td><td>m</td><td>NA</td><td>16.00%</td><td>22.58%</td><td></td><td>446 R | 2828 A</td><td>86%</td><td>HiSeq</td><td>TP</td><td></td><td></td></tr>
<tr><td>23</td><td>14</td><td>chr16:2162361</td><td>c.3275T>C</td><td>p.Met1092Thr</td><td>hom</td><td>rs2549677</td><td>likely neutral</td><td>m</td><td>NA</td><td>17.00%</td><td>0.00%</td><td></td><td>157 R | 1251 A</td><td>89%</td><td>HiSeq</td><td>TP</td><td></td><td></td></tr>
<tr><td>23</td><td>15</td><td>chr16:2159996</td><td>c.5172C>T</td><td>p.Ala1724Ala</td><td>het</td><td>rs9935526</td><td>likely neutral</td><td>m</td><td>NA</td><td>20.00%</td><td>27.23%</td><td></td><td>212 R | 2461 A</td><td>92%</td><td>HiSeq</td><td>TP</td><td></td><td></td></tr>
<tr><td>23</td><td>15</td><td>chr16:2160503</td><td>c.4665A>C</td><td>p.Ala1555Ala</td><td>het</td><td>rs71385734</td><td>likely neutral</td><td>m</td><td>NA</td><td>21.00%</td><td>28.26%</td><td></td><td>238 R | 2722 A</td><td>92%</td><td>HiSeq</td><td>TP</td><td></td><td></td></tr>
<tr><td>23</td><td>15</td><td>chr16:2160866</td><td>c.4302C>T</td><td>p.Ile1434Ile</td><td>het</td><td>rs78509585</td><td>likely neutral</td><td>m</td><td>NA</td><td>1.00%</td><td>1.19%</td><td></td><td>1110 R | 1015 A</td><td>48%</td><td>HiSeq</td><td>TP</td><td></td><td></td></tr>
<tr><td>23</td><td>15</td><td>chr16:2161793</td><td>c.3375C>T</td><td>p.Ser1125Ser</td><td>hom</td><td>rs74331768</td><td>likely neutral</td><td>m</td><td>NA</td><td>11.00%</td><td>14.45%</td><td></td><td>224 R | 1877 A</td><td>89%</td><td>HiSeq</td><td>TP</td><td></td><td></td></tr>
<tr><td>23</td><td>15</td><td>chr16:2161796</td><td>c.3372C>T</td><td>p.Ala1124Ala</td><td>het</td><td>rs75510884</td><td>likely neutral</td><td>m</td><td>NA</td><td>11.00%</td><td>14.61%</td><td></td><td>223 R | 1877 A</td><td>89%</td><td>HiSeq</td><td>TP</td><td></td><td></td></tr>
<tr><td>23</td><td>17</td><td>chr16:2156850</td><td>c.7165T>C</td><td>p.Leu2389Leu</td><td>hom</td><td>rs2457533</td><td>likely neutral</td><td>d</td><td>NA</td><td>15.00%</td><td>0.00%</td><td></td><td>160 R | 361 A</td><td>69%</td><td>HiSeq</td><td>TP</td><td></td><td>zygosity</td></tr>
<tr><td>23</td><td>18</td><td>chr16:2156369</td><td>c.7489+30C>T</td><td>NA</td><td>het</td><td>rs142761413</td><td>NA</td><td>m</td><td>NA</td><td>2.00%</td><td>1.60%</td><td></td><td>1261 R | 1002 A</td><td>44%</td><td>HiSeq</td><td>TP</td><td></td><td></td></tr>
<tr><td>23</td><td>18</td><td>chr16:2156447</td><td>c.7441C>T</td><td>p.Leu2481Leu</td><td>hom</td><td>rs2003782</td><td>likely neutral</td><td>m</td><td>NA</td><td>21.00%</td><td>20.68%</td><td></td><td>149 R | 1675 A</td><td>92%</td><td>HiSeq</td><td>TP</td><td></td><td></td></tr>
<tr><td>23</td><td>20</td><td>chr16:2156021</td><td>c.7708T>C</td><td>p.Leu2570Leu</td><td>het</td><td>rs28575767</td><td>likely neutral</td><td>m</td><td>NA</td><td>15.00%</td><td>0.00%</td><td></td><td>277 R | 1624 A</td><td>85%</td><td>HiSeq</td><td>TP</td><td></td><td></td></tr>
<tr><td>23</td><td>21</td><td>chr16:2155297</td><td>c.8016+26T>C</td><td>NA</td><td>hom</td><td>rs9934488</td><td>likely neutral</td><td>m</td><td>NA</td><td>20.00%</td><td>0.00%</td><td></td><td>240 R | 2172 A</td><td>90%</td><td>HiSeq</td><td>TP</td><td></td><td></td></tr>
<tr><td>23</td><td>21</td><td>chr16:2155426</td><td>c.7913A>G</td><td>p.His2638Arg</td><td>het</td><td>rs9936785</td><td>likely neutral</td><td>m</td><td>NA</td><td>14.00%</td><td>0.00%</td><td></td><td>1383 R | 363 A</td><td>21%</td><td>HiSeq</td><td>TP</td><td></td><td></td></tr>
<tr><td>23</td><td>21</td><td>chr16:2155853</td><td>c.7863+13T>C</td><td>NA</td><td>het</td><td>rs115024665</td><td>NA</td><td>m</td><td>NA</td><td>1.00%</td><td>1.27%</td><td></td><td>1321 R | 1072 A</td><td>45%</td><td>HiSeq</td><td>TP</td><td></td><td></td></tr>
<tr><td>23</td><td>23</td><td>chr16:2153414</td><td>c.8644T>A</td><td>p.Trp2882Arg</td><td>het</td><td>rs13333553</td><td>likely neutral</td><td>m</td><td>NA</td><td>4.00%</td><td>4.43%</td><td></td><td>1232 R | 951 A</td><td>44%</td><td>HiSeq</td><td>TP</td><td></td><td></td></tr>
<tr><td>23</td><td>22</td><td>chr16:2154478</td><td>c.8161+21T>C</td><td>NA</td><td>hom</td><td>rs4786209</td><td>likely neutral</td><td>m</td><td>NA</td><td>56.00%</td><td>0.00%</td><td></td><td>10 R | 714 A</td><td>99%</td><td>HiSeq</td><td>TP</td><td></td><td></td></tr>
<tr><td>23</td><td>24</td><td>chr16:2152865</td><td>c.8898G>C</td><td>p.Glu2966Asp</td><td>het</td><td>rs13337123</td><td>likely neutral</td><td>m</td><td>'11216660</td><td>4.00%</td><td>5.32%</td><td></td><td>1091 R | 982 A</td><td>47%</td><td>HiSeq</td><td>TP</td><td></td><td></td></tr>
<tr><td>23</td><td>25</td><td>chr16:2152387</td><td>c.9196T>C</td><td>p.Phe3066Leu</td><td>het</td><td>rs77028972</td><td>likely neutral</td><td>m</td><td>NA</td><td>19.00%</td><td>16.13%</td><td></td><td>1291 R | 1142 A</td><td>47%</td><td>HiSeq</td><td>TP</td><td></td><td></td></tr>
<tr><td>23</td><td>25</td><td>chr16:2152388</td><td>c.9195G>C</td><td>p.Val3065Val</td><td>het</td><td>rs9935834</td><td>NA</td><td>m</td><td>NA</td><td>19.00%</td><td>16.44%</td><td></td><td>1091 R | 982 A</td><td>47%</td><td>HiSeq</td><td>TP</td><td></td><td></td></tr>
<tr><td>23</td><td>25</td><td>chr16:2152651</td><td>c.8949-17A>G</td><td>NA</td><td>hom</td><td>rs9928278</td><td>likely neutral</td><td>m</td><td>NA</td><td>21.00%</td><td>27.84%</td><td></td><td>154 R | 2268 A</td><td>94%</td><td>HiSeq</td><td>TP</td><td></td><td></td></tr>
<tr><td>23</td><td>26</td><td>chr16:2152129</td><td>c.9330T>C</td><td>p.Pro3110Pro</td><td>hom</td><td>rs144582212</td><td>likely neutral</td><td>d</td><td>NA</td><td>11.00%</td><td>0.00%</td><td></td><td>177 R | 536 A</td><td>75%</td><td>HiSeq</td><td>TP</td><td></td><td>zygosity</td></tr>
<tr><td>23</td><td>28</td><td>chr16:2150323</td><td>c.9569-13T>C</td><td>NA</td><td>het</td><td>rs11248911</td><td>likely neutral</td><td>m</td><td>NA</td><td>11.00%</td><td>0.00%</td><td></td><td>1484 R | 1143 A</td><td>44%</td><td>HiSeq</td><td>TP</td><td></td><td></td></tr>
<tr><td>23</td><td>33</td><td>chr16:2147500</td><td>c.10225G>C</td><td>p.Val3409Leu</td><td>het</td><td>rs61747420</td><td>likely neutral</td><td>m</td><td>NA</td><td>4.00%</td><td>4.26%</td><td></td><td>1467 R | 1255 A</td><td>46%</td><td>HiSeq</td><td>TP</td><td></td><td></td></tr>
<tr><td>23</td><td>42</td><td>chr16:2141396</td><td>c.11712+28G>C</td><td>NA</td><td>hom</td><td>rs11866494</td><td>likely neutral</td><td>d</td><td>NA</td><td>26.00%</td><td>0.00%</td><td></td><td>9 R | 23 A</td><td>72%</td><td>HiSeq</td><td>TP</td><td></td><td>zygosity; edge of coverage, coding region covered properly, no adaption of pipeline</td></tr>
<tr><td>23</td><td>43</td><td>chr16:2140927</td><td>c.11961C>T</td><td>p.Ala3987Ala</td><td>het</td><td>rs116236042</td><td>likely neutral</td><td>m</td><td>NA</td><td>2.00%</td><td>1.57%</td><td></td><td>846 R | 733 A</td><td>46%</td><td>HiSeq</td><td>TP</td><td></td><td></td></tr>
<tr><td>23</td><td>44</td><td>chr16:2140680</td><td>c.12133A>G</td><td>p.Ile4045Val</td><td>hom</td><td>rs10960</td><td>likely neutral</td><td>m</td><td>NA</td><td>24.00%</td><td>31.10%</td><td></td><td>248 R | 2493 A</td><td>91%</td><td>HiSeq</td><td>TP</td><td></td><td></td></tr>
</table>

23	45	chr16:2140454	c.12276A>G	p.Ala4092Ala	hom	rs3087632	likely neutral	m	NA	26.00%	32.61%		143 R | 2016 A	93%	HiSeq	TP		
23	46	chr16:2140010	c.12630T>C	p.Pro4210Pro	hom	rs7203729	likely neutral	m	NA	26.00%	33.03%		188 R | 1887 A	91%	HiSeq	TP	128	
24	1	chr16:2185584	c.107C>A	p.Pro36His	het	NA	likely neutral	m	NA	0.00%	0.00%		25 R | 24 A	49%	HiSeq	TP		
24	5	chr16:2167874	c.1119C>T	p.Leu373Leu	het	rs199685642	likely neutral	d	NA	0.00%	0.00%		3597 R | 1340 A	27%	HiSeq	TP		zygosity
24	10	chr16:2165395	c.2081C>T	p.Pro694Leu	NA	rs138575342	NA	p	NA	0.00%	0.00%		2063 R | 546 A	21%	HiSeq	FP		
24	11	chr16:2164211	c.2813C>T	p.Thr938Met	NA	rs148709380	NA	p	NA	5.00%	0.00%		2287 R | 1144 A	33%	HiSeq	FP		
24	11	chr16:2164808	c.2216G>A	p.Arg739Gln	hom	rs40433	likely neutral	m	NA	91.00%	0.00%		34 R | 1268 A	97%	HiSeq	TP		
24	22	chr16:2154478	c.8161+21T>C	NA	het	rs4786209	likely neutral	m	NA	56.00%	0.00%		29 R | 926 A	97%	HiSeq	TP		
24	33	chr16:2147410	c.10315C>G	p.Arg3439Gly	het	NA	NA	m	NA	0.00%	0.00%	mutation?	1764 R | 1389 A	44%	HiSeq	TP		
24	46	chr16:2140198	c.12445-3C>G	NA	het	NA	likely pathogenic	m	NA	0.00%	0.00%	mutation?	846 R | 716 A	46%	HiSeq	TP	154	
25	5	chr16:2167874	c.1119C>T	p.Leu373Leu	hom	rs199685642	likely neutral	d	NA	0.00%	0.00%		1746 R | 1702 A	49%	HiSeq	TP		zygosity
25	11	chr16:2164808	c.2216G>A	p.Arg739Gln	hom	rs40433	likely neutral	m	NA	91.00%	0.00%		27 R | 1588 A	98%	HiSeq	TP		
25	22	chr16:2154478	c.8161+21T>C	NA	hom	rs4786209	likely neutral	m	NA	56.00%	0.00%		16 R | 855 A	98%	HiSeq	TP		
25	36	chr16:2143865	c.10768C>T	p.Leu3590Leu	het	rs116114803	likely neutral	m	NA	1.00%	2.45%		1483 R | 1156 A	44%	HiSeq	TP		
25	40	chr16:2142114	c.11338_11344dup	p.Asp3782Glyfs*36	het	NA	NA	m	NA	0.00%	0.00%	mutation	1089 R | 1031 A	49%	HiSeq	TP		
25	43	chr16:2140972	c.11916C>T	p.Arg3972Arg	het	rs77634115	likely neutral	m	NA	1.00%	2.04%		856 R | 951 A	53%	HiSeq	TP		
25	45	chr16:2140262	c.12444+23dupG	NA	het	NA	likely neutral	m	NA	0.00%	0.00%		931 R | 790	46%	HiSeq	TP		
25	44	chr16:2140653	c.12138+22delG	NA	het	rs199701927	likely neutral	m	NA	1.00%	2.84%		1834 R | 1618 A	47%	HiSeq	TP	152	
26	5	chr16:2167874	c.1119C>T	p.Leu373Leu	hom	rs199685642	likely neutral	d	NA	0.00%	0.00%		3145 R | 1763 A	36%	HiSeq	TP		zygosity
26	5	chr16:2168181	c.812C>A	p.Ala271Asp	het	NA	likely pathogenic	m	17574468	0.00%	0.00%	mutation?	432 R | 345 A	44%	HiSeq	TP		
26	11	chr16:2164808	c.2216G>A	p.Arg739Gln	hom	rs40433	likely neutral	m	NA	91.00%	0.00%		28 R | 1889 A	99%	HiSeq	TP		
26	22	chr16:2154478	c.8161+21T>C	NA	het	rs4786209	likely neutral	m	NA	56.00%	0.00%		441 R | 530 A	55%	HiSeq	TP		
26	46	chr16:2139875	c.12765C>T	p.Pro4255Pro	het	rs62038811	likely neutral	m	NA	0.23%	0.47%		1143 R | 1038 A	48%	HiSeq	TP	155	
27	5	chr16:2167874	c.1119C>T	p.Leu373Leu	hom	rs199685642	likely neutral	d	NA	0.00%	0.00%		2575 R | 1665 A	39%	HiSeq	TP		zygosity
27	10	chr16:2165630	c.1850-4A>G	NA	het	rs35929659	likely neutral	m	NA	21.00%	22.78%		1168 R | 1052 A	47%	HiSeq	TP		
27	11	chr16:2164808	c.2216G>A	p.Arg739Gln	hom	rs40433	likely neutral	m	NA	91.00%	0.00%		23 R | 1642 A	99%	HiSeq	TP		
27	13	chr16:2162887	c.3063T>C	p.Gly1021Gly	het	rs2369068	likely neutral	m	NA	16.00%	22.58%		2182 R | 1685 A	44%	HiSeq	TP		
27	14	chr16:2162361	c.3275T>C	p.Met1092Thr	het	rs2549677	likely neutral	m	NA	17.00%	0.00%		1295 R | 797 A	38%	HiSeq	TP		
27	15	chr16:2159996	c.5172C>T	p.Ala1724Ala	het	rs9935526	likely neutral	m	NA	20.00%	27.23%		1786 R | 1545 A	46%	HiSeq	TP		
27	15	chr16:2160494	c.4674G>A	p.Thr1558Thr	het	rs79884128	likely neutral	m	NA	8.00%	5.41%		1835 R | 1554 A	46%	HiSeq	TP		
27	15	chr16:2160503	c.4665A>C	p.Ala1555Ala	het	rs71385734	likely neutral	m	NA	21.00%	28.26%		1763 R | 1586 A	47%	HiSeq	TP		
27	15	chr16:2160973	c.4195T>C	p.Trp1399Arg	het	rs116092985	likely neutral	m	NA	5.00%	0.00%		1875 R | 1368 A	42%	HiSeq	TP		
27	15	chr16:2161793	c.3375C>T	p.Ser1125Ser	het	rs74331768	likely neutral	m	NA	11.00%	14.45%		1473 R | 1176 A	44%	HiSeq	TP		
27	15	chr16:2161796	c.3372C>T	p.Ala1124Ala	het	rs75510884	likely neutral	m	NA	11.00%	14.61%		1462 R | 1173 A	45%	HiSeq	TP		
27	17	chr16:2156850	c.7165T>C	p.Leu2389Leu	het	rs2457533	likely neutral	d	NA	15.00%	0.00%		1333 R | 236 A	15%	HiSeq	TP		
27	18	chr16:2156447	c.7441C>T	p.Leu2481Leu	hom	rs2003782	likely neutral	m	NA	21.00%	20.68%		1229 R | 1058 A	46%	HiSeq	TP		
27	20	chr16:2155925	c.7804C>T	p.Gln2602*	het	NA	NA	m	22383692	0.00%	0.00%	mutation	2175 R | 1572 A	42%	HiSeq	TP		
27	20	chr16:2156021	c.7708T>C	p.Leu2570Leu	het	rs28575767	likely neutral	m	NA	15.00%	0.00%		1702 R | 1112 A	40%	HiSeq	TP		
27	21	chr16:2155297	c.8016+26T>C	NA	het	rs9934488	likely neutral	m	NA	20.00%	0.00%		1811 R | 1171 A	39%	HiSeq	TP		
27	21	chr16:2155426	c.7913A>G	p.His2638Arg	het	rs9936785	likely neutral	m	NA	14.00%	0.00%		1804 R | 478 A	21%	HiSeq	TP		
27	22	chr16:2154478	c.8161+21T>C	NA	het	rs4786209	likely neutral	m	NA	56.00%	0.00%		318 R | 411 A	56%	HiSeq	TP		
27	25	chr16:2152387	c.9196T>C	p.Phe3066Leu	het	rs77028972	likely neutral	m	NA	19.00%	16.13%		1569 R | 1338 A	46%	HiSeq	TP		
27	25	chr16:2152388	c.9195G>C	p.Val3065Val	het	rs9935834	NA	m	NA	19.00%	16.44%		1570 R | 1337 A	46%	HiSeq	TP		
27	25	chr16:2152651	c.8949-17A>G	NA	het	rs9928278	likely neutral	m	NA	21.00%	27.84%		1480 R | 1401 A	49%	HiSeq	TP		
27	26	chr16:2152129	c.9330T>C	p.Pro3110Pro	het	rs144582212	likely neutral	d	NA	11.00%	0.00%		1597 R | 327 A	17%	HiSeq	TP		
27	27	chr16:2150466	c.9499A>T	p.Ile3167Phe	het	rs139945204	likely neutral	m	11967008	0.00%	0.07%		2108 R | 1634 A	44%	HiSeq	TP		
27	44	chr16:2140680	c.12133A>G	p.Ile4045Val	het	rs10960	likely neutral	m	NA	24.00%	31.10%		1741 R | 1581 A	48%	HiSeq	TP		
27	45	chr16:2140454	c.12276A>G	p.Ala4092Ala	het	rs3087632	likely neutral	m	NA	26.00%	32.61%		1427 R | 1152 A	45%	HiSeq	TP		
27	46	chr16:2140010	c.12630T>C	p.Pro4210Pro	het	rs7203729	likely neutral	m	NA	26.00%	33.03%		1510 R | 1209 A	44%	HiSeq	TP	134	
28	5	chr16:2167874	c.1119C>T	p.Leu373Leu	hom	rs199685642	likely neutral	d	NA	0.00%	0.00%		4107 R | 1648 A	29%	HiSeq	TP		zygosity
28	8	chr16:2166672	c.1607-27C>T	NA	het	rs4787158	likely neutral	m	NA	4.00%	2.87%		594 R | 676 A	53%	HiSeq	TP		
28	10	chr16:2165395	c.2081C>T	p.Pro694Leu	NA	rs138575342	NA	p	NA	0.00%	0.00%		2292 R | 577 A	20%	HiSeq	FP		
28	11	chr16:2164211	c.2813C>T	p.Thr938Met	NA	rs148709380	NA	p	NA	5.00%	0.00%		2782 R | 777 A	22%	HiSeq	FP		
28	11	chr16:2164808	c.2216G>A	p.Arg739Gln	hom	rs40433	likely neutral	m	NA	91.00%	0.00%		11 R | 1654 A	99%	HiSeq	TP		
28	22	chr16:2154478	c.8161+21T>C	NA	het	rs4786209	likely neutral	m	NA	56.00%	0.00%		391 R | 565 A	59%	HiSeq	TP		
28	24	chr16:2152818	c.8945delC	p.Pro2982Argfs*12	het	NA	NA	m	NA	0.00%	0.00%	mutation	1429 R | 1236 A	46%	HiSeq	TP	155	
29	11	chr16:2164808	c.2216G>A	p.Arg739Gln	het	rs40433	likely neutral	m	NA	91.00%	0.00%		1476 R | 1372 A	48%	HiSeq	TP		
29	15	chr16:2161739	c.3425_3428dupGGCC	p.Val1144Alafs*68	het	NA	NA	m	NA	0.00%	0.00%	mutation	1486 R | 637 A	30%	HiSeq	TP		
29	22	chr16:2154478	c.8161+21T>C	NA	hom	rs4786209	likely neutral	m	NA	56.00%	0.00%		32 R | 919 A	97%	HiSeq	TP	157	
31	5	chr16:2167874	c.1119C>T	p.Leu373Leu	hom	rs199685642	likely neutral	d	NA	0.00%	0.00%		4039 R | 1834 A	31%	HiSeq	TP		zygosity
31	10	chr16:2165630	c.1850-4A>G	NA	het	rs35929659	likely neutral	m	NA	21.00%	22.78%		1507 R | 1244 A	45%	HiSeq	TP		
31	11	chr16:2164211	c.2813C>T	p.Thr938Met	NA	rs148709380	NA	p	NA	5.00%	0.00%		3167 R | 929 A	23%	HiSeq	FP		
31	11	chr16:2164808	c.2216G>A	p.Arg739Gln	hom	rs40433	likely neutral	m	NA	91.00%	0.00%		31 R | 1924 A	98%	HiSeq	TP		
31	13	chr16:2162887	c.3063T>C	p.Gly1021Gly	het	rs2369068	likely neutral	m	NA	16.00%	22.58%		2737 R | 2161 A	44%	HiSeq	TP		
31	14	chr16:.2162361	c.3275T>C	p.Met1092Thr	het	rs2549677	likely neutral	m	NA	17.00%	0.00%		1764 R | 929 A	34%	HiSeq	TP		
31	15	chr16:2159996	c.5172C>T	p.Ala1724Ala	het	rs9935526	likely neutral	m	NA	20.00%	27.23%		2201 R | 1980 A	47%	HiSeq	TP		
31	15	chr16:2160503	c.4665A>C	p.Ala1555Ala	het	rs71385734	likely neutral	m	NA	21.00%	28.26%		2285 R | 2054 A	47%	HiSeq	TP		
31	15	chr16:2160973	c.4195T>C	p.Trp1399Arg	het	rs116092985	likely neutral	m	NA	5.00%	0.00%		2547 R | 1703 A	40%	HiSeq	TP		
31	15	chr16:2161793	c.3375C>T	p.Ser1125Ser	het	rs74331768	likely neutral	m	NA	11.00%	14.45%		1832 R | 1438 A	44%	HiSeq	TP		
31	15	chr16:2161796	c.3372C>T	p.Ala1124Ala	het	rs75510884	likely neutral	m	NA	11.00%	14.61%		1803 R | 1441 A	44%	HiSeq	TP		
31	20	chr16:2156021	c.7708T>C	p.Leu2570Leu	het	rs28575767	likely neutral	m	NA	15.00%	0.00%		2362 R | 1232 A	34%	HiSeq	TP		
31	21	chr16:2155297	c.8016+26T>C	NA	het	rs9934488	likely neutral	m	NA	20.00%	0.00%		2068 R | 1478 A	42%	HiSeq	TP		
31	21	chr16:2155426	c.7913A>G	p.His2638Arg	het	rs9936785	likely neutral	d	NA	14.00%	0.00%		2264 R | 514 A	19%	HiSeq	TP		
31	25	chr16:2152387	c.9196T>C	p.Phe3066Leu	het	rs77028972	likely neutral	m	NA	19.00%	16.13%		2087 R | 1690 A	45%	HiSeq	TP		
31	25	chr16:2152388	c.9195G>C	p.Val3065Val	het	rs9935834	NA	m	NA	19.00%	16.44%		2090 R | 1693 A	45%	HiSeq	TP		
31	25	chr16:2152651	c.8949-17A>G	NA	het	rs9928278	likely neutral	m	NA	21.00%	27.84%		1854 R | 1705 A	48%	HiSeq	TP		
31	26	chr16:2152129	c.9330T>C	p.Pro3110Pro	het	rs144582212	likely neutral	d	NA	11.00%	0.00%		1971 R | 402 A	17%	HiSeq	TP		
31	36	chr16:2143865	c.10768C>T	p.Leu3590Leu	het	NA	likely neutral	m	NA	1.00%	2.45%		1612 R | 1374 A	46%	HiSeq	TP		
31	41	chr16:g.2141795_2141784	c.11524_11535del	p.Trp3842_Asn3845del	het	NA	NA	m	NA	0.00%	0.00%	mutation?	1612 R | 1073 A	40%	HiSeq	TP		
31	43	chr16:2140972	c.11916C>T	p.Arg3972Arg	het	rs77634115	likely neutral	m	NA	1.00%	2.04%		1133 R | 1199 A	51%	HiSeq	TP		
31	44	chr16:2140653	c.12138+22delG	NA	het	rs199701927	likely neutral	m	NA	1.00%	2.84%		2196 R | 1994 A	48%	HiSeq	TP	138	

32	5	chr16:2167874	c.1119C>T	p.Leu373Leu	hom	rs199685642	likely neutral	d	NA	0.00%	0.00%		1332 R | 703 A	35%	HiSeq	TP		zygosity
32	11	chr16:2164808	c.2216G>A	p.Arg739Gln	hom	rs40433	likely neutral	m	NA	91.00%	0.00%		15 R | 534 A	97%	HiSeq	TP		
32	15	chr16:2161626	c.3542A>G	p.Tyr1181Cys	het	NA	NA	m	NA	0.00%	0.00%	mutation?	698 R | 484 A	41%	HiSeq	TP		
32	22	chr16:2154478	c.8161+21T>C	NA	hom	rs4786209	likely neutral	m	NA	56.00%	0.00%		18 R | 264 A	94%	HiSeq	TP	156	
33	5	chr16:2167874	c.1119C>T	p.Leu373Leu	het	rs199685642	likely neutral	d	NA	0.00%	0.00%		5173 R | 2134 A	29%	HiSeq	TP		zygosity
33	9	chr16:2165965_2165959	c.1849+28_1849+34del	NA	het	rs56173969	likely neutral	m	NA	9.00%	0.00%		210 R | 612 A	74%	HiSeq	TP		
33	10	chr16:2165395	c.2081C>T	p.Pro694Leu	NA	rs138575342	NA	p	NA	0.00%	0.00%		2487 R | 679 A	21%	HiSeq	FP		
33	10	chr16:2165630	c.1850-4A>G	NA	het	rs35929659	likely neutral	m	NA	21.00%	22.78%		1715 R | 1494 A	47%	HiSeq	TP		
33	11	chr16:2164185	c.2839C>T	p.Gln947*	het	NA	definitely pathogenic	m	NA	0.00%	0.00%	mutation	3256 R | 1572 A	33%	HiSeq	TP		
33	11	chr16:2164211	c.2813C>T	p.Thr938Met	NA	rs148709380	NA	p	NA	5.00%	0.00%		3617 R | 1090 A	23%	HiSeq	FP		
33	11	chr16:2164294	c.2730C>T	p.Asp910Asp	het	rs35965348	likely neutral	m	NA	3.00%	5.33%		2552 R | 2118 A	45%	HiSeq	TP		
33	11	chr16:2164324	c.2700G>A	p.Pro900Pro	het	rs35667726	likely neutral	m	NA	3.00%	5.31%		2531 R | 2240 A	47%	HiSeq	TP		
33	11	chr16:2164808	c.2216G>A	p.Arg739Gln	hom	rs40433	likely neutral	m	NA	91.00%	0.00%		28 R | 2347 A	99%	HiSeq	TP		
33	15	chr16:2159405	c.5763G>A	p.Leu1921Leu	het	rs2575313	likely neutral	m	NA	2.00%	3.44%		1776 R | 1489 A	46%	HiSeq	TP		
33	15	chr16:2159996	c.5172C>T	p.Ala1724Ala	het	rs9935526	likely neutral	m	NA	20.00%	27.23%		2775 R | 2572 A	48%	HiSeq	TP		
33	15	chr16:2160503	c.4665A>C	p.Ala1555Ala	het	rs71385734	likely neutral	m	NA	21.00%	28.26%		2814 R | 2390 A	46%	HiSeq	TP		
33	15	chr16:2161655	c.3513C>G	p.Thr1171Thr	het	rs143784787	likely neutral	m	NA	1.00%	0.40%		2347 R | 2205 A	48%	HiSeq	TP		
33	17	chr16:2156850	c.7165T>C	p.Leu2389Leu	het	rs2457533	likely neutral	d	NA	15.00%	0.00%		1961 R | 254 A	11%	HiSeq	TP		
33	18	chr16:2156447	c.7441C>T	p.Leu2481Leu	het	rs2003782	likely neutral	m	NA	21.00%	20.68%		1638 R | 1311 A	44%	HiSeq	TP		
33	21	chr16:2155297	c.8016+26T>C	NA	het	rs9934488	likely neutral	m	NA	20.00%	0.00%		2648 R | 2170 A	45%	HiSeq	TP		
33	22	chr16:2154478	c.8161+21T>C	NA	hom	rs4786209	likely neutral	m	NA	56.00%	0.00%		44 R | 1430 A	97%	HiSeq	TP		
33	25	chr16:2152387	c.9196T>C	p.Phe3066Leu	het	rs77028972	likely neutral	m	NA	19.00%	16.13%		2364 R | 1805 A	43%	HiSeq	TP		
33	25	chr16:2152388	c.9195G>C	p.Val3065Val	het	rs9935834	NA	m	NA	19.00%	16.44%		2370 R | 1810 A	43%	HiSeq	TP		
33	25	chr16:2152651	c.8949-17A>G	NA	het	rs9928278	likely neutral	m	NA	21.00%	27.84%		2215 R | 1944 A	47%	HiSeq	TP		
33	26	chr16:2152129	c.9330T>C	p.Pro3110Pro	het	rs144582212	likely neutral	d	NA	11.00%	0.00%		2389 R | 374 A	14%	HiSeq	TP		
33	28	chr16:2150323	c.9569-13T>C	NA	het	rs11248911	likely neutral	m	NA	11.00%	0.00%		2678 R | 2052 A	43%	HiSeq	TP		
33	35	chr16:2144176	c.10535C>T	p.Ala3512Val	het	rs34197769	likely neutral	m	NA	7.00%	9.61%		1390 R | 1317 A	49%	HiSeq	TP		
33	42	chr16:2141396	c.11712+28G>C	NA	hom	rs11866494	likely neutral	d	NA	26.00%	0.00%		124 R | 28 A	18%	HiSeq	TP		edge of coverage, coding region covered properly, no adaption of pipeline
33	44	chr16:2140680	c.12133A>G	p.Ile4045Val	het	rs10960	likely neutral	m	NA	24.00%	31.10%		2558 R | 2306 A	47%	HiSeq	TP		
33	45	chr16:2140321	c.12409C>T	p.Leu4137Leu	het	rs79899502	likely neutral	m	NA	4.00%	5.84%		1155 R | 990 A	46%	HiSeq	TP		
33	45	chr16:2140454	c.12276A>G	p.Ala4092Ala	het	rs3087632	likely neutral	m	NA	26.00%	32.61%		1824 R | 1531 A	46%	HiSeq	TP		
33	45	chr16:2140554	c.12176C>T	p.Ala4059Val	het	rs3209986	likely neutral	m	NA	4.00%	5.82%		2891 R | 2309 A	44%	HiSeq	TP		
33	46	chr16:2140010	c.12630T>C	p.Pro4210Pro	het	rs7203729	likely neutral	m	NA	26.00%	33.03%		2308 R | 1892 A	45%	HiSeq	TP	133	
34	5	chr16:2167874	c.1119C>T	p.Leu373Leu	hom	rs199685642	likely neutral	d	NA	0.00%	0.00%		2595 R | 1300 A	33%	HiSeq	TP		zygosity
34	9	chr16:2165965_2165959	c.1849+28_1849+34del	NA	hom	rs56173969	NA	m	NA	9.00%	0.00%		13 R | 945 A	99%	HiSeq	TP		
34	11	chr16:2165630	c.1850-4A>G	NA	hom	rs35929659	likely neutral	m	NA	21.00%	22.78%		184 R | 1695 A	90%	HiSeq	TP		
34	11	chr16:2164808	c.2216G>A	p.Arg739Gln	hom	rs40433	likely neutral	m	NA	91.00%	0.00%		45 R | 1388 A	97%	HiSeq	TP		
34	11	chr16:2164324	c.2700G>A	p.Pro900Pro	hom	rs35667726	likely neutral	m	NA	3.00%	5.31%		337 R | 2295 A	87%	HiSeq	TP		
34	11	chr16:2164294	c.2730C>T	p.Asp910Asp	hom	rs35965348	likely neutral	m	NA	3.00%	5.33%		354 R | 2254 A	86%	HiSeq	TP		
34	15	chr16:2160503	c.4665A>C	p.Ala1555Ala	hom	rs71385734	likely neutral	m	NA	21.00%	28.26%		314 R | 2361 A	88%	HiSeq	TP		
34	15	chr16:2159996	c.5172C>T	p.Ala1724Ala	hom	rs9935526	likely neutral	m	NA	20.00%	27.23%		313 R | 2249 A	88%	HiSeq	TP		
34	15	chr16:2159405	c.5763G>A	p.Leu1921Leu	hom	rs2575313	likely neutral	m	NA	2.00%	3.44%		230 R | 1862 A	89%	HiSeq	TP		
34	17	chr16:2156850	c.7165T>C	p.Leu2389Leu	hom	rs2457533	likely neutral	d	NA	15.00%	0.00%		196 R | 331 A	63%	HiSeq	TP		zygosity
34	18	chr16:2156447	c.7441C>T	p.Leu2481Leu	hom	rs2003782	likely neutral	m	NA	21.00%	20.68%		208 R | 1693 A	89%	HiSeq	TP		
34	21	chr16:2155297	c.8016+26T>C	NA	hom	rs9934488	likely neutral	m	NA	20.00%	0.00%		266 R | 2148 A	89%	HiSeq	TP		
34	22	chr16:2154537	c.8123C>T	p.Thr2708Met	hom	rs147350387	likely neutral	m	NA	0.00%	1.14%		174 R | 997 A	85%	HiSeq	TP		
34	22	chr16:2154478	c.8161+21T>C	NA	hom	rs4786209	likely neutral	m	NA	56.00%	0.00%		24 R | 534 A	96%	HiSeq	TP		
34	25	chr16:2152651	c.8949-17A>G	NA	hom	rs9928278	likely neutral	m	NA	21.00%	27.84%		233 R | 2058 A	90%	HiSeq	TP		
34	25	chr16:2152388	c.9195G>C	p.Val3065Val	hom	rs9935834	NA	m	NA	19.00%	16.44%		350 R | 2032 A	85%	HiSeq	TP		
34	25	chr16:2152387	c.9196T>C	p.Phe3066Leu	hom	rs77028972	likely neutral	m	NA	19.00%	16.13%		352 R | 2017 A	85%	HiSeq	TP		
34	26	chr16:2152264	c.9202-7C>T	NA	het	NA	NA	d	NA	0.00%	0.00%		1844 R | 232 A	11%	HiSeq	TP		
34	26	chr16:2152129	c.9330T>C	p.Pro3110Pro	hom	rs144582212	likely neutral	d	NA	11.00%	0.00%		254 R | 579 A	70%	HiSeq	TP		zygosity
34	28	chr16:2150323	c.9569-13T>C	NA	hom	rs11248911	likely neutral	m	NA	11.00%	0.00%		316 R | 2227 A	88%	HiSeq	TP		
34	30	chr16:2149691	c.10004C>T	p.Pro3335Leu	het	NA	NA	m	NA	0.00%	0.00%	mutation?	1452 R | 1033 A	42%	HiSeq	TP		
34	35	chr16:2144176	c.10535C>T	p.Ala3512Val	hom	rs34197769	likely neutral	m	NA	7.00%	9.61%		145 R | 1792 A	93%	HiSeq	TP		
34	42	chr16:2141396	c.11712+28G>C	NA	hom	rs11866494	likely neutral	m	NA	26.00%	0.00%		22 R | 126 A	85%	HiSeq	TP		
34	42	chr16:2141395	c.11712+29C>T	NA	hom	NA	likely neutral	m	NA	0.00%	0.00%		25 R | 123 A	83%	HiSeq	TP		
34	44	chr16:2140680	c.12133A>G	p.Ile4045Val	hom	rs10960	likely neutral	m	NA	24.00%	31.10%		314 R | 2497 A	89%	HiSeq	TP		
34	45	chr16:2140554	c.12176C>T	p.Ala4059Val	hom	rs3209986	likely neutral	m	NA	4.00%	5.82%		428 R | 2400 A	85%	HiSeq	TP		
34	45	chr16:2140454	c.12276A>G	p.Ala4092Ala	hom	rs3087632	likely neutral	m	NA	26.00%	32.61%		223 R | 1886 A	89%	HiSeq	TP		
34	45	chr16:2140321	c.12409C>T	p.Leu4137Leu	hom	rs79899502	likely neutral	m	NA	4.00%	5.84%		165 R | 1572 A	91%	HiSeq	TP		
34	46	chr16:2140010	c.12630T>C	p.Pro4210Pro	hom	rs7203729	likely neutral	m	NA	26.00%	33.03%		257 R | 1941 A	88%	HiSeq	TP	131	
35	5	chr16:2167874	c.1119C>T	p.Leu373Leu	het	rs199685642	likely neutral	m	NA	0.00%	0.00%		2125 R | 568 A	21%	HiSeq	TP		
35	11	chr16:2164808	c.2216G>A	p.Arg739Gln	hom	rs40433	likely neutral	m	NA	91.00%	0.00%		51 R | 1197 A	96%	HiSeq	TP		
35	13	chr16:2162839	c.3111A>G	p.Leu1037Leu	het	rs2099534	likely neutral	m	NA	2.00%	4.13%		1544 R | 1205 A	44%	HiSeq	TP		
35	22	chr16:2154478	c.8161+21T>C	NA	hom	rs4786209	likely neutral	m	NA	56.00%	0.00%		27 R | 720 A	96%	HiSeq	TP		
35	28	chr16:2150296	c.9583T>C	p.Trp3195Arg	het	NA	NA	m	23985799	0.00%	0.00%	mutation?	1480 R | 1012 A	41%	HiSeq	TP		
35	29	chr16:2149848	c.9923+14G>C	NA	het	NA	NA	m	NA	0.00%	0.00%		1621 R | 1271 A	44%	HiSeq	TP	154	
36	5	chr16:2167874	c.1119C>T	p.Leu373Leu	hom	rs199685642	likely neutral	d	NA	0.00%	0.00%		2219 R | 1523 A	41%	HiSeq	TP		zygosity
36	9	chr16:2165965_2165959	c.1849+28_1849+34del	NA	het	rs56173969	NA	m	NA	9.00%	0.00%		155 R | 554 A	78%	HiSeq	TP		
36	11	chr16:2165630	c.1850-4A>G	NA	het	rs35929659	likely neutral	m	NA	21.00%	22.78%		1183 R | 1058 A	47%	HiSeq	TP		
36	11	chr16:2164808	c.2216G>A	p.Arg739Gln	hom	rs40433	likely neutral	m	NA	91.00%	0.00%		54 R | 1515 A	97%	HiSeq	TP		
36	11	chr16:2164324	c.2700G>A	p.Pro900Pro	het	rs35667726	likely neutral	m	NA	3.00%	5.31%		1600 R | 1244 A	44%	HiSeq	TP		
36	11	chr16:2164294	c.2730C>T	p.Asp910Asp	het	rs35965348	likely neutral	m	NA	3.00%	5.33%		1586 R | 1197 A	43%	HiSeq	TP		
36	15	chr16:2161655	c.3513C>G	p.Thr1171Thr	het	rs143784787	likely neutral	m	NA	1.00%	0.40%		1587 R | 1346 A	46%	HiSeq	TP		
36	15	chr16:2161501	c.3667G>A	p.Val1223Met	het	NA	NA	m	NA	0.00%	0.00%	mutation?	1996 R | 1450 A	42%	HiSeq	TP		
36	15	chr16:2160503	c.4665A>C	p.Ala1555Ala	het	rs71385734	likely neutral	m	NA	21.00%	28.26%		1921 R | 1673 A	47%	HiSeq	TP		
36	15	chr16:2159996	c.5172C>T	p.Ala1724Ala	het	rs9935526	likely neutral	m	NA	20.00%	27.23%		1836 R | 1533 A	46%	HiSeq	TP		
36	15	chr16:2159405	c.5763G>A	p.Leu1921Leu	het	rs2575313	likely neutral	m	NA	2.00%	3.44%		1343 R | 1010 A	43%	HiSeq	TP		

36	17	chr16:2156850	c.7165T>C	p.Leu2389Leu	het	rs2457533	likely neutral	d	NA	15.00%	0.00%		1306 R | 163 A	11%	HiSeq	TP		
36	18	chr16:2156447	c.7441C>T	p.Leu2481Leu	het	rs2003782	likely neutral	m	NA	21.00%	20.68%		1359 R | 1057 A	44%	HiSeq	TP		
36	21	chr16:2155424	c.7915C>T	p.Arg2639*	het	NA	definitely pathogenic	m	10854095	0.00%	0.00%	mutation	1699 R | 1240 A	42%	HiSeq	TP		
36	21	chr16:2155297	c.8016+26T>C	NA	het	rs9934488	likely neutral	m	NA	20.00%	0.00%		1724 R | 1222 A	41%	HiSeq	TP		
36	22	chr16:2154478	c.8161+21T>C	NA	hom	rs4786209	likely neutral	m	NA	56.00%	0.00%		33 R | 961 A	97%	HiSeq	TP		
36	25	chr16:2152651	c.8949-17A>G	NA	het	rs9928278	likely neutral	m	NA	21.00%	27.84%		1560 R | 1407 A	47%	HiSeq	TP		
36	25	chr16:2152388	c.9195G>C	p.Val3065Val	het	rs9935834	NA	m	NA	19.00%	16.44%		1530 R | 1257 A	45%	HiSeq	TP		
36	25	chr16:2152387	c.9196T>C	p.Phe3066Leu	het	rs77028972	likely neutral	m	NA	19.00%	16.13%		1523 R | 1248 A	45%	HiSeq	TP		
36	26	chr16:2152129	c.9330T>C	p.Pro3110Pro	het	rs144582212	likely neutral	d	NA	11.00%	0.00%		1509 R | 325 A	18%	HiSeq	TP		
36	28	chr16:2150323	c.9569-13T>C	NA	het	rs11248911	likely neutral	m	NA	11.00%	0.00%		1782 R | 1366 A	43%	HiSeq	TP		
36	35	chr16:2144176	c.10535C>T	p.Ala3512Val	het	rs34197769	likely neutral	m	NA	7.00%	9.61%		1334 R | 1126 A	46%	HiSeq	TP		
36	42	chr16:2141396	c.11712+28G>C	NA	het	rs11866494	likely neutral	m	NA	26.00%	0.00%		112 R | 36 A	24%	HiSeq	TP		coverage issue at the edge of exon, coding region covered properly, no adaption of pipeline necessary
36	44	chr16:2140680	c.12133A>G	p.Ile4045Val	het	rs10960	likely neutral	m	NA	24.00%	31.10%		1759 R | 1557 A	47%	HiSeq	TP		
36	45	chr16:2140554	c.12176C>T	p.Ala4059Val	het	rs3209986	likely neutral	m	NA	4.00%	5.82%		1846 R | 1534 A	45%	HiSeq	TP		
36	45	chr16:2140454	c.12276A>G	p.Ala4092Ala	het	rs3087632	likely neutral	m	NA	26.00%	32.61%		1294 R | 1220 A	49%	HiSeq	TP		
36	45	chr16:2140321	c.12409C>T	p.Leu4137Leu	het	rs79899502	likely neutral	m	NA	4.00%	5.84%		1131 R | 1013 A	47%	HiSeq	TP		
36	46	chr16:2140010	c.12630T>C	p.Pro4210Pro	het	rs7203729	likely neutral	m	NA	26.00%	33.03%		1576 R | 1208 A	43%	HiSeq	TP	132	
37	5	chr16:2167874	c.1119C>T	p.Leu373Leu	het	rs199685642	likely neutral	m	NA	0.00%	0.00%		2883 R | 706 A	20%	HiSeq	TP		
37	11	chr16:2164808	c.2216G>A	p.Arg739Gln	hom	rs40433	likely neutral	m	NA	91.00%	0.00%		35 R | 1303 A	97%	HiSeq	TP		
37	19	chr16:2156255	c.7540C>T	p.Leu2514Leu	het	rs183417822	NA	m	NA	0.05%	0.01%		1368 R | 1003 A	42%	HiSeq	TP		
37	19	chr16:2156159	c.7636C>T	p.His2546Tyr	het	rs200037070	likely neutral	m	21115670	0.00%	0.10%		1429 R | 940 A	40%	HiSeq	TP		
37	22	chr16:2154478	c.8161+21T>C	NA	hom	rs4786209	likely neutral	m	NA	56.00%	0.00%		21 R | 773 A	97%	HiSeq	TP		
37	31	chr16:2147895	c.10141C>T	p.Leu3381Phe	het	rs142799331	NA	m	NA	0.00%	0.01%	mutation?	1305 R | 896 A	41%	HiSeq	TP	154	
38	5	chr16:2167874	c.1119C>T	p.Leu373Leu	het	rs199685642	likely neutral	m	NA	0.00%	0.00%		3716 R | 902 A	20%	HiSeq	TP		
38	8	chr16:2166623	c.1626_1628dupGAA	p.Asn543delinsLysAsn	het	NA	NA	m	NA	0.00%	0.00%	mutation?	888 R | 662 A	43%	HiSeq	TP		
38	11	chr16:2164808	c.2216G>A	p.Arg739Gln	hom	rs40433	likely neutral	m	NA	91.00%	0.00%		53 R | 1628 A	97%	HiSeq	TP		
38	15	chr16:2159316	c.5852G>A	p.Arg1951Gln	het	rs137905643	NA	m	NA	0.00%	0.06%		2089 R | 1603 A	43%	HiSeq	TP		
38	22	chr16:2154478	c.8161+21T>C	NA	hom	rs4786209	likely neutral	m	NA	56.00%	0.00%		61 R | 1057 A	95%	HiSeq	TP		
38	28	chr16:2150210	c.9669G>A	p.Thr3223Thr	het	rs144817614	likely neutral	m	NA	0.00%	1.24%		2589 R | 1656 A	39%	HiSeq	TP	154	
39	5	chr16:2167874	c.1119C>T	p.Leu373Leu	het	rs199685642	likely neutral	d	NA	0.00%	0.00%		3122 R | 701 A	18%	HiSeq	TP		
39	11	chr16:2165630	c.1850-4A>G	NA	het	rs35929659	likely neutral	m	NA	21.00%	22.78%		1129 R | 978 A	46%	HiSeq	TP		
39	11	chr16:2164808	c.2216G>A	p.Arg739Gln	hom	rs40433	likely neutral	m	NA	91.00%	0.00%		44 R | 1343 A	97%	HiSeq	TP		
39	13	chr16:2162887	c.3063T>C	p.Gly1021Gly	het	rs2369068	likely neutral	m	NA	16.00%	22.58%		1934 R | 1378 A	42%	HiSeq	TP		
39	14	chr16:2162361	c.3275T>C	p.Met1092Thr	het	rs2549677	likely neutral	m	NA	17.00%	0.00%		1283 R | 692 A	35%	HiSeq	TP		
39	15	chr16:2161796	c.3372C>T	p.Ala1124Ala	het	rs75510884	likely neutral	m	NA	11.00%	14.61%		1426 R | 1030 A	42%	HiSeq	TP		
39	15	chr16:2161793	c.3375C>T	p.Ser1125Ser	het	rs74331768	likely neutral	m	NA	11.00%	14.45%		1438 R | 1025 A	42%	HiSeq	TP		
39	15	chr16:2160973	c.4195T>C	p.Trp1399Arg	het	rs116092985	likely neutral	m	NA	5.00%	0.00%		1673 R | 1206 A	42%	HiSeq	TP		
39	15	chr16:2160503	c.4665A>C	p.Ala1555Ala	het	rs71385734	likely neutral	m	NA	21.00%	28.26%		1681 R | 1349 A	45%	HiSeq	TP		
39	15	chr16:2159996	c.5172C>T	p.Ala1724Ala	het	rs9935526	likely neutral	m	NA	20.00%	27.23%		1493 R | 1260 A	46%	HiSeq	TP		
39	20	chr16:2156021	c.7708T>C	p.Leu2570Leu	het	rs28575767	likely neutral	m	NA	15.00%	0.00%		1546 R | 816 A	35%	HiSeq	TP		
39	21	chr16:2155426	c.7913A>G	p.His2638Arg	het	rs9936785	likely neutral	m	NA	14.00%	0.00%		1607 R | 355 A	18%	HiSeq	TP		
39	21	chr16:2155297	c.8016+26T>C	NA	het	rs9934488	likely neutral	m	NA	20.00%	0.00%		1507 R | 1042 A	41%	HiSeq	TP		
39	22	chr16:2154478	c.8161+21T>C	NA	het	rs4786209	likely neutral	m	NA	56.00%	0.00%		338 R | 437 A	56%	HiSeq	TP		
39	25	chr16:2152651	c.8949-17A>G	NA	het	rs9928278	likely neutral	m	NA	21.00%	27.84%		1290 R | 1186 A	48%	HiSeq	TP		
39	25	chr16:2152388	c.9195G>C	p.Val3065Val	het	rs9935834	NA	m	NA	19.00%	16.44%		1495 R | 1247 A	45%	HiSeq	TP		
39	25	chr16:2152387	c.9196T>C	p.Phe3066Leu	het	rs77028972	likely neutral	m	NA	19.00%	16.13%		1497 R | 1242 A	45%	HiSeq	TP		
39	26	chr16:2152129	c.9330T>C	p.Pro3110Pro	het	rs144582212	likely neutral	d	NA	11.00%	0.00%		1346 R | 304 A	18%	HiSeq	TP		
39	46	chr16:2139958	c.12682C>T	p.Arg4228*	het	rs199476095	definitely pathogenic	m	8554072	0.00%	0.00%	mutation	1428 R | 1007 A	41%	HiSeq	TP	141	
40	5	chr16:2168237	c.755dupC	p.Pro253Alafs*8	het	NA	Definitely pathogenic	m	15772804	0.00%	0.00%	mutation	475 R | 260 A	35%	HiSeq	TP		
40	5	chr16:2167874	c.1119C>T	p.Leu373Leu	het	rs199685642	likely neutral	m	NA	0.00%	0.00%		3435 R | 862 A	20%	HiSeq	TP		
40	11	chr16:2164808	c.2216G>A	p.Arg739Gln	het	rs40433	likely neutral	m	NA	91.00%	0.00%		1388 R | 755 A	35%	HiSeq	TP		
40	11	chr16:2164330	c.2694A>C	p.Ala898Ala	het	rs142357713	likely neutral	m	NA	1.00%	1.57%		1993 R | 1295 A	39%	HiSeq	TP		
40	23	chr16:2153619	c.8439C>T	p.Ser2813Ser	het	rs117856830	likely neutral	m	NA	1.00%	0.57%		1482 R | 1090 A	42%	HiSeq	TP		
40	42	chr16:2141454	c.11682C>T	p.Ser3894Ser	het	NA	likely neutral	m	NA	0.00%	0.00%		160 R | 193 A	55%	HiSeq	TP	154	
41	3	chr16:2169129	c.345delT	p.Phe115Leufs*3	het	NA	NA	m	NA	0.00%	0.00%	mutation	290 R | 213 A	42%	HiSeq	TP		
41	5	chr16:2167874	c.1119C>T	p.Leu373Leu	hom	rs199685642	likely neutral	d	NA	0.00%	0.00%		1789 R | 1356 A	43%	HiSeq	TP		zygosity
41	11	chr16:2164808	c.2216G>A	p.Arg739Gln	hom	rs40433	likely neutral	m	NA	91.00%	0.00%		53 R | 1410 A	96%	HiSeq	TP		
41	19	chr16:2156369	c.7489+30C>T	NA	het	rs142761413	NA	m	NA	2.00%	1.60%		1500 R | 982 A	40%	HiSeq	TP		
41	22	chr16:2154478	c.8161+21T>C	NA	het	rs4786209	likely neutral	m	NA	56.00%	0.00%		339 R | 373 A	52%	HiSeq	TP		
41	40	chr16:2142113	c.11346C>T	p.Asp3782Asp	het	rs145955373	likely neutral	m	NA	0.46%	0.35%		1081 R | 748 A	41%	HiSeq	TP		
41	41	chr16:2141776	c.11537+3_11537+5dupG(	NA	het	rs201204878	likely neutral	m	10862097	0.00%	0.00%		1215 R | 917 A	43%	HiSeq	TP		
41	43	chr16:2140912	c.11976C>G	p.Ala3992Ala	het	rs112387277	likely neutral	m	NA	0.05%	0.23%		1150 R | 837 A	42%	HiSeq	TP		
41	45	chr16:2140294	c.12433G>A	p.Val4146Ile	het	rs148478410	likely neutral	m	NA	0.41%	0.32%		976 R | 633 A	39%	HiSeq	TP	151	
42	1	chr16:2185509	c.182C>T	p.Pro61Leu	het	NA	likely neutral	m	NA	0.00%	0.00%		267 R | 129 A	33%	HiSeq	TP		
42	1	chr16:2185476	c.215T>A	p.Leu72Gln	het	NA	highly likely pathoger	m	NA	0.00%	0.00%	mutation	546 R | 183 A	25%	HiSeq	TP		
42	5	chr16:2168405	c.588C>T	p.Ser196Ser	het	NA	likely neutral	m	NA	0.00%	0.00%		1538 R | 486 A	24%	HiSeq	TP		
42	5	chr16:2167874	c.1119C>T	p.Leu373Leu	hom	rs199685642	likely neutral	d	NA	0.00%	0.00%		1535 R | 1154 A	43%	HiSeq	TP		zygosity
42	9	chr16:2165965_2165959	c.1849+28_1849+34del	NA	hom	rs56173969	NA	m	NA	9.00%	0.00%		130 R | 472 A	78%	HiSeq	TP		
42	11	chr16:2165630	c.1850-4A>G	NA	hom	rs35929659	likely neutral	m	NA	21.00%	22.78%		162 R | 1501 A	90%	HiSeq	TP		
42	11	chr16:2164808	c.2216G>A	p.Arg739Gln	hom	rs40433	likely neutral	m	NA	91.00%	0.00%		26 R | 1141 A	98%	HiSeq	TP		
42	11	chr16:2164324	c.2700G>A	p.Pro900Pro	het	rs35667726	likely neutral	m	NA	3.00%	5.31%		1258 R | 962 A	43%	HiSeq	TP		
42	11	chr16:2164294	c.2730C>T	p.Asp910Asp	het	rs35965348	likely neutral	m	NA	3.00%	5.33%		1210 R | 923 A	43%	HiSeq	TP		
42	13	chr16:2162887	c.3063T>C	p.Gly1021Gly	het	rs2369068	likely neutral	m	NA	16.00%	22.58%		1532 R | 1148 A	43%	HiSeq	TP		
42	14	chr16:2162361	c.3275T>C	p.Met1092Thr	het	rs2549677	likely neutral	m	NA	17.00%	0.00%		968 R | 561 A	37%	HiSeq	TP		
42	15	chr16:2161796	c.3372C>T	p.Ala1124Ala	het	rs75510884	likely neutral	m	NA	11.00%	14.61%		1089 R | 806 A	43%	HiSeq	TP		
42	15	chr16:2161793	c.3375C>T	p.Ser1125Ser	het	rs74331768	likely neutral	m	NA	11.00%	14.45%		1102 R | 810 A	42%	HiSeq	TP		
42	15	chr16:2161655	c.3513C>G	p.Thr1171Thr	het	rs143784787	likely neutral	m	NA	1.00%	0.40%		1182 R | 968 A	45%	HiSeq	TP		
42	15	chr16:2160973	c.4195T>C	p.Trp1399Arg	het	rs116092985	likely neutral	m	NA	5.00%	0.00%		1468 R | 901 A	38%	HiSeq	TP		
42	15	chr16:2160503	c.4665A>C	p.Ala1555Ala	hom	rs71385734	likely neutral	m	NA	21.00%	28.26%		268 R | 2071 A	89%	HiSeq	TP		
42	15	chr16:2159996	c.5172C>T	p.Ala1724Ala	hom	rs9935526	likely neutral	m	NA	20.00%	27.23%		253 R | 2117 A	89%	HiSeq	TP		

42	15	chr16:2159405	c.5763G>A	p.Leu1921Leu	het	rs2575313	likely neutral	m	NA	2.00%	3.44%		1040 R | 832 A	44%	HiSeq	TP		
42	17	chr16:2156850	c.7165T>C	p.Leu2389Leu	hom	rs2457533	likely neutral	d	NA	15.00%	0.00%		190 R | 291 A	60%	HiSeq	TP		zygosity
42	18	chr16:2156447	c.7441C>T	p.Leu2481Leu	hom	rs2003782	likely neutral	m	NA	21.00%	20.68%		211 R | 1518 A	88%	HiSeq	TP		
42	20	chr16:2156021	c.7708T>C	p.Leu2570Leu	het	rs28575767	likely neutral	m	NA	15.00%	0.00%		1406 R | 606 A	30%	HiSeq	TP		
42	21	chr16:2155426	c.7913A>G	p.His2638Arg	het	rs9936785	likely neutral	m	NA	14.00%	0.00%		1198 R | 303 A	20%	HiSeq	TP		
42	21	chr16:2155297	c.8016+26T>C	NA	hom	rs9934488	likely neutral	m	NA	20.00%	0.00%		287 R | 1712 A	86%	HiSeq	TP		
42	22	chr16:2154478	c.8161+21T>C	NA	het	rs4786209	likely neutral	m	NA	56.00%	0.00%		288 R | 328 A	53%	HiSeq	TP		
42	25	chr16:2152651	c.8949-17A>G	NA	hom	rs9928278	likely neutral	m	NA	21.00%	27.84%		178 R | 1895 A	91%	HiSeq	TP		
42	25	chr16:2152388	c.9195G>C	p.Val3065Val	hom	rs9935834	NA	m	NA	19.00%	16.44%		229 R | 1884 A	89%	HiSeq	TP		
42	25	chr16:2152387	c.9196T>C	p.Phe3066Leu	hom	rs77028972	likely neutral	m	NA	19.00%	16.13%		230 R | 1876 A	89%	HiSeq	TP		
42	26	chr16:2152129	c.9330T>C	p.Pro3110Pro	hom	rs144582212	likely neutral	d	NA	11.00%	0.00%		219 R | 554 A	72%	HiSeq	TP		zygosity
42	28	chr16:2150323	c.9569-13T>C	NA	het	rs11248911	likely neutral	m	NA	11.00%	0.00%		1323 R | 998 A	43%	HiSeq	TP		
42	35	chr16:2144176	c.10535C>T	p.Ala3512Val	het	rs34197769	likely neutral	m	NA	7.00%	9.61%		994 R | 852 A	46%	HiSeq	TP		
42	42	chr16:2141396	c.11712+28G>C	NA	hom	rs11866494	likely neutral	d	NA	26.00%	0.00%		27 R | 27 A	50%	HiSeq	TP		zygosity; edge of coverage, coding region covered properly, no adaption of pipeline
42	44	chr16:2140680	c.12133A>G	p.Ile4045Val	hom	rs10960	likely neutral	m	NA	24.00%	31.10%		211 R | 2035 A	91%	HiSeq	TP		
42	45	chr16:2140554	c.12176C>T	p.Ala4059Val	het	rs3209986	likely neutral	m	NA	4.00%	5.82%		1328 R | 1055 A	44%	HiSeq	TP		
42	45	chr16:2140454	c.12276A>G	p.Ala4092Ala	hom	rs3087632	likely neutral	m	NA	26.00%	32.61%		203 R | 1803 A	90%	HiSeq	TP		
42	45	chr16:2140321	c.12409C>T	p.Leu4137Leu	het	rs79899502	likely neutral	m	NA	4.00%	5.84%		860 R | 765 A	47%	HiSeq	TP		
42	46	chr16:2140010	c.12630T>C	p.Pro4210Pro	hom	rs7203729	likely neutral	m	NA	26.00%	33.03%		226 R | 1722 A	88%	HiSeq	TP	124	
43	5	chr16:2167874	c.1119C>T	p.Leu373Leu	het	rs199685642	likely neutral	m	NA	0.00%	0.00%		2473 R | 759 A	23%	HiSeq	TP		
43	11	chr16:2164808	c.2216G>A	p.Arg739Gln	hom	rs40433	likely neutral	m	NA	91.00%	0.00%		43 R | 1499 A	97%	HiSeq	TP		
43	22	chr16:2154640	c.8020C>T	p.Pro2674Ser	het	rs144557371	likely neutral	m	NA	0.00%	0.58%		1471 R | 862 A	37%	HiSeq	TP		
43	22	chr16:2154478	c.8161+21T>C	NA	het	rs4786209	likely neutral	m	NA	56.00%	0.00%		334 R | 390 A	54%	HiSeq	TP		
43	46	chr16:2139969_2139966	c.12671_12674del	p.Thr4224Serfs*133	het	NA	NA	m	NA	0.00%	0.00%	mutation	1479 R | 1040 A	41%	HiSeq	TP	155	
44	5	chr16:2167874	c.1119C>T	p.Leu373Leu	het	rs199685642	likely neutral	d	NA	0.00%	0.00%		1893 R | 395 A	17%	HiSeq	TP		
44	11	chr16:2164808	c.2216G>A	p.Arg739Gln	hom	rs40433	likely neutral	m	NA	91.00%	0.00%		39 R | 1003 A	96%	HiSeq	TP		
44	13	chr16:2162839	c.3111A>G	p.Leu1037Leu	het	rs2099534	likely neutral	m	NA	2.00%	4.13%		1295 R | 1014 A	44%	HiSeq	TP		
44	15	chr16:2160333	c.4835C>T	p.Thr1612Met	het	NA	NA	m	NA	0.00%	0.00%	mutation?	1222 R | 853 A	41%	HiSeq	TP		
44	15	chr16:2158484	c.6684C>G	p.Tyr2228*	het	NA	NA	m	NA	0.00%	0.00%	mutation	941 R | 730 A	44%	HiSeq	TP		
44	16	chr16:2158022	c.6927C>T	p.Gly2309Gly	het	rs189277711	likely neutral	m	NA	1.00%	1.20%		666 R | 600 A	47%	HiSeq	TP		
44	21	chr16:2156038	c.7704-13C>G	NA	het	NA	NA	m	NA	0.00%	0.00%		1022 R | 730 A	42%	HiSeq	TP		
44	22	chr16:2154478	c.8161+21T>C	NA	hom	rs4786209	likely neutral	m	NA	56.00%	0.00%		25 R | 521 A	95%	HiSeq	TP	152	
45	5	chr16:2167874	c.1119C>T	p.Leu373Leu	hom	rs199685642	likely neutral	d	NA	0.00%	0.00%		1107 R | 815 A	42%	HiSeq	TP		zygosity
45	11	chr16:2164808	c.2216G>A	p.Arg739Gln	hom	rs40433	likely neutral	m	NA	91.00%	0.00%		30 R | 816 A	96%	HiSeq	TP		
45	15	chr16:2161666	c.3502C>T	p.Pro1168Ser	het	rs146887330	likely neutral	m	NA	1.00%	1.07%		834 R | 595 A	42%	HiSeq	TP		
45	15	chr16:2161098	c.4070delT	p.Leu1357Argfs*9	het	NA	definitely pathogenic	m	17582161	0.00%	0.00%	mutation	1132 R | 749 A	40%	HiSeq	TP		
45	22	chr16:2154478	c.8161+21T>C	NA	het	rs4786209	likely neutral	m	NA	56.00%	0.00%		109 R | 187 A	63%	HiSeq	TP	155	
47	5	chr16:2167874	c.1119C>T	p.Leu373Leu	hom	rs199685642	likely neutral	d	NA	0.00%	0.00%		2989 R | 1613 A	35%	HiSeq	TP		zygosity
47	9	chr16:2165965_2165959	c.1849+28_1849+34del	NA	het	rs56173969	NA	m	NA	9.00%	0.00%		144 R | 555 A	79%	HiSeq	TP		
47	11	chr16:2165630	c.1850-4A>G	NA	het	rs35929659	likely neutral	m	NA	21.00%	22.78%		1214 R | 1086 A	47%	HiSeq	TP		
47	11	chr16:2164808	c.2216G>A	p.Arg739Gln	hom	rs40433	likely neutral	m	NA	91.00%	0.00%		50 R | 1666 A	97%	HiSeq	TP		
47	11	chr16:2164324	c.2700G>A	p.Pro900Pro	het	rs35667726	likely neutral	m	NA	3.00%	5.31%		1624 R | 1381 A	46%	HiSeq	TP		
47	11	chr16:2164294	c.2730C>T	p.Asp910Asp	het	rs35965348	likely neutral	m	NA	3.00%	5.33%		1581 R | 1309 A	45%	HiSeq	TP		
47	13	chr16:2162839	c.3111A>G	p.Leu1037Leu	het	rs2099534	likely neutral	m	NA	2.00%	4.13%		1916 R | 1464 A	43%	HiSeq	TP		
47	15	chr16:2160503	c.4665A>C	p.Ala1555Ala	het	rs71385734	likely neutral	m	NA	21.00%	28.26%		1822 R | 1557 A	46%	HiSeq	TP		
47	15	chr16:2159996	c.5172C>T	p.Ala1724Ala	het	rs9935526	likely neutral	m	NA	20.00%	27.23%		1663 R | 1596 A	49%	HiSeq	TP		
47	17	chr16:2156850	c.7165T>C	p.Leu2389Leu	het	rs2457533	likely neutral	d	NA	15.00%	0.00%		1301 R | 308 A	19%	HiSeq	TP		
47	18	chr16:2156447	c.7441C>T	p.Leu2481Leu	het	rs2003782	likely neutral	m	NA	21.00%	20.68%		1372 R | 1006 A	42%	HiSeq	TP		
47	21	chr16:2155297	c.8016+26T>C	NA	het	rs9934488	likely neutral	m	NA	20.00%	0.00%		1784 R | 1349 A	43%	HiSeq	TP		
47	22	chr16:2154478	c.8161+21T>C	NA	hom	rs4786209	likely neutral	m	NA	56.00%	0.00%		41 R | 695 A	94%	HiSeq	TP		
47	23	chr16:2153618	c.8440G>A	p.Gly2814Arg	het	rs149151043	likely neutral	m	NA	0.37%	0.59%		1407 R | 1121 A	44%	HiSeq	TP		
47	25	chr16:2152651	c.8949-17A>G	NA	het	rs9928278	likely neutral	m	NA	21.00%	27.84%		1431 R | 1263 A	47%	HiSeq	TP		
47	25	chr16:2152388	c.9195G>C	p.Val3065Val	het	rs9935834	NA	m	NA	19.00%	16.44%		1740 R | 1337 A	43%	HiSeq	TP		
47	25	chr16:2152387	c.9196T>C	p.Phe3066Leu	het	rs77028972	likely neutral	m	NA	19.00%	16.13%		1737 R | 1337 A	43%	HiSeq	TP		
47	26	chr16:2152129	c.9330T>C	p.Pro3110Pro	het	rs144582212	likely neutral	m	NA	11.00%	0.00%		1592 R | 375 A	19%	HiSeq	TP		
47	28	chr16:2150323	c.9569-13T>C	NA	het	rs11248911	likely neutral	m	NA	11.00%	0.00%		1830 R | 1364 A	43%	HiSeq	TP		
47	35	chr16:2144176	c.10535C>T	p.Ala3512Val	het	rs34197769	likely neutral	m	NA	7.00%	9.61%		1193 R | 1098 A	48%	HiSeq	TP		
47	42	chr16:2141396	c.11712+28G>C	NA	het	rs11866494	likely neutral	d	NA	26.00%	0.00%		46 R | 5 A	10%	HiSeq	TP		edge of coverage, coding region covered properly, no adaption of pipeline
47	44	chr16:2140680	c.12133A>G	p.Ile4045Val	het	rs10960	likely neutral	m	NA	24.00%	31.10%		1819 R | 1541 A	46%	HiSeq	TP		
47	45	chr16:2140554	c.12176C>T	p.Ala4059Val	het	rs3209986	likely neutral	m	NA	4.00%	5.82%		1834 R | 1599 A	47%	HiSeq	TP		
47	45	chr16:2140454	c.12276A>G	p.Ala4092Ala	het	rs3087632	likely neutral	m	NA	26.00%	32.61%		1338 R | 1340 A	50%	HiSeq	TP		
47	45	chr16:2140321	c.12409C>T	p.Leu4137Leu	het	rs79899502	likely neutral	m	NA	4.00%	5.84%		1059 R | 876 A	45%	HiSeq	TP		
47	46	chr16:2140149	c.12489_12490dupTC	p.Arg4164Leufs*35	het	NA	NA	m	NA	0.00%	0.00%	mutation	1022 R | 756 A	43%	HiSeq	TP		
47	46	chr16:2140010	c.12630T>C	p.Pro4210Pro	het	rs7203729	likely neutral	m	NA	26.00%	33.03%		1499 R | 1173 A	44%	HiSeq	TP	133	
48	5	chr16:2167874	c.1119C>T	p.Leu373Leu	hom	rs199685642	likely neutral	d	NA	0.00%	0.00%		2218 R | 1718 A	44%	HiSeq	TP		zygosity
48	8	chr16:2166542	c.1710C>T	p.His570His	het	rs367983387	likely neutral	m	NA	0.00%	0.34%		1215 R | 994 A	45%	HiSeq	TP		
48	11	chr16:2164808	c.2216G>A	p.Arg739Gln	hom	rs40433	likely neutral	m	NA	91.00%	0.00%		58 R | 1790 A	97%	HiSeq	TP		
48	19	chr16:2156240	c.7555C>T	p.Gln2519*	het	NA	definitely pathogenic	m	22508176	0.00%	0.00%	mutation	1795 R | 1316 A	42%	HiSeq	TP		
48	22	chr16:2154478	c.8161+21T>C	NA	het	rs4786209	likely neutral	m	NA	56.00%	0.00%		327 R | 361 A	52%	HiSeq	TP		
48	35	chr16:2144176	c.10535C>T	p.Ala3512Val	het	rs34197769	likely neutral	m	NA	7.00%	9.61%		1254 R | 1187 A	49%	HiSeq	TP		
48	42	chr16:2141396	c.11712+28G>C	NA	het	rs11866494	likely neutral	m	NA	26.00%	0.00%		56 R | 37 A	40%	HiSeq	TP		
48	42	chr16:2141395	c.11712+29C>T	NA	het	NA	likely neutral	m	NA	0.00%	0.00%		57 R | 35 A	38%	HiSeq	TP		
48	44	chr16:2140680	c.12133A>G	p.Ile4045Val	het	rs10960	likely neutral	m	NA	24.00%	31.10%		1830 R | 1726 A	49%	HiSeq	TP		
48	45	chr16:2140554	c.12176C>T	p.Ala4059Val	het	rs3209986	likely neutral	m	NA	4.00%	5.82%		2008 R | 1585 A	44%	HiSeq	TP		
48	45	chr16:2140454	c.12276A>G	p.Ala4092Ala	het	rs3087632	likely neutral	m	NA	26.00%	32.61%		1536 R | 1244 A	45%	HiSeq	TP		
48	45	chr16:2140321	c.12409C>T	p.Leu4137Leu	het	rs79899502	likely neutral	m	NA	4.00%	5.84%		1232 R | 1019 A	45%	HiSeq	TP		
48	46	chr16:2140010	c.12630T>C	p.Pro4210Pro	het	rs7203729	likely neutral	m	NA	26.00%	33.03%		1588 R | 1293 A	45%	HiSeq	TP	147	
49	5	chr16:2168390	c.603C>G	p.His201Gln	het	NA	likely neutral	m	NA	0.00%	0.00%		3280 R | 1108 A	25%	HiSeq	TP		
49	5	chr16:2167970	c.1023C>T	p.Ala341Ala	het	NA	likely neutral	m	NA	0.00%	0.00%		3302 R | 1538 A	32%	HiSeq	TP		

49	5	chr16:2167874	c.1119C>T	p.Leu373Leu	het	rs199685642	likely neutral	d	NA	0.00%	0.00%		6012 R | 1265 A	17%	HiSeq	TP		
49	11	chr16:2164808	c.2216G>A	p.Arg739Gln	het	rs40433	likely neutral	m	NA	91.00%	0.00%		2045 R | 1264 A	38%	HiSeq	TP		
49	11	chr16:2164211	c.2813C>T	p.Thr938Met	NA	rs148709380	NA	p	NA	5.00%	0.00%		3529 R | 938 A	21%	HiSeq	FP		
49	22	chr16:2154478	c.8161+21T>C	NA	het	rs4786209	likely neutral	m	NA	56.00%	0.00%		405 R | 520 A	56%	HiSeq	TP		
49	23	chr16:2153695	c.8363C>G	p.Ser2788Trp	het	NA	likely pathogenic	m	NA	0.00%	0.00%	mutation?	1770 R | 1575 A	47%	HiSeq	TP	154	
50	5	chr16:2167874	c.1119C>T	p.Leu373Leu	het	rs199685642	likely neutral	d	NA	0.00%	0.00%		4581 R | 845 A	16%	HiSeq	TP		
50	11	chr16:2164808	c.2216G>A	p.Arg739Gln	hom	rs40433	likely neutral	m	NA	91.00%	0.00%		47 R | 1690 A	97%	HiSeq	TP		
50	22	chr16:2154478	c.8161+21T>C	NA	hom	rs4786209	likely neutral	m	NA	56.00%	0.00%		25 R | 915 A	97%	HiSeq	TP		
50	23	chr16:2153765	c.8293C>T	p.Arg2765Cys	het	rs144979397	likely hypomorphic	m	19165178	0.46%	0.58%	mutation?	1838 R | 1492 A	45%	HiSeq	TP		
50	25	chr16:2152396	c.9187C>T	p.Arg3063Cys	het	rs145906459	likely neutral	m	17582161	0.00%	0.03%		2053 R | 1402 A	41%	HiSeq	TP		
50	45	chr16:2140291_2140289	c.12439_12441del	p.Lys4147del	het	NA	NA	m	NA	0.00%	0.00%	mutation?	1055 R | 855 A	45%	HiSeq	TP	154	
51	5	chr16:2167874	c.1119C>T	p.Leu373Leu	hom	rs199685642	likely neutral	d	NA	0.00%	0.00%		2598 R | 1705 A	40%	HiSeq	TP		zygosity
51	11	chr16:2165630	c.1850-4A>G	NA	hom	rs35929659	likely neutral	m	NA	21.00%	22.78%		254 R | 2178 A	90%	HiSeq	TP		
51	11	chr16:2164808	c.2216G>A	p.Arg739Gln	hom	rs40433	likely neutral	m	NA	91.00%	0.00%		46 R | 1826 A	98%	HiSeq	TP		
51	12	chr16:2163189	c.2958T>G	p.Tyr986*	het	NA	definitely pathogenic	m	22508176	0.00%	0.00%	mutation	678 R | 343 A	34%	HiSeq	TP		
51	13	chr16:2162887	c.3063T>C	p.Gly1021Gly	hom	rs2369068	likely neutral	m	NA	16.00%	22.58%		658 R | 4503 A	87%	HiSeq	TP		
51	14	chr16:2162361	c.3275T>C	p.Met1092Thr	hom	rs2549677	likely neutral	m	NA	17.00%	0.00%		350 R | 1683 A	83%	HiSeq	TP		slightly below homozygosity calling threshold, correct
51	15	chr16:2161796	c.3372C>T	p.Ala1124Ala	hom	rs75510884	likely neutral	m	NA	11.00%	14.61%		350 R | 2621 A	88%	HiSeq	TP		
51	15	chr16:2161793	c.3375C>T	p.Ser1125Ser	hom	rs74331768	likely neutral	m	NA	11.00%	14.45%		355 R | 2634 A	88%	HiSeq	TP		
51	15	chr16:2161150	c.4018C>T	p.Arg1340Trp	het	rs143690392	likely neutral	m	11967008	0.14%	0.31%		2659 R | 2158 A	45%	HiSeq	TP		
51	15	chr16:2160973	c.4195T>C	p.Trp1399Arg	hom	rs116092985	likely neutral	m	NA	5.00%	0.00%		525 R | 3195 A	86%	HiSeq	TP		
51	15	chr16:2160503	c.4665A>C	p.Ala1555Ala	hom	rs71385734	likely neutral	m	NA	21.00%	28.26%		365 R | 3761 A	91%	HiSeq	TP		
51	15	chr16:2159996	c.5172C>T	p.Ala1724Ala	hom	rs9935526	likely neutral	m	NA	20.00%	27.23%		334 R | 3904 A	92%	HiSeq	TP		
51	17	chr16:2156850	c.7165T>C	p.Leu2389Leu	hom	rs2457533	likely neutral	d	NA	15.00%	0.00%		282 R | 350 A	55%	HiSeq	TP		zygosity
51	18	chr16:2156447	c.7441C>T	p.Leu2481Leu	hom	rs2003782	likely neutral	m	NA	21.00%	20.68%		288 R | 2171 A	88%	HiSeq	TP		
51	20	chr16:2156021	c.7708T>C	p.Leu2570Leu	hom	rs28575767	likely neutral	m	NA	15.00%	0.00%		422 R | 2303 A	85%	HiSeq	TP		
51	21	chr16:2155426	c.7913A>G	p.His2638Arg	hom	rs9936785	likely neutral	d	NA	14.00%	0.00%		344 R | 818 A	70%	HiSeq	TP		zygosity
51	21	chr16:2155297	c.8016+26T>C	NA	hom	rs9934488	likely neutral	m	NA	20.00%	0.00%		387 R | 2757 A	88%	HiSeq	TP		
51	22	chr16:2154537	c.8123C>T	p.Thr2708Met	het	rs147350387	likely neutral	m	NA	0.00%	1.14%		1257 R | 938 A	43%	HiSeq	TP		
51	22	chr16:2154478	c.8161+21T>C	NA	het	rs4786209	likely neutral	m	NA	56.00%	0.00%		545 R | 484 A	47%	HiSeq	TP		
51	25	chr16:2152651	c.8949-17A>G	NA	hom	rs9928278	likely neutral	m	NA	21.00%	27.84%		301 R | 3011 A	91%	HiSeq	TP		
51	25	chr16:2152388	c.9195G>C	p.Val3065Val	hom	rs9935834	NA	m	NA	19.00%	16.44%		354 R | 2869 A	89%	HiSeq	TP		
51	25	chr16:2152387	c.9196T>C	p.Phe3066Leu	hom	rs77028972	likely neutral	m	NA	19.00%	16.13%		354 R | 2864 A	89%	HiSeq	TP		
51	26	chr16:2152129	c.9330T>C	p.Pro3110Pro	hom	rs144582212	likely neutral	d	NA	11.00%	0.00%		319 R | 637 A	67%	HiSeq	TP		zygosity
51	36	chr16:2143865	c.10768C>T	p.Leu3590Leu	het	rs116114803	likely neutral	m	NA	1.00%	2.45%		1549 R | 1280 A	45%	HiSeq	TP		
51	42	chr16:2141396	c.11712+28G>C	NA	het	rs11866494	likely neutral	m	NA	26.00%	0.00%		113 R | 33 A	23%	HiSeq	TP		
51	43	chr16:2140972	c.11916C>T	p.Arg3972Arg	het	rs77634115	likely neutral	m	NA	1.00%	2.04%		1028 R | 1144 A	53%	HiSeq	TP		
51	44	chr16:2140680	c.12133A>G	p.Ile4045Val	het	rs10960	likely neutral	m	NA	24.00%	31.10%		2238 R | 1916 A	46%	HiSeq	TP		
51	44	chr16:2140653	c.12138+22delG	NA	het	rs199701927	NA	m	NA	1.00%	2.84%		2245 R | 1790 A	44%	HiSeq	TP		
51	45	chr16:2140454	c.12276A>G	p.Ala4092Ala	het	rs3087632	likely neutral	m	NA	26.00%	32.61%		1484 R | 1187 A	44%	HiSeq	TP		
51	46	chr16:2140010	c.12630T>C	p.Pro4210Pro	het	rs7203729	likely neutral	m	NA	26.00%	33.03%		1895 R | 1485 A	44%	HiSeq	TP	130	
52	5	chr16:2167874	c.1119C>T	p.Leu373Leu	het	rs199685642	likely neutral	m	NA	0.00%	0.00%		3092 R | 912 A	23%	HiSeq	TP		
52	11	chr16:2164808	c.2216G>A	p.Arg739Gln	hom	rs40433	likely neutral	m	NA	91.00%	0.00%		47 R | 1913 A	98%	HiSeq	TP		
52	16	chr16:2158022	c.6927C>T	p.Gly2309Gly	het	rs189277711	likely neutral	m	NA	1.00%	1.20%		1392 R | 1253 A	47%	HiSeq	TP		
52	22	chr16:2154478	c.8161+21T>C	NA	hom	rs4786209	likely neutral	m	NA	56.00%	0.00%		30 R | 943 A	97%	HiSeq	TP		
52	38	chr16:2142968	c.11143delC	p.Leu3715Trpfs*111	het	NA	definitely pathogenic	m	9399039	0.00%	0.00%	mutation	1873 R | 1323 A	41%	HiSeq	TP	155	
53	5	chr16:2167970	c.1023C>T	p.Ala341Ala	het	NA	likely neutral	m	NA	0.00%	0.00%		526 R | 292 A	36%	HiSeq	TP		
53	5	chr16:2167874	c.1119C>T	p.Leu373Leu	hom	rs199685642	likely neutral	d	NA	0.00%	0.00%		648 R | 451 A	41%	HiSeq	TP		zygosity
53	11	chr16:2164808	c.2216G>A	p.Arg739Gln	hom	rs40433	likely neutral	m	NA	91.00%	0.00%		26 R | 399 A	94%	HiSeq	TP		
53	15	chr16:2161524	c.3644T>A	p.Leu1215His	het	NA	NA	m	NA	0.00%	0.00%	mutation?	596 R | 416 A	41%	HiSeq	TP		
53	15	chr16:2161113	c.4055G>A	p.Ser1352Asn	het	rs141274774	indeterminate	m	NA	0.00%	0.10%	mutation?	732 R | 420 A	36%	HiSeq	TP		
53	15	chr16:2160494	c.4674G>A	p.Thr1558Thr	het	rs79884128	likely neutral	m	NA	8.00%	5.41%		605 R | 410 A	40%	HiSeq	TP		
53	22	chr16:2154478	c.8161+21T>C	NA	het	rs4786209	likely neutral	m	NA	56.00%	0.00%		125 R | 159 A	56%	HiSeq	TP		
53	27	chr16:2150466	c.9499A>T	p.Ile3167Phe	het	rs139945204	likely neutral	m	11967008	0.00%	0.07%		648 R | 474 A	42%	HiSeq	TP	152	
54	5	chr16:2167874	c.1119C>T	p.Leu373Leu	het	rs199685642	likely neutral	d	NA	0.00%	0.00%		3322 R | 615 A	16%	HiSeq	TP		
54	11	chr16:2164808	c.2216G>A	p.Arg739Gln	het	rs40433	likely neutral	m	NA	91.00%	0.00%		1011 R | 661 A	40%	HiSeq	TP		
54	11	chr16:2164211	c.2813C>T	p.Thr938Met	NA	rs148709380	NA	p	NA	5.00%	0.00%		1972 R | 690 A	26%	HiSeq	FP		
54	21	chr16:2155426	c.7913A>G	p.His2638Arg	het	rs9936785	likely neutral	m	NA	14.00%	0.00%		1376 R | 376 A	21%	HiSeq	TP		
54	22	chr16:2154478	c.8161+21T>C	NA	het	rs4786209	likely neutral	m	NA	56.00%	0.00%		338 R | 409 A	55%	HiSeq	TP		
54	23	chr16:2153765	c.8293C>T	p.Arg2765Cys	het	rs144979397	likely hypomorphic	m	19165178	0.46%	0.58%	mutation?	1342 R | 905 A	40%	HiSeq	TP		
54	25	chr16:2152396	c.9187C>T	p.Arg3063Cys	het	rs145906459	likely neutral	m	NA	0.00%	0.03%		1518 R | 1014 A	40%	HiSeq	TP		
54	31	chr16:2147986	c.10051-1G>A	NA	het	NA	NA	m	NA	0.00%	0.00%	mutation	1422 R | 855 A	38%	HiSeq	TP	153	
55	5	chr16:2167874	c.1119C>T	p.Leu373Leu	het	rs199685642	likely neutral	m	NA	0.00%	0.00%		4003 R | 1110 A	22%	HiSeq	TP		
55	7	chr16:2166924	c.1516G>T	p.Glu506*	het	NA	definitely pathogenic	m	22508176	0.00%	0.00%	mutation	1758 R | 1126 A	39%	HiSeq	TP		
55	11	chr16:2165630	c.1850-4A>G	NA	het	rs35929659	likely neutral	m	NA	21.00%	22.78%		1703 R | 1414 A	45%	HiSeq	TP		
55	11	chr16:2164808	c.2216G>A	p.Arg739Gln	het	rs40433	likely neutral	m	NA	91.00%	0.00%		1956 R | 1459 A	43%	HiSeq	TP		
55	15	chr16:2161415	c.3753C>T	p.Thr1251Thr	het	rs143556315	NA	m	NA	0.00%	0.01%		3099 R | 2448 A	44%	HiSeq	TP		
55	15	chr16:2161097	c.4071G>T	p.Leu1357Leu	het	rs145737766	likely neutral	m	NA	1.00%	0.68%		3436 R | 2717 A	44%	HiSeq	TP		
55	15	chr16:2159098	c.6070C>T	p.Arg2024Cys	het	rs199943712	NA	m	NA	0.05%	0.00%	mutation?	2614 R | 2050 A	44%	HiSeq	TP		
55	22	chr16:2154478	c.8161+21T>C	NA	het	rs4786209	likely neutral	m	NA	56.00%	0.00%		539 R | 691 A	56%	HiSeq	TP		
55	42	chr16:2141396	c.11712+28G>C	NA	het	rs11866494	likely neutral	m	NA	26.00%	0.00%		84 R | 44 A	34%	HiSeq	TP		
55	44	chr16:2140680	c.12133A>G	p.Ile4045Val	het	rs10960	likely neutral	m	NA	24.00%	31.10%		2625 R | 2268 A	46%	HiSeq	TP		
55	45	chr16:2140454	c.12276A>G	p.Ala4092Ala	het	rs3087632	likely neutral	m	NA	26.00%	32.61%		1814 R | 1544 A	46%	HiSeq	TP		
55	46	chr16:2140010	c.12630T>C	p.Pro4210Pro	het	rs7203729	likely neutral	m	NA	26.00%	33.03%		2160 R | 1703 A	44%	HiSeq	TP	148	

Comment 1: Variant could not be called due to default read filter "MappingQualityUnavailableFilter" of the UnifiedGenotyper, which cannot be disabled (see GATK discussion entry http://gatkforums.broadinstitute.org/discussion/2026/is-it-possible-to-disable-default-read-filters); high number of reads with mapping quality of zero (reference allele: 107 reads with MQ = 0 and 868 reads with MQ > 0; alternative allele: 32 reads with MQ = 0 and 46 reads with MQ > 0); variant was detected in patient 8

Cohort statistics	# samples	# samples with correctly identified mutation	detection rate
validation cohort	55	54	98.2%
SNV/indel validation	53	52	98.1%
CNV validation	2	2	100%

Variant statistics	#variants	# different variants	detection rate
Sanger verified variants (SNV, indels)	683	160	
variants detected	681	159	99.6%
definite and putatively pathogenic mutations	63	58	
detected mutations	62	58	98.4%
novel mutations	36	36	
true positive variants (TP)	681	-	
true negative sites (TN)	7645	-	
variants not detected (FN)	2	2	
false positive variants (FP)	17	2	
putative FP Sanger variants*	3	3	
sensitivity	99.7%		
specificity	99.8%		

Exact validation statistics in the *PKD1* patient cohort.
All identified and Sanger-verified variants regardless their pathogenic nature and allele frequency in databases were regarded as true positives (TP=681) with two missed variants as false negatives (FN=2). The sites of the 160 different and Sanger validated variants were used for determination of the false negative rate per sample (statistics-column) and specificity (TN=7642). Three Sanger positive variants with spurious amplification of pseudogene regions (Supplemental Figure S9D) were also defined as true negatives. *counted as TN sites in NGS.
Sensitivity was calculated as TP/(TP+FN)
Specificity was calculated as TN/(TN+FP)
