# Supplementary material for: An Efficient and Comprehensive Strategy for Genetic Diagnostics of Polycystic Kidney Disease
Source: PLoS One. 2015 Feb 3;10(2):e0116680. doi: 10.1371/journal.pone.0116680 (PMC4315576; doi:10.1371/journal.pone.0116680)
Supplement: S5 Table — (PDF) [file pone.0116680.s015.pdf]

| exon | c.-pos | p.-pos | zygosity | NGS | times detected with lower threshold/wrong zygosity | lowest % reads accross samples | comment |
|---|---|---|---|---|---|---|---|
| 1 | c.96C>T | p.Pro32Pro | het | n | 1 (FN = 1) | 0% | no coverage of exon 1 in MiSeq sequencing; site eventually covered by HiSeq sequencing, detectable |
| 5 | c.1119C>T | p.Leu373Leu | het | d | 38 | 16% | sequency identity with pseudogene regions at this site; discrimination only possible by paired read upstream of site; positive effect when filtering against low mapping quality reads (MQ>10) |
| 26 | c.9202-7C>T | NA | het | d | 1 | 11% | sequency identity with pseudogene regions at this site; discrimination only possible by paired read; positive effect when filtering against low mapping quality reads (MQ>10) |
| 10 | c.2081C>T | p.Pro694Leu | NA | p | 7 (FP=7) | 20% | master gene duplicated regions at site identical; annotated in dbSNP; possible NGS artefacts as detected slightly above the 20% alternative reads threshold and not detectable by Sanger sequencing with different primer sets; to be filtered out against internal database |
| 11 | c.2813C>T | p.Thr938Met | NA | p | 10 (FP=10) | 21% | |
| 11 | c.2180T>C | p.Leu727Pro | het | d | 1 (FN = 1) | 10% | reads with variant show 100% sequence identity with at least one pseudogene (gene conversion event?); read pairs favourably map against homologous pseudogene regions. Only few read pairs carrying the variant (mainly with low mapping quality) with possible discrimination map to the master gene; filtering against mapping quality not appropriate |
| 17 | c.7165T>C* | p.Leu2389Leu | het | d | 14 | 8% | |
| 21 | c.7913A>G | p.His2638Arg | het | d | 3 | 16% | |
| 26 | c.9330T>C | p.Pro3110Pro | het | d | 14 | 14% | |

**Table S5.** Sites with discordance between Sanger sequencing and NGS.

NA - not available; c.-pos – coding position; p.-pos - amino acid position; FN – false negative; het - heterozygous; hom – homozygous;

d - detected (below 20% reads-threshold, different zygosity); n – not detected; p – only detected in bioinformatic NGS pipeline

* of note: when nucleotide exchange is towards c.7165T>G (patient 8), read discrimination is supported and variant is clearly detectable.
