# Supplementary material for: An Efficient and Comprehensive Strategy for Genetic Diagnostics of Polycystic Kidney Disease
Source: PLoS One. 2015 Feb 3;10(2):e0116680. doi: 10.1371/journal.pone.0116680 (PMC4315576; doi:10.1371/journal.pone.0116680)
Supplement: S6 Table — (PDF) [file pone.0116680.s016.pdf]

**Table S6.** List of all "putatively" pathogenic *PKD1* variants previously reported in patients of the validation cohort.

Bioinformatic prediction of pathogenicity for so called putatively pathogenic *PKD1* variants. Either these variants were predicted clearly pathogenic by the described criteria or they had been reported as being listed in the *PKD1* database as indeterminate or putatively pathogenic, being private to the patient or due to uncertain assessment at the time of the report. Patients and variants had been included in this study for technical reasons and validation purposes and not only because of their clear pathogenicity.

| Pat-no | exon | hg19 position | c.-pos | p.-pos | rs-number | PKD1 DB entry | PubMed-ID | classification Table S4 | SIFT Score | SIFT Pred. | PolyP.-2 HumVar Score | PolyP.-2 HumVar Pred. | M.T. Score | M.T. Pred. | M.A. Score | M.A. Pred. | FATHMM Score | FATHMM Pred. | PhyloP Score | PhyloP Pred. | GERP++ RS Score | GERP++ Pred. |
|---|---|---|---|---|---|---|---|---|---|---|---|---|---|---|---|---|---|---|---|---|---|---|
| 13 | 4 | chr16:2168742 | c.464G>A | p.Cys155Tyr | NA | highly likely pathogenic | 22508176 | mutation? | 0 | Damaging | 0.545 | Possibly Damaging | 0.999 | Polymorphism | 4.355 | High | -2.66 | Damaging | 2.104 | Conserved | 4.58 | Conserved |
| 26 | 5 | chr16:2168181 | c.812C>A | p.Ala271Asp | NA | likely pathogenic | 17574468 | mutation? | 0.01 | Damaging | 0.255 | Benign | 0.556 | Disease Causing | 2.075 | Medium | -0.31 | Tolerated | 2.228 | Conserved | 4.78 | Conserved |
| 8, 21 | 11 | chr16:2164844 | c.2180T>C | p.Leu727Pro | NA | NA | 17582161 | mutation? | 0 | Damaging | 0.994 | Probably Damaging | 1 | Disease Causing | 2.28 | Medium | 0.64 | Tolerated | 1.945 | Conserved | 5.19 | Conserved |
| 7 | 15 | chr16:2160547 | c.4621A>C | p.Asn1541His | NA | NA | NA | mutation? | 0.06 | Tolerated | 0.982 | Probably Damaging | 0.992 | Polymorphism | 2.395 | Medium | -0.3 | Tolerated | 2.037 | Conserved | 5.36 | Conserved |
| 20 | 15 | chr16:2158260 | c.6908C>T | p.Ser2303Leu | rs143021666 | NA | NA | mutation? | 0.12 | Tolerated | 0.478 | Possibly Damaging | 1 | Polymorphism | 1.52 | Low | -0.5 | Tolerated | 2.539 | Conserved | 5.05 | Conserved |
| 32 | 15 | chr16:2161626 | c.3542A>G | p.Tyr1181Cys | NA | NA | NA | mutation? | 0 | Damaging | 0.989 | Probably Damaging | 0.885 | Polymorphism | 3.735 | High | -1.62 | Damaging | 0.037 | Conserved | 0.239 | Conserved |
| 36 | 15 | chr16:2161501 | c.3667G>A | p.Val1223Met | NA | NA | NA | mutation? | 0.04 | Damaging | 0.961 | Probably Damaging | 1 | Polymorphism | 1.87 | Low | -0.17 | Tolerated | 2.671 | Conserved | 4.52 | Conserved |
| 44 | 15 | chr16:2160333 | c.4835C>T | p.Thr1612Met | NA | NA | NA | mutation? | 0.01 | Damaging | 1 | Probably Damaging | 1 | Disease Causing | 1.1 | Low | -0.35 | Tolerated | 2.403 | Conserved | 5.12 | Conserved |
| 53 | 15 | chr16:2161524 | c.3644T>A | p.Leu1215His | NA | NA | NA | mutation? | 0 | Damaging | 1 | Probably Damaging | 1 | Disease Causing | 2.67 | Medium | 1.04 | Tolerated | 2.157 | Conserved | 5.66 | Conserved |
| 53 | 15 | chr16:2161113 | c.4055G>A | p.Ser1352Asn | rs141274774 | indeterminate | NA | mutation? | 0.15 | Tolerated | 0.57 | Possibly Damaging | 1 | Polymorphism | 1.87 | Low | 0.14 | Tolerated | 0.682 | Conserved | 2.26 | Conserved |
| 55 | 15 | chr16:2159098 | c.6070C>T | p.Arg2024Cys | rs199943712 | NA | NA | mutation? | 0.02 | Damaging | 0.988 | Probably Damaging | 0.963 | Polymorphism | 1.39 | Low | 0.12 | Tolerated | 2.597 | Conserved | 5.49 | Conserved |
| 49 | 23 | chr16:2153695 | c.8363C>G | p.Ser2788Trp | NA | likely pathogenic | NA | mutation? | 0.01 | Damaging | 0.994 | Probably Damaging | 1 | Disease Causing | 2.015 | Medium | 1.21 | Tolerated | 2.394 | Conserved | 4.62 | Conserved |
| 10, 15, 50, 54 | 23 | chr16:2153765 | c.8293C>T | p.Arg2765Cys | rs144979397 | likely hypomorphic | 19165178 | mutation? | 0.05 | Damaging | 0.79 | Possibly Damaging | 0.995 | Disease Causing | 1.78 | Low | 1.14 | Tolerated | 2.535 | Conserved | 3.88 | Conserved |
| 5 | 27 | chr16:2150481 | c.9484C>T | p.Arg3162Cys | NA | likely hypomorphic | 22034641 | mutation? | 0 | Damaging | 1 | Probably Damaging | 1 | Disease Causing | 4.005 | High | -0.9 | Tolerated | 2.443 | Conserved | 4.73 | Conserved |
| 35 | 28 | chr16:2150296 | c.9583T>C | p.Trp3195Arg | NA | NA | 23985799 | mutation? | 0 | Damaging | 1 | Probably Damaging | 1 | Disease Causing | 3.84 | High | -0.72 | Tolerated | 2.024 | Conserved | 4.84 | Conserved |
| 34 | 30 | chr16:2149691 | c.10004C>T | p.Pro3335Leu | NA | NA | NA | mutation? | 0 | Damaging | 1 | Probably Damaging | 1 | Disease Causing | 3.18 | Medium | -0.28 | Tolerated | 1.196 | Conserved | 3.74 | Conserved |
| 37 | 31 | chr16:2147895 | c.10141C>T | p.Leu3381Phe | rs142799331 | NA | NA | mutation? | 0.03 | Damaging | 0.977 | Probably Damaging | 0.602 | Disease Causing | 2.255 | Medium | 1.03 | Tolerated | 0.448 | Conserved | 2.29 | Conserved |
| 24 | 33 | chr16:2147410 | c.10315C>G | p.Arg3439Gly | NA | NA | NA | mutation? | 0.02 | Damaging | 0.02 | Benign | 1 | Polymorphism | 2.045 | Medium | 1.09 | Tolerated | -0.129 | Non-conser | 0.00302 | Conserved |
| 22 | 36 | chr16:2143829 | c.10804G>C | p.Gly3602Arg | NA | NA | NA | mutation? | 0.02 | Damaging | 0.857 | Possibly Damaging | 1 | Polymorphism | 1.61 | Low | 1.36 | Tolerated | 0.771 | Conserved | 0.594 | Conserved |
| 1 | 44 | chr16:2140794 | c.12019C>T | p.Arg4007Cys | NA | highly likely pathogenic | NA | mutation? | 0 | Damaging | 1 | Probably Damaging | 1 | Disease Causing | 2.135 | Medium | -0.77 | Tolerated | 1.669 | Conserved | 3.25 | Conserved |
| 50 | 45 | chr16:2140291_2140289 | c.12439_12441del | p.Lys4147del | NA | NA | NA | mutation? | NA | NA | NA | NA | 0.999 | Disease Causing | NA | NA | NA | NA | NA | NA | NA | NA |
| 24 | 46 | chr16:2140198 | c.12445-3C>G | NA | NA | likely pathogenic | NA | mutation? | NA | NA | NA | NA | 1 | Disease Causing | NA | NA | NA | NA | NA | NA | NA | NA |
| 38 | 8 | chr16:2166623 | c.1626_1628dupGAA | p.Asn543delinsLysAsn | NA | NA | NA | mutation? | NA | NA | NA | NA | 0.999 | Disease Causing | NA | NA | NA | NA | NA | NA | NA | NA |
| 23 | 11 | chr16:2164925_2164924 | c.2099_2100delinsAA | p.Val700Glu | NA | NA | NA | mutation? | NA | NA | NA | NA | 0.621 | Polymorphism | NA | NA | NA | NA | NA | NA | NA | NA |
| 3 | 18 | chr16:2156543_2156532 | c.7345_7356del | p.Thr2449_Gly2452del | NA | NA | NA | mutation? | NA | NA | NA | NA | 0.923 | Disease Causing | NA | NA | NA | NA | NA | NA | NA | NA |
| 4 | 18 | chr16:2156570 | c.7303_7317dup | p.Arg2435_Arg2439dup | NA | NA | NA | mutation? | NA | NA | NA | NA | 0.999 | Polymorphism | NA | NA | NA | NA | NA | NA | NA | NA |
| 12 | 23 | chr16:2153532_2153530 | c.8526_8528delCAC | p.Tyr2842_Thr2843delinsT | NA | NA | NA | mutation? | NA | NA | NA | NA | 0.999 | Disease Causing | NA | NA | NA | NA | NA | NA | NA | NA |
| 31 | 41 | chr16:g.2141795_2141784 | c.11524_11535del | p.Trp3842_Asn3845del | NA | NA | NA | mutation? | NA | NA | NA | NA | 0.849 | Disease Causing | NA | NA | NA | NA | NA | NA | NA | NA |

A nSNV was considered likely pathogenic, when at least three of these algorithms predicted that the variants is probably damaging and when it was predicted as conserved with the conservation prediction algorithms PhyloP and GERP++. All prediction tools were used with default parameters as previously been described [11]. Prediction score thresholds were applied as described in the dbNSFP database documentation (https://sites.google.com/site/jpopgen/dbNSFP). Precalculated predictions were used from dbNSFP v2.5 database. In detail, the following settings were applied:

MutationTaster (M.T.) (http://www.mutationtaster.org), version NA: Prob., probability (values close to 1 indicating a high probability).

SIFT (Sequence) (http://sift.jcvi.org/www/SIFT_seq_submit2.html), version NA: A SIFT score between 0 and 0.05 denotes a damaging variant.

Polyphen-2 HumVar (http://genetics.bwh.harvard.edu/pph2), version 2.2.2: A Polyphen score between 0.909 and 1 denotes a probably damaging variant, a score between 0.447 and 0.908 a possibly damaging variant and a score below 0.446 a benign variant.

MutationAssessor (http://mutationassessor.org), version 2: The FI score predicts the functional impact of amino acid substitutions, with high FI scores denoting more severe impact.

FATHMM (http://fathmm.biocompute.org.uk/inherited.html), version 2.3: For scores below -1.5, the corresponding alterations are predicted as damaging.

NA, not available. For the non-frameshift deletions most prediction tools were not able to predict a pathogenicity; there only the Mutation Taster score is available.
