# Supplementary material for: An Efficient and Comprehensive Strategy for Genetic Diagnostics of Polycystic Kidney Disease
Source: PLoS One. 2015 Feb 3;10(2):e0116680. doi: 10.1371/journal.pone.0116680 (PMC4315576; doi:10.1371/journal.pone.0116680)
Supplement: S7 Table — (PDF) [file pone.0116680.s017.pdf]

Table S7. List of all variants simulated in the duplicated region of *PKD1*.
All variants simulated by Wgsim in the coding region and the exon-intron-boundary (splice site) of all exons in the duplicated *PKD1* region (exon 1-33) are listed with their exact position and annotation by the bioinformatic pipeline including sequencing statistics. m – match (correct detection of variant; 100% concordance of variant simulation and NGS result); d - detected (incorrectly detected: below 20% reads-threshold, wrong zygosity); Ref – reference alleles; Alt - alternative alleles.

WGSIM							Bioinformatic pipeline							
Chromosome	Location	Hg19 Position	Ref. Nuc.	Alt. Nuc.	Strand	Comment	cDNA	Protein	Ref. Reads	Alt. Reads	Percentage Alt. Reads	Zygosity	Detected	Comment
16	E1	2185636	G	C	-	exonic	c.55C>G	p.Leu19Val	8	1211	99.34%	hom.	m	
16	E1	2185587	G	R	+	exonic	c.104C>T	p.Pro35Leu	456	438	48.99%	het.	m	
16	E1	2185580	G	R	+	exonic	c.111C>T	p.Cys37Cys	413	423	50.60%	het.	m	
16	E1	2185534	C	Y	+	exonic	c.157G>A	p.Gly53Ser	253	226	47.18%	het.	m	
16	E1	2185490	C	M	+	exonic	c.201G>T	p.Ala67Ala	128	79	38.16%	het.	m	
16	E2	2169376	G	K	+	exonic	c.219C>A	p.Asp73Glu	582	452	43.71%	het.	m	
16	E2	2169372	A	-	-	exonic	c.223delT	p.Ser75fs	512	437	46.05%	het.	m	
16	E2	2169344	A	M	+	exonic	c.251T>G	p.Val84Gly	671	526	43.94%	het.	m	
16	E2	2169320	G	C	-	exonic	c.275C>G	p.Ala92Gly	350	969	73.46%	het.	m	
16	E3	2169181	A	M	+	exonic	c.293T>G	p.Ile98Arg	836	544	39.42%	het.	m	
16	E3	2169164	A	G	-	exonic	c.310T>C	p.Ser104Pro	199	1105	84.74%	het.	m	
16	E3	2169143	A	M	+	exonic	c.331T>G	p.Phe111Val	642	539	45.64%	het.	m	
16	E3	2169119	-	C	+	exonic	c.355_356insG	p.Glu119fs	517	520	50.14%	het.	m	
16	E4	2168820	C	M	+	exonic	c.386G>T	p.Cys129Phe	543	474	46.61%	het.	m	
16	E4	2168788	C	A	-	exonic	c.418G>T	p.Ala140Ser	76	998	92.92%	prob. hom.	m	
16	E4	2168710	G	R	+	exonic	c.496C>T	p.Leu166Leu	555	437	44.05%	het.	m	
16	E4	2168706	A	R	+	exonic	c.500T>C	p.Leu167Pro	545	429	44.05%	het.	m	
16	E4	2168688	T	G	-	exonic	c.518A>C	p.Asp173Ala	64	863	93.10%	prob. hom.	m	
16	E5	2168433	T	Y	+	exonic	c.560A>G	p.Asn187Ser	401	146	26.69%	het.	m	
16	E5	2168428	A	R	+	exonic	c.565T>C	p.Ser189Pro	441	158	26.38%	het.	m	
16	E5	2168304	C	Y	+	exonic	c.689G>A	p.Cys230Tyr	935	789	45.77%	het.	m	
16	E5	2168167	T	W	+	exonic	c.826A>T	p.Thr276Ser	1076	922	46.15%	het.	m	
16	E5	2168151	T	K	+	exonic	c.842A>C	p.His281Pro	1068	923	46.36%	het.	m	
16	E5	2168068	-	TC	-	exonic	c.926_927insGA	p.Asp309fs	1	1740	99.94%	hom.	m	
16	E5	2168007	C	Y	+	exonic	c.986G>A	p.Gly329Glu	969	803	45.32%	het.	m	
16	E5	2168000	A	T	-	exonic	c.993T>A	p.Tyr331*	131	1620	92.52%	prob. hom.	m	
16	E5	2167982	C	S	+	exonic	c.1011G>C	p.Leu337Leu	966	735	43.21%	het.	m	
16	E5	2167930	C	S	+	exonic	c.1063G>C	p.Ala355Pro	958	589	38.07%	het.	m	
16	E5	2167920	-	A	+	exonic	c.1072_1073insT	p.Ala358fs	279	405	59.21%	het.	m	
16	E5	2167888	T	K	+	exonic	c.1105A>C	p.Ser369Arg	988	386	28.09%	het.	m	
16	E5	2167838	C	S	+	exonic	c.1155G>C	p.Leu385Leu	955	175	15.49%	het.	d	Below 20% threshold
16	E5	2167801	-	G	+	exonic	c.1193_1194insC	p.Pro398fs	59	53	47.32%	het.	m	
16	E6	2167637	A	C	-	exonic	c.1238T>G	p.Phe413Cys	8	995	99.20%	hom.	m	
16	E6	2167602	C	M	+	exonic	c.1273G>T	p.Glu425*	530	549	50.88%	het.	m	
16	E6	2167500	G	R	+	exonic	c.1375C>T	p.Arg459Trp	451	437	49.21%	het.	m	
16	E7	2167035	-	C	-	exonic	c.1406_1407insG	p.Gly469fs	0	943	100.00%	hom.	m	
16	E7	2166943	-	C	+	exonic	c.1497_1498insG	p.Pro500fs	597	534	47.21%	het.	m	
16	E7	2166922	C	A	-	exonic	c.1518G>T	p.Glu506Asp	107	1163	91.57%	prob. hom.	m	
16	E7	2166886	G	S	+	exonic	c.1554C>G	p.Asn518Lys	629	558	47.01%	het.	m	
16	E7	2166843	G	K	+	exonic	c.1597C>A	p.Gln533Lys	463	448	49.18%	het.	m	
16	E8	2166631	C	S	+	exonic	c.1621G>C	p.Ala541Pro	520	505	49.27%	het.	m	
16	E8	2166606	G	R	+	exonic	c.1646C>T	p.Pro549Leu	533	555	51.01%	het.	m	
16	E8	2166544	G	S	+	exonic	c.1708C>G	p.His570Asp	550	493	47.27%	het.	m	

16	E8	2166540	T	Y	+	exonic	c.1712A>G	p.Glu571Gly	483	547	53.11%	het.	m	
16	E9	2166104	C	M	+	exonic	c.1738G>T	p.Gly580Cys	590	492	45.47%	het.	m	
16	E9	2166091	C	M	+	exonic	c.1751G>T	p.Ser584Ile	641	463	41.94%	het.	m	
16	E9	2166059	C	S	+	exonic	c.1783G>C	p.Gly595Arg	644	474	42.40%	het.	m	
16	E9	2166017	C	Y	+	exonic	c.1825G>A	p.Val609Met	597	470	44.05%	het.	m	
16	E9	2166008	G	R	+	exonic	c.1834C>T	p.Leu612Phe	573	461	44.58%	het.	m	
16	E10	2165570	G	K	+	exonic	c.1906C>A	p.Leu636Met	623	601	49.10%	het.	m	
16	E10	2165563	G	A	-	exonic	c.1913C>T	p.Pro638Leu	0	1257	100.00%	hom.	m	
16	E10	2165528	C	M	+	exonic	c.1948G>T	p.Ala650Ser	668	682	50.52%	het.	m	
16	E10	2165514	C	A	-	exonic	c.1962G>T	p.Leu654Phe	0	1368	100.00%	hom.	m	
16	E10	2165509	A	R	+	exonic	c.1967T>C	p.Leu656Pro	709	673	48.70%	het.	m	
16	E10	2165468	T	W	+	exonic	c.2008A>T	p.Thr670Ser	718	655	47.71%	het.	m	
16	E10	2165429	A	M	+	exonic	c.2047T>G	p.Trp683Gly	656	585	47.14%	het.	m	
16	E11	2164830	C	-	-	exonic	c.2194delG	p.Ala732fs	543	516	48.73%	het.	m	
16	E11	2164754	T	K	+	exonic	c.2270A>C	p.Gln757Pro	830	826	49.88%	het.	m	
16	E11	2164752	G	K	+	exonic	c.2272C>A	p.Leu758Met	832	826	49.82%	het.	m	
16	E11	2164727	G	C	-	exonic	c.2297C>G	p.Ala766Gly	0	1769	100.00%	hom.	m	
16	E11	2164689	T	K	+	exonic	c.2335A>C	p.Thr779Pro	914	894	49.45%	het.	m	
16	E11	2164686	C	S	+	exonic	c.2338G>C	p.Val780Leu	903	893	49.72%	het.	m	
16	E11	2164599	G	T	-	exonic	c.2425C>A	p.His809Asn	2	1842	99.89%	hom.	m	
16	E11	2164565	G	T	-	exonic	c.2459C>A	p.Pro820Gln	2	1827	99.89%	hom.	m	
16	E11	2164499	-	CG	+	exonic	c.2524_2525insCG	p.Gly842fs	494	890	64.31%	het.	m	
16	E11	2164449	C	-	-	exonic	c.2575delG	p.Ala859fs	928	905	49.37%	het.	m	
16	E11	2164394	A	T	-	exonic	c.2630T>A	p.Leu877Gln	0	1765	100.00%	hom.	m	
16	E11	2164384	G	A	-	exonic	c.2640C>T	p.Thr880Thr	0	1753	100.00%	hom.	m	
16	E11	2164367	G	C	-	exonic	c.2657C>G	p.Pro886Arg	2	1660	99.88%	hom.	m	
16	E11	2164309	C	T	-	exonic	c.2715G>A	p.Gly905Gly	0	1428	100.00%	hom.	m	
16	E11	2164288	C	M	+	exonic	c.2736G>T	p.Val912Val	679	578	45.98%	het.	m	
16	E11	2164273	G	C	-	exonic	c.2751C>G	p.Ala917Ala	0	1076	100.00%	hom.	m	
16	E11	2164222	-	A	+	exonic	c.2801_2802insT	p.Gly934fs	352	305	46.42%	het.	m	
16	E11	2164181	C	M	+	exonic	c.2843G>T	p.Gly948Val	141	86	37.89%	het.	m	
16	E12	2163288	G	R	+	exonic	c.2859C>T	p.Tyr953Tyr	490	512	51.10%	het.	m	
16	E12	2163270	G	R	+	exonic	c.2877C>T	p.Ala959Ala	558	470	45.72%	het.	m	
16	E12	2163264	C	M	+	exonic	c.2883G>T	p.Ser961Ser	515	523	50.39%	het.	m	
16	E12	2163248	A	C	-	exonic	c.2899T>G	p.Trp967Gly	31	1044	97.12%	hom.	m	
16	E12	2163238	T	-	-	exonic	c.2909delA	p.Asn970fs	492	539	52.28%	het.	m	
16	E12	2163165	G	S	+	exonic	c.2982C>G	p.Leu994Leu	501	436	46.53%	het.	m	
16	E13	2162963	A	R	+	exonic	c.2987T>C	p.Leu996Pro	397	435	52.28%	het.	m	
16	E13	2162879	A	R	+	exonic	c.3071T>C	p.Val1024Ala	552	502	47.63%	het.	m	
16	E13	2162840	A	R	+	exonic	c.3110T>C	p.Leu1037Pro	520	525	50.24%	het.	m	
16	E13	2162794	G	S	+	exonic	c.3156C>G	p.Ala1052Ala	446	468	51.20%	het.	m	
16	E14	2162471	CC	-	-	exonic	c.3164_3165delCC	p.1055_1055del	453	483	51.60%	het.	m	
16	E14	2162427	G	K	+	exonic	c.3209C>A	p.Pro1070Gln	552	500	47.53%	het.	m	
16	E14	2162398	G	A	-	exonic	c.3238C>T	p.Pro1080Ser	0	1052	100.00%	hom.	m	
16	E14	2162391	A	G	-	exonic	c.3245T>C	p.Val1082Ala	0	1058	100.00%	hom.	m	
16	E14	2162363	G	C	-	exonic	c.3273C>G	p.Val1091Val	0	1022	100.00%	hom.	m	
16	E15	2161864	G	S	+	exonic	c.3304C>G	p.Leu1102Val	97	72	42.60%	het.	m	
16	E15	2161809	-	CT	-	exonic	c.3358_3359insAG	p.Val1120fs	6	613	99.03%	hom.	m	
16	E15	2161787	G	T	-	exonic	c.3381C>A	p.Pro1127Pro	2	868	99.77%	hom.	m	
16	E15	2161766	A	M	+	exonic	c.3402T>G	p.Ser1134Arg	514	582	53.10%	het.	m	
16	E15	2161652	C	Y	+	exonic	c.3516G>A	p.Gln1172Gln	860	688	44.44%	het.	m	
16	E15	2161649	G	R	+	exonic	c.3519C>T	p.Ser1173Ser	857	697	44.85%	het.	m	
16	E15	2161648	G	K	+	exonic	c.3520C>A	p.Gln1174Lys	721	855	54.25%	het.	m	
16	E15	2161515	T	-	-	exonic	c.3653delA	p.Asp1218fs	0	1419	100.00%	hom.	m	
16	E15	2161512	A	W	+	exonic	c.3656T>A	p.Met1219Lys	741	697	48.47%	het.	m	
16	E15	2161391	T	Y	+	exonic	c.3777A>G	p.Thr1259Thr	765	735	49.00%	het.	m	
16	E15	2161338	G	R	+	exonic	c.3830C>T	p.Ala1277Val	762	721	48.62%	het.	m	
16	E15	2161251	G	C	-	exonic	c.3917C>G	p.Pro1306Arg	0	1494	100.00%	hom.	m	

16	E15	2161230	A	M	+	exonic	c.3938T>G	p.Leu1313Arg	745	706	48.66%	het.	m	
16	E15	2161033	C	S	+	exonic	c.4135G>C	p.Glu1379Gln	768	788	50.64%	het.	m	
16	E15	2160935	G	A	-	exonic	c.4233C>T	p.Arg1411Arg	0	1522	100.00%	hom.	m	
16	E15	2160913	C	M	+	exonic	c.4255G>T	p.Glu1419*	769	702	47.72%	het.	m	
16	E15	2160845	A	R	+	exonic	c.4323T>C	p.Tyr1441Tyr	766	717	48.35%	het.	m	
16	E15	2160775	G	K	+	exonic	c.4393C>A	p.Pro1465Thr	764	749	49.50%	het.	m	
16	E15	2160755	G	C	-	exonic	c.4413C>G	p.Ile1471Met	10	1521	99.35%	hom.	m	
16	E15	2160725	C	M	+	exonic	c.4443G>T	p.Leu1481Leu	754	812	51.85%	het.	m	
16	E15	2160724	G	K	+	exonic	c.4444C>A	p.Gln1482Lys	823	741	47.38%	het.	m	
16	E15	2160691	-	TG	+	exonic	c.4476_4477insCA	p.Gly1493fs	437	740	62.87%	het.	m	
16	E15	2160551	-	T	-	exonic	c.4616_4617insA	p.Trp1539_1540*	0	1519	100.00%	hom.	m	
16	E15	2160426	G	K	+	exonic	c.4742C>A	p.Ser1581Tyr	783	806	50.72%	het.	m	
16	E15	2160157	C	Y	+	exonic	c.5011G>A	p.Asp1671Asn	787	728	48.05%	het.	m	
16	E15	2160153	C	T	-	exonic	c.5015G>A	p.Arg1672Lys	0	1523	100.00%	hom.	m	
16	E15	2160142	G	R	+	exonic	c.5026C>T	p.Leu1676Leu	792	743	48.40%	het.	m	
16	E15	2160115	G	R	+	exonic	c.5053C>T	p.Leu1685Phe	798	751	48.48%	het.	m	
16	E15	2160000	A	T	-	exonic	c.5168T>A	p.Val1723Glu	4	1504	99.73%	hom.	m	
16	E15	2159993	G	K	+	exonic	c.5175C>A	p.Ala1725Ala	802	722	47.38%	het.	m	
16	E15	2159882	G	C	-	exonic	c.5286C>G	p.Thr1762Thr	0	1525	100.00%	hom.	m	
16	E15	2159869	T	K	+	exonic	c.5299A>C	p.Thr1767Pro	741	767	50.86%	het.	m	
16	E15	2159854	G	R	+	exonic	c.5314C>T	p.Pro1772Ser	765	741	49.20%	het.	m	
16	E15	2159799	-	ACT	+	exonic	c.5368_5369insAGT	p.Ala1790delinsGluSer	402	755	65.25%	het.	m	
16	E15	2159633	G	S	+	exonic	c.5535C>G	p.Ser1845Arg	801	703	46.74%	het.	m	
16	E15	2159613	A	M	+	exonic	c.5555T>G	p.Val1852Gly	706	789	52.78%	het.	m	
16	E15	2159608	TG	-	-	exonic	c.5559_5560delTG	p.1853_1854del	789	640	44.79%	het.	m	
16	E15	2159605	C	S	+	exonic	c.5563G>C	p.Val1855Leu	798	654	45.04%	het.	m	
16	E15	2159596	C	S	+	exonic	c.5572G>C	p.Asp1858His	779	734	48.51%	het.	m	
16	E15	2159531	G	C	-	exonic	c.5637C>G	p.Tyr1879*	0	1506	100.00%	hom.	m	
16	E15	2159527	G	K	+	exonic	c.5641C>A	p.Leu1881Ile	779	727	48.27%	het.	m	
16	E15	2159361	A	C	-	exonic	c.5807T>G	p.Phe1936Cys	3	1608	99.81%	hom.	m	
16	E15	2159326	C	-	-	exonic	c.5842delG	p.Val1948*	848	718	45.85%	het.	m	
16	E15	2159321	G	S	+	exonic	c.5847C>G	p.Ser1949Arg	852	748	46.75%	het.	m	
16	E15	2159284	-	T	+	exonic	c.5884dupA	p.Gln1962fs	374	797	68.06%	het.	m	
16	E15	2159226	T	K	+	exonic	c.5942A>C	p.Glu1981Ala	785	787	50.06%	het.	m	
16	E15	2159088	G	K	+	exonic	c.6080C>A	p.Thr2027Asn	782	745	48.79%	het.	m	
16	E15	2159084	G	K	+	exonic	c.6084C>A	p.Tyr2028*	791	751	48.70%	het.	m	
16	E15	2159038	T	G	-	exonic	c.6130A>C	p.Asn2044His	0	1561	100.00%	hom.	m	
16	E15	2158818	G	K	+	exonic	c.6350C>A	p.Pro2117His	760	752	49.74%	het.	m	
16	E15	2158774	A	R	+	exonic	c.6394T>C	p.Phe2132Leu	722	776	51.80%	het.	m	
16	E15	2158756	T	W	+	exonic	c.6412A>T	p.Thr2138Ser	772	738	48.87%	het.	m	
16	E15	2158639	-	C	+	exonic	c.6529_6530insG	p.Asp2177fs	722	710	49.58%	het.	m	
16	E15	2158612	G	R	+	exonic	c.6556C>T	p.Arg2186Cys	731	735	50.14%	het.	m	
16	E15	2158502	C	A	-	exonic	c.6666G>T	p.Ala2222Ala	1	1549	99.94%	hom.	m	
16	E15	2158480	A	-	-	exonic	c.6690delT	p.Phe2230fs	770	729	48.63%	het.	m	
16	E15	2158452	-	C	+	exonic	c.6716dupG	p.Thr2239fs	359	748	67.57%	het.	m	
16	E15	2158401	A	M	+	exonic	c.6767T>G	p.Leu2256Arg	665	688	50.85%	het.	m	
16	E15	2158293	G	C	-	exonic	c.6875C>G	p.Thr2292Arg	0	473	100.00%	hom.	m	
16	E16	2158017	C	Y	+	exonic	c.6932G>A	p.Cys2311Tyr	447	527	54.11%	het.	m	
16	E16	2157979	-	GC	-	exonic	c.6969_6970insGC	p.Thr2324fs	19	1022	98.17%	hom.	m	
16	E16	2157918	-	AG	+	exonic	c.7030_7031insCT	p.Lys2344fs	341	474	58.16%	het.	m	
16	E16	2157892	T	W	+	exonic	c.7057A>T	p.Asn2353Tyr	509	456	47.25%	het.	m	
16	E17	2156954	C	M	+	splice site	c.7066-5G>T	-	471	435	48.01%	het.	m	
16	E17	2156925	G	R	+	exonic	c.7090C>T	p.Pro2364Ser	520	478	47.90%	het.	m	
16	E17	2156921	A	M	+	exonic	c.7094T>G	p.Ile2365Ser	487	519	51.59%	het.	m	
16	E17	2156900	G	R	+	exonic	c.7115C>T	p.Ser2372Phe	520	523	50.14%	het.	m	
16	E17	2156825	C	S	+	exonic	c.7190G>C	p.Ser2397Thr	535	467	46.61%	het.	m	
16	E17	2156821	G	R	+	exonic	c.7194C>T	p.Ser2398Ser	499	493	49.70%	het.	m	
16	E18	2156655	G	C	-	exonic	c.7233C>G	p.Ser2411Arg	34	979	96.64%	hom.	m	

16	E18	2156644	A	W	+	exonic	c.7244T>A	p.Leu2415Gln	544	550	50.27%	het.	m	
16	E18	2156604	G	R	+	exonic	c.7284C>T	p.Gly2428Gly	765	698	47.71%	het.	m	
16	E18	2156566	C	G	-	exonic	c.7322G>C	p.Gly2441Ala	1	1584	99.94%	hom.	m	
16	E18	2156527	G	R	+	exonic	c.7361C>T	p.Ser2454Phe	777	823	51.44%	het.	m	
16	E18	2156440	G	R	+	exonic	c.7448C>T	p.Ala2483Val	580	519	47.22%	het.	m	
16	E18	2156437	A	M	+	exonic	c.7451T>G	p.Val2484Gly	570	504	46.93%	het.	m	
16	E19	2156279	C	T	-	exonic	c.7516G>A	p.Ala2506Thr	0	1066	100.00%	hom.	m	
16	E19	2156244	-	C	+	exonic	c.7550_7551insG	p.Cys2517fs	298	649	68.53%	het.	m	
16	E19	2156237	C	M	+	exonic	c.7558G>T	p.Gly2520Cys	674	593	46.80%	het.	m	
16	E19	2156226	C	M	+	exonic	c.7569G>T	p.Glu2523Asp	691	604	46.64%	het.	m	
16	E19	2156222	A	G	-	exonic	c.7573T>C	p.Phe2525Leu	0	1299	100.00%	hom.	m	
16	E19	2156133	-	TC	+	exonic	c.7662_7663insAG	p.Val2555fs	733	526	41.78%	het.	m	
16	E19	2156088	T	W	+	splice site	c.7703+4A>T	-	862	375	30.32%	het.	m	
16	E20	2156023	G	C	-	exonic	c.7706C>G	p.Ser2569Cys	302	938	75.65%	het.	m	
16	E20	2156016	G	R	+	exonic	c.7713C>T	p.Ala2571Ala	764	455	37.33%	het.	m	
16	E20	2155968	C	Y	+	exonic	c.7761G>A	p.Trp2587*	522	540	50.85%	het.	m	
16	E20	2155890	C	Y	+	exonic	c.7839G>A	p.Leu2613Leu	489	498	50.46%	het.	m	
16	E20	2155878	G	S	+	exonic	c.7851C>G	p.Thr2617Thr	492	473	49.02%	het.	m	
16	E21	2155435	T	A	-	exonic	c.7904A>T	p.Glu2635Val	1	1063	99.91%	hom.	m	
16	E21	2155427	G	R	+	exonic	c.7912C>T	p.His2638Tyr	513	571	52.68%	het.	m	
16	E21	2155356	G	-	-	exonic	c.7984delC	p.Gln2662fs	497	519	51.08%	het.	m	
16	E22	2154643	C	G	-	exonic	c.8017G>C	p.Gly2673Arg	167	886	84.14%	het.	m	
16	E22	2154606	T	W	+	exonic	c.8054A>T	p.Lys2685Met	611	518	45.88%	het.	m	
16	E22	2154555	G	K	+	exonic	c.8105C>A	p.Thr2702Asn	548	558	50.45%	het.	m	
16	E22	2154547	CC	-	-	exonic	c.8113_8114delCC	p.2705_2705del	543	529	49.35%	het.	m	
16	E22	2154514	T	G	-	exonic	c.8146A>C	p.Ile2716Leu	0	1009	100.00%	hom.	m	
16	E23	2153881	A	R	+	exonic	c.8177T>C	p.Leu2726Pro	123	121	49.59%	het.	m	
16	E23	2153816	C	Y	+	exonic	c.8242G>A	p.Val2748Met	469	437	48.23%	het.	m	
16	E23	2153685	C	Y	+	exonic	c.8373G>A	p.Arg2791Arg	923	939	50.43%	het.	m	
16	E23	2153640	G	R	+	exonic	c.8418C>T	p.Phe2806Phe	1003	937	48.30%	het.	m	
16	E23	2153627	CC	-	-	exonic	c.8430_8431delCC	p.2810_2811del	911	992	52.13%	het.	m	
16	E23	2153509	G	-	-	exonic	c.8549delC	p.Ser2850*	914	983	51.82%	het.	m	
16	E23	2153438	C	S	+	exonic	c.8620G>C	p.Val2874Leu	886	914	50.78%	het.	m	
16	E23	2153277	C	Y	+	exonic	c.8781G>A	p.Thr2927Thr	128	105	45.06%	het.	m	
16	E24	2152952	T	K	+	exonic	c.8811A>C	p.Glu2937Asp	469	480	50.58%	het.	m	
16	E24	2152903	C	G	-	exonic	c.8860G>C	p.Glu2954Gln	1	1021	99.90%	hom.	m	
16	E24	2152899	T	-	-	exonic	c.8864delA	p.His2955fs	521	500	48.97%	het.	m	
16	E24	2152893	C	Y	+	exonic	c.8870G>A	p.Cys2957Tyr	539	525	49.34%	het.	m	
16	E24	2152842	C	M	+	exonic	c.8921G>T	p.Arg2974Leu	531	501	48.55%	het.	m	
16	E25	2152630	T	W	+	exonic	c.8953A>T	p.Arg2985*	381	391	50.65%	het.	m	
16	E25	2152592	G	S	+	exonic	c.8991C>G	p.Ser2997Arg	580	558	49.03%	het.	m	
16	E25	2152587	A	T	-	exonic	c.8996T>A	p.Phe2999Tyr	0	1199	100.00%	hom.	m	
16	E25	2152518	T	K	+	exonic	c.9065A>C	p.Asp3022Ala	767	717	48.32%	het.	m	
16	E25	2152480	-	C	+	exonic	c.9104dupG	p.Glu3035fs	688	731	51.52%	het.	m	
16	E25	2152458	A	R	+	exonic	c.9125T>C	p.Val3042Ala	746	660	46.94%	het.	m	
16	E25	2152420	G	R	+	exonic	c.9163C>T	p.Leu3055Phe	650	561	46.33%	het.	m	
16	E26	2152206	G	R	+	exonic	c.9253C>T	p.Leu3085Leu	705	474	40.20%	het.	m	
16	E26	2152168	C	A	-	exonic	c.9291G>T	p.Lys3097Asn	100	1078	91.51%	prob. hom.	m	
16	E26	2152166	A	M	+	exonic	c.9293T>G	p.Leu3098Arg	657	513	43.85%	het.	m	
16	E26	2152148	C	A	-	exonic	c.9311G>T	p.Ser3104Ile	51	1125	95.66%	hom.	m	
16	E26	2152095	T	G	-	exonic	c.9364A>C	p.Ile3122Leu	0	1052	100.00%	hom.	m	
16	E27	2150553	C	M	+	exonic	c.9412G>T	p.Val3138Leu	494	450	47.67%	het.	m	
16	E27	2150545	G	K	+	exonic	c.9420C>A	p.Ile3140Ile	503	461	47.82%	het.	m	
16	E27	2150416	T	K	+	exonic	c.9549A>C	p.Arg3183Arg	685	493	41.85%	het.	m	
16	E27	2150410	C	S	+	exonic	c.9555G>C	p.Trp3185Cys	744	442	37.27%	het.	m	
16	E28	2150287	G	R	+	exonic	c.9592C>T	p.Gln3198*	705	490	41.00%	het.	m	
16	E28	2150233	T	Y	+	exonic	c.9646A>G	p.Asn3216Asp	528	550	51.02%	het.	m	
16	E28	2150189	C	Y	+	exonic	c.9690G>A	p.Val3230Val	546	495	47.55%	het.	m	

16	E28	2150180	C	Y	+	exonic	c.9699G>A	p.Glu3233Glu	554	508	47.83%	het.	m	
16	E29	2150050	G	S	+	exonic	c.9735C>G	p.Phe3245Leu	674	450	40.04%	het.	m	
16	E29	2150016	A	R	+	exonic	c.9769T>C	p.Phe3257Leu	623	495	44.28%	het.	m	
16	E29	2150001	T	C	-	exonic	c.9784A>G	p.Ile3262Val	0	1151	100.00%	hom.	m	
16	E29	2149987	T	Y	+	exonic	c.9798A>G	p.Ile3266Met	651	535	45.11%	het.	m	
16	E29	2149931	A	M	+	exonic	c.9854T>G	p.Val3285Gly	538	617	53.42%	het.	m	
16	E29	2149926	G	T	-	exonic	c.9859C>A	p.Leu3287Ile	39	1122	96.64%	hom.	m	
16	E29	2149912	G	A	-	exonic	c.9873C>T	p.Phe3291Phe	102	1094	91.47%	prob. hom.	m	
16	E29	2149872	A	W	+	exonic	c.9913T>A	p.Ser3305Thr	731	430	37.04%	het.	m	
16	E30	2149774	G	K	+	splice site	c.9924-3C>A	-	633	483	43.28%	het.	m	
16	E30	2149758	A	G	-	exonic	c.9937T>C	p.Ser3313Pro	115	961	89.31%	prob. hom.	m	
16	E30	2149695	A	R	+	exonic	c.10000T>C	p.Tyr3334His	556	503	47.50%	het.	m	
16	E30	2149654	G	S	+	exonic	c.10041C>G	p.Ser3347Ser	500	495	49.75%	het.	m	
16	E30	2149651	C	Y	+	exonic	c.10044G>A	p.Arg3348Arg	492	495	50.15%	het.	m	
16	E31	2147990	A	W	+	splice site	c.10051-5T>A	-	485	512	51.35%	het.	m	
16	E31	2147964	T	K	+	exonic	c.10072A>C	p.Thr3358Pro	592	508	46.18%	het.	m	
16	E31	2147961	G	S	+	exonic	c.10075C>G	p.Pro3359Ala	608	507	45.47%	het.	m	
16	E31	2147930	C	T	-	exonic	c.10106G>A	p.Ser3369Asn	269	1036	79.39%	het.	m	
16	E31	2147899	G	K	+	exonic	c.10137C>A	p.Ser3379Ser	991	495	33.31%	het.	m	
16	E31	2147871	-	A	-	exonic	c.10164_10165insT	p.Glu3389_3390*	91	949	91.25%	prob. hom.	m	
16	E32	2147748	A	W	+	exonic	c.10201T>A	p.Phe3401Ile	749	517	40.84%	het.	m	
16	E33	2147492	C	Y	+	exonic	c.10233G>A	p.Trp3411*	461	438	48.72%	het.	m	
16	E33	2147380	C	Y	+	exonic	c.10345G>A	p.Glu3449Lys	546	536	49.54%	het.	m	
16	E33	2147340	G	R	+	exonic	c.10385C>T	p.Ala3462Val	465	509	52.26%	het.	m	
