# Supplementary material for: An Efficient and Comprehensive Strategy for Genetic Diagnostics of Polycystic Kidney Disease
Source: PLoS One. 2015 Feb 3;10(2):e0116680. doi: 10.1371/journal.pone.0116680 (PMC4315576; doi:10.1371/journal.pone.0116680)
Supplement: S8 Table — (PDF) [file pone.0116680.s018.pdf]

Table S8. Results from CNV analysis.

The results of CNV analysis using VarScan are listed for the two positive control samples (patient 30 and 46) as well as for remaining false positive (FP) calls after filtering. Percentage - % of the control patients against who the CNV is detected in mutual comparison. The initial stringent threshold was set at 85%, in a second-step analysis a less stringent threshold of 50% was applied. TP- true positive.

patient- no	chr.	NM number	exon	CNV effect	gene	control read depth	probe read depth	percentage	classification	comment
11	16	NM_000296	38	del	PKD1	3991	1904.4	100.00%	FP	detection of 9bp-deletion c.11096_11104del in this exon
31	16	NM_000296	41	del	PKD1	3651.6	1794.5	100.00%	FP	detection of 12bp-deletion c.11524_11535del in this exon
30	16	NM_000296	22	del	PKD1	3836.5	1214.4	100.00%	TP	clearly detected
43	16	NM_000296	9	del	PKD1	1292.8	289.7	52.17%	FP	MLPA negative, low stringent threshold
46	16	NM_000296	15	del	PKD1	5891.3	1783.6	100.00%	TP	clearly detected
46	16	NM_000296	16	del	PKD1	3875.7	1533.1	52.17%	TP	detected only with less stingent criteria
46	16	NM_000296	17	del	PKD1	3649.1	1193.2	69.57%	TP	detected only with less stingent criteria
46	16	NM_000296	19	del	PKD1	4403.6	1445.1	78.26%	TP	detected only with less stingent criteria
46	16	NM_000296	21	del	PKD1	4698.1	1480.9	52.17%	TP	detected only with less stingent criteria
