# Supplementary material for: An Efficient and Comprehensive Strategy for Genetic Diagnostics of Polycystic Kidney Disease
Source: PLoS One. 2015 Feb 3;10(2):e0116680. doi: 10.1371/journal.pone.0116680 (PMC4315576; doi:10.1371/journal.pone.0116680)
Supplement: S9 Table — (PDF) [file pone.0116680.s019.pdf]

Table S9. Coverage statistics for a proof-of-principle ultra-deep sequencing setup for *PKD1*. Coverage statistics of a proof-of-principle ultra-deep sequencing experiment including five patients sequenced on a MiSeq system. Coverage of more than 2000x can be guaranteed for the vast majority of *PKD1* exons as a prerequisite for efficient mosaic detection. Coverage might be further increased when sequencing on a HiSeq platform. Bioinformatic calculation of the coverage statistics has been performed by GATK.

PKD1 exon	1	2	3	4	5	6	7	8	9	10	11	12	13	14	15	16	17	18	19	20	21	22	23	24	25	26	27	28	29	30	31	32	33	34	35	36	37	38	39	40	41	42	43	44	45	46	# exons covered > 90%	total mean coverage
**20X Coverage (%)**																																																
Patient A	100%	100%	100%	100%	100%	100%	100%	100%	100%	100%	100%	100%	100%	100%	100%	100%	100%	100%	100%	100%	100%	100%	100%	100%	100%	100%	100%	100%	100%	100%	100%	100%	100%	100%	100%	100%	100%	100%	100%	100%	100%	100%	100%	100%	100%	100%	46	100%
Patient B	100%	100%	100%	100%	100%	100%	100%	100%	100%	100%	100%	100%	100%	100%	100%	100%	100%	100%	100%	100%	100%	100%	100%	100%	100%	100%	100%	100%	100%	100%	100%	100%	100%	100%	100%	100%	100%	100%	100%	100%	100%	100%	100%	100%	100%	100%	46	100%
Patient C	100%	100%	100%	100%	100%	100%	100%	100%	100%	100%	100%	100%	100%	100%	100%	100%	100%	100%	100%	100%	100%	100%	100%	100%	100%	100%	100%	100%	100%	100%	100%	100%	100%	100%	100%	100%	100%	100%	100%	100%	100%	100%	100%	100%	100%	100%	46	100%
Patient D	100%	100%	100%	100%	100%	100%	100%	100%	100%	100%	100%	100%	100%	100%	100%	100%	100%	100%	100%	100%	100%	100%	100%	100%	100%	100%	100%	100%	100%	100%	100%	100%	100%	100%	100%	100%	100%	100%	100%	100%	100%	100%	100%	100%	100%	100%	46	100%
Patient E	100%	100%	100%	100%	100%	100%	100%	100%	100%	100%	100%	100%	100%	100%	100%	100%	100%	100%	100%	100%	100%	100%	100%	100%	100%	100%	100%	100%	100%	100%	100%	100%	100%	100%	100%	100%	100%	100%	100%	100%	100%	100%	100%	100%	100%	100%	46	100%
**Min**	100%	100%	100%	100%	100%	100%	100%	100%	100%	100%	100%	100%	100%	100%	100%	100%	100%	100%	100%	100%	100%	100%	100%	100%	100%	100%	100%	100%	100%	100%	100%	100%	100%	100%	100%	100%	100%	100%	100%	100%	100%	100%	100%	100%	100%	100%	46	100%
**Max**	100%	100%	100%	100%	100%	100%	100%	100%	100%	100%	100%	100%	100%	100%	100%	100%	100%	100%	100%	100%	100%	100%	100%	100%	100%	100%	100%	100%	100%	100%	100%	100%	100%	100%	100%	100%	100%	100%	100%	100%	100%	100%	100%	100%	100%	100%	46	100%
**Mean**	100%	100%	100%	100%	100%	100%	100%	100%	100%	100%	100%	100%	100%	100%	100%	100%	100%	100%	100%	100%	100%	100%	100%	100%	100%	100%	100%	100%	100%	100%	100%	100%	100%	100%	100%	100%	100%	100%	100%	100%	100%	100%	100%	100%	100%	100%	46	100%
**100X Coverage (%)**																																																
Patient A	100%	100%	100%	100%	100%	100%	100%	100%	100%	100%	100%	100%	100%	100%	100%	100%	100%	100%	100%	100%	100%	100%	100%	100%	100%	100%	100%	100%	100%	100%	100%	100%	100%	100%	100%	100%	100%	100%	100%	100%	100%	100%	100%	100%	100%	100%	46	100%
Patient B	100%	100%	100%	100%	100%	100%	100%	100%	100%	100%	100%	100%	100%	100%	100%	100%	100%	100%	100%	100%	100%	100%	100%	100%	100%	100%	100%	100%	100%	100%	100%	100%	100%	100%	100%	100%	100%	100%	100%	100%	100%	100%	100%	100%	100%	100%	46	100%
Patient C	100%	100%	100%	100%	100%	100%	100%	100%	100%	100%	100%	100%	100%	100%	100%	100%	100%	100%	100%	100%	100%	100%	100%	100%	100%	100%	100%	100%	100%	100%	100%	100%	100%	100%	100%	100%	100%	100%	100%	100%	100%	100%	100%	100%	100%	100%	46	100%
Patient D	100%	100%	100%	100%	100%	100%	100%	100%	100%	100%	100%	100%	100%	100%	100%	100%	100%	100%	100%	100%	100%	100%	100%	100%	100%	100%	100%	100%	100%	100%	100%	100%	100%	100%	100%	100%	100%	100%	100%	100%	100%	100%	100%	100%	100%	100%	46	100%
Patient E	100%	100%	100%	100%	100%	100%	100%	100%	100%	100%	100%	100%	100%	100%	100%	100%	100%	100%	100%	100%	100%	100%	100%	100%	100%	100%	100%	100%	100%	100%	100%	100%	100%	100%	100%	100%	100%	100%	100%	100%	100%	100%	100%	100%	100%	100%	46	100%
**Min**	100%	100%	100%	100%	100%	100%	100%	100%	100%	100%	100%	100%	100%	100%	100%	100%	100%	100%	100%	100%	100%	100%	100%	100%	100%	100%	100%	100%	100%	100%	100%	100%	100%	100%	100%	100%	100%	100%	100%	100%	100%	100%	100%	100%	100%	100%	46	100%
**Max**	100%	100%	100%	100%	100%	100%	100%	100%	100%	100%	100%	100%	100%	100%	100%	100%	100%	100%	100%	100%	100%	100%	100%	100%	100%	100%	100%	100%	100%	100%	100%	100%	100%	100%	100%	100%	100%	100%	100%	100%	100%	100%	100%	100%	100%	100%	46	100%
**Mean**	100%	100%	100%	100%	100%	100%	100%	100%	100%	100%	100%	100%	100%	100%	100%	100%	100%	100%	100%	100%	100%	100%	100%	100%	100%	100%	100%	100%	100%	100%	100%	100%	100%	100%	100%	100%	100%	100%	100%	100%	100%	100%	100%	100%	100%	100%	46	100%
**500X Coverage (%)**																																																
Patient A	100%	100%	100%	100%	100%	100%	100%	100%	100%	100%	100%	100%	100%	100%	100%	100%	100%	100%	100%	100%	100%	100%	100%	100%	100%	100%	100%	100%	100%	100%	100%	100%	100%	100%	100%	100%	100%	100%	100%	100%	100%	100%	100%	100%	100%	100%	46	100%
Patient B	100%	100%	100%	100%	100%	100%	100%	100%	100%	100%	100%	100%	100%	100%	100%	100%	100%	100%	100%	100%	100%	100%	100%	100%	100%	100%	100%	100%	100%	100%	100%	100%	100%	100%	100%	100%	100%	100%	100%	100%	100%	100%	100%	100%	100%	100%	46	100%
Patient C	100%	100%	100%	100%	100%	100%	100%	100%	100%	100%	100%	100%	100%	100%	100%	100%	100%	100%	100%	100%	100%	100%	100%	100%	100%	100%	100%	100%	100%	100%	100%	100%	100%	100%	100%	100%	100%	100%	100%	100%	100%	100%	100%	100%	100%	100%	46	100%
Patient D	100%	100%	100%	100%	100%	100%	100%	100%	100%	100%	100%	100%	100%	100%	100%	100%	100%	100%	100%	100%	100%	100%	100%	100%	100%	100%	100%	100%	100%	100%	100%	100%	100%	100%	100%	100%	100%	100%	100%	100%	100%	100%	100%	100%	100%	100%	46	100%
Patient E	100%	100%	100%	100%	100%	100%	100%	100%	100%	100%	100%	100%	100%	100%	100%	100%	100%	100%	100%	100%	100%	100%	100%	100%	100%	100%	100%	100%	100%	100%	100%	100%	100%	100%	100%	100%	100%	100%	100%	100%	100%	100%	100%	100%	100%	100%	46	100%
**Min**	100%	100%	100%	100%	100%	100%	100%	100%	100%	100%	100%	100%	100%	100%	100%	100%	100%	100%	100%	100%	100%	100%	100%	100%	100%	100%	100%	100%	100%	100%	100%	100%	100%	100%	100%	100%	100%	100%	100%	100%	100%	100%	100%	100%	100%	100%	46	100%
**Max**	100%	100%	100%	100%	100%	100%	100%	100%	100%	100%	100%	100%	100%	100%	100%	100%	100%	100%	100%	100%	100%	100%	100%	100%	100%	100%	100%	100%	100%	100%	100%	100%	100%	100%	100%	100%	100%	100%	100%	100%	100%	100%	100%	100%	100%	100%	46	100%
**Mean**	100%	100%	100%	100%	100%	100%	100%	100%	100%	100%	100%	100%	100%	100%	100%	100%	100%	100%	100%	100%	100%	100%	100%	100%	100%	100%	100%	100%	100%	100%	100%	100%	100%	100%	100%	100%	100%	100%	100%	100%	100%	100%	100%	100%	100%	100%	46	100%
**1000X Coverage (%)**																																																
Patient A	88.0%	100%	100%	100%	100%	100%	100%	100%	100%	100%	100%	100%	100%	100%	100%	100%	100%	100%	100%	100%	100%	100%	100%	100%	100%	100%	100%	100%	100%	100%	100%	100%	100%	100%	100%	100%	100%	100%	100%	100%	100%	100%	100%	100%	100%	100%	45	100%
Patient B	60.9%	100%	100%	100%	100%	100%	100%	100%	100%	100%	100%	100%	100%	100%	100%	100%	100%	100%	100%	100%	100%	100%	100%	100%	100%	81.6%	100%	100%	100%	100%	100%	100%	100%	100%	100%	100%	100%	100%	100%	100%	100%	100%	100%	100%	100%	100%	44	98.7%
Patient C	93.8%	100%	100%	100%	100%	100%	100%	100%	100%	100%	100%	100%	100%	100%	100%	100%	100%	100%	100%	100%	100%	100%	100%	100%	100%	100%	100%	100%	100%	100%	100%	100%	100%	100%	100%	100%	100%	100%	100%	100%	100%	95.7%	100%	100%	100%	100%	46	100%
Patient D	83.1%	100%	100%	100%	100%	100%	100%	100%	100%	100%	100%	100%	100%	100%	100%	100%	100%	100%	100%	100%	100%	100%	100%	100%	100%	100%	100%	100%	100%	100%	100%	100%	100%	100%	100%	100%	100%	100%	100%	100%	100%	100%	100%	100%	100%	100%	45	100%
Patient E	79.1%	100%	100%	100%	100%	100%	100%	100%	100%	100%	100%	100%	100%	100%	100%	100%	100%	100%	100%	100%	100%	100%	100%	100%	100%	100%	100%	100%	100%	100%	100%	100%	100%	100%	100%	100%	100%	100%	100%	100%	100%	98.4%	100%	100%	100%	100%	45	100%
**Min**	60.9%	100%	100%	100%	100%	100%	100%	100%	100%	100%	100%	100%	100%	100%	100%	100%	100%	100%	100%	100%	100%	100%	100%	100%	100%	81.6%	100%	100%	100%	100%	100%	100%	100%	100%	100%	100%	100%	100%	100%	100%	100%	95.7%	100%	100%	100%	100%	44	98.7%
**Max**	93.8%	100%	100%	100%	100%	100%	100%	100%	100%	100%	100%	100%	100%	100%	100%	100%	100%	100%	100%	100%	100%	100%	100%	100%	100%	100%	100%	100%	100%	100%	100%	100%	100%	100%	100%	100%	100%	100%	100%	100%	100%	100%	100%	100%	100%	100%	46	100%
**Mean**	81.0%	100%	100%	100%	100%	100%	100%	100%	100%	100%	100%	100%	100%	100%	100%	100%	100%	100%	100%	100%	100%	100%	100%	100%	100%	96.3%	100%	100%	100%	100%	100%	100%	100%	100%	100%	100%	100%	100%	100%	100%	100%	98.8%	100%	100%	100%	100%	45	99.5%
**2000X Coverage (%)**																																																
Patient A	34.7%	100%	100%	100%	93.0%	100%	100%	100%	100%	100%	100%	93.0%	100%	100%	100%	100%	81.8%	100%	100%	100%	100%	100%	100%	100%	100%	97.1%	100%	100%	100%	100%	100%	100%	100%	100%	100%	100%	100%	100%	100%	100%	100%	82.2%	93.0%	100%	100%	100%	43	97.3%
Patient B	18.2%	100%	100%	100%	93.4%	100%	100%	100%	100%	100%	100%	93.7%	100%	100%	100%	100%	93.5%	100%	100%	100%	100%	100%	61.7%	69.5%	100%	35.0%	63.5%	94.8%	100%	45.3%	100%	100%	100%	100%	100%	100%	100%	100%	100%	100%	100%	74.6%	92.4%	100%	100%	100%	39	92.1%
Patient C	49.3%	100%	100%	100%	93.4%	100%	100%	100%	100%	100%	100%	94.4%	100%	100%	100%	100%	97.4%	100%	100%	100%	100%	100%	100%	100%	100%	95.1%	100%	100%	100%	100%	100%	100%	100%	100%	100%	100%	100%	100%	100%	100%	100%	66.5%	86.4%	100%	100%	100%	43	97.4%
Patient D	40.0%	100%	100%	100%	93.3%	100%	100%	100%	100%	100%	100%	93.7%	100%	100%	100%	100%	72.1%	95.2%	100%	100%	100%	98.1%	100%	100%	100%	87.9%	100%	100%	100%	100%	100%	100%	100%	100%	100%	100%	100%	100%	100%	100%	100%	81.1%	94.7%	100%	100%	100%	42	96.9%
Patient E	34.2%	100%	100%	100%	93.1%	100%	100%	100%	100%	100%	100%	93.7%	100%	100%	100%	100%	87.0%	100%	100%	100%	100%	100%	100%	100%	100%	87.9%	100%	100%	100%	100%	100%	100%	100%	100%	100%	100%	100%	100%	100%	100%	100%	72.4%	88.4%	100%	100%	100%	41	96.9%
**Min**	18.2%	100%	100%	100%	93.0%	100%	100%	100%	100%	100%	100%	93.0%	100%	100%	100%	100%	72.1%	95.2%	100%	100%	100%	98.1%	61.7%	69.5%	100%	35.0%	63.5%	94.8%	100%	45.3%	100%	100%	100%	100%	100%	100%	100%	100%	100%	100%	100%	66.5%	86.4%	100%	100%	100%	39	91.1%
**Max**	49.3%	100%	100%	100%	93.4%	100%	100%	100%	100%	100%	100%	94.4%	100%	100%	100%	100%	97.4%	100%	100%	100%	100%	100%	100%	100%	100%	97.1%	100%	100%	100%	100%	100%	100%	100%	100%	100%	100%	100%	100%	100%	100%	100%	82.2%	94.7%	100%	100%	100%	43	98.0%
**Mean**	35.3%	100%	100%	100%	93.2%	100%	100%	100%	100%	100%	100%	93.7%	100%	100%	100%	100%	86.4%	99.0%	100%	100%	100%	100%	92%	94%	100%	80.6%	92.7%	99.0%	100%	89.1%	100%	100%	100%	100%	100%	100%	100%	100%	100%	100%	100%	75.4%	91.0%	100%	100%	100%	42	96.1%
**5000X Coverage (%)**																																																
Patient A	0.0%	100%	100%	100%	52.8%	100%	98.3%	100%	56.9%	100%	100%	62.7%	100%	0.0%	99.3%	0.0%	0.0%	0.0%	0.0%	0.0%	0.0%	78.7%	84.2%	72.5%	100%	0.0%	100%	100%	100%	100%	100%	100%	100%	92.3%	86.8%	100%	100%	100%	100%	100%	100%	0.0%	42.5%	100%	100%	100%	28	72.3%
Patient B	0.0%	100%	100%	100%	54.3%	100%	100%	100%	62.8%	100%	100%	64.1%	100%	0.0%	99.8%	30.6%	36.4%	0.3%	61.2%	55.3%	0.0%	81.3%	0.0%	0.0%	0.0%	0.0%	0.0%	0.0%	0.0%	0.0%	100%	100%	100%	87.5%	89.9%	100%	100%	100%	100%	100%	100%	0.0%	41.5%	100%	100%	100%	22	62.3%
Patient C	0.0%	100%	100%	100%	71.0%	100%	100%	100%	70.1%	100%	100%	67.6%	100%	34.0%	99.9%	100%	50.0%	22.4%	76.8%	70.0%	74.8%	74.8%	91.9%	98.2%	100%	0.0%	100%	100%	100%	100%	100%	100%	100%	100%	85.3%	100%	100%	100%	100%	78.3%	100%	0.0%	11.0%	100%	100%	79.9%	29	81.7%
Patient D	0.0%	100%	100%	100%	53.1%	100%	100%	100%	60.6%	100%	100%	63.4%	100%	0.0%	99.7%	0.0%	0.0%	0.0%	0.0%	0.0%	0.0%	63.2%	88.9%	75.4%	100%	0.0%	100%	100%	100%	100%	100%	100%	100%	97.1%	73.6%	100%	100%	100%	100%	100%	100%	0.0%	50.8%	100%	100%	100%	28	72.3%
Patient E	0.0%	100%	100%	100%	53.4%	100%	100%	100%	60.6%	100%	100%	63.4%	100%	0.0%	99.7%	12.5%	22.7%	0.0%	53.6%	47.6%	0.0%	72.9%	82.2%	64.1%	100%	0.0%	100%	100%	100%	100%	100%	100%	100%	100%	89.9%	100%	100%	100%	100%	90.8%	100%	0.0%	29.9%	100%	100%	82.6%	27	74.5%
**Min**	0.0%	100%	100%	100%	52.8%	100%	98.3%	100%	56.9%	100%	100%	62.7%	100%	0.0%	99.3%	0.0%	0.0%	0.0%	0.0%	0.0%	0.0%	63.2%	0.0%	0.0%	0.0%	0.0%	0.0%	0.0%	0.0%	0.0%	100%	100%	100%	87.5%	73.6%	100%	100%	100%	100%	78.3%	100%	0.0%	11.0%	100%	100%	79.9%	22	55.7%
**Max**	0.0%	100%	100%	100%	71.0%	100%	100%	100%	70.1%	100%	100%	67.6%	100%	34.0%	99.9%	100%	50.0%	22.4%	76.8%	70.0%	74.8%	81.3%	91.9%	98.2%	100%	0.0%	100%	100%	100%	100%	100%	100%	100%	100%	89.9%	100%	100%	100%	100%	100%	100%	0.0%	50.8%	100%	100%	100%	29	83.7%
**Mean**	0.0%	100%	100%	100%	56.9%	100%	100%	100%	62.2%	100%	100%	64.2%	100%	6.8%	99.7%	28.6%	21.8%	4.6%	38.3%	34.6%	15.0%	74.2%	69.4%	62.0%	80.0%	0.0%	80.0%	80.0%	80.0%	80.0%	100%	100%	100%	95.4%	85.1%	100%	100%	100%	100%	93.8%	100%	0.0%	35.1%	100%	100%	92.4%	27	72.6%
